# The impact of respiratory infections and probiotic use on the nasal microbiota of frail residents in long-term care homes

**DOI:** 10.1183/23120541.00212-2023

**Published:** 2023-09-25

**Authors:** Dawn M.E. Bowdish, Laura Rossi, Mark Loeb, Jennie Johnstone, Louis P. Schenck, Michelle Fontes, Michael G. Surette, Fiona J. Whelan

**Affiliations:** 1 Department of Medicine, McMaster University, Hamilton, ON, Canada; 2 M.G. DeGroote Institute for Infectious Disease Research, McMaster University, Hamilton, ON, Canada; 3 Firestone Institute for Respiratory Health, St Joseph's Healthcare Hamilton, Hamilton, ON, Canada; 4 Farncombe Family Digestive Health Research Institute, McMaster University, Hamilton, ON, Canada; 5 Department of Health Research Methods, Evidence & Impact, McMaster University, Hamilton, ON, Canada; 6 Department of Pathology and Molecular Medicine, McMaster University, Hamilton, ON, Canada; 7 Department of Laboratory Medicine & Pathobiology, University of Toronto, Toronto, ON, Canada; 8 Department of Biochemistry and Biomedical Sciences, McMaster University, Hamilton, ON, Canada; 9 School of Life Sciences, University of Nottingham, Nottingham, UK

## Abstract

**Background:**

Residents in long-term care homes, who tend to be of advanced age and frail, are at increased risk of respiratory infections. The respiratory microbiota is known to change with age, but whether these changes contribute to the risk of infection is not known. Our goal was to determine how the nasal microbiota of frail older adults changes during symptoms of influenza-like illness (ILI) and how this may be impacted by enrolment in a placebo-controlled trial testing the feasibility of administering a *Lactobacillus rhamnosus* GG probiotic to prevent respiratory infection (2014–2017).

**Methods:**

The microbiome of the nasal (mid-turbinate) of 150 residents of long-term care homes was interrogated using 16S rRNA gene sequencing.

**Results:**

We identified a diverse and individualised microbiota which could be separated into nine distinct clusters based on Bray–Curtis distances. Samples collected during symptoms of ILI differed statistically from those collected pre- and post-cold and influenza season, and we observed decreased temporal stability (as measured by movement between clusters) in individuals who experienced ILI compared to those who did not.

**Conclusions:**

The use of probiotics decreased ILI-induced changes to the microbiota; however, it is not clear whether this decrease is sufficient to prevent respiratory illness.

## Tweetable abstract

**This study identified nine distinct nasal microbiome “types” that changed when individuals experienced influenza-like illness, and that probiotic use decreased the frequency of these changes** https://bit.ly/476448v

## Background

The burden of respiratory infections in long-term care residents is high [1], and the devastating mortality and frequent outbreaks that occurred during the severe acute respiratory syndrome coronavirus 2 pandemic were a painful reminder that systemic features of care homes such as staffing patterns, ventilation and crowding can be major factors in infection rates [2, 3]. Independent of the increase in risk associated with long-term care homes, residents are still vulnerable to infection due to their advanced age, frailty and chronic health conditions. In fact, frailty is a better predictor of infection risk and poor outcomes than chronological age [4, 5], probably due to the systemic inflammation and immune remodelling that occurs in frail individuals [6].

Carriage rates of common pathogens like *Streptococcus pneumoniae* are counterintuitively reported to decrease with age, despite the fact that susceptibility to pneumococcal infection increases with age [7, 8]. It is believed that this is because pneumococcal carriage stimulates antibacterial immunity in the lungs; alveolar macrophages from individuals who are experimentally colonised have enhanced killing of both pneumococcus and other respiratory pathogens [9]. Age-related changes in other members of the airway microbiota have also been reported, and these may contribute to susceptibility to both bacterial and viral infections. As an example, individuals who are colonised with *Corynebacterium* spp*.* are less likely to naturally carry or be experimentally colonised with pneumococcus [10, 11]. Lower relative abundance of both *Moraxella* spp. and *Dolosigranulum pigrum* have been reported in children hospitalised for serious respiratory infections, but whether these are truly associated with protection from infection or whether they decrease in abundance during the course of infection is unclear [12, 13]. Similarly, older patients with pneumonia have outgrowth of some microbes in the upper respiratory tract microbiota [14], but the degree to which this contributes to infection is not known. Age, frailty, long-term care home residency, specific health conditions and immune senescence have been previously shown to be associated with age-associated changes in the gut microbiota [15, 16], but whether these factors influence the upper respiratory tract is not known. Understanding if members of the upper respiratory tract microbiota can protect against infection may provide novel preventative strategies in older, frail individuals who are the most likely to have poor outcomes resulting from respiratory infection.

In order to understand the role of the microbiota in respiratory infection in frail older adults, we analysed samples from 150 residents of long-term care homes who had been enrolled in a randomised, double-blinded, placebo-controlled clinical trial testing the feasibility of probiotics to prevent respiratory infection [17]. Samples were collected from individuals not experiencing respiratory illness at the onset of cold and influenza season (November–December), whenever a resident experienced an influenza-like illness (ILI) event, and after the cold and influenza season in the absence of illness (May–June). We investigated whether frailty, health conditions and systemic inflammation altered the composition of the nasal microbiota and whether there were features of the microbiota that predicted susceptibility to ILI. We found a diverse microbiota that could be divided into nine clusters. The microbiota of samples collected during ILI was statistically distinct from those collected outside of illness, and, when examined longitudinally, individuals who experienced symptoms of respiratory infection experienced decreased temporal stability of their nasal microbiota compared with those who did not. These affects appear to be mitigated with the use of probiotics; however, a larger follow-up study is warranted to reach a definitive conclusion.

## Methods

### Participant recruitment and sample collection

Samples were collected from individuals (n=150) as part of a multi-site, randomised, placebo-controlled trial on the feasibility of administering probiotics to prevent respiratory tract infections in long-term care residents [17]. This study represents a *post hoc* analysis of the samples collected as part of this randomised, placebo-controlled trial. Residents from 12 long-term care homes in Ontario, Canada who were aged ≥65 years were recruited over a 4-year period (2014–2017). Here, we sampled the nasal microbiota of samples collected from the later 3 years of the pilot study (supplementary figure S1). Participants provided a flocked nasal (mid-turbinate) swab in universal transport medium (Copan Italia, Brescia, Italy) prior to cold and influenza season (November–December), when they had symptoms consistent with an ILI (symptoms of ILI were assessed twice weekly by a trained nurse and included a temperature 1.5°C above baseline, cough, nasal congestion, sore throat, headache, sinus problems, muscle aches, fatigue, earache or infection or chills, as described by Wang *et al.* [17]) and following the end of cold and influenza season (May–June). Exclusion criteria included residents on immunosuppressive drugs; those who had haematological malignancy, structural heart disease, gastro-oesophageal or intestinal injury; or individuals who were at high risk of an endovascular infection. Participants were randomised to receive a probiotic (two capsules of *Lactobacillus rhamnosus* GG (Culturelle; CH Hansen, Hoersholm, Denmark; estimated 10 billion CFU per capsule) daily or a placebo (calcium carbonate) for 6 months. Details of probiotic administration have been published previously [17]. There were no differences in participant demographics between the placebo and probiotic groups (table 1) [17].

**TABLE 1:** Participant (n=150) characteristics across the dataset split by active and placebo arms of the trial

	**Active**	**Placebo**
**Female**	56 (73)	47 (64.4)
**Age years**	86±7.4	87±6.7
**Smoking history**		
Nonsmoker	33	38
Ex-smoker	40	33
Current smoker	3	2
Unknown	1	
**Number of medications**	9±3.2	9±4.0
**Number of comorbidities**	9±3.3	8±2.9
**Barthel score**	30±28.1	45±29.8
**Individuals reporting respiratory events**	21 (27.3)	30 (41.1)
**Events per individual (mean)**	1.11	1.16
**Probiotic group**	77 (51.3)	73 (48.7)

Data are presented as n (%), median±sd or n.

Informed consent was given by the participants or their substitute decision makers. All protocols were approved by the Hamilton integrated research ethics board. Following collection of flocked nasal swabs, swabs were stored in universal transport medium at −20°C until they were processed (described later).

Of the collected samples, 334 from 150 individuals (n=150 pre-cold and influenza season, 57 ILI, 127 post-cold and influenza season) passed stringent quality control measures including verification of the 16S rRNA gene PCR product on an agarose gel, quantification of 16S rRNA gene DNA load and a minimum number of high-quality DNA sequencing reads (described later in more detail).

### DNA extraction and amplification of the 16S rRNA gene

DNA extraction was performed as described previously [18]. 300 μL of universal transport medium (in which the nasal swab was stored) was combined with 800 μL of 200 mM sodium phosphate (monobasic) NaH_2_PO_4,_ 100 μL of guanidine thiocyanate-EDTA-Sarkosyl, and together homogenised using 0.2 g of 0.1-mm glass beads (Mo Bio, Carlsbad, CA, USA) for 3 min at 3000 rpm. 50 μL of lysozyme (100 mg·mL^−1^) and 10 μL RNase A (10 mg·mL^−1^) were added to the sample and incubated at 37°C for 1 h to enzymatically lyse the sample. Next, 25 μL of 25% SDS, 25 μL proteinase K and 62.5 μL 5 M sodium chloride were added and incubated at 65°C for 1 h. Samples were then subject to centrifugation at 12 000×*g*. The supernatant was subsequently removed to a new microcentrifuge tube to which an equal volume of phenol-chloroform-isoamyl alcohol was added and the sample again centrifuged. The solution with the lowest density was transferred to a new microcentrifuge tube and 200 μL of DNA binding buffer (Zymo, Irvine, CA, USA) added. The solution was then transferred to a DNA column (Zymo), washed, and DNA eluted using ultrapure H_2_O.

Following amplification of the 16S rRNA gene variable 3 (v3) region was performed as described previously [19] with some modifications. 341F and 518R 16S rRNA gene primers were adapted to the Illumina (San Diego, CA, USA) platform with the inclusion of unique six-base pair barcodes to the reverse primer to allow for multiplex amplification [19]. A 50 μL PCR reaction was performed in three equal-volume reactions, collectively containing 5 pmol of each primer, 200 μM of each deoxynucleoside triphosphate, 0.4 mg·mL^−1^ bovine serum albumin, 1.5 mM MgCl_2_ and 1 U Taq polymerase (Life Technologies, Carlsbad, CA, USA). The PCR reaction was subject to an initial denaturation step at 95°C for 5 min followed by 35 cycles of 95°C for 30 s, 50°C for 30 s and 72°C for 30 s; the incubation ended with an extension step at 72°C for 7 min. The presence of a PCR product was verified by electrophoresis (2% agarose gel) and only those samples with visible bands were sent for normalisation using the SequelPrep kit (ThermoFisher; #A1051001) and DNA sequencing on the Illumina MiSeq platform. A positive control sample of known community composition sequenced in parallel to these data contained the same 50 amplicon sequence variants (ASVs) in similar proportions as the positive control samples run on prior and subsequent MiSeq runs. Four negative controls, including DNA extraction and PCR controls, resulted in <1520 bacterial reads per sample, none of which were consistently assigned to the same ASVs (supplementary figure S2). All raw sequencing data is available on the *National Center for Biotechnology Information*'s Sequence Read Archive accession number PRJNA858212.

### Processing of 16S rRNA gene sequencing data

Raw reads were initially processed with Cutadapt [20] to trim the adapter and PCR primer sequences and filter to a minimum quality score of 30 and a minimum length of 100 bp. DADA2 [21] was used to resolve sequence variants for results from each separate Illumina run prior to merging data from all runs together. ASVs were then filtered for bimeras; taxons were classified using the SILVA database version 1.2.8 [22].

### Quantification of 16S rRNA gene DNA load *via* qPCR

Because samples from the nose have low microbial concentrations, we assessed the extracted DNA *via* quantitative (q)PCR in order to quantify the number of copies of 16S rRNA gene present in each sample. The protocol was adapted from [23]; briefly, reactions were carried out in a 96-well plate in a 20 μL mixture containing 10 pmol of forward (926F AAA CTC AAA KGA ATT GAC GG) and reverse (1062R CTC ACR RRC ACG AGC TGA C) primer [24], 1 μL of extracted swab DNA, 10 μg of bovine serum albumin, water and SsoFast EvaGreen supermix (Bio-Rad, Mississauga, ON, Canada). Samples were placed in a Bio-Rad CFX96 Thermocycler and were subject to an initial denaturing step (98°C for 2 min), followed by 40 cycles of 5 s at 98°C and 5 s at 60°C. Melt curve analysis was generated by 0.5°C increments for 5 s from 65°C to 95°C to ensure the generation of a single PCR product. Each reaction was performed in triplicate, with cycle thresholds converted to copies of 16S rRNA gene *via* standard curve of known quantities of *Escherichia coli* DNA within each qPCR plate. 12 samples which had <10^3^ copies of 16S rRNA gene sequence per sample were removed from all subsequent analyses.

### Statistical analyses of 16S rRNA gene sequencing data

The quality control measures described resulted in a total of 334 samples included in microbiome analyses. 39 metadata data points were collected; to avoid over-interpreting any correlations of such data with microbial composition, metadata variables were only considered if 1) ≤15% of the data points were unknown/missing; 2) for binary variables, there was ≥10% variation; 3) for nonbinary discrete variables, each value accounted for >3% of the overall variation (otherwise, the value was omitted). All continuous variables were included.

To identify any possible correlations between metadata variables and avoid reporting any indirect associations between metadata and microbial composition, each pair of variables were investigated using a Chi-squared or ANOVA test (depending on the data type). When the statistical test resulted in a p-value <0.05, we rejected the null hypothesis that the variables tested were independent. A list of all correlating variables is included in supplementary table S1.

All statistical analyses were performed in R v3.6.1 [25] primarily using the phyloseq v1.28.0 [26] and vegan v2.5.6 [27] packages. α-diversity was calculated using the Shannon index. β-diversity was determined using both Bray–Curtis and Aitchison distances (using R's microbiome package v1.14.0 [28] in addition to phyloseq); to calculate Bray–Curtis distances, the dataset was rarefied to the minimum number of reads per sample in the dataset (n=1268). The composition of microbiome communities in relation to included metadata variables was interrogated using a permanova statistical test (adonis function of the vegan package v2.5.6 [27]). Differentially abundant ASVs were determined using ANCOMBC [29]. Networks of cluster movement between pre-cold and influenza, ILI and post-cold and influenza clusters were determined using R's igraph v1.2.6 and visualised using Gephi [30]. R packages ggplot2 [31] and patchwork (https://github.com/thomasp85/patchwork) were used to generate visuals. All code is provided as a supplemental R markdown file (supplementary file S1).

Alluvial plots were generated using the R package ggalluvial [32]. When individuals had at least one ILI sample, the first was included in the summary graphic.

### Clustering of samples *via* hierarchical clustering

The Silhouette method, encoded in R's factoextra v1.0.7 package [33], was used to determine the optimal number of clusters for complete hierarchical clustering based on the Bray–Curtis distance between samples and associated principal coordinate analysis scores. Using this method, the optimal number of clusters was determined to be 10; of these, one cluster had a size of n=1 and was thus excluded from future analyses. Hierarchical clustering was performed using R's cluster v2.1.0 package [34]. The resulting nine clusters containing 334 samples were tested with the vegan package for statistically significant dispersion (betadisper [27]; p≤2.2×10^−16^), differences between cluster centroids (adonis [27]; p=0.001), and found to be significantly different in an analysis of similarity (anoism [27]; R=0.726, p=0.001), indicating that subsetting the data in this way generated statistically significant clusters. Similar analyses of clustering conducted with Aitchison distances similarly identifies statistically significant clustering (betadisper, p=4.543×10^−15^; adonis, p=0.001; anoism, R=0.168, p=0.001). A dendrogram of all samples was split into nine clusters with dendextend v1.15.1 [25] and visualised with ggtree v3.0.3 [35].

## Results

### Participant demographics

Mid-turbinate samples of the nose were collected from 150 individuals residing in long-term care homes in Ontario, Canada (described in the methods section). Participants were predominantly female, with a median age of 86.5 years (table 1). Individuals were on a median of nine medications and had a median of nine comorbidities. Participants' Barthel scores (an index between 0 and 20 used to describe performance in daily living [36]) were diverse, ranging from 0 to 20 with a median value of 7 (table 1). 34.0% of study participants reported symptoms of ILI, with 44 participants experiencing one event and seven participants experiencing two events. Participants in this trial were split into daily probiotic and placebo arms of this study which were evenly matched for all participant characteristics (table 1).

### The composition of the nasal microbiota of long-term care residents

**FIGURE 1:**
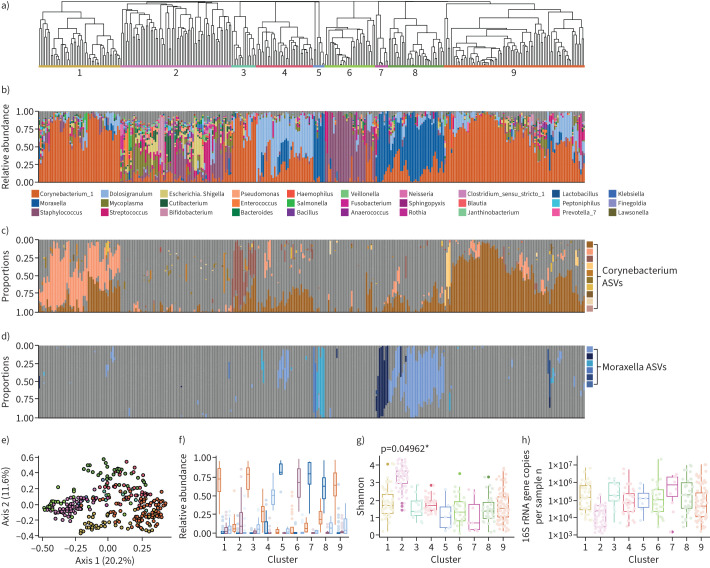
The nasal microbiota of frail older adults groups into nine distinct clusters. a) A dendrogram based on hierarchical clustering of Bray–Curtis distances between samples. Coloured and numbered bars indicate the nine clusters determined by hierarchical clustering. b) Taxonomic summaries ordered according to the sample order in the dendrogram accompanied by a legend of the most abundant 30 genera. c, d) The relative abundance of each amplicon sequence variant (ASV) within c) *Corynebacterium* and d) *Moraxella*. Each ASV is coloured a different shade of orange/blue; grey bars indicate relative abundances of other taxa. e) A principal coordinate analysis plot of the Bray–Curtis distances of all samples within the dataset coloured by cluster membership. Colours of each cluster match those used in panel a). f) The mean relative abundances of the four most abundant taxa separated by cluster. Colours of each taxon match those used in panel b): *Corynebacterium*: orange, *Moraxella*: dark blue, *Staphylococcus*: purple, *Dolosigranulum*: light blue. g) The median Shannon diversity metric differs significantly between clusters (p=0.04962; Levene's test); h) however, the median quantitative PCR concentrations do not (p=0.08108, Levene's test).

The nasal microbiota of long-term care residing older adults is highly variable (figure 1a,b). Within the 334 mid-turbinate samples analysed, we observed 720 genera, of which 662 had a cumulative relative abundance of ≥0.01%, and 122 of which were observed at ≥1% relative abundance in at least one sample. On average, an individual sample contained 56 genera; however, this ranged from seven to 146 (sd ±27.4). *Corynebacterium* is the most abundant and most prevalent genus, with a mean relative abundance of 37.9% (median 32.0%, range 0.04–98.7%), present in 332 of the 334 samples. Other abundant genera include *Moraxella* (mean relative abundance 1.18%, range 0.002–99.8%), *Staphylococcus* (mean relative abundance 1.17%, range 0.01–99.77%) and *Dolosigranulum* (mean relative abundance 1.11%, range 0.004–81.49%). The variability between individuals was large, as evidenced by a mean Bray–Curtis distance of 0.80 (median 0.87).

Because of the observed interindividual variability, we applied hierarchical clustering on the composition of the microbiome which identified nine clusters in our dataset (figure 1a) which were verified using multiple statistical tests (see methods section; supplementary figure S3A–C). Samples from each cluster separate in a principal coordinate analysis across multiple axes (figure 1e; supplementary figure S3D-G), and have more similar Bray–Curtis distances within clusters than comparisons across clusters (supplementary figure S3C,H). The median number of samples per cluster is 34, ranging from n=7 (cluster 5) to n=86 (cluster 9). All but one cluster is associated with a dominant taxon (>35% relative abundance in >70% of samples; figure 1b,f). For example, clusters 6 and 4 are associated with a high mean relative abundance of *Staphylococcus* and *Dolosigranulum*, respectively. There are three clusters (clusters 1, 3, 9) in which *Corynebacterium* is the dominant genus, but each is associated with a unique ASV (figure 1c). Similarly, *Moraxella* is the dominant genus of clusters 5, 7 and 8 and each cluster is associated with a particular profile of ASVs (figure 1d).

Uniquely, cluster 2 is not associated with a particular dominant taxon (figure 1f) and is the most diverse (Shannon index; figure 1g), and shared the least Bray–Curtis similarity across samples (supplementary figure S3H). We hypothesised that the absence of a dominant taxon might mean a decrease in the total bacterial load. When we quantified total microbial DNA by qPCR, we did not find a statistically significant difference in bacterial DNA between clusters although the median value was lower in cluster 2 than in any other cluster (figure 1h). However, when we investigated whether bacterial load significantly correlated with any particular ASV(s), we did not find a correlation with any dominant taxa. Instead, decreasing microbial load was associated with increased levels of other organisms also commonly associated with the microbiome of the oral cavity and skin (*e.g. Streptococcus* and *Cutibacterium*, respectively; supplementary figure S4). We hypothesise that this may mean that in samples with a low bacterial load, the unique biogeography of the mid-turbinates is lost, as we have shown previously in the nasopharyngeal microbiome of frail older adults [18].

### Participant characteristics and relationship to the composition of the airway microbiota

We tested 30 metadata variables (table 2), five of which (sex, long-term care home site, time of collection, frailty (as measured by the Barthel score), cardiovascular disease) were significantly correlated with the composition of the mid-turbinate microbiota against either of two β-diversity metrics employed (p≤0.05, PERMANOVA using either Bray–Curtis or Aitchison distance; table 2, see methods section). However, only time of collection passes multiple test correction (p≤0.00167, Bonferroni correction across 30 metadata variables) and none were significant across both β-diversity distance metrics and could each only explain <5% of the observed variance in the data (table 2); in contrast, 68.84% of the variability in the dataset was explained by inter–individual differences.

**TABLE 2:** p-values and R^2^ values of ANOVA statistical test of association between metadata variables and the composition of the microbiome.

	**By sample distance metrics (Bray–Curtis p-value (R^2^ value)/Aitchison p-value (R^2^ value))**	**By cluster membership (test statistic in brackets)**
**Age at enrolment**	0.224 (0.008)/0.821 (0.006)	0.295 (ANOVA)
**Sex**	0.047* (0.013)/0.149 (0.008)	0.248 (Chi-squared)
**Long-term care home site**	0.033* (0.082)/0.084 (0.068)	0.616 (Chi-squared)
**Month**	0.699 (0.018)/0.001* (0.034)	0.745 (Chi-squared)
**Season**	0.966 (0.003)/0.004* (0.011)	0.734 (Chi-squared)
**Year**	0.204 (0.017)/0.001* (0.032)	0.418 (Chi-squared)
**Clinical trial treatment group (*i.e.* probiotics or placebo)**	0.921 (0.004)/0.439 (0.007)	0.821 (Chi-squared)
**Had respiratory event**	0.402 (0.007)/0.079 (0.008)	0.121 (Chi-squared)
**Smoker**	0.782 (0.017)/0.367 (0.021)	0.788 (Chi-squared)
**Medications (number of)**	0.346 (0.007)/0.859 (0.006)	0.674 (ANOVA)
**Influenza vaccination (current season)**	0.837 (0.004)/0.307 (0.007)	0.689 (Chi-squared)
**Influenza vaccination (previous season)**	0.261 (0.015)/0.358 (0.014)	0.145 (Chi-squared)
**Influenza vaccination (ever)**	0.095 (0.019)/0.175 (0.015)	0.100 (Chi-squared)
**Pneumococcal vaccination (ever)**	0.689 (0.006)/0.586 (0.007)	0.891 (Chi-squared)
**Shared room (yes/no)**	0.480 (0.006)/0.370 (0.007)	0.478 (Chi-squared)
**Barthel total**	0.121 (0.01)/0.003* (0.011)	0.653 (ANOVA)
**COPD**	0.059 (0.011)/0.441 (0.007)	0.904 (Chi-squared)
**CHF**	0.365 (0.007)/0.825 (0.006)	0.638 (Chi-squared)
**CVD**	0.017* (0.015)/0.083 (0.009)	0.465 (Chi-squared)
**Anaemia**	0.923 (0.003)/0.673 (0.006)	0.974 (Chi-squared)
**Dementia**	0.912 (0.004)/0.597 (0.006)	0.438 (Chi-squared)
**Stroke**	0.074 (0.011)/0.449 (0.007)	0.201 (Chi-squared)
**Diabetes mellitus**	0.108 (0.01)/0.191 (0.007)	0.106 (Chi-squared)
**Hypothyroid**	0.957 (0.003)/0.863 (0.006)	0.998 (Chi-squared)
**Comorbidities (number of)**	0.765 (0.005)/0.337 (0.007)	0.891 (ANOVA)
**Seizures**	0.555 (0.006)/0.609 (0.006)	0.810 (Chi-squared)
**Cancer**	0.531 (0.006)/0.422 (0.007)	0.376 (Chi-squared)
**IL-1β**	0.887 (0.004)/0.830 (0.005)	0.483 (ANOVA)
**IL-6**	0.866 (0.004)/0.921 (0.005)	0.269 (Kruskal–Wallis)
**TNF-α**	0.652 (0.006)/0.990 (0.004)	0.177 (ANOVA)

Bold type indicates that values pass Bonferroni multiple test correction across the tested metadata variables. CHF: congestive heart failure; CVD: cardiovascular disease; IL: interleukin; TNF: tumour necrosis factor. *: p<0.05.

Biological sex significantly correlated with the composition of the microbiome (p=0.047, R^2^=0.013, PERMANOVA using Bray–Curtis); however, there was no association of β-diversity or cluster membership with age. In contrast, frailty correlated with microbiota composition (p=0.003, R^2^=0.011, PERMANOVA using Aitchison). Because chronic inflammation (“inflamm-ageing”) is associated with both frailty and immune dysfunction, we investigated whether there were any relationships with circulating inflammatory mediators, specifically tumour necrosis factor, interleukin (IL)-1β and IL-6 and chronic health conditions such as cardiovascular disease, dementia and COPD. Although there were no associations with inflammatory cytokines, community composition in individuals with cardiovascular disease was significantly different (p=0.017, R^2^=0.015, PERMANOVA using Bray–Curtis).

The composition of the microbiome differed between the 10 long-term care facilities (p=0.033, R^2^=0.082, PERMANOVA using Bray–Curtis), consistent with previous studies of the gut microbiota [16]. Nine ASVs with a mean relative abundance >0.1% were differentially abundant across long-term care sites (ANCOMBC); many of these ASVs correspond to the dominant taxa in the dataset, including *Moraxella*, *Corynebacterium* and *Dolosigranulum* (supplementary figure S5A–C). There were eight metadata variables that correlated with long-term care home site including those associated with the time of collection (*i.e.* month, season and year of collection) and other variables that probably reflect differences in long-term care home practices (*e.g.* influenza and pneumococcal vaccination, whether the resident was in a shared or private room; supplementary table S1). Of these, the time of sample collection also significantly correlated with microbiome composition (table 2). Five ASVs with >0.1% relative abundance were differentially abundant by month of sample collection, including the same *Moraxella* ASV which was differentially abundant by long-term care home site (supplementary figure S5D). Collectively, these data demonstrate that seasonality and long-term care home site may have some effect on the mid-turbinate microbiota.

### The effect of ILI on the composition of the nasal microbiota

**FIGURE 2:**
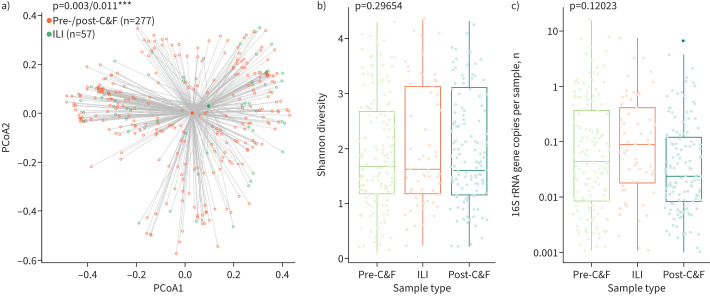
The composition, but not the α-diversity or bacterial load, of the frail older adult nasal microbiota is significantly altered during influenza-like illness (ILI) events. a) Community-wide, the microbiome composition changes significantly between samples collected during ILI *versus* times of relative health (p=0.003/0.0011, PERMANOVA with Bray–Curtis and Aitchison distances; principal coordinate analysis (PCoA) displayed is based on Bray–Curtis distances). b,c) However, there is no significant change in α-diversity (as measured by the Shannon metric, p=0.297, Levene's test; b) or bacterial load (as measured by quantitative PCR concentration, p=0.120, Levene's test; c) when samples collected during ILI were compared to those collected pre- and post-cold and influenza (C&F) season. ***: 0<p<0.001.

We compared the composition of the nasal microbiota when individuals had symptoms of ILI and when they did not. We found that the microbial community significantly differs between samples collected pre-/post-cold and influenza season compared to during ILI (p=0.003, R^2^=0.05 (Bray–Curtis); p=0.011, R^2^=0.04 (Aitchison) PERMANOVA; figure 2a). These results are supported by another recent comparison of the effect of ILI on the frail, aged nasopharyngeal microbiota [37]. α-Diversity, total bacterial load and cluster membership were not altered between ILI and non-ILI samples (p=0.297, Levene's test, figure 2b; p=0.120, Levene's test, figure 2c; p=0.148, Chi-squared test, supplementary figure S6). Furthermore, there was no difference in the composition or cluster membership of the pre-cold and influenza season microbiota between those who went on to experience ILI and those who did not (p=0.378, R^2^=0.007 (Bray–Curtis); p=0.095, R^2^=0.008 (Aitchison) PERMANOVA, supplementary figure S7A; p=0.121349, Chi-squared test, supplementary figure S7B), indicating that we cannot predict who will get an infection based on the composition of the microbiota alone.

Given that the composition of the microbiome changes with ILI, we next investigated whether this change could be attributed to specific ASVs. Using ANCOMBC [29], we identified eight ASVs that were differentially abundant between the pre-cold and influenza season, ILI and post-cold and influenza season; however, none of these has a mean relative abundance >0.1% (supplementary figure S8).

### A decrease in temporal stability of the nasal microbiota with ILI

Having determined that ILI affects the microbiome composition during illness, we next asked what effect ILI has following illness. Of those participants who reported symptoms of ILI during the study period, the composition of their microbiome before (pre-cold and influenza season) and after (post-cold and influenza season) ILI differed statistically from each other based on Aitchison (p=0.002, R^2^=0.015), but not Bray–Curtis (p=0.267, R^2^=0.009) distance or cluster membership (p=0.528, Chi-squared test). When the post-cold and influenza season composition of individuals who did and did not experience ILI were compared, the composition of the microbiome did not differ significantly (Aitchison, p=0.186, R^2^=0.009; Bray–Curtis, p=0.095, R^2^=0.012; cluster membership, p=0.133, Chi-squared test). Together, these results indicate that there is some effect of ILI on the microbiota following illness at the community level, but that this effect is not consistent enough to distinguish between individuals who had and had not experienced these respiratory events.

**FIGURE 3:**
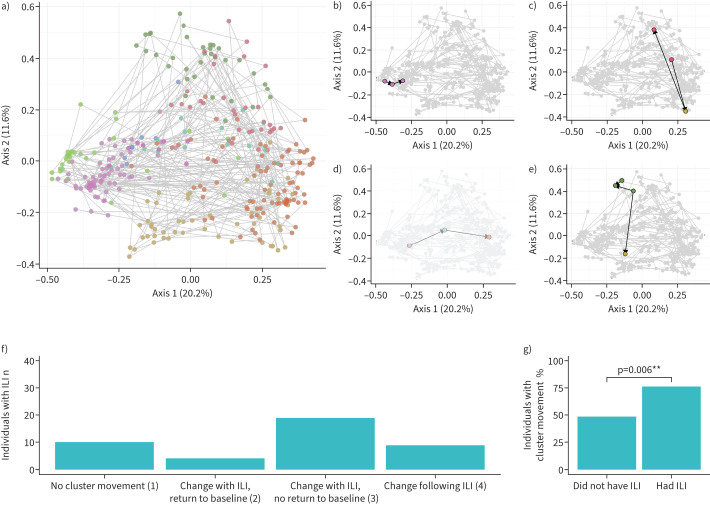
The longitudinal effect of influenza-like illness (ILI) on the frail older adult nasal microbiota. a) The principal coordinate analysis of all samples coloured by cluster with lines connecting longitudinal samples of each individual. Examples of individuals who b) stay in the same cluster throughout sampling (category 1), c) change cluster upon ILI, but return to their pre-ILI cluster upon resolution (category 2), d) change cluster upon ILI, but do not return to their original cluster (category 3) and e) change cluster following an ILI (category 4). f) Counts of individuals who fall into each of these cluster movement categories are quantified, and g) the frequency of cluster movement is compared to that of individuals who did not experience respiratory events (p=0.006, Chi-squared test).

When we analysed each individual (as opposed to focusing on community-wide metrics), we observed substantial changes to the microbiome during and following ILI. We tracked each individual's microbiota across principal coordinate analysis space and asked whether the rate of movement between clusters is affected by ILI. As shown previously, the dataset separates by cluster in a principal coordinate analysis; examining the chronological sampling of each individual, we see within-individual movement across principal coordinate analysis space (figure 3a). By focusing on individuals who experienced ILI (n=51), we define four movement categories: 1) individuals who stay in the same cluster before, during and after ILI (figure 3b); and individuals who move between clusters: 2) with ILI, but later returning to the pre-ILI cluster (figure 3c); 3) with ILI, but not returning to the pre-ILI cluster (figure 3d); and 4) following (but not during) ILI (figure 3e). These categories do not correlate with collected metadata (supplementary table S2); however, the within-individual mean Bray–Curtis distance are increased in categories which resulted in a permanent change in cluster membership (3 and 4) when compared to individuals who did not change clusters (category 1; supplementary figure S9), indicating the increased diversity between samples from individuals who experience significant cluster movement. 76.2% of individuals who experienced ILI moved between clusters, with 87.5% not returning to their original cluster by the end of the study period (categories 3 and 4; figure 3f). Importantly, individuals who experienced ILI were statistically more likely to move between clusters when compared to those who did not have respiratory infection (76.2% *versus* 48.7% of individuals; p=0.006, Chi-squared test; figure 3g). Movement between clusters was not predictable (*e.g.* there was no preference for a sample in a particular cluster to move to another at the next time point; supplementary figure S10). Together, these results indicate a significant legacy of change to the nasal microbiota associated with ILI events.

### The impact of probiotic use on ILI

There is no community-wide difference in the nasal microbiome between those on probiotic or placebo (p=0.13; R^2^=0.004 (Bray–Curtis); p=0.072; R^2^=0.004 (Aitchison)). Splitting individuals into those who did or did not experience ILI, there was also no difference between those on probiotic or placebo who experienced (p=0.438, R^2^=0.018 (Bray–Curtis); p=0.529, R^2^=0.017, (Aitchison)) or did not experience ILI (p=0.411; R^2^=0.005 (Bray–Curtis); p=0.357; R^2^=0.006 (Aitchison)). Similarly, cluster membership did not change significantly between probiotic or placebo use (p=0.220, Chi-squared test ILI; p=0.128 no ILI). Cluster membership nor microbiome composition were affected by the probiotic itself (supplementary figure S11).

**FIGURE 4:**
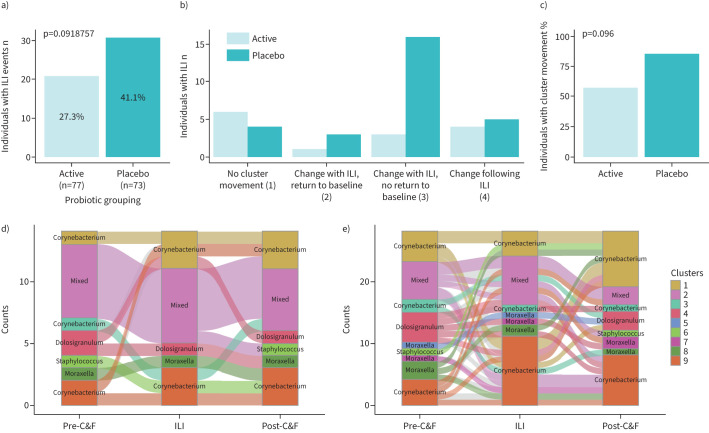
The effect of an oral *Lactobacillus rhamnosus GG* probiotic on influenza-like illness (ILI). a) There was no statistical difference in the number of individuals who experienced ILI between the probiotic (active) and placebo arms of the trial (p=0.0918757, Chi-squared test). b) The number of participants in each of the four cluster movement categories separated by whether they were part of the probiotic/active or placebo arms of the trial. c) The frequency of cluster movement between individuals receiving probiotic *versus* placebo treatments (p=0.096, Chi-squared test). d,e) Alluvial graph representation of cluster movement of individuals who experienced ILI on d) active and e) placebo treatment. Each cluster is labelled with the dominant genus that defines it; cluster 2 does not have a dominant genus and is instead labelled as a mixed community.

The number of individuals experiencing ILI and the mean number of reported ILI events per individual did not differ statistically between the active and placebo arms of this study, as reported previously [17] (p=0.092, Chi-squared test, figure 4a; p=0.589, t-test, data not shown). Similarly, the post-cold and influenza season microbiota did not differ statistically with probiotic use (p=0.458, R^2^=0.008 (Bray–Curtis); p=0.809, R^2^=0.007 (Aitchison); p=0.178, Chi-squared test). Collectively, this indicates that probiotic use does not affect the microbial composition of the nose during or following ILI in community-wide analyses.

In contrast, individuals who experienced ILI while receiving probiotic were less likely to move between clusters compared to those experiencing ILI on placebo treatment (figure 4b); 24 (85.7%) individuals on placebo treatment moved between clusters in comparison to eight (57.1%) individuals on probiotics (figure 4c). This observation is not statistically significant (p=0.096, Chi-squared test); further investigation of a larger cohort is needed to determine whether probiotic use could significantly affect the stability of the microbiome. In particular, there was more movement between clusters at the onset/resolution of ILI in individuals treated with placebo *versus* probiotic (figure 4d,e). This includes movement across clusters with different dominating genera; for example, individuals on placebo treatment go from cluster 3 (*Corynebacterium*), to cluster 2 (no dominant organism), to cluster 4 (*Dolosigranulum*) over the course of the study (figure 4e). These data indicate large overarching changes to the composition of these communities. Together, these results indicate that the administration of probiotics did not have an observable effect on the overall nasal microbiota and that further studies are needed to assess whether probiotics can mitigate the long-term impact of ILI on the individual.

## Discussion

Here we show that the diverse microbiota of frail, older residents of long-term care homes could be grouped into nine distinct clusters based on ASV presence and abundance. We find that the nasal microbiota of frail older adults exhibits an individualised response to ILI, often resulting in a lack of stability which is possibly mitigated, at least in part, by probiotic use.

The observed diversity of this community is perhaps not surprising given that inter-individual variability is also a feature of the ageing immune system, where a lifetime of environmental exposures and immune experiences shapes the immune response and age-associated inflammation [38]. Interestingly, age did not correlate with microbiota composition, but frailty did, results which are in line with frailty being a better indicator of infection risk than age [4, 5].

Although this study is unique in its focus on a more frail, long-term care dwelling cohort, previous studies have similarly identified changes to the respiratory tract microbiota in individuals who experience respiratory infection [39, 40]. In particular, a recent study of older (mean age 70 years) community-dwelling adults found similar distinctions between individuals experiencing ILI *versus* healthy controls [37]. Interestingly, this study found a difference in the stability of the microbiota post-ILI in individuals with higher abundances of core microbiota species (including *Corynebacterium*, *Dolosigranulum* and *Staphylococcus*) [37]; in contrast, we see no evidence of a difference in stability between individuals in clusters associated with or without a dominant taxon, perhaps suggesting that any protective effect of dominant taxa from ILI-induced changes to the microbiota in healthy older adults is weaker in this frail population.

We identified a microbiome that lacked stability and changed longitudinally in 76% of individuals with ILI. The microbiota did not change in a predictable way or converge on a particular ASV or cluster, but instead was highly individualised. These results indicate that the introduction of a pathogen, be it viral or bacterial, often leads to profound changes in the frail, aged nasal microbiota. The results of the pilot study of a probiotic targeted for the gastrointestinal tract suggest that it may be possible to mitigate these changes, at least in part. Only 21 (out of 77) individuals on probiotic treatment experienced ILI, and of those only eight moved between microbiome clusters; this is in contrast to 30 out of 73 individuals on placebo experiencing ILI with 24 of those 30 moving between clusters. This pilot trial is too small to be able to statistically conclude that probiotic use is beneficial to the stability of the nasal microbiota during and following ILI; furthermore, the use of antibiotics [17] was not investigated here due to the small sample size. Further investigation, perhaps with a nasal probiotic, is encouraged.

Our analyses identify nine distinct clusters of nasal microbial communities across this dataset. All but one of these clusters are associated with a dominant taxon (present at >35% relative abundance in >70% of samples), similar to that of the community-dwelling older adult microbiota [37]. Our use of hierarchical clustering outlined the four ASVs of *Corynebacterium* and five ASVs of *Moraxella* prevalent in the dataset. The ASVs of each species rarely co-occur within an individual (with the exception of cluster 1), suggesting possible intraspecies competition within this niche. This may have downstream implications on the ecology of these communities, especially when we consider that certain *Corynebacterium* and *Moraxella* ASVs were differentially abundant across various collected metadata (supplementary figure S5). Cluster 2 was unique in that it was not dominated by a particular taxon and that it was more diverse than the other clusters as measured by the Shannon diversity index. Interestingly, this increase in diversity correlated with a decrease in bacterial load (as measured by qPCR concentration), perhaps indicating the loss of a once-present prevalent taxon leaving only the less abundant, but highly diverse, taxa in its wake.

Of the tested metadata variables, we identified a correlation of microbial composition with long-term care home site, sex, time of collection, frailty and cardiovascular disease with either Bray–Curtis or Aitchison β-diversity metric. Variability of the microbiota with long-term care home is already well-established in the gut [16] and underlies known variations in management practices, air quality, diet, location, *etc*. between long-term care home sites. Similarly, changes to the nasal microbiota with the seasons has been documented previously in children [41], and year-to-year differences may represent the effect of circulating viruses (and variants thereof) on the nasal microbiota. Importantly, none of these correlations were found to be significant by both β-diversity metrics employed; here, we used the well-established Bray–Curtis metric due to its robustness and popularity in the field in addition to the compositionally aware Aitchison metric.

Preventing respiratory infection, and/or the long-term consequences thereof, in frail older adults will have an outsized impact on their care, quality of life and use of healthcare resources. Frailty, disability and loss of independence are exacerbated by having a respiratory infection [42], and hospitalisation rates (especially for strokes and cardiorespiratory events) increase months to years after infection [43]. Some, but not all vaccines, are less effective in frail individuals [44], so understanding the features of the frail microbiome and exploring new preventative measures are essential to reducing the burden of respiratory infections.

## Supplementary material

10.1183/23120541.00212-2023.Supp1 **Please note:** supplementary material is not edited by the Editorial Office, and is uploaded as it has been supplied by the author.
Supplementary material 00212-2023.SUPPLEMENT
R markdown 00212-2023.SUPPLEMENT2
