# Supplementary material for: The impact of respiratory infections and probiotic use on the nasal microbiota of frail residents in long-term care homes
Source: ERJ Open Res. 2023 Sep 25;9(5):00212-2023. doi: 10.1183/23120541.00212-2023 (PMC10518876; doi:10.1183/23120541.00212-2023)
Supplement: Supplementary file 1 [file 00212-2023.SUPPLEMENT.pdf]

Supplemental Material: The impact of respiratory infections and probiotic use on the nasal microbiota of frail residents in long-term care homes

Bowdish Dawn ME[1,2,3], Rossi Laura[1,4], Loeb Mark[2,5,6], Johnstone Jennie[7], Schenck Louis P[4,8], Fontes Michelle[1,4], Surette Michael G[1,2,4,8], & Whelan Fiona J[9*]

corresponding author
Dr. Fiona J Whelan
fiona.whelan@nottingham.ac.uk
B79 Life Sciences building,
University of Nottingham,
East Dr, Nottingham, NG7 2TQ
UK

Affiliations
[1] Department of Medicine, McMaster University
[2] M. G. DeGroote Institute for Infectious Disease Research McMaster University
[3] Firestone Institute for Respiratory Health, St. Joseph's Healthcare Hamilton
[4] Farncombe Family Digestive Health Research Institute, McMaster University
[5] Department of Health Research Methods, Evidence, & Impact, McMaster University
[6] Department of Pathology and Molecular Medicine, McMaster University
[7] Department of Laboratory Medicine & Pathobiology, University of Toronto
[8] Department of Biochemistry and Biomedical Sciences, McMaster University
[9] School of Life Sciences, University of Nottingham

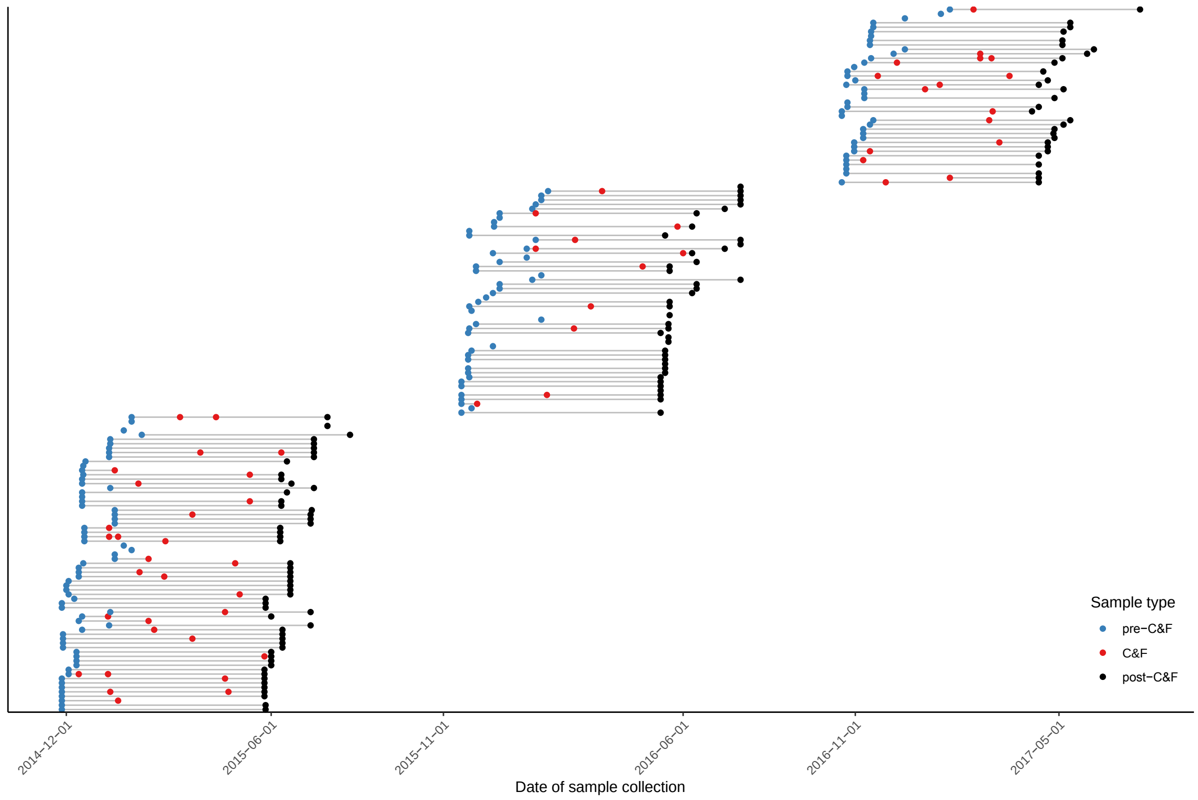

Supplemental Figure 1: A time series schematic of mid-turbinate sample collection. A time series of sample collection in this multi-year, longitudinal dataset (n=334). Labelled dates on the x-axis represent approximate start and end dates of collection for each cohort of samples. pre-C&F = pre-cold and influenza season; post-C&F = post-cold and influenza season; ILI = influenza like illness.

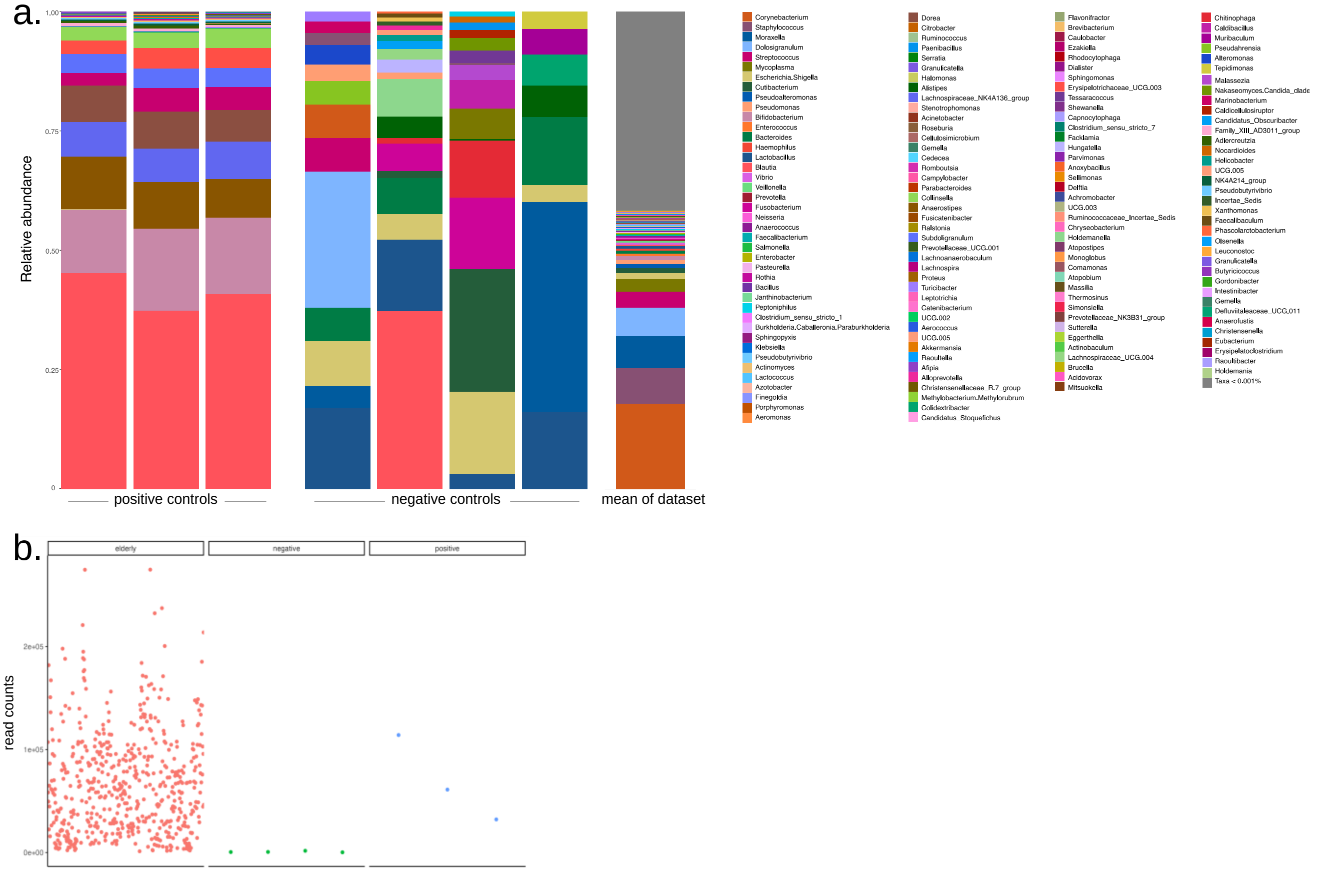

Supplemental Figure 2: Details of the control data versus the aging frail dataset. (a) Taxonomic summaries of 3 mock community positive control samples, 4 negative control samples, and the mean taxonomic diversity of the dataset. Positive samples remain consistent in their composition over sequencing runs, and negative controls do not contain any universal ASV and very few ASVs at very different proportions than the dataset itself. **(b)** Read counts of each sample from the dataset, negative, and positive controls.

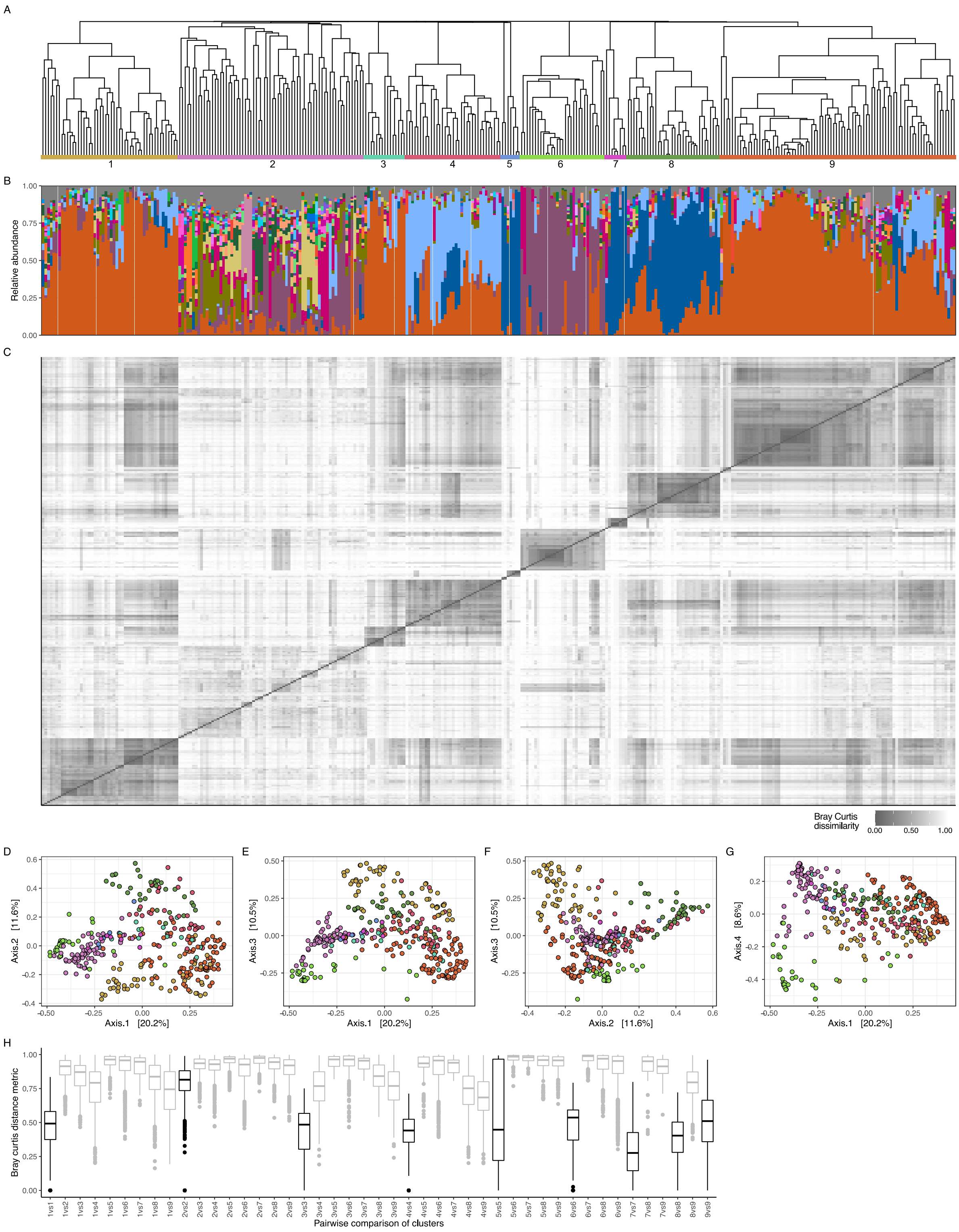

Supplemental Figure 3: **Evidence that 9 clusters is optimal. A-B**. The dendrogram and taxonomic summaries as shown in **Fig 1** redisplayed here for comparison. A detailed legend matching colours to genus-level taxonomic assignments is provided in **Sup Fig 3**. **C.** A heatmap of Bray Curtis distances between samples ordered as in **panels A-B**. **D-G**. PCoA analyses of various axes show separation of samples from each cluster across PCoA space. **H**. Median Bray

Curtis distances within (**black**) and between (**grey**) each cluster show more similarity within clusters (with the exception of cluster 2) than between clusters.

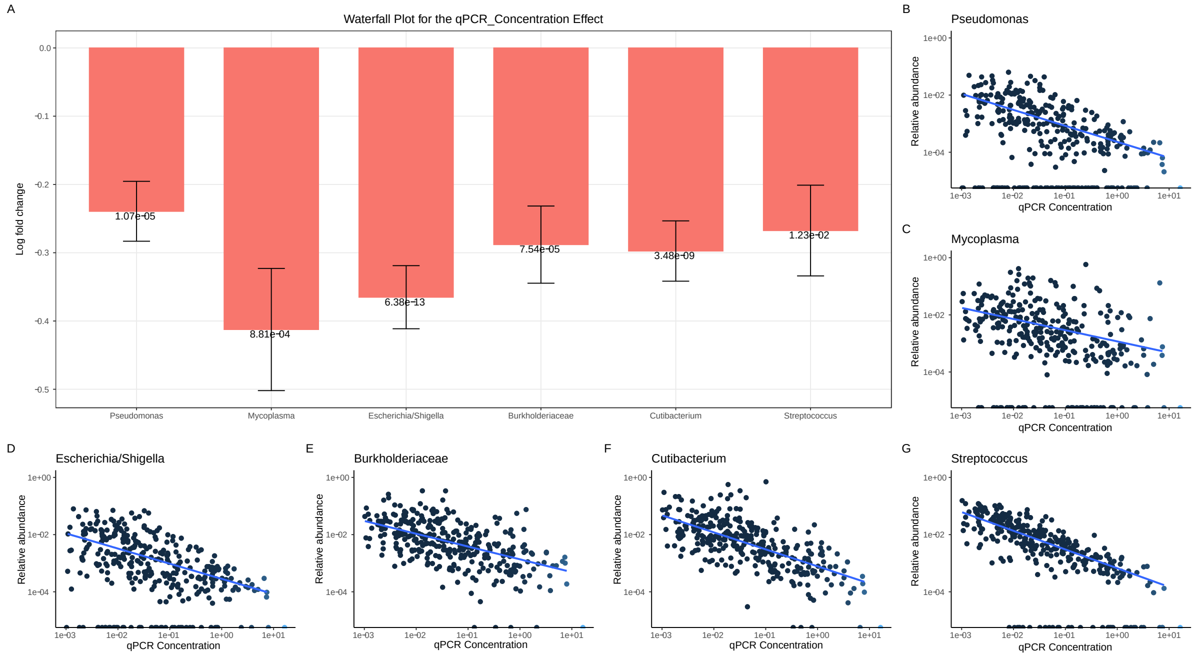

Supplemental Figure 4: ASVs which are differentially abundant with qPCR concentration. A. ANCOMBC was used to determine ASVs which were differentially abundant with qPCR concentration. Here, the log fold change of each differentially abundant ASV is displayed as well as the adjusted p-value. The genus (or family if the genus was undefined) taxonomic id is used to identify each ASV. **B-G.** The log-transformed qPCR concentration and relative abundance of each differentially abundant ASV is shown. A regression line with confidence intervals is shown in **blue.**

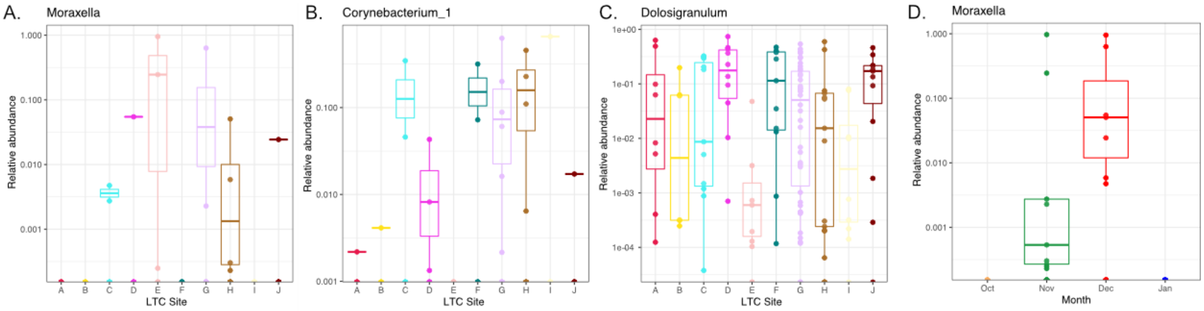

Supplemental Figure 5: ASVs which significantly correlate with particular metadata variables. A-C. When examining the LTC site, among the 9 differentially abundant ASVs with a mean relative abundance >0.1% include 3 taxa which are dominant across the dataset. **D.** Of the 5 differentially abundant ASVs across the month of collection, 2 also correlated with LTC site, including a *Moraxella* ASV.

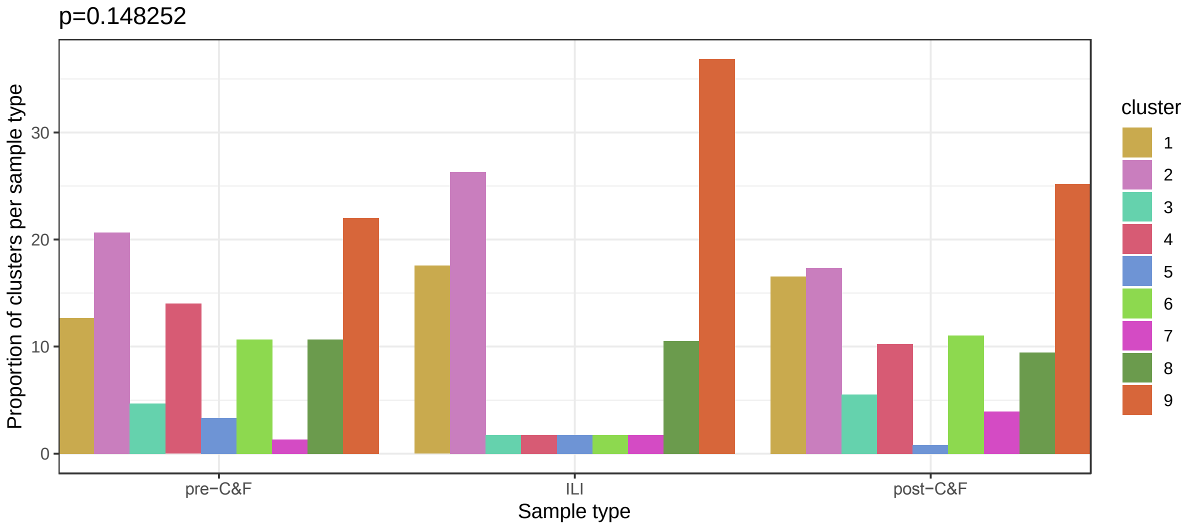

Supplemental Figure 6: **The proportion of pre-C&F, ILI, and post-C&F samples in each cluster type**. Samples collected at different points in the study period were not preferentially found in any particular cluster (p=0.148252, chi-squared test).

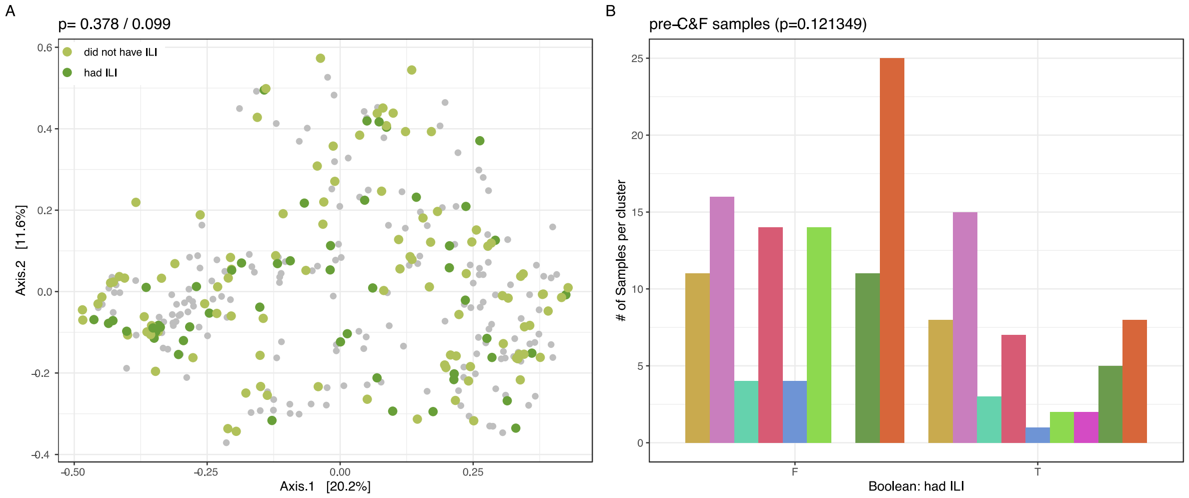

Supplemental Figure 7: Respiratory events are not predictable from *a priori* collected samples. A. There are no observable differences between pre-C&F samples collected before ILI events did or did not occur (p=0.378/0.099, permanova with Bray Curtis and Aitchison distances, respectively). **B.** Clustering of pre-C&F samples did not statistically differ between individuals who did and did not subsequently experience ILI (p=0.121349, chi-squared test).

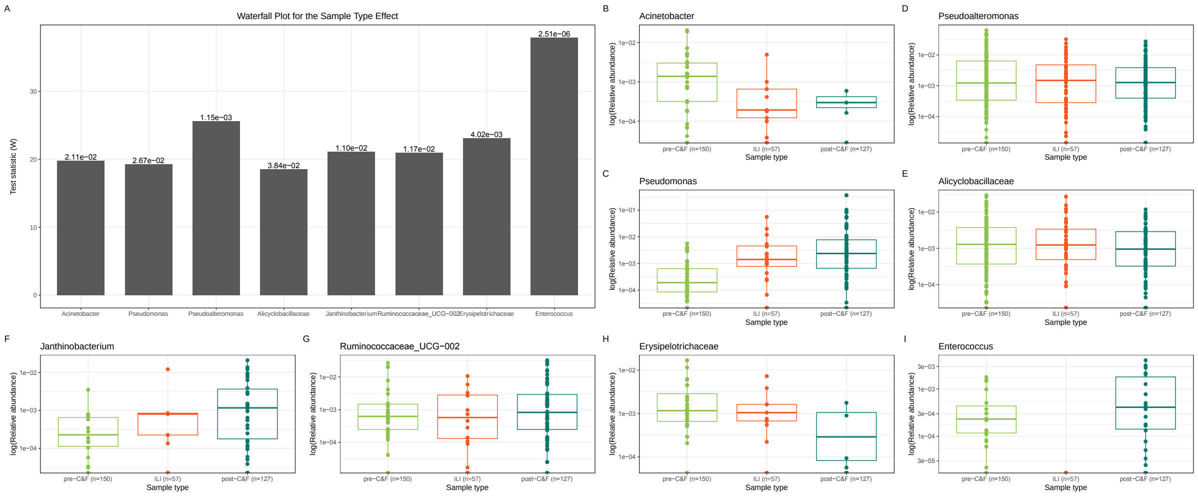

Supplemental Figure 8: ASVs which are differentially abundant across pre-C&F, ILI, and post-C&F sample types. A. ANCOMBC was used to determine the ASVs which were differentially abundant across the three sample types. Here, the test statistic (W) of each differentially abundant ASV is displayed along with the adjusted p-value. The genus (or family if the genus was undefined) taxonomic ID is used to identify each ASV. **B-I.** The log-transformed relative abundance of each differentially abundant ASV across the three sample types.

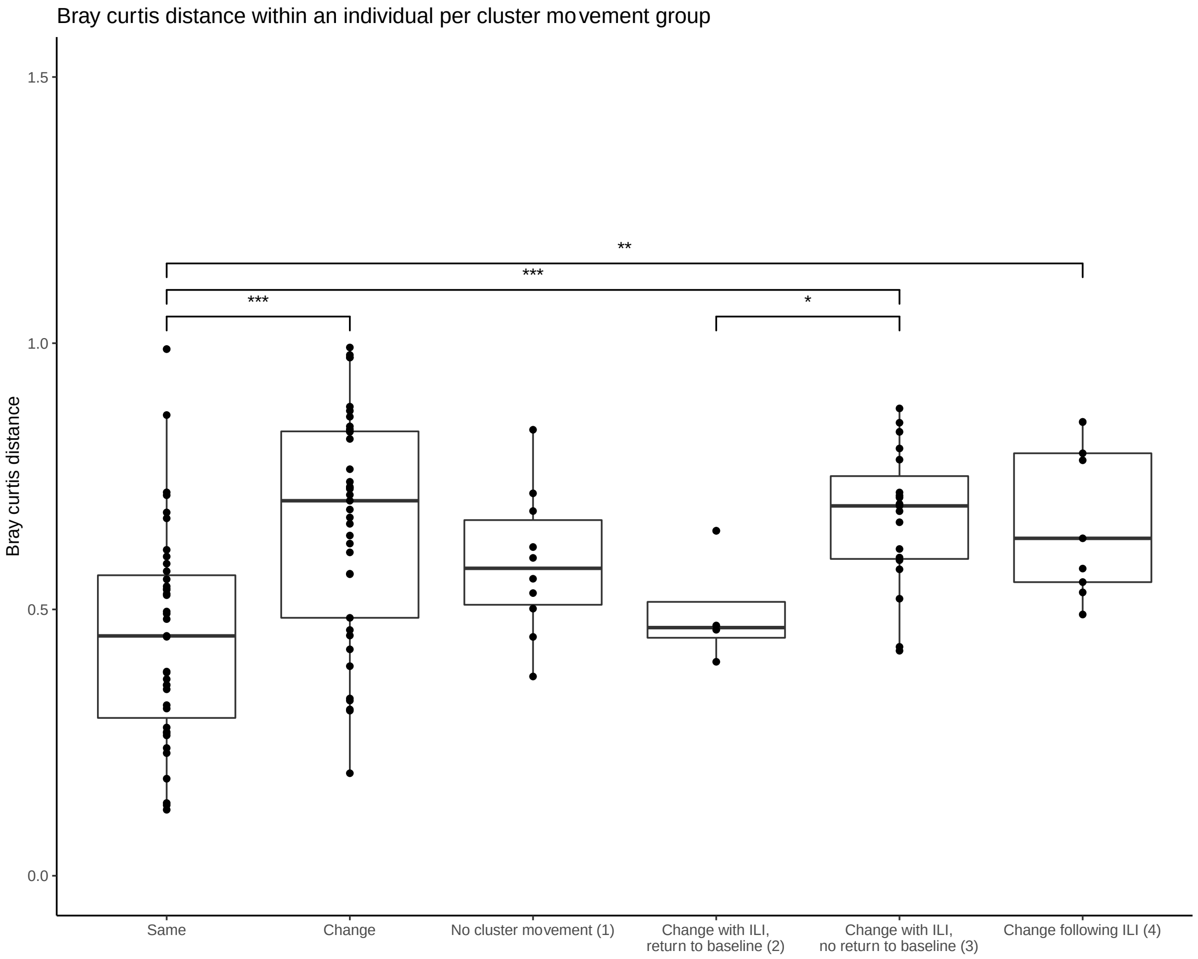

Supplemental Figure 9: The intra-individual Bray Curtis distance is increased when significant cluster movement is observed. In individuals who did not have

events, those whose microbiome moved between clusters (**“change”**) have a statistically significant increased Bray Curtis distance when compared to those who did not move between clusters (**“same”**). Similarly, individuals who experienced influenza-like illness (ILI) had an increased intra-individual Bray Curtis distance when the illness resulted in a permanent change in cluster membership (categories 3-4) when compared to individuals who did not change clusters (category 1).

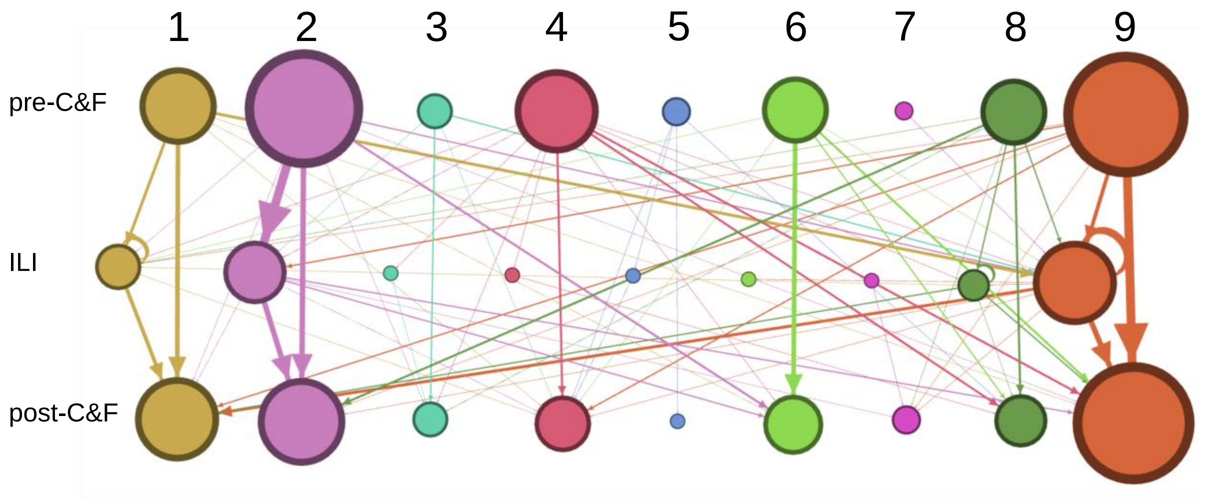

Supplemental Figure 10: There is no discernible pattern in cluster movement between samples. Nodes and edges are weighted based on the number of samples in each category. Rows and columns of nodes are labelled with the sample type (pre-C&F, ILI, post-C&F) and cluster number (1-9).

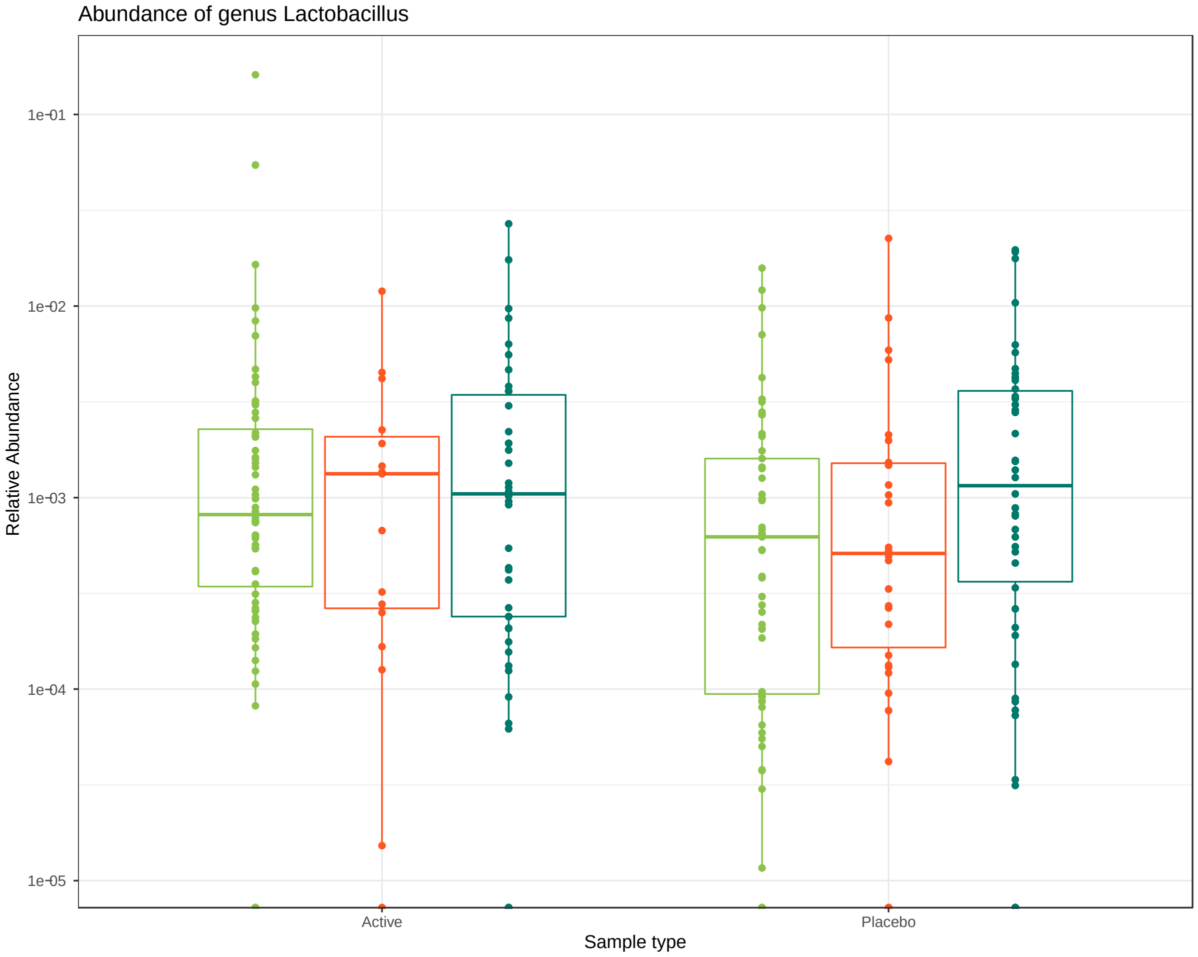

Supplemental Figure 11: Relative abundance of *Lactobacillus* across samples. There were no differentially abundant ASVs between individuals on active and placebo treatments, including any ASV with the taxonomic assignment of *Lactobacillus*.

Supplemental Table 1: Correlating metadata variables.

**Variable 1**	**Variable 2**	**Statistical test, p-value**
Age at enrolment	Sex	aov, p=0.040
Age at enrolment	Smoker	aov, p=0.0002
Age at enrolment	Influenza vaccination (current season)	aov, p=0.044
Age at enrolment	Anemia	aov, p=0.006
Age at enrolment	Seizures	aov, p=0.020
Site at enrolment	Month	chisq, p=2.519e-05
LTC Site	Season	chisq, p=0.009
LTC Site	Year	chisq, p=2.427e-13
LTC Site	Influenza vaccination (previous season)	chisq, p=0.026
LTC Site	Pneumococcal vaccine (ever)	chisq, p=0.001
LTC Site	Shared room	chisq, p=9.711e-10

LTC Site	Seizures	chisq, p=0.041
Month	Season	chisq, p < 2.2e-16
Month	Year	chisq, p=7.831e-16
Month	Smoker	chisq, p=0.0004
Month	Pneumococcal vaccine (ever)	chisq, p=0.024
Month	IL1B	aov, p=0.005
Season	Year	chisq, p=0.0003
Season	Pneumococcal vaccine (ever)	chisq, p=0.019
Season	Dementia	chisq, p=0.018
Season	IL1B	aov, p=0.027
Had respiratory event	IL1B	aov,p=0.042
Smoker	COPD	chisq, p=0.012
Smoker	IL1B	aov, p=0.050
Medications (number of)	COPD	aov, p=0.015
Medications (number of)	CHF	aov, p=0.0003
Medications (number of)	Dementia	aov, p=0.017
Medications (number of)	DM	aov, p=0.022
Medications (number of)	Comorbidities (number of)	aov, p=8.03e-07
Influenza vaccination (current season)	Influenza vaccination (previous season)	chisq, p=0.001
Influenza vaccination (current season)	Influenza vaccination (ever)	chisq, p=3.596e-08
Influenza vaccination (current season)	Pneumococcal vaccine (ever)	chisq, p=7.083e-05
Influenza vaccination (current season)	IL1B	aov, p=0.032
Influenza vaccination (previous season)	Influenza vaccination (ever)	chisq, p=7.785e-10
Influenza vaccination (previous season)	Pneumococcal vaccine (ever)	chisq, p=0.001
Influenza vaccination (previous season)	Comorbidities (number of)	aov, p=0.014
Influenza vaccination (ever)	Pneumococcal vaccine (ever)	chisq, p=0.003
Influenza vaccination (ever)	Barthel total	aov, p=0.037
Influenza vaccination (ever)	Comorbidities (number of)	aov, p=0.044
Influenza vaccination (ever)	IL1B	aov, p=0.015
Pneumococcal vaccine (ever)	Cancer	chisq, p= 0.029
COPD	CVD	chisq, p=0.002
CHF	Comorbidities (number of)	aov, p=0.002
CVD	Comorbidities (number of)	aov, p=2.56e-06
Anemia	Comorbidities (number of)	aov,p= 0.008
Dementia	IL6	aov, p=0.027

Stroke	Comorbidities (number of)	aov, p=0.017
Diabetes mellitus	Comorbidities (number of)	aov, p=0.045

Supplemental Table 2: p-values of correlation tests (per individual) between collected metadata variables and cluster movement categories.

Characteristic	p-value	Statistical test
Age at enrolment	0.312	aov
Sex	0.123	chisq
LTC home site	0.722	chisq
Allocation Group Probiotics	0.091	chisq
Smoker	0.192	chisq
Num Medications	0.326	aov
Influenza vacc this season	0.743	aov
Influenza vacc last season	0.419	chisq
Influenza vaccine ever	0.447	chisq
Has pt received pneumonia vaccine	0.290	chisq
Is pt in shared room	0.985	chisq
Barthel total	0.668	aov
COPD	0.190	chisq
CHF	0.148	chisq
CVD	0.424	chisq
Anemia	0.206	chisq
Dementia	0.312	chisq
CVA Stroke	0.123	chisq
DM	0.403	chisq
Hypothyroid	0.176	chisq
Num Comorbidities	0.725	aov
Seizures	0.309	chisq
Cancer	0.468	chisq
IL1B	0.438	aov
IL6	0.569	aov
TNFA	0.675	aov

Correlation was determined either with a chi squared or aov test, depending on the data type of the variable. COPD: chronic obstructive pulmonary disease, CHF: congestive heart failure, CVD: cardiovascular disease.
