# Supplementary material for: The impact of respiratory infections and probiotic use on the nasal microbiota of frail residents in long-term care homes
Source: ERJ Open Res. 2023 Sep 25;9(5):00212-2023. doi: 10.1183/23120541.00212-2023 (PMC10518876; doi:10.1183/23120541.00212-2023)
Supplement: Supplementary file 2 [file 00212-2023.SUPPLEMENT2.pdf]

````
---
title: "LTC-probiotic-manuscript-Figure-script"
output: html_document
editor_options:
  chunk_output_type: console
---

A complete start to finish of data analysis post-dada2 processing to
produce all Figures and statistics in the LTC-probiotic manuscript.

```{r setup, include=FALSE}
knitr::opts_chunk$set(echo = TRUE)
```

# Setup script

Setup: load libraries, check packageVersions, set ggplot theme
```{r load, include=FALSE}
library("phyloseq")
library("plyr")
library("vegan")
library("ggplot2")
library("cowplot")
library("data.table")
library("lmerTest")
library("RColorBrewer")
library("hash")
library("car")
library("psych")
library("patchwork")
library("DESeq2")
library("ANCOMBC")
library("pheatmap")
library("ggplotify")
library("cluster")
library("factoextra")
library("zoo")
library("dplyr")
library("tidytree")
library("ggsignif")
library("ape")
library("MASS")
library("corrplot")
library("microbiome")
library("factoextra")
library("NbClust")
library("dendextend")
library("stringr")
library("ggcorrplot")
library("tibble")
library("Biobase")
#also requires ggtree, treeio; called in line later in the script

packageVersion("phyloseq")
````
````

````
packageVersion("plyr")
packageVersion("vegan")
packageVersion("ggplot2")
packageVersion("cowplot")
packageVersion("data.table")
packageVersion("lmerTest")
packageVersion("RColorBrewer")
packageVersion("hash")
packageVersion("car")
packageVersion("psych")
packageVersion("patchwork")
packageVersion("DESeq2")
packageVersion("ANCOMBC")
packageVersion("pheatmap")
packageVersion("ggplotify")
packageVersion("cluster")
packageVersion("factoextra")
packageVersion("zoo")
packageVersion("dplyr")
packageVersion("tidytree")
packageVersion("ggsignif")
packageVersion("ape")
packageVersion("MASS")
packageVersion("corrplot")
packageVersion("microbiome")
packageVersion("factoextra")
packageVersion("NbClust")
packageVersion("dendextend")
packageVersion("stringr")
packageVersion("ggcorrplot")
packageVersion("tibble")
packageVersion("Biobase")

theme_set(theme_classic())
```

Setup: define colours & labels for L6 taxa (dada2_L6 function)
```{r colours, include=FALSE}
dada2_L6 <- function(x) {
  #Setup hash
  h <- hash()
  h["Corynebacterium_1"] = "#d15919" #009ce4"
  h["Staphylococcus"] = "#875073"
  h["Moraxella"] = "#005b9e"
  h["Dolosigranulum"] = "#7eb5ff"
  h["Streptococcus"] = "#c7006f"
  h["Mycoplasma"] = "#7a7800"
  h["Escherichia.Shigella"] = "#d5c870"
  h["Cutibacterium"] = "#245d3a"
  h["Pseudoalteromonas"] = "#025bb0"
  h["Pseudomonas"] = "#ff9e73"
  h["Bifidobacterium"] = "#c888a8"
  h["Enterococcus"] = "#ff772b"
  h["Bacteroides"] = "#007c45"
````

```
h["Haemophilus"] = "#f14735"
h["Lactobacillus"] = "#1c558d"
h["Vibrio"] = "#d95fe4"
h["Veillonella"] = "#6add7f"
h["Blautia"] = "#ff525b"
h["Fusobacterium"] = "#cd0498"
h["Neisseria"] = "#fb5cd7"
h["Anaerococcus"] = "#8d1a94"
h["Faecalibacterium"] = "#02abaf"
h["Salmonella"] = "#1ebc42"
h["Pasteurella"] = "#f2b1eb"
h["Tyzzerella_4"] = "#d96900"
h["Rothia"] = "#c30b9d"
h["Bacillus"] = "#7132a5"
h["Janthinobacterium"] = "#77da98"
h["Burkholderia.Caballeronia.Paraburkholderia"] = "#e2aaff"
h["Peptoniphilus"] = "#01d2e9"
h["Clostridium_sensu_stricto_1"] = "#f177fd"
h["Distigma"] = "#647441"
h["Prevotella_7"] = "#ff50ae"
h["Klebsiella"] = "#006dda"
h["Sphingopyxis"] = "#931a80"
h["Actinomyces"] = "#eebf71"
h["Pseudobutyrivibrio"] = "#6fcbff"
h["Lactococcus"] = "#52c4ff"
h["Azotobacter"] = "#fab5a1"
h["Finegoldia"] = "#8794ff"
h["Porphyromonas"] = "#c15100"
h["Eisenbergiella"] = "#0275ca"
h["Aeromonas"] = "#ff8a3d"
h["Dorea"] = "#8b4f41"
h["Lawsonella"] = "#665e2b"
h["Paenibacillus"] = "#0293ed"
h["Serratia"] = "#00830c"
h["Halomonas"] ="#c8cc5e"
h["Citrobacter"] = "#c56100"
h["Alistipes"] = "#006205"
h["Stenotrophomonas"] = "#ffaba0"
h["Lachnospiraceae_NK4A136_group"] = "#6167f0"
h["Acinetobacter"] = "#92331d"
h["Granulicatella"] = "#7d59e2"
h["Malassezia"] = "#b449cf"
h["Prevotella_9"] = "#007df5"
h["Raoultella"] = "#029efb"
h["Gemella"] = "#3a8166"
h["Roseburia"] = "#895f3b"
h["Romboutsia"] = "#d42eaf"
h["Cedecea"] = "#53d5fe"
h["Campylobacter"] = "#ff58a6"
h["Parabacteroides"] = "#bd3400"
h["Collinsella"] = "#91d957"
h["Fusicatenibacter"] = "#9a4900"
h["Anaerostipes"] = "#895500"
h["Prevotella"] = "#9f1e32"
```

```
h["Enterobacter"] = "#b3b300"
h["Ruminococcus_2"] = "#35c94e"
h["Ralstonia"] = "#a48d00"
h["Subdoligranulum"] = "#5d71fc"
h["Corynebacterium"] = "#90d5ad"
h["Proteus"] = "#a06100"
h["Lachnospira"] = "#b6007d"
h["Turicibacter"] = "#9e7cff"
h["Prevotellaceae_UCG.001"] = "#355c1d"
h["Ruminococcus_1"] = "#98d332"
h["Leptotrichia"] = "#ff63b9"
h["Catenibacterium"] = "#ff73ce"
h["Coprococcus_1"] = "#01b7ca"
h["Prevotella_2"] = "#784702"
h["Ruminococcaceae_UCG.002"] = "#966f47"
h["Ruminococcaceae_UCG.005"] = "#a10262"
h["Aerococcus"] = "#295ae0"
h["Akkermansia"] = "#e47a00"
h["Ruminococcaceae_UCG.014"] = "#007142"
h["Alloprevotella"] = "#ee289c"
h["Ezakiella"] = "#b4006b"
h["Ruminiclostridium_9"] = "#d88e9b"
h["Christensenellaceae_R.7_group"] = "#705900"
h["Methylobacterium"] = "#7ac7ad"
h["Candidatus_Stoquefichus"] = "#ff9ee3"
h["Flavonifractor"] = "#95a670"
h["Brevibacterium"] = "#f4bd68"
h["Caulobacter"] = "#9f1947"
h["Rhodocytophaga"] = "#b3000d"
h["Erysipelotrichaceae_UCG.003"] = "#ff4f46"
h["Shewanella"] = "#80308b"
h["Capnocytophaga"] = "#a99fff"
h["Clostridium_sensu_stricto_7"] = "#00816d"
h["Comamonas"] = "#995561"
h["Pluralibacter"] = "#b459e1"
h["Facklamia"] = "#008126"
h["Parvimonas"] = "#8b22ab"
h["Hungatella"] = "#bcb4ff"
h["Anoxybacillus"] = "#f66327"
h["Delftia"] = "#b50022"
h["Coprococcus_2"] = "#646e00"
h["Sellimonas"] = "#ea8b00"
h["Simonsiella"] = "#ee234b"
h["Ruminococcaceae_UCG.003"] = "#a36a52"
h["Chryseobacterium"] = "#ff6abf"
h["Holdemanella"] = "#8dd78d"
h["Ruminococcaceae_UCG.013"] = "#e26700"
h["Atopobium"] = "#80d7b6"
h["Lachnoclostridium_5"] = "#cf2d17"
h["Ruminiclostridium"] = "#942c47"
h["Eggerthella"] = "#abd532"
h["Intestinimonas"] = "#1cdc6c"
h["Actinobaculum"] = "#53cb46"
h["Acetatifactor"] = "#b90060"
```

```
h["Ensifer"] = "#f5bd5a"
h["Zea"] = "#d43dbe"
h["Acidovorax"] = "#ff57c3"
h["Kingella"] = "#8a3744"
h["Mitsuokella"] = "#863e11"
h["Peptostreptococcus"] = "#ff985b"
h["Odoribacter"] = "#ff99c6"
h["Actinobacillus"] = "#5dd9d3"
h["Lachnospiraceae_UCG.001"] = "#dd3c20"
h["Lachnoanaerobaculum"] = "#0273da"
h["Abiotrophia"] = "#ff5950"
h["Phenylobacterium"] = "#fb298b"
h["Erysipelatoclostridium"] = "#a0016a"
h["Negativicoccus"] = "#01d3b6"
h["Lachnospiraceae_UCG.006"] = "#bdaa00"
h["Phormidesmis_ANT.LACV5.1"] = "#2d69f2"
h["A2"] = "#006996"
h["Deinococcus"] = "#b07d00"
h["Pseudoglutamicibacter"] = "#b0008b"
h["Cloacibacterium"] = "#0080aa"
h["Selenomonas_3"] = "#f8b1da"
h["Actinotignum"] = "#792ba7"
h["Clostridium_sensu_stricto_12"] = "#84da78"
h["Ruminococcaceae_UCG.004"] = "#3791ff"
h["Sphingomonas"] = "#eb85ff"
h["Ileibacterium"] = "#4ae088"
h["Muribaculum"] = "#a50096"
h["Aeribacillus"] = "#dd007f"
h["Oceanobacillus"] = "#0285ea"
h["Tepidicella"] = "#ffa126"
h["Oligella"] = "#91d867"
h["Ornithinibacillus"] = "#e1c556"
h["Filifactor"] = "#4c5902"
h["Photobacterium"] = "#ff9076"
h["Tyzzerella_3"] = "#82404b"
h["Micrococcus"] = "#932f39"
h["Catonella"] = "#3ce187"
h["Brevundimonas"] = "#b77600"
h["Porphyrobacter"] = "#f00a6d"
h["Trueperella"] = "#0c601c"
h["Tibeticola"] = "#4dd253"
h["Weissella"] = "#4a591d"
h["possible_genus_Sk018"] = "#ff4b72"
h["Oscillibacter"] = "#a525ae"
h["Taxa < 1%"] = "#808080"
h["other"] = "#808080"
#Make a vector of colours to return
colours <- character(0)
for (i in 1:length(x)){
    col <- h[[x[i]]]
    #print(col)
    if (is.null(col)) {
      print(x[i])
    }
```

```
      colours <- c(colours, col)
  }
  return(colours)
}

#myLabels_L6 function
myLabels_L6 <- function(x,y) {
  #Setup hash
  h <- hash()

h["Root.p__Actinobacteria.c__Actinobacteria.o__Actinomycetales.f__Ac
tinomycetaceae.g__Actinobaculum"] = paste("g__Actinobaculum (",
format(round(100*mean(y$Root.p__Actinobacteria.c__Actinobacteria.o__
Actinomycetales.f__Actinomycetaceae.g__Actinobaculum),2),
nsmall=2),"%)", sep="")

h["Root.p__Actinobacteria.c__Actinobacteria.o__Actinomycetales.f__Ac
tinomycetaceae.g__Actinomyces"] = paste("g__Actinomyces (",
format(round(100*mean(y$Root.p__Actinobacteria.c__Actinobacteria.o__
Actinomycetales.f__Actinomycetaceae.g__Actinomyces),2),
nsmall=2),"%)", sep="")

h["Root.p__Actinobacteria.c__Actinobacteria.o__Coriobacteriales.f__C
oriobacteriaceae.g__Adlercreutzia"] = paste("g__Adlercreutzia (",
format(round(100*mean(y$Root.p__Actinobacteria.c__Actinobacteria.o__
Actinomycetales.f__Actinomycetaceae.g__Adlercreutzia),2),
nsmall=2),"%)", sep="")

h["Root.p__Actinobacteria.c__Actinobacteria.o__Actinomycetales.f__Ac
tinomycetaceae.g__Arcanobacterium"] = paste("g__Arcanobacterium (",
format(round(100*mean(y$Root.p__Actinobacteria.c__Actinobacteria.o__
Actinomycetales.f__Actinomycetaceae.g__Arcanobacterium),2),
nsmall=2),"%)", sep="")

h["Root.p__Actinobacteria.c__Actinobacteria.o__Actinomycetales.f__Br
evibacteriaceae.g__Brevibacterium"] = paste("g__Brevibacterium (",
format(round(100*mean(y$Root.p__Actinobacteria.c__Actinobacteria.o__
Actinomycetales.f__Brevibacteriaceae.g__Brevibacterium),2),
nsmall=2),"%)", sep="")

h["Root.p__Actinobacteria.c__Actinobacteria.o__Actinomycetales.f__Co
rynebacteriaceae.g__"] = paste("f__Corynebacteriaceae (",
format(round(100*mean(y$Root.p__Actinobacteria.c__Actinobacteria.o__
Actinomycetales.f__Corynebacteriaceae.g__),2), nsmall=2),"%)",
sep="")

h["Root.p__Actinobacteria.c__Actinobacteria.o__Actinomycetales.f__Co
rynebacteriaceae.g__Corynebacterium"] = paste("g__Corynebacterium
(",
format(round(100*mean(y$Root.p__Actinobacteria.c__Actinobacteria.o__
Actinomycetales.f__Corynebacteriaceae.g__Corynebacterium),2),
nsmall=2),"%)", sep="")

h["Root.p__Actinobacteria.c__Actinobacteria.o__Actinomycetales.f__In
```

```
trasporangiaceae.g__"] = paste("f__Intrasporangiaceae (",
format(round(100*mean(y$Root.p__Actinobacteria.c__Actinobacteria.o__
Actinomycetales.f__Intrasporangiaceae.g__),2), nsmall=2),"%)",
sep="")

h["Root.p__Actinobacteria.c__Actinobacteria.o__Actinomycetales.f__Mi
crococcaceae.g__Rothia"] = paste("g__Rothia (",
format(round(100*mean(y$Root.p__Actinobacteria.c__Actinobacteria.o__
Actinomycetales.f__Micrococcaceae.g__Rothia),2), nsmall=2),"%)",
sep="")

h["Root.p__Actinobacteria.c__Actinobacteria.o__Actinomycetales.f__Mi
cromonosporaceae.Other"] = paste("f__Micromonosporaceae (",
format(round(100*mean(y$Root.p__Actinobacteria.c__Actinobacteria.o__
Actinomycetales.f__Micromonosporaceae.Other),2), nsmall=2),"%)",
sep="")

h["Root.p__Actinobacteria.c__Actinobacteria.o__Actinomycetales.f__Pr
omicromonosporaceae.g__Cellulosimicrobium"] =
paste("g__Cellulosimicrobium (",
format(round(100*mean(y$Root.p__Actinobacteria.c__Actinobacteria.o__
Actinomycetales.f__Promicromonosporaceae.g__Cellulosimicrobium),2),
nsmall=2),"%)", sep="")

h["Root.p__Actinobacteria.c__Actinobacteria.o__Actinomycetales.f__Pr
opionibacteriaceae.g__Propionibacterium"] =
paste("g__Propionibacterium (",
format(round(100*mean(y$Root.p__Actinobacteria.c__Actinobacteria.o__
Actinomycetales.f__Propionibacteriaceae.g__Propionibacterium),2),
nsmall=2),"%)", sep="")

h["Root.p__Actinobacteria.c__Actinobacteria.o__Actinomycetales.f__Pr
opionibacteriaceae.Other"] = paste("f__Propionibacteriaceae (",
format(round(100*mean(y$Root.p__Actinobacteria.c__Actinobacteria.o__
Actinomycetales.f__Propionibacteriaceae.Other),2), nsmall=2),"%)",
sep="")

h["Root.p__Actinobacteria.c__Actinobacteria.o__Actinomycetales.Other
.Other"] = paste("o__Actinomycetales (",
format(round(100*mean(y$Root.p__Actinobacteria.c__Actinobacteria.o__
Actinomycetales.Other.Other),2), nsmall=2),"%)", sep="")

h["Root.p__Actinobacteria.c__Actinobacteria.o__Bifidobacteriales.f__
Bifidobacteriaceae.g__Bifidobacterium"] = paste("g__Bifidobacterium
(",
format(round(100*mean(y$Root.p__Actinobacteria.c__Actinobacteria.o__
Bifidobacteriales.f__Bifidobacteriaceae.g__Bifidobacterium),2),
nsmall=2),"%)", sep="")

h["Root.p__Actinobacteria.c__Actinobacteria.o__Bifidobacteriales.f__
Bifidobacteriaceae.g__Gardnerella"] = paste("g__Gardnerella (",
format(round(100*mean(y$Root.p__Actinobacteria.c__Actinobacteria.o__
Bifidobacteriales.f__Bifidobacteriaceae.g__Gardnerella),2),
nsmall=2),"%)", sep="")
```

```
h["Root.p__Actinobacteria.c__Actinobacteria.o__Bifidobacteriales.f__
Bifidobacteriaceae.g__Scardovia"] = paste("g__Scardovia (",
format(round(100*mean(y$Root.p__Actinobacteria.c__Actinobacteria.o__
Bifidobacteriales.f__Bifidobacteriaceae.g__Scardovia),2),
nsmall=2),"%)", sep="")

h["Root.p__Actinobacteria.c__Actinobacteria.o__Coriobacteriales.f__C
oriobacteriaceae.g__Collinsella"] = paste("g__Collinsella (",
format(round(100*mean(y$Root.p__Actinobacteria.c__Actinobacteria.o__
Coriobacteriales.f__Coriobacteriaceae.g__Collinsella),2),
nsmall=2),"%)", sep="")
  h["Root.p__Bacteroidetes.c__Bacteroidia.o__Bacteroidales.f__.g__"]
= paste("o__Bacteroidales (",
format(round(100*mean(y$Root.p__Bacteroidetes.c__Bacteroidia.o__Bact
eroidales.f__.g__),2), nsmall=2),"%)", sep="")

h["Root.p__Bacteroidetes.c__Bacteroidia.o__Bacteroidales.f__Bacteroi
daceae.g__Bacteroides"] = paste("g__Bacteroides (",
format(round(100*mean(y$Root.p__Bacteroidetes.c__Bacteroidia.o__Bact
eroidales.f__Bacteroidaceae.g__Bacteroides),2), nsmall=2),"%)",
sep="")

h["Root.p__Bacteroidetes.c__Bacteroidia.o__Bacteroidales.f__Porphyro
monadaceae.g__"] = paste("f__Porphyromonadaceae (",
format(round(100*mean(y$Root.p__Bacteroidetes.c__Bacteroidia.o__Bact
eroidales.f__Porphyromonadaceae.g__),2), nsmall=2),"%)", sep="")

h["Root.p__Bacteroidetes.c__Bacteroidia.o__Bacteroidales.f__Porphyro
monadaceae.g__Parabacteroides"] = paste("g__Parabacteroides (",
format(round(100*mean(y$Root.p__Bacteroidetes.c__Bacteroidia.o__Bact
eroidales.f__Porphyromonadaceae.g__Parabacteroides),2),
nsmall=2),"%)", sep="")

h["Root.p__Bacteroidetes.c__Bacteroidia.o__Bacteroidales.f__Porphyro
monadaceae.g__Porphyromonas"] = paste("g__Porphyromonas (",
format(round(100*mean(y$Root.p__Bacteroidetes.c__Bacteroidia.o__Bact
eroidales.f__Porphyromonadaceae.g__Porphyromonas),2),
nsmall=2),"%)", sep="")

h["Root.p__Bacteroidetes.c__Bacteroidia.o__Bacteroidales.f__Prevotel
laceae.g__Prevotella"] = paste("g__Prevotella (",
format(round(100*mean(y$Root.p__Bacteroidetes.c__Bacteroidia.o__Bact
eroidales.f__Prevotellaceae.g__Prevotella),2), nsmall=2),"%)",
sep="")

h["Root.p__Bacteroidetes.c__Bacteroidia.o__Bacteroidales.f__Rikenell
aceae.g__Alistipes"] = paste("g__Alistipes (",
format(round(100*mean(y$Root.p__Bacteroidetes.c__Bacteroidia.o__Bact
eroidales.f__Rikenellaceae.g__Alistipes),2), nsmall=2),"%)", sep="")

h["Root.p__Bacteroidetes.c__Flavobacteria.o__Flavobacteriales.f__Fla
vobacteriaceae.g__Capnocytophaga"] = paste("g__Capnocytophaga (",
format(round(100*mean(y$Root.p__Bacteroidetes.c__Flavobacteria.o__Fl
```

```
avobacteriales.f__Flavobacteriaceae.g__Capnocytophaga),2),
nsmall=2),"%)", sep="")

h["Root.p__Bacteroidetes.c__Flavobacteria.o__Flavobacteriales.f__Fla
vobacteriaceae.g__Chryseobacterium"] = paste("g__Chryseobacterium
(",
format(round(100*mean(y$Root.p__Bacteroidetes.c__Flavobacteria.o__Fl
avobacteriales.f__Flavobacteriaceae.g__Chryseobacterium),2),
nsmall=2),"%)", sep="")

h["Root.p__Bacteroidetes.c__Sphingobacteria.o__Sphingobacteriales.f_
_Flexibacteraceae.g__"] = paste("f__Flexibacteraceae (",
format(round(100*mean(y$Root.p__Bacteroidetes.c__Sphingobacteria.o__
Sphingobacteriales.f__Flexibacteraceae.g__),2), nsmall=2),"%)",
sep="")

h["Root.p__Bacteroidetes.c__Sphingobacteria.o__Sphingobacteriales.f_
_Sphingobacteriaceae.g__Sphingobacterium"] =
paste("g__Sphingobacterium (",
format(round(100*mean(y$Root.p__Bacteroidetes.c__Sphingobacteria.o__
Sphingobacteriales.f__Sphingobacteriaceae.g__Sphingobacterium),2),
nsmall=2),"%)", sep="")
  h["Root.p__Chloroflexi.c__SOGA31.o__.f__.g__"] = paste("c__SOGA31
(",
format(round(100*mean(y$Root.p__Chloroflexi.c__SOGA31.o__.f__.g__),2
), nsmall=2),"%)", sep="")
  h["Root.p__Crenarchaeota.Other.Other.Other.Other"] =
paste("p__Crenarchaeota (",
format(round(100*mean(y$Root.p__Crenarchaeota.Other.Other.Other.Othe
r),2), nsmall=2),"%)", sep="")
  h["Root.p__Cyanobacteria.c__Chloroplast.o__Streptophyta.f__.g__"]
= paste("o__Streptophyta (",
format(round(100*mean(y$Root.p__Cyanobacteria.c__Chloroplast.o__Stre
ptophyta.f__.g__),2), nsmall=2),"%)", sep="")

h["Root.p__Cyanobacteria.c__Nostocophycideae.o__Nostocales.f__Nostoc
aceae.Other"] = paste("f__Nostocaceae (",
format(round(100*mean(y$Root.p__Cyanobacteria.c__Nostocophycideae.o_
_Nostocales.f__Nostocaceae.Other),2), nsmall=2),"%)", sep="")

h["Root.p__Cyanobacteria.c__Synechococcophycideae.o__Pseudanabaenale
s.f__Pseudanabaenaceae.g__Halomicronema"] = paste("g__Halomicronema
(",
format(round(100*mean(y$Root.p__Cyanobacteria.c__Synechococcophycide
ae.o__Pseudanabaenales.f__Pseudanabaenaceae.g__Halomicronema),2),
nsmall=2),"%)", sep="")

h["Root.p__Firmicutes.c__Bacilli.o__Bacillales.f__Bacillaceae.g__Ano
xybacillus"] = paste("g__Anoxybacillus (",
format(round(100*mean(y$Root.p__Firmicutes.c__Bacilli.o__Bacillales.
f__Bacillaceae.g__Anoxybacillus),2), nsmall=2),"%)", sep="")

h["Root.p__Firmicutes.c__Bacilli.o__Bacillales.f__Bacillaceae.g__Bac
illus"] = paste("g__Bacillus (",
```

```
format(round(100*mean(y$Root.p__Firmicutes.c__Bacilli.o__Bacillales.
f__Bacillaceae.g__Bacillus),2), nsmall=2),"%)", sep="")

h["Root.p__Firmicutes.c__Bacilli.o__Bacillales.f__Paenibacillaceae.g
__Paenibacillus"] = paste("g__Paenibacillus (",
format(round(100*mean(y$Root.p__Firmicutes.c__Bacilli.o__Bacillales.
f__Paenibacillaceae.g__Paenibacillus),2), nsmall=2),"%)", sep="")

h["Root.p__Firmicutes.c__Bacilli.o__Bacillales.f__Staphylococcaceae.
g__Staphylococcus"] = paste("g__Staphylococcus (",
format(round(100*mean(y$Root.p__Firmicutes.c__Bacilli.o__Bacillales.
f__Staphylococcaceae.g__Staphylococcus),2), nsmall=2),"%)", sep="")

h["Root.p__Firmicutes.c__Bacilli.o__Gemellales.f__Gemellaceae.g__Gem
ella"] = paste("g__Gemella (",
format(round(100*mean(y$Root.p__Firmicutes.c__Bacilli.o__Gemellales.
f__Gemellaceae.g__Gemella),2), nsmall=2),"%)", sep="")

h["Root.p__Firmicutes.c__Bacilli.o__Lactobacillales.f__Aerococcaceae
.g__Aerococcus"] = paste("g__Aerococcus (",
format(round(100*mean(y$Root.p__Firmicutes.c__Bacilli.o__Lactobacill
ales.f__Aerococcaceae.g__Aerococcus),2), nsmall=2),"%)", sep="")

h["Root.p__Firmicutes.c__Bacilli.o__Lactobacillales.f__Aerococcaceae
.g__Facklamia"] = paste("g__Facklamia (",
format(round(100*mean(y$Root.p__Firmicutes.c__Bacilli.o__Lactobacill
ales.f__Aerococcaceae.g__Facklamia),2), nsmall=2),"%)", sep="")

h["Root.p__Firmicutes.c__Bacilli.o__Lactobacillales.f__Carnobacteria
ceae.g__"] = paste("f__Carnobacteriaceae (",
format(round(100*mean(y$Root.p__Firmicutes.c__Bacilli.o__Lactobacill
ales.f__Carnobacteriaceae.g__),2), nsmall=2),"%)", sep="")

h["Root.p__Firmicutes.c__Bacilli.o__Lactobacillales.f__Carnobacteria
ceae.g__Granulicatella"] = paste("g__Granulicatella (",
format(round(100*mean(y$Root.p__Firmicutes.c__Bacilli.o__Lactobacill
ales.f__Carnobacteriaceae.g__Granulicatella),2), nsmall=2),"%)",
sep="")

h["Root.p__Firmicutes.c__Bacilli.o__Lactobacillales.f__Enterococcace
ae.g__Enterococcus"] = paste("g__Enterococcus (",
format(round(100*mean(y$Root.p__Firmicutes.c__Bacilli.o__Lactobacill
ales.f__Enterococcaceae.g__Enterococcus),2), nsmall=2),"%)", sep="")

h["Root.p__Firmicutes.c__Bacilli.o__Lactobacillales.f__Lactobacillac
eae.g__Lactobacillus"] = paste("g__Lactobacillus (",
format(round(100*mean(y$Root.p__Firmicutes.c__Bacilli.o__Lactobacill
ales.f__Lactobacillaceae.g__Lactobacillus),2), nsmall=2),"%)",
sep="")

h["Root.p__Firmicutes.c__Bacilli.o__Lactobacillales.f__Leuconostocac
eae.g__Weissella"] = paste("g__Weissella (",
format(round(100*mean(y$Root.p__Firmicutes.c__Bacilli.o__Lactobacill
ales.f__Leuconostocaceae.g__Weissella),2), nsmall=2),"%)", sep="")
```

```
h["Root.p__Firmicutes.c__Bacilli.o__Lactobacillales.f__Streptococcac
eae.g__Lactococcus"] = paste("g__Lactococcus (",
format(round(100*mean(y$Root.p__Firmicutes.c__Bacilli.o__Lactobacill
ales.f__Streptococcaceae.g__Lactococcus),2), nsmall=2),"%)", sep="")

h["Root.p__Firmicutes.c__Bacilli.o__Lactobacillales.f__Streptococcac
eae.g__Streptococcus"] = paste("g__Streptococcus (",
format(round(100*mean(y$Root.p__Firmicutes.c__Bacilli.o__Lactobacill
ales.f__Streptococcaceae.g__Streptococcus),2), nsmall=2),"%)",
sep="")

h["Root.p__Firmicutes.c__Bacilli.o__Lactobacillales.f__Streptococcac
eae.Other"] = paste("f__Streptococcaceae (",
format(round(100*mean(y$Root.p__Firmicutes.c__Bacilli.o__Lactobacill
ales.f__Streptococcaceae.Other),2), nsmall=2),"%)", sep="")

h["Root.p__Firmicutes.c__Bacilli.o__Turicibacterales.f__Turicibacter
aceae.g__"] = paste("f__Turicibacteraceae (",
format(round(100*mean(y$Root.p__Firmicutes.c__Bacilli.o__Turicibacte
rales.f__Turicibacteraceae.g__),2), nsmall=2),"%)", sep="")

h["Root.p__Firmicutes.c__Bacilli.o__Turicibacterales.f__Turicibacter
aceae.g__Turicibacter"] = paste("g__Turicibacter (",
format(round(100*mean(y$Root.p__Firmicutes.c__Bacilli.o__Turicibacte
rales.f__Turicibacteraceae.g__Turicibacter),2), nsmall=2),"%)",
sep="")
  h["Root.p__Firmicutes.c__Clostridia.o__Clostridiales.f__.g__"] =
paste("o__Clostridiales (",
format(round(100*mean(y$Root.p__Firmicutes.c__Clostridia.o__Clostrid
iales.f__.g__),2), nsmall=2),"%)", sep="")

h["Root.p__Firmicutes.c__Clostridia.o__Clostridiales.f__Catabacteria
ceae.g__"] = paste("f__Catabacteriaceae (",
format(round(100*mean(y$Root.p__Firmicutes.c__Clostridia.o__Clostrid
iales.f__Catabacteriaceae.g__),2), nsmall=2),"%)", sep="")

h["Root.p__Firmicutes.c__Clostridia.o__Clostridiales.f__Clostridiace
ae.g__Clostridium"] = paste("g__Clostridium (",
format(round(100*mean(y$Root.p__Firmicutes.c__Clostridia.o__Clostrid
iales.f__Clostridiaceae.g__Clostridium),2), nsmall=2),"%)", sep="")

h["Root.p__Firmicutes.c__Clostridia.o__Clostridiales.f__Clostridiace
ae.Other"] = paste("f__Clostridiaceae (",
format(round(100*mean(y$Root.p__Firmicutes.c__Clostridia.o__Clostrid
iales.f__Clostridiaceae.Other),2), nsmall=2),"%)", sep="")

h["Root.p__Firmicutes.c__Clostridia.o__Clostridiales.f__Clostridiale
sFamilyXI.IncertaeSedis.g__"] =
paste("f__ClostridialesFamilyXI.IncertaeSedis (",
format(round(100*mean(y$Root.p__Firmicutes.c__Clostridia.o__Clostrid
iales.f__ClostridialesFamilyXI.IncertaeSedis.g__),2),
nsmall=2),"%)", sep="")
```

```
h["Root.p__Firmicutes.c__Clostridia.o__Clostridiales.f__Clostridiale
sFamilyXI.IncertaeSedis.g__Anaerococcus"] = paste("g__Anaerococcus
(",
format(round(100*mean(y$Root.p__Firmicutes.c__Clostridia.o__Clostrid
iales.f__ClostridialesFamilyXI.IncertaeSedis.g__Anaerococcus),2),
nsmall=2),"%)", sep="")

h["Root.p__Firmicutes.c__Clostridia.o__Clostridiales.f__Clostridiale
sFamilyXI.IncertaeSedis.g__Finegoldia"] = paste("g__Finegoldia (",
format(round(100*mean(y$Root.p__Firmicutes.c__Clostridia.o__Clostrid
iales.f__ClostridialesFamilyXI.IncertaeSedis.g__Finegoldia),2),
nsmall=2),"%)", sep="")

h["Root.p__Firmicutes.c__Clostridia.o__Clostridiales.f__Clostridiale
sFamilyXI.IncertaeSedis.g__Peptoniphilus"] = paste("g__Peptoniphilus
(",
format(round(100*mean(y$Root.p__Firmicutes.c__Clostridia.o__Clostrid
iales.f__ClostridialesFamilyXI.IncertaeSedis.g__Peptoniphilus),2),
nsmall=2),"%)", sep="")

h["Root.p__Firmicutes.c__Clostridia.o__Clostridiales.f__Lachnospirac
eae.g__"] = paste("f__Lachnospiraceae (",
format(round(100*mean(y$Root.p__Firmicutes.c__Clostridia.o__Clostrid
iales.f__Lachnospiraceae.g__),2), nsmall=2),"%)", sep="")

h["Root.p__Firmicutes.c__Clostridia.o__Clostridiales.f__Lachnospirac
eae.g__Blautia"] = paste("g__Blautia (",
format(round(100*mean(y$Root.p__Firmicutes.c__Clostridia.o__Clostrid
iales.f__Lachnospiraceae.g__Blautia),2), nsmall=2),"%)", sep="")

h["Root.p__Firmicutes.c__Clostridia.o__Clostridiales.f__Lachnospirac
eae.g__Catonella"] = paste("g__Catonella (",
format(round(100*mean(y$Root.p__Firmicutes.c__Clostridia.o__Clostrid
iales.f__Lachnospiraceae.g__Catonella),2), nsmall=2),"%)", sep="")

h["Root.p__Firmicutes.c__Clostridia.o__Clostridiales.f__Lachnospirac
eae.g__Clostridium"] = paste("g__Clostridium (",
format(round(100*mean(y$Root.p__Firmicutes.c__Clostridia.o__Clostrid
iales.f__Lachnospiraceae.g__Clostridium),2), nsmall=2),"%)", sep="")

h["Root.p__Firmicutes.c__Clostridia.o__Clostridiales.f__Lachnospirac
eae.g__Lachnobacterium"] = paste("g__Lachnobacterium (",
format(round(100*mean(y$Root.p__Firmicutes.c__Clostridia.o__Clostrid
iales.f__Lachnospiraceae.g__Lachnobacterium),2), nsmall=2),"%)",
sep="")

h["Root.p__Firmicutes.c__Clostridia.o__Clostridiales.f__Lachnospirac
eae.g__Lachnospira"] = paste("g__Lachnospira (",
format(round(100*mean(y$Root.p__Firmicutes.c__Clostridia.o__Clostrid
iales.f__Lachnospiraceae.g__Lachnospira),2), nsmall=2),"%)", sep="")

h["Root.p__Firmicutes.c__Clostridia.o__Clostridiales.f__Lachnospirac
eae.g__Pseudobutyrivibrio"] = paste("g__Pseudobutyrivibrio (",
format(round(100*mean(y$Root.p__Firmicutes.c__Clostridia.o__Clostrid
```

```
iales.f__Lachnospiraceae.g__Pseudobutyrivibrio),2), nsmall=2),"%)",
sep="")

h["Root.p__Firmicutes.c__Clostridia.o__Clostridiales.f__Lachnospirac
eae.g__Ruminococcus"] = paste("g__Ruminococcus (",
format(round(100*mean(y$Root.p__Firmicutes.c__Clostridia.o__Clostrid
iales.f__Lachnospiraceae.g__Ruminococcus),2), nsmall=2),"%)",
sep="")

h["Root.p__Firmicutes.c__Clostridia.o__Clostridiales.f__Lachnospirac
eae.g__Shuttleworthia"] = paste("g__Shuttleworthia (",
format(round(100*mean(y$Root.p__Firmicutes.c__Clostridia.o__Clostrid
iales.f__Lachnospiraceae.g__Shuttleworthia),2), nsmall=2),"%)",
sep="")

h["Root.p__Firmicutes.c__Clostridia.o__Clostridiales.f__Lachnospirac
eae.Other"] = paste("f__Lachnospiraceae (",
format(round(100*mean(y$Root.p__Firmicutes.c__Clostridia.o__Clostrid
iales.f__Lachnospiraceae.Other),2), nsmall=2),"%)", sep="")

h["Root.p__Firmicutes.c__Clostridia.o__Clostridiales.f__Ruminococcac
eae.g__"] = paste("f__Ruminococcaceae (",
format(round(100*mean(y$Root.p__Firmicutes.c__Clostridia.o__Clostrid
iales.f__Ruminococcaceae.g__),2), nsmall=2),"%)", sep="")

h["Root.p__Firmicutes.c__Clostridia.o__Clostridiales.f__Ruminococcac
eae.Other"] = paste("f__Ruminococcaceae (",
format(round(100*mean(y$Root.p__Firmicutes.c__Clostridia.o__Clostrid
iales.f__Ruminococcaceae.Other),2), nsmall=2),"%)", sep="")

h["Root.p__Firmicutes.c__Clostridia.o__Clostridiales.f__Ruminococcac
eae.g__Bacteroides"] = paste("g__Bacteroides (",
format(round(100*mean(y$Root.p__Firmicutes.c__Clostridia.o__Clostrid
iales.f__Ruminococcaceae.g__Bacteroides),2), nsmall=2),"%)", sep="")

h["Root.p__Firmicutes.c__Clostridia.o__Clostridiales.f__Ruminococcac
eae.g__Clostridium"] = paste("g__Clostridium (",
format(round(100*mean(y$Root.p__Firmicutes.c__Clostridia.o__Clostrid
iales.f__Ruminococcaceae.g__Clostridium),2), nsmall=2),"%)", sep="")

h["Root.p__Firmicutes.c__Clostridia.o__Clostridiales.f__Ruminococcac
eae.g__Eubacterium"] = paste("g__Eubacterium (",
format(round(100*mean(y$Root.p__Firmicutes.c__Clostridia.o__Clostrid
iales.f__Ruminococcaceae.g__Eubacterium),2), nsmall=2),"%)", sep="")

h["Root.p__Firmicutes.c__Clostridia.o__Clostridiales.f__Ruminococcac
eae.g__Faecalibacterium"] = paste("g__Faecalibacterium (",
format(round(100*mean(y$Root.p__Firmicutes.c__Clostridia.o__Clostrid
iales.f__Ruminococcaceae.g__Faecalibacterium),2), nsmall=2),"%)",
sep="")

h["Root.p__Firmicutes.c__Clostridia.o__Clostridiales.f__Ruminococcac
eae.g__Oscillospira"] = paste("g__Oscillospira (",
format(round(100*mean(y$Root.p__Firmicutes.c__Clostridia.o__Clostrid
```

```
iales.f__Ruminococcaceae.g__Oscillospira),2), nsmall=2),"%)",
sep="")

h["Root.p__Firmicutes.c__Clostridia.o__Clostridiales.f__Ruminococcac
eae.g__Ruminococcus"] = paste("g__Ruminococcus (",
format(round(100*mean(y$Root.p__Firmicutes.c__Clostridia.o__Clostrid
iales.f__Ruminococcaceae.g__Ruminococcus),2), nsmall=2),"%)",
sep="")

h["Root.p__Firmicutes.c__Clostridia.o__Clostridiales.f__Ruminococcac
eae.g__Subdoligranulum"] = paste("g__Subdoligranulum (",
format(round(100*mean(y$Root.p__Firmicutes.c__Clostridia.o__Clostrid
iales.f__Ruminococcaceae.g__Subdoligranulum),2), nsmall=2),"%)",
sep="")

h["Root.p__Firmicutes.c__Clostridia.o__Clostridiales.f__Veillonellac
eae.g__Dialister"] = paste("g__Dialister (",
format(round(100*mean(y$Root.p__Firmicutes.c__Clostridia.o__Clostrid
iales.f__Veillonellaceae.g__Dialister),2), nsmall=2),"%)", sep="")

h["Root.p__Firmicutes.c__Clostridia.o__Clostridiales.f__Veillonellac
eae.g__Mitsuokella"] = paste("g__Mitsuokella (",
format(round(100*mean(y$Root.p__Firmicutes.c__Clostridia.o__Clostrid
iales.f__Veillonellaceae.g__Mitsuokella),2), nsmall=2),"%)", sep="")

h["Root.p__Firmicutes.c__Clostridia.o__Clostridiales.f__Veillonellac
eae.g__Veillonella"] = paste("g__Veillonella (",
format(round(100*mean(y$Root.p__Firmicutes.c__Clostridia.o__Clostrid
iales.f__Veillonellaceae.g__Veillonella),2), nsmall=2),"%)", sep="")
  h["Root.p__Firmicutes.c__Clostridia.o__Clostridiales.Other.Other"]
= paste("o__Clostridiales (",
format(round(100*mean(y$Root.p__Firmicutes.c__Clostridia.o__Clostrid
iales.Other.Other),2), nsmall=2),"%)", sep="")
  h["Root.p__Firmicutes.c__Clostridia.Other.Other.Other"] =
paste("c__Clostridia (",
format(round(100*mean(y$Root.p__Firmicutes.c__Clostridia.Other.Other
.Other),2), nsmall=2),"%)", sep="")

h["Root.p__Fusobacteria.c__Fusobacteria.o__Fusobacteriales.f__Fusoba
cteriaceae.g__Fusobacterium"] = paste("g__Fusobacterium (",
format(round(100*mean(y$Root.p__Fusobacteria.c__Fusobacteria.o__Fuso
bacteriales.f__Fusobacteriaceae.g__Fusobacterium),2),
nsmall=2),"%)", sep="")

h["Root.p__Fusobacteria.c__Fusobacteria.o__Fusobacteriales.f__Fusoba
cteriaceae.g__Leptotrichia"] = paste("g__Leptotrichia (",
format(round(100*mean(y$Root.p__Fusobacteria.c__Fusobacteria.o__Fuso
bacteriales.f__Fusobacteriaceae.g__Leptotrichia),2), nsmall=2),"%)",
sep="")

h["Root.p__Fusobacteria.c__Fusobacteria.o__Fusobacteriales.f__Fusoba
cteriaceae.g__Sneathia"] = paste("g__Sneathia (",
format(round(100*mean(y$Root.p__Fusobacteria.c__Fusobacteria.o__Fuso
bacteriales.f__Fusobacteriaceae.g__Sneathia),2), nsmall=2),"%)",
```

```
sep="")

h["Root.p__Fusobacteria.c__Fusobacteria.o__Fusobacteriales.f__Fusobacteriaceae.Other"] = paste("f__Fusobacteriaceae (", format(round(100*mean(y$Root.p__Fusobacteria.c__Fusobacteria.o__Fusobacteriales.f__Fusobacteriaceae.Other),2), nsmall=2),"%)", sep="")

h["Root.p__Proteobacteria.c__Alphaproteobacteria.o__Caulobacterales.f__Caulobacteraceae.g__Caulobacter"] = paste("g__Caulobacter (", format(round(100*mean(y$Root.p__Proteobacteria.c__Alphaproteobacteria.o__Caulobacterales.f__Caulobacteraceae.g__Caulobacter),2), nsmall=2),"%)", sep="")

h["Root.p__Proteobacteria.c__Alphaproteobacteria.o__Caulobacterales.f__Caulobacteraceae.g__Phenylobacterium"] = paste("g__Phenylobacterium (", format(round(100*mean(y$Root.p__Proteobacteria.c__Alphaproteobacteria.o__Caulobacterales.f__Caulobacteraceae.g__Phenylobacterium),2), nsmall=2),"%)", sep="")

h["Root.p__Proteobacteria.c__Alphaproteobacteria.o__Rhizobiales.f__Bradyrhizobiaceae.g__Bradyrhizobium"] = paste("g__Bradyrhizobium (", format(round(100*mean(y$Root.p__Proteobacteria.c__Alphaproteobacteria.o__Rhizobiales.f__Bradyrhizobiaceae.g__Bradyrhizobium),2), nsmall=2),"%)", sep="")

h["Root.p__Proteobacteria.c__Alphaproteobacteria.o__Rhizobiales.f__Hyphomicrobiaceae.g__Devosia"] = paste("g__Devosia (", format(round(100*mean(y$Root.p__Proteobacteria.c__Alphaproteobacteria.o__Rhizobiales.f__Hyphomicrobiaceae.g__Devosia),2), nsmall=2),"%)", sep="")

h["Root.p__Proteobacteria.c__Alphaproteobacteria.o__Rhodobacterales.f__Rhodobacteraceae.g__Thioclava"] = paste("g__Thioclava (", format(round(100*mean(y$Root.p__Proteobacteria.c__Alphaproteobacteria.o__Rhodobacterales.f__Rhodobacteraceae.g__Thioclava),2), nsmall=2),"%)", sep="")

h["Root.p__Proteobacteria.c__Alphaproteobacteria.o__Sphingomonadales.f__Sphingomonadaceae.Other"] = paste("f__Sphingomonadaceae (", format(round(100*mean(y$Root.p__Proteobacteria.c__Alphaproteobacteria.o__Sphingomonadales.f__Sphingomonadaceae.Other),2), nsmall=2),"%)", sep="")

h["Root.p__Proteobacteria.c__Alphaproteobacteria.o__Sphingomonadales.Other.Other"] = paste("o__Sphingomonadales (", format(round(100*mean(y$Root.p__Proteobacteria.c__Alphaproteobacteria.o__Sphingomonadales.Other.Other),2), nsmall=2),"%)", sep="")

h["Root.p__Proteobacteria.c__Alphaproteobacteria.Other.Other.Other"] = paste("c__Alphaproteobacteria (", format(round(100*mean(y$Root.p__Proteobacteria.c__Alphaproteobacteria.Other.Other.Other),2), nsmall=2),"%)", sep="")
```

```
h["Root.p__Proteobacteria.c__Betaproteobacteria.o__Burkholderiales.f
__Alcaligenaceae.g__Oligella"] = paste("g__Oligella (",
format(round(100*mean(y$Root.p__Proteobacteria.c__Betaproteobacteria
.o__Burkholderiales.f__Alcaligenaceae.g__Oligella),2),
nsmall=2),"%)", sep="")

h["Root.p__Proteobacteria.c__Betaproteobacteria.o__Burkholderiales.f
__Burkholderiaceae.g__Burkholderia"] = paste("g__Burkholderia (",
format(round(100*mean(y$Root.p__Proteobacteria.c__Betaproteobacteria
.o__Burkholderiales.f__Burkholderiaceae.g__Burkholderia),2),
nsmall=2),"%)", sep="")

h["Root.p__Proteobacteria.c__Betaproteobacteria.o__Burkholderiales.f
__Burkholderiaceae.g__Ralstonia"] = paste("g__Ralstonia (",
format(round(100*mean(y$Root.p__Proteobacteria.c__Betaproteobacteria
.o__Burkholderiales.f__Burkholderiaceae.g__Ralstonia),2),
nsmall=2),"%)", sep="")

h["Root.p__Proteobacteria.c__Betaproteobacteria.o__Burkholderiales.f
__Comamonadaceae.g__Acidovorax"] = paste("g__Acidovorax (",
format(round(100*mean(y$Root.p__Proteobacteria.c__Betaproteobacteria
.o__Burkholderiales.f__Comamonadaceae.g__Acidovorax),2),
nsmall=2),"%)", sep="")

h["Root.p__Proteobacteria.c__Betaproteobacteria.o__Burkholderiales.f
__Oxalobacteraceae.g__Janthinobacterium"] =
paste("g__Janthinobacterium (",
format(round(100*mean(y$Root.p__Proteobacteria.c__Betaproteobacteria
.o__Burkholderiales.f__Oxalobacteraceae.g__Janthinobacterium),2),
nsmall=2),"%)", sep="")

h["Root.p__Proteobacteria.c__Betaproteobacteria.o__Burkholderiales.f
__Oxalobacteraceae.Other"] = paste("f__Oxalobacteraceae (",
format(round(100*mean(y$Root.p__Proteobacteria.c__Betaproteobacteria
.o__Burkholderiales.f__Oxalobacteraceae.Other),2), nsmall=2),"%)",
sep="")

h["Root.p__Proteobacteria.c__Betaproteobacteria.o__Neisseriales.f__N
eisseriaceae.g__"] = paste("f__Neisseriaceae (",
format(round(100*mean(y$Root.p__Proteobacteria.c__Betaproteobacteria
.o__Neisseriales.f__Neisseriaceae.g__),2), nsmall=2),"%)", sep="")

h["Root.p__Proteobacteria.c__Betaproteobacteria.o__Neisseriales.f__N
eisseriaceae.g__Neisseria"] = paste("g__Neisseria (",
format(round(100*mean(y$Root.p__Proteobacteria.c__Betaproteobacteria
.o__Neisseriales.f__Neisseriaceae.g__Neisseria),2), nsmall=2),"%)",
sep="")

h["Root.p__Proteobacteria.c__Betaproteobacteria.o__Neisseriales.f__N
eisseriaceae.g__Simonsiella"] = paste("g__Simonsiella (",
format(round(100*mean(y$Root.p__Proteobacteria.c__Betaproteobacteria
.o__Neisseriales.f__Neisseriaceae.g__Simonsiella),2),
nsmall=2),"%)", sep="")
```

```
h["Root.p__Proteobacteria.c__Epsilonproteobacteria.o__Campylobactera
les.f__Campylobacteraceae.g__Campylobacter"] =
paste("g__Campylobacter (",
format(round(100*mean(y$Root.p__Proteobacteria.c__Epsilonproteobacte
ria.o__Campylobacterales.f__Campylobacteraceae.g__Campylobacter),2),
nsmall=2),"%)", sep="")

h["Root.p__Proteobacteria.c__Gammaproteobacteria.o__Aeromonadales.f_
_Aeromonadaceae.g__Aeromonas"] = paste("g__Aeromonas (",
format(round(100*mean(y$Root.p__Proteobacteria.c__Gammaproteobacteri
a.o__Aeromonadales.f__Aeromonadaceae.g__Aeromonas),2),
nsmall=2),"%)", sep="")

h["Root.p__Proteobacteria.c__Gammaproteobacteria.o__Alteromonadales.
f__Pseudoalteromonadaceae.g__Pseudoalteromonas"] =
paste("g__Pseudoalteromonas (",
format(round(100*mean(y$Root.p__Proteobacteria.c__Gammaproteobacteri
a.o__Alteromonadales.f__Pseudoalteromonadaceae.g__Pseudoalteromonas)
,2), nsmall=2),"%)", sep="")

h["Root.p__Proteobacteria.c__Gammaproteobacteria.o__Enterobacteriale
s.f__Enterobacteriaceae.g__Averyella"] = paste("g__Averyella (",
format(round(100*mean(y$Root.p__Proteobacteria.c__Gammaproteobacteri
a.o__Enterobacteriales.f__Enterobacteriaceae.g__Averyella),2),
nsmall=2),"%)", sep="")

h["Root.p__Proteobacteria.c__Gammaproteobacteria.o__Enterobacteriale
s.f__Enterobacteriaceae.g__Citrobacter"] = paste("g__Citrobacter (",
format(round(100*mean(y$Root.p__Proteobacteria.c__Gammaproteobacteri
a.o__Enterobacteriales.f__Enterobacteriaceae.g__Citrobacter),2),
nsmall=2),"%)", sep="")

h["Root.p__Proteobacteria.c__Gammaproteobacteria.o__Enterobacteriale
s.f__Enterobacteriaceae.g__Escherichia"] = paste("g__Escherichia (",
format(round(100*mean(y$Root.p__Proteobacteria.c__Gammaproteobacteri
a.o__Enterobacteriales.f__Enterobacteriaceae.g__Escherichia),2),
nsmall=2),"%)", sep="")

h["Root.p__Proteobacteria.c__Gammaproteobacteria.o__Enterobacteriale
s.f__Enterobacteriaceae.g__Klebsiella"] = paste("g__Klebsiella (",
format(round(100*mean(y$Root.p__Proteobacteria.c__Gammaproteobacteri
a.o__Enterobacteriales.f__Enterobacteriaceae.g__Klebsiella),2),
nsmall=2),"%)", sep="")

h["Root.p__Proteobacteria.c__Gammaproteobacteria.o__Enterobacteriale
s.f__Enterobacteriaceae.g__Leclercia"] = paste("g__Leclercia (",
format(round(100*mean(y$Root.p__Proteobacteria.c__Gammaproteobacteri
a.o__Enterobacteriales.f__Enterobacteriaceae.g__Leclercia),2),
nsmall=2),"%)", sep="")

h["Root.p__Proteobacteria.c__Gammaproteobacteria.o__Enterobacteriale
s.f__Enterobacteriaceae.g__Proteus"] = paste("g__Proteus (",
format(round(100*mean(y$Root.p__Proteobacteria.c__Gammaproteobacteri
a.o__Enterobacteriales.f__Enterobacteriaceae.g__Proteus),2),
```

```
nsmall=2),"%)", sep="")

h["Root.p__Proteobacteria.c__Gammaproteobacteria.o__Enterobacteriale
s.f__Enterobacteriaceae.g__Serratia"] = paste("g__Serratia (",
format(round(100*mean(y$Root.p__Proteobacteria.c__Gammaproteobacteri
a.o__Enterobacteriales.f__Enterobacteriaceae.g__Serratia),2),
nsmall=2),"%)", sep="")

h["Root.p__Proteobacteria.c__Gammaproteobacteria.o__Enterobacteriale
s.f__Enterobacteriaceae.Other"] = paste("f__Enterobacteriaceae (",
format(round(100*mean(y$Root.p__Proteobacteria.c__Gammaproteobacteri
a.o__Enterobacteriales.f__Enterobacteriaceae.Other),2),
nsmall=2),"%)", sep="")

h["Root.p__Proteobacteria.c__Gammaproteobacteria.o__Oceanospirillale
s.f__Halomonadaceae.g__Halomonas"] = paste("g__Halomonas (",
format(round(100*mean(y$Root.p__Proteobacteria.c__Gammaproteobacteri
a.o__Oceanospirillales.f__Halomonadaceae.g__Halomonas),2),
nsmall=2),"%)", sep="")

h["Root.p__Proteobacteria.c__Gammaproteobacteria.o__Pasteurellales.f
__Pasteurellaceae.g__Actinobacillus"] = paste("g__Actinobacillus (",
format(round(100*mean(y$Root.p__Proteobacteria.c__Gammaproteobacteri
a.o__Pasteurellales.f__Pasteurellaceae.g__Actinobacillus),2),
nsmall=2),"%)", sep="")

h["Root.p__Proteobacteria.c__Gammaproteobacteria.o__Pasteurellales.f
__Pasteurellaceae.g__Haemophilus"] = paste("g__Haemophilus (",
format(round(100*mean(y$Root.p__Proteobacteria.c__Gammaproteobacteri
a.o__Pasteurellales.f__Pasteurellaceae.g__Haemophilus),2),
nsmall=2),"%)", sep="")

h["Root.p__Proteobacteria.c__Gammaproteobacteria.o__Pasteurellales.f
__Pasteurellaceae.g__Pasteurella"] = paste("g__Pasteurella (",
format(round(100*mean(y$Root.p__Proteobacteria.c__Gammaproteobacteri
a.o__Pasteurellales.f__Pasteurellaceae.g__Pasteurella),2),
nsmall=2),"%)", sep="")

h["Root.p__Proteobacteria.c__Gammaproteobacteria.o__Pseudomonadales.
f__Moraxellaceae.g__"] = paste("f__Moraxellaceae (",
format(round(100*mean(y$Root.p__Proteobacteria.c__Gammaproteobacteri
a.o__Pseudomonadales.f__Moraxellaceae.g__),2), nsmall=2),"%)",
sep="")

h["Root.p__Proteobacteria.c__Gammaproteobacteria.o__Pseudomonadales.
f__Moraxellaceae.g__Acinetobacter"] = paste("g__Acinetobacter (",
format(round(100*mean(y$RRoot.p__Proteobacteria.c__Gammaproteobacter
ia.o__Pseudomonadales.f__Moraxellaceae.g__Acinetobacter),2),
nsmall=2),"%)", sep="")

h["Root.p__Proteobacteria.c__Gammaproteobacteria.o__Pseudomonadales.
f__Moraxellaceae.g__Moraxella"] = paste("g__Moraxella (",
format(round(100*mean(y$Root.p__Proteobacteria.c__Gammaproteobacteri
a.o__Pseudomonadales.f__Moraxellaceae.g__Moraxella),2),
```

```
nsmall=2),"%)", sep="")

h["Root.p__Proteobacteria.c__Gammaproteobacteria.o__Pseudomonadales.
f__Pseudomonadaceae.g__Pseudomonas"] = paste("g__Pseudomonas (",
format(round(100*mean(y$Root.p__Proteobacteria.c__Gammaproteobacteri
a.o__Pseudomonadales.f__Pseudomonadaceae.g__Pseudomonas),2),
nsmall=2),"%)", sep="")

h["Root.p__Proteobacteria.c__Gammaproteobacteria.o__Pseudomonadales.
f__Pseudomonadaceae.Other"] = paste("f__Pseudomonadaceae (",
format(round(100*mean(y$Root.p__Proteobacteria.c__Gammaproteobacteri
a.o__Pseudomonadales.f__Pseudomonadaceae.Other),2), nsmall=2),"%)",
sep="")

h["Root.p__Proteobacteria.c__Gammaproteobacteria.o__Vibrionales.f__V
ibrionaceae.g__Photobacterium"] = paste("g__Photobacterium (",
format(round(100*mean(y$Root.p__Proteobacteria.c__Gammaproteobacteri
a.o__Vibrionales.f__Vibrionaceae.g__Photobacterium),2),
nsmall=2),"%)", sep="")

h["Root.p__Proteobacteria.c__Gammaproteobacteria.o__Vibrionales.f__V
ibrionaceae.g__Vibrio"] = paste("g__Vibrio (",
format(round(100*mean(y$Root.p__Proteobacteria.c__Gammaproteobacteri
a.o__Vibrionales.f__Vibrionaceae.g__Vibrio),2), nsmall=2),"%)",
sep="")

h["Root.p__Proteobacteria.c__Gammaproteobacteria.o__Vibrionales.f__V
ibrionaceae.Other"] = paste("f__Vibrionaceae (",
format(round(100*mean(y$Root.p__Proteobacteria.c__Gammaproteobacteri
a.o__Vibrionales.f__Vibrionaceae.Other),2), nsmall=2),"%)", sep="")

h["Root.p__Proteobacteria.c__Gammaproteobacteria.o__Xanthomonadales.
f__Xanthomonadaceae.g__Stenotrophomonas"] =
paste("g__Stenotrophomonas (",
format(round(100*mean(y$Root.p__Proteobacteria.c__Gammaproteobacteri
a.o__Xanthomonadales.f__Xanthomonadaceae.g__Stenotrophomonas),2),
nsmall=2),"%)", sep="")

h["Root.p__Tenericutes.c__Erysipelotrichi.o__Erysipelotrichales.f__E
rysipelotrichaceae.g__"] = paste("f__Erysipelotrichaceae (",
format(round(100*mean(y$Root.p__Tenericutes.c__Erysipelotrichi.o__Er
ysipelotrichales.f__Erysipelotrichaceae.g__),2), nsmall=2),"%)",
sep="")

h["Root.p__Tenericutes.c__Erysipelotrichi.o__Erysipelotrichales.f__E
rysipelotrichaceae.g__Catenibacterium"] = paste("g__Catenibacterium
(",
format(round(100*mean(y$Root.p__Tenericutes.c__Erysipelotrichi.o__Er
ysipelotrichales.f__Erysipelotrichaceae.g__Catenibacterium),2),
nsmall=2),"%)", sep="")

h["Root.p__Tenericutes.c__Erysipelotrichi.o__Erysipelotrichales.f__E
rysipelotrichaceae.g__Clostridium"] = paste("g__Clostridium (",
format(round(100*mean(y$Root.p__Tenericutes.c__Erysipelotrichi.o__Er
```

````
ysipelotrichales.f__Erysipelotrichaceae.g__Clostridium),2),
nsmall=2),"%)", sep="")

h["Root.p__Tenericutes.c__Erysipelotrichi.o__Erysipelotrichales.f__E
rysipelotrichaceae.g__Coprobacillus"] = paste("g__Coprobacillus (",
format(round(100*mean(y$Root.p__Tenericutes.c__Erysipelotrichi.o__Er
ysipelotrichales.f__Erysipelotrichaceae.g__Coprobacillus),2),
nsmall=2),"%)", sep="")

h["Root.p__Tenericutes.c__Mollicutes.o__Mycoplasmatales.f__Mycoplasm
ataceae.g__Mycoplasma"] = paste("g__Mycoplasma (",
format(round(100*mean(y$Root.p__Tenericutes.c__Mollicutes.o__Mycopla
smatales.f__Mycoplasmataceae.g__Mycoplasma),2), nsmall=2),"%)",
sep="")

h["Root.p__Thermi.c__Deinococci.o__Deinococcales.f__Deinococcaceae.g
__Deinococcus"] = paste("g__Deinococcus (",
format(round(100*mean(y$Root.p__Thermi.c__Deinococci.o__Deinococcale
s.f__Deinococcaceae.g__Deinococcus),2), nsmall=2),"%)", sep="")

h["Root.p__Verrucomicrobia.c__Verrucomicrobiae.o__Verrucomicrobiales
.f__Verrucomicrobiaceae.g__Akkermansia"] = paste("g__Akkermansia (",
format(round(100*mean(y$Root.p__Verrucomicrobia.c__Verrucomicrobiae.
o__Verrucomicrobiales.f__Verrucomicrobiaceae.g__Akkermansia),2),
nsmall=2),"%)", sep="")
  h["Root.Other.Other.Other.Other.Other"] = paste("Root (",
format(round(100*mean(y$Root.Other.Other.Other.Other.Other),2),
nsmall=2),"%)", sep="")
  h["Taxa < 1%"] = paste("Taxa < 1% (",
format(round(100*mean(y$"Taxa < 1%"),2), nsmall=2),"%)", sep="")
  h["other"] = paste("Other (", format(round(100*mean(y$other),2),
nsmall=2),"%)", sep="")
  #Make a vector of colours to return
  labels <- character(0)
  for (i in 1:length(x)){
      col <- h[[x[i]]]
      #print(col)
      if (is.null(col)) {
        print(x[i])
      }
      labels <- c(labels, col)
  }
  return(labels)
}
```

Setup: define colours & labels for L7 taxa (dada2_L7 function)
```{r colours, include=FALSE}
dada2_L7 <- function(x) {
  #Setup hash
  h <- hash()
  h["Corynebacterium_1"] = "#d15919" #009ce4"
  h["Staphylococcus"] = "#875073"
  h["Moraxella"] = "#005b9e"
````

```
h["Dolosigranulum"] = "#7eb5ff"
h["Streptococcus"] = "#c7006f"
h["Mycoplasma"] = "#7a7800"
h["Escherichia.Shigella"] = "#d5c870"
h["Cutibacterium"] = "#245d3a"
h["Pseudoalteromonas"] = "#025bb0"
h["Pseudomonas"] = "#ff9e73"
h["Bifidobacterium"] = "#c888a8"
h["Enterococcus"] = "#ff772b"
h["Bacteroides"] = "#007c45"
h["Haemophilus"] = "#f14735"
h["Lactobacillus"] = "#1c558d"
h["Vibrio"] = "#d95fe4"
h["Veillonella"] = "#6add7f"
h["Blautia"] = "#ff525b"
h["Fusobacterium"] = "#cd0498"
h["Neisseria"] = "#fb5cd7"
h["Anaerococcus"] = "#8d1a94"
h["Faecalibacterium"] = "#02abaf"
h["Salmonella"] = "#1ebc42"
h["Pasteurella"] = "#f2b1eb"
h["Tyzzerella_4"] = "#d96900"
h["Rothia"] = "#c30b9d"
h["Bacillus"] = "#7132a5"
h["Janthinobacterium"] = "#77da98"
h["Burkholderia.Caballeronia.Paraburkholderia"] = "#e2aaff"
h["Peptoniphilus"] = "#01d2e9"
h["Clostridium_sensu_stricto_1"] = "#f177fd"
h["Distigma"] = "#647441"
h["Prevotella_7"] = "#ff50ae"
h["Klebsiella"] = "#006dda"
h["Sphingopyxis"] = "#931a80"
h["Actinomyces"] = "#eebf71"
h["Pseudobutyrivibrio"] = "#6fcbff"
h["Lactococcus"] = "#52c4ff"
h["Azotobacter"] = "#fab5a1"
h["Finegoldia"] = "#8794ff"
h["Porphyromonas"] = "#c15100"
h["Eisenbergiella"] = "#0275ca"
h["Aeromonas"] = "#ff8a3d"
h["Dorea"] = "#8b4f41"
h["Lawsonella"] = "#665e2b"
h["Paenibacillus"] = "#0293ed"
h["Serratia"] = "#00830c"
h["Halomonas"] ="#c8cc5e"
h["Citrobacter"] = "#c56100"
h["Alistipes"] = "#006205"
h["Stenotrophomonas"] = "#ffaba0"
h["Lachnospiraceae_NK4A136_group"] = "#6167f0"
h["Acinetobacter"] = "#92331d"
h["Granulicatella"] = "#7d59e2"
h["Malassezia"] = "#b449cf"
h["Prevotella_9"] = "#007df5"
h["Raoultella"] = "#029efb"
```

```
h["Gemella"] = "#3a8166"
h["Roseburia"] = "#895f3b"
h["Romboutsia"] = "#d42eaf"
h["Cedecea"] = "#53d5fe"
h["Campylobacter"] = "#ff58a6"
h["Parabacteroides"] = "#bd3400"
h["Collinsella"] = "#91d957"
h["Fusicatenibacter"] = "#9a4900"
h["Anaerostipes"] = "#895500"
h["Prevotella"] = "#9f1e32"
h["Enterobacter"] = "#b3b300"
h["Ruminococcus_2"] = "#35c94e"
h["Ralstonia"] = "#a48d00"
h["Subdoligranulum"] = "#5d71fc"
h["Corynebacterium"] = "#90d5ad"
h["Proteus"] = "#a06100"
h["Lachnospira"] = "#b6007d"
h["Turicibacter"] = "#9e7cff"
h["Prevotellaceae_UCG.001"] = "#355c1d"
h["Ruminococcus_1"] = "#98d332"
h["Leptotrichia"] = "#ff63b9"
h["Catenibacterium"] = "#ff73ce"
h["Coprococcus_1"] = "#01b7ca"
h["Prevotella_2"] = "#784702"
h["Ruminococcaceae_UCG.002"] = "#966f47"
h["Ruminococcaceae_UCG.005"] = "#a10262"
h["Aerococcus"] = "#295ae0"
h["Akkermansia"] = "#e47a00"
h["Ruminococcaceae_UCG.014"] = "#007142"
h["Alloprevotella"] = "#ee289c"
h["Ezakiella"] = "#b4006b"
h["Ruminiclostridium_9"] = "#d88e9b"
h["Christensenellaceae_R.7_group"] = "#705900"
h["Methylobacterium"] = "#7ac7ad"
h["Candidatus_Stoquefichus"] = "#ff9ee3"
h["Flavonifractor"] = "#95a670"
h["Brevibacterium"] = "#f4bd68"
h["Caulobacter"] = "#9f1947"
h["Rhodocytophaga"] = "#b3000d"
h["Erysipelotrichaceae_UCG.003"] = "#ff4f46"
h["Shewanella"] = "#80308b"
h["Capnocytophaga"] = "#a99fff"
h["Clostridium_sensu_stricto_7"] = "#00816d"
h["Comamonas"] = "#995561"
h["Pluralibacter"] = "#b459e1"
h["Facklamia"] = "#008126"
h["Parvimonas"] = "#8b22ab"
h["Hungatella"] = "#bcb4ff"
h["Anoxybacillus"] = "#f66327"
h["Delftia"] = "#b50022"
h["Coprococcus_2"] = "#646e00"
h["Sellimonas"] = "#ea8b00"
h["Simonsiella"] = "#ee234b"
h["Ruminococcaceae_UCG.003"] = "#a36a52"
```

```
h["Chryseobacterium"] = "#ff6abf"
h["Holdemanella"] = "#8dd78d"
h["Ruminococcaceae_UCG.013"] = "#e26700"
h["Atopobium"] = "#80d7b6"
h["Lachnoclostridium_5"] = "#cf2d17"
h["Ruminiclostridium"] = "#942c47"
h["Eggerthella"] = "#abd532"
h["Intestinimonas"] = "#1cdc6c"
h["Actinobaculum"] = "#53cb46"
h["Acetatifactor"] = "#b90060"
h["Ensifer"] = "#f5bd5a"
h["Zea"] = "#d43dbe"
h["Acidovorax"] = "#ff57c3"
h["Kingella"] = "#8a3744"
h["Mitsuokella"] = "#863e11"
h["Peptostreptococcus"] = "#ff985b"
h["Odoribacter"] = "#ff99c6"
h["Actinobacillus"] = "#5dd9d3"
h["Lachnospiraceae_UCG.001"] = "#dd3c20"
h["Lachnoanaerobaculum"] = "#0273da"
h["Abiotrophia"] = "#ff5950"
h["Phenylobacterium"] = "#fb298b"
h["Erysipelatoclostridium"] = "#a0016a"
h["Negativicoccus"] = "#01d3b6"
h["Lachnospiraceae_UCG.006"] = "#bdaa00"
h["Phormidesmis_ANT.LACV5.1"] = "#2d69f2"
h["A2"] = "#006996"
h["Deinococcus"] = "#b07d00"
h["Pseudoglutamicibacter"] = "#b0008b"
h["Cloacibacterium"] = "#0080aa"
h["Selenomonas_3"] = "#f8b1da"
h["Actinotignum"] = "#792ba7"
h["Clostridium_sensu_stricto_12"] = "#84da78"
h["Ruminococcaceae_UCG.004"] = "#3791ff"
h["Sphingomonas"] = "#eb85ff"
h["Ileibacterium"] = "#4ae088"
h["Muribaculum"] = "#a50096"
h["Aeribacillus"] = "#dd007f"
h["Oceanobacillus"] = "#0285ea"
h["Tepidicella"] = "#ffa126"
h["Oligella"] = "#91d867"
h["Ornithinibacillus"] = "#e1c556"
h["Filifactor"] = "#4c5902"
h["Photobacterium"] = "#ff9076"
h["Tyzzerella_3"] = "#82404b"
h["Micrococcus"] = "#932f39"
h["Catonella"] = "#3ce187"
h["Brevundimonas"] = "#b77600"
h["Porphyrobacter"] = "#f00a6d"
h["Trueperella"] = "#0c601c"
h["Tibeticola"] = "#4dd253"
h["Weissella"] = "#4a591d"
h["possible_genus_Sk018"] = "#ff4b72"
h["Oscillibacter"] = "#a525ae"
```

```
  h["Taxa < 1%"] = "#808080"
  h["other"] = "#808080"
  h["NA"] = "#181818"
  #Make a vector of colours to return
  colours <- character(0)
  for (i in 1:length(x)){
    if (x[i] == "Taxa < 1%") {
      col <- h[[x[i]]]
    } else {
      x.sub  <- str_split(x[i], "_L6_")[[1]][2]
      #x.sub <- str_split(x.su, "_L5_")[[1]][1]
      col <- h[[x.sub]]
    }
      #print(col)
      if (is.null(col)) {
        print(x[i])
      }
      colours <- c(colours, col)
  }
  return(colours)
}

#myLabels_L6 function
myLabels_L6 <- function(x,y) {
  #Setup hash
  h <- hash()

h["Root.p__Actinobacteria.c__Actinobacteria.o__Actinomycetales.f__Ac
tinomycetaceae.g__Actinobaculum"] = paste("g__Actinobaculum (",
format(round(100*mean(y$Root.p__Actinobacteria.c__Actinobacteria.o__
Actinomycetales.f__Actinomycetaceae.g__Actinobaculum),2),
nsmall=2),"%)", sep="")

h["Root.p__Actinobacteria.c__Actinobacteria.o__Actinomycetales.f__Ac
tinomycetaceae.g__Actinomyces"] = paste("g__Actinomyces (",
format(round(100*mean(y$Root.p__Actinobacteria.c__Actinobacteria.o__
Actinomycetales.f__Actinomycetaceae.g__Actinomyces),2),
nsmall=2),"%)", sep="")

h["Root.p__Actinobacteria.c__Actinobacteria.o__Coriobacteriales.f__C
oriobacteriaceae.g__Adlercreutzia"] = paste("g__Adlercreutzia (",
format(round(100*mean(y$Root.p__Actinobacteria.c__Actinobacteria.o__
Actinomycetales.f__Actinomycetaceae.g__Adlercreutzia),2),
nsmall=2),"%)", sep="")

h["Root.p__Actinobacteria.c__Actinobacteria.o__Actinomycetales.f__Ac
tinomycetaceae.g__Arcanobacterium"] = paste("g__Arcanobacterium (",
format(round(100*mean(y$Root.p__Actinobacteria.c__Actinobacteria.o__
Actinomycetales.f__Actinomycetaceae.g__Arcanobacterium),2),
nsmall=2),"%)", sep="")

h["Root.p__Actinobacteria.c__Actinobacteria.o__Actinomycetales.f__Br
evibacteriaceae.g__Brevibacterium"] = paste("g__Brevibacterium (",
format(round(100*mean(y$Root.p__Actinobacteria.c__Actinobacteria.o__
```

```
Actinomycetales.f__Brevibacteriaceae.g__Brevibacterium),2),
nsmall=2),"%)", sep="")

h["Root.p__Actinobacteria.c__Actinobacteria.o__Actinomycetales.f__Co
rynebacteriaceae.g__"] = paste("f__Corynebacteriaceae (",
format(round(100*mean(y$Root.p__Actinobacteria.c__Actinobacteria.o__
Actinomycetales.f__Corynebacteriaceae.g__),2), nsmall=2),"%)",
sep="")

h["Root.p__Actinobacteria.c__Actinobacteria.o__Actinomycetales.f__Co
rynebacteriaceae.g__Corynebacterium"] = paste("g__Corynebacterium
(",
format(round(100*mean(y$Root.p__Actinobacteria.c__Actinobacteria.o__
Actinomycetales.f__Corynebacteriaceae.g__Corynebacterium),2),
nsmall=2),"%)", sep="")

h["Root.p__Actinobacteria.c__Actinobacteria.o__Actinomycetales.f__In
trasporangiaceae.g__"] = paste("f__Intrasporangiaceae (",
format(round(100*mean(y$Root.p__Actinobacteria.c__Actinobacteria.o__
Actinomycetales.f__Intrasporangiaceae.g__),2), nsmall=2),"%)",
sep="")

h["Root.p__Actinobacteria.c__Actinobacteria.o__Actinomycetales.f__Mi
crococcaceae.g__Rothia"] = paste("g__Rothia (",
format(round(100*mean(y$Root.p__Actinobacteria.c__Actinobacteria.o__
Actinomycetales.f__Micrococcaceae.g__Rothia),2), nsmall=2),"%)",
sep="")

h["Root.p__Actinobacteria.c__Actinobacteria.o__Actinomycetales.f__Mi
cromonosporaceae.Other"] = paste("f__Micromonosporaceae (",
format(round(100*mean(y$Root.p__Actinobacteria.c__Actinobacteria.o__
Actinomycetales.f__Micromonosporaceae.Other),2), nsmall=2),"%)",
sep="")

h["Root.p__Actinobacteria.c__Actinobacteria.o__Actinomycetales.f__Pr
omicromonosporaceae.g__Cellulosimicrobium"] =
paste("g__Cellulosimicrobium (",
format(round(100*mean(y$Root.p__Actinobacteria.c__Actinobacteria.o__
Actinomycetales.f__Promicromonosporaceae.g__Cellulosimicrobium),2),
nsmall=2),"%)", sep="")

h["Root.p__Actinobacteria.c__Actinobacteria.o__Actinomycetales.f__Pr
opionibacteriaceae.g__Propionibacterium"] =
paste("g__Propionibacterium (",
format(round(100*mean(y$Root.p__Actinobacteria.c__Actinobacteria.o__
Actinomycetales.f__Propionibacteriaceae.g__Propionibacterium),2),
nsmall=2),"%)", sep="")

h["Root.p__Actinobacteria.c__Actinobacteria.o__Actinomycetales.f__Pr
opionibacteriaceae.Other"] = paste("f__Propionibacteriaceae (",
format(round(100*mean(y$Root.p__Actinobacteria.c__Actinobacteria.o__
Actinomycetales.f__Propionibacteriaceae.Other),2), nsmall=2),"%)",
sep="")
```

```
h["Root.p__Actinobacteria.c__Actinobacteria.o__Actinomycetales.Other
.Other"] = paste("o__Actinomycetales (",
format(round(100*mean(y$Root.p__Actinobacteria.c__Actinobacteria.o__
Actinomycetales.Other.Other),2), nsmall=2),"%)", sep="")

h["Root.p__Actinobacteria.c__Actinobacteria.o__Bifidobacteriales.f__
Bifidobacteriaceae.g__Bifidobacterium"] = paste("g__Bifidobacterium
(",
format(round(100*mean(y$Root.p__Actinobacteria.c__Actinobacteria.o__
Bifidobacteriales.f__Bifidobacteriaceae.g__Bifidobacterium),2),
nsmall=2),"%)", sep="")

h["Root.p__Actinobacteria.c__Actinobacteria.o__Bifidobacteriales.f__
Bifidobacteriaceae.g__Gardnerella"] = paste("g__Gardnerella (",
format(round(100*mean(y$Root.p__Actinobacteria.c__Actinobacteria.o__
Bifidobacteriales.f__Bifidobacteriaceae.g__Gardnerella),2),
nsmall=2),"%)", sep="")

h["Root.p__Actinobacteria.c__Actinobacteria.o__Bifidobacteriales.f__
Bifidobacteriaceae.g__Scardovia"] = paste("g__Scardovia (",
format(round(100*mean(y$Root.p__Actinobacteria.c__Actinobacteria.o__
Bifidobacteriales.f__Bifidobacteriaceae.g__Scardovia),2),
nsmall=2),"%)", sep="")

h["Root.p__Actinobacteria.c__Actinobacteria.o__Coriobacteriales.f__C
oriobacteriaceae.g__Collinsella"] = paste("g__Collinsella (",
format(round(100*mean(y$Root.p__Actinobacteria.c__Actinobacteria.o__
Coriobacteriales.f__Coriobacteriaceae.g__Collinsella),2),
nsmall=2),"%)", sep="")
  h["Root.p__Bacteroidetes.c__Bacteroidia.o__Bacteroidales.f__.g__"]
= paste("o__Bacteroidales (",
format(round(100*mean(y$Root.p__Bacteroidetes.c__Bacteroidia.o__Bact
eroidales.f__.g__),2), nsmall=2),"%)", sep="")

h["Root.p__Bacteroidetes.c__Bacteroidia.o__Bacteroidales.f__Bacteroi
daceae.g__Bacteroides"] = paste("g__Bacteroides (",
format(round(100*mean(y$Root.p__Bacteroidetes.c__Bacteroidia.o__Bact
eroidales.f__Bacteroidaceae.g__Bacteroides),2), nsmall=2),"%)",
sep="")

h["Root.p__Bacteroidetes.c__Bacteroidia.o__Bacteroidales.f__Porphyro
monadaceae.g__"] = paste("f__Porphyromonadaceae (",
format(round(100*mean(y$Root.p__Bacteroidetes.c__Bacteroidia.o__Bact
eroidales.f__Porphyromonadaceae.g__),2), nsmall=2),"%)", sep="")

h["Root.p__Bacteroidetes.c__Bacteroidia.o__Bacteroidales.f__Porphyro
monadaceae.g__Parabacteroides"] = paste("g__Parabacteroides (",
format(round(100*mean(y$Root.p__Bacteroidetes.c__Bacteroidia.o__Bact
eroidales.f__Porphyromonadaceae.g__Parabacteroides),2),
nsmall=2),"%)", sep="")

h["Root.p__Bacteroidetes.c__Bacteroidia.o__Bacteroidales.f__Porphyro
monadaceae.g__Porphyromonas"] = paste("g__Porphyromonas (",
format(round(100*mean(y$Root.p__Bacteroidetes.c__Bacteroidia.o__Bact
```

```
eroidales.f__Porphyromonadaceae.g__Porphyromonas),2),
nsmall=2),"%)", sep="")

h["Root.p__Bacteroidetes.c__Bacteroidia.o__Bacteroidales.f__Prevotel
laceae.g__Prevotella"] = paste("g__Prevotella (",
format(round(100*mean(y$Root.p__Bacteroidetes.c__Bacteroidia.o__Bact
eroidales.f__Prevotellaceae.g__Prevotella),2), nsmall=2),"%)",
sep="")

h["Root.p__Bacteroidetes.c__Bacteroidia.o__Bacteroidales.f__Rikenell
aceae.g__Alistipes"] = paste("g__Alistipes (",
format(round(100*mean(y$Root.p__Bacteroidetes.c__Bacteroidia.o__Bact
eroidales.f__Rikenellaceae.g__Alistipes),2), nsmall=2),"%)", sep="")

h["Root.p__Bacteroidetes.c__Flavobacteria.o__Flavobacteriales.f__Fla
vobacteriaceae.g__Capnocytophaga"] = paste("g__Capnocytophaga (",
format(round(100*mean(y$Root.p__Bacteroidetes.c__Flavobacteria.o__Fl
avobacteriales.f__Flavobacteriaceae.g__Capnocytophaga),2),
nsmall=2),"%)", sep="")

h["Root.p__Bacteroidetes.c__Flavobacteria.o__Flavobacteriales.f__Fla
vobacteriaceae.g__Chryseobacterium"] = paste("g__Chryseobacterium
(",
format(round(100*mean(y$Root.p__Bacteroidetes.c__Flavobacteria.o__Fl
avobacteriales.f__Flavobacteriaceae.g__Chryseobacterium),2),
nsmall=2),"%)", sep="")

h["Root.p__Bacteroidetes.c__Sphingobacteria.o__Sphingobacteriales.f_
_Flexibacteraceae.g__"] = paste("f__Flexibacteraceae (",
format(round(100*mean(y$Root.p__Bacteroidetes.c__Sphingobacteria.o__
Sphingobacteriales.f__Flexibacteraceae.g__),2), nsmall=2),"%)",
sep="")

h["Root.p__Bacteroidetes.c__Sphingobacteria.o__Sphingobacteriales.f_
_Sphingobacteriaceae.g__Sphingobacterium"] =
paste("g__Sphingobacterium (",
format(round(100*mean(y$Root.p__Bacteroidetes.c__Sphingobacteria.o__
Sphingobacteriales.f__Sphingobacteriaceae.g__Sphingobacterium),2),
nsmall=2),"%)", sep="")
  h["Root.p__Chloroflexi.c__SOGA31.o__.f__.g__"] = paste("c__SOGA31
(",
format(round(100*mean(y$Root.p__Chloroflexi.c__SOGA31.o__.f__.g__),2
), nsmall=2),"%)", sep="")
  h["Root.p__Crenarchaeota.Other.Other.Other.Other"] =
paste("p__Crenarchaeota (",
format(round(100*mean(y$Root.p__Crenarchaeota.Other.Other.Other.Othe
r),2), nsmall=2),"%)", sep="")
  h["Root.p__Cyanobacteria.c__Chloroplast.o__Streptophyta.f__.g__"]
= paste("o__Streptophyta (",
format(round(100*mean(y$Root.p__Cyanobacteria.c__Chloroplast.o__Stre
ptophyta.f__.g__),2), nsmall=2),"%)", sep="")

h["Root.p__Cyanobacteria.c__Nostocophycideae.o__Nostocales.f__Nostoc
aceae.Other"] = paste("f__Nostocaceae (",
```

```
format(round(100*mean(y$Root.p__Cyanobacteria.c__Nostocophycideae.o_
_Nostocales.f__Nostocaceae.Other),2), nsmall=2),"%)", sep="")

h["Root.p__Cyanobacteria.c__Synechococcophycideae.o__Pseudanabaenale
s.f__Pseudanabaenaceae.g__Halomicronema"] = paste("g__Halomicronema
(",
format(round(100*mean(y$Root.p__Cyanobacteria.c__Synechococcophycide
ae.o__Pseudanabaenales.f__Pseudanabaenaceae.g__Halomicronema),2),
nsmall=2),"%)", sep="")

h["Root.p__Firmicutes.c__Bacilli.o__Bacillales.f__Bacillaceae.g__Ano
xybacillus"] = paste("g__Anoxybacillus (",
format(round(100*mean(y$Root.p__Firmicutes.c__Bacilli.o__Bacillales.
f__Bacillaceae.g__Anoxybacillus),2), nsmall=2),"%)", sep="")

h["Root.p__Firmicutes.c__Bacilli.o__Bacillales.f__Bacillaceae.g__Bac
illus"] = paste("g__Bacillus (",
format(round(100*mean(y$Root.p__Firmicutes.c__Bacilli.o__Bacillales.
f__Bacillaceae.g__Bacillus),2), nsmall=2),"%)", sep="")

h["Root.p__Firmicutes.c__Bacilli.o__Bacillales.f__Paenibacillaceae.g
__Paenibacillus"] = paste("g__Paenibacillus (",
format(round(100*mean(y$Root.p__Firmicutes.c__Bacilli.o__Bacillales.
f__Paenibacillaceae.g__Paenibacillus),2), nsmall=2),"%)", sep="")

h["Root.p__Firmicutes.c__Bacilli.o__Bacillales.f__Staphylococcaceae.
g__Staphylococcus"] = paste("g__Staphylococcus (",
format(round(100*mean(y$Root.p__Firmicutes.c__Bacilli.o__Bacillales.
f__Staphylococcaceae.g__Staphylococcus),2), nsmall=2),"%)", sep="")

h["Root.p__Firmicutes.c__Bacilli.o__Gemellales.f__Gemellaceae.g__Gem
ella"] = paste("g__Gemella (",
format(round(100*mean(y$Root.p__Firmicutes.c__Bacilli.o__Gemellales.
f__Gemellaceae.g__Gemella),2), nsmall=2),"%)", sep="")

h["Root.p__Firmicutes.c__Bacilli.o__Lactobacillales.f__Aerococcaceae
.g__Aerococcus"] = paste("g__Aerococcus (",
format(round(100*mean(y$Root.p__Firmicutes.c__Bacilli.o__Lactobacill
ales.f__Aerococcaceae.g__Aerococcus),2), nsmall=2),"%)", sep="")

h["Root.p__Firmicutes.c__Bacilli.o__Lactobacillales.f__Aerococcaceae
.g__Facklamia"] = paste("g__Facklamia (",
format(round(100*mean(y$Root.p__Firmicutes.c__Bacilli.o__Lactobacill
ales.f__Aerococcaceae.g__Facklamia),2), nsmall=2),"%)", sep="")

h["Root.p__Firmicutes.c__Bacilli.o__Lactobacillales.f__Carnobacteria
ceae.g__"] = paste("f__Carnobacteriaceae (",
format(round(100*mean(y$Root.p__Firmicutes.c__Bacilli.o__Lactobacill
ales.f__Carnobacteriaceae.g__),2), nsmall=2),"%)", sep="")

h["Root.p__Firmicutes.c__Bacilli.o__Lactobacillales.f__Carnobacteria
ceae.g__Granulicatella"] = paste("g__Granulicatella (",
format(round(100*mean(y$Root.p__Firmicutes.c__Bacilli.o__Lactobacill
ales.f__Carnobacteriaceae.g__Granulicatella),2), nsmall=2),"%)",
```

```
sep="")

h["Root.p__Firmicutes.c__Bacilli.o__Lactobacillales.f__Enterococcace
ae.g__Enterococcus"] = paste("g__Enterococcus (",
format(round(100*mean(y$Root.p__Firmicutes.c__Bacilli.o__Lactobacill
ales.f__Enterococcaceae.g__Enterococcus),2), nsmall=2),"%)", sep="")

h["Root.p__Firmicutes.c__Bacilli.o__Lactobacillales.f__Lactobacillac
eae.g__Lactobacillus"] = paste("g__Lactobacillus (",
format(round(100*mean(y$Root.p__Firmicutes.c__Bacilli.o__Lactobacill
ales.f__Lactobacillaceae.g__Lactobacillus),2), nsmall=2),"%)",
sep="")

h["Root.p__Firmicutes.c__Bacilli.o__Lactobacillales.f__Leuconostocac
eae.g__Weissella"] = paste("g__Weissella (",
format(round(100*mean(y$Root.p__Firmicutes.c__Bacilli.o__Lactobacill
ales.f__Leuconostocaceae.g__Weissella),2), nsmall=2),"%)", sep="")

h["Root.p__Firmicutes.c__Bacilli.o__Lactobacillales.f__Streptococcac
eae.g__Lactococcus"] = paste("g__Lactococcus (",
format(round(100*mean(y$Root.p__Firmicutes.c__Bacilli.o__Lactobacill
ales.f__Streptococcaceae.g__Lactococcus),2), nsmall=2),"%)", sep="")

h["Root.p__Firmicutes.c__Bacilli.o__Lactobacillales.f__Streptococcac
eae.g__Streptococcus"] = paste("g__Streptococcus (",
format(round(100*mean(y$Root.p__Firmicutes.c__Bacilli.o__Lactobacill
ales.f__Streptococcaceae.g__Streptococcus),2), nsmall=2),"%)",
sep="")

h["Root.p__Firmicutes.c__Bacilli.o__Lactobacillales.f__Streptococcac
eae.Other"] = paste("f__Streptococcaceae (",
format(round(100*mean(y$Root.p__Firmicutes.c__Bacilli.o__Lactobacill
ales.f__Streptococcaceae.Other),2), nsmall=2),"%)", sep="")

h["Root.p__Firmicutes.c__Bacilli.o__Turicibacterales.f__Turicibacter
aceae.g__"] = paste("f__Turicibacteraceae (",
format(round(100*mean(y$Root.p__Firmicutes.c__Bacilli.o__Turicibacte
rales.f__Turicibacteraceae.g__),2), nsmall=2),"%)", sep="")

h["Root.p__Firmicutes.c__Bacilli.o__Turicibacterales.f__Turicibacter
aceae.g__Turicibacter"] = paste("g__Turicibacter (",
format(round(100*mean(y$Root.p__Firmicutes.c__Bacilli.o__Turicibacte
rales.f__Turicibacteraceae.g__Turicibacter),2), nsmall=2),"%)",
sep="")
  h["Root.p__Firmicutes.c__Clostridia.o__Clostridiales.f__.g__"] =
paste("o__Clostridiales (",
format(round(100*mean(y$Root.p__Firmicutes.c__Clostridia.o__Clostrid
iales.f__.g__),2), nsmall=2),"%)", sep="")

h["Root.p__Firmicutes.c__Clostridia.o__Clostridiales.f__Catabacteria
ceae.g__"] = paste("f__Catabacteriaceae (",
format(round(100*mean(y$Root.p__Firmicutes.c__Clostridia.o__Clostrid
iales.f__Catabacteriaceae.g__),2), nsmall=2),"%)", sep="")
```

```
h["Root.p__Firmicutes.c__Clostridia.o__Clostridiales.f__Clostridiace
ae.g__Clostridium"] = paste("g__Clostridium (",
format(round(100*mean(y$Root.p__Firmicutes.c__Clostridia.o__Clostrid
iales.f__Clostridiaceae.g__Clostridium),2), nsmall=2),"%)", sep="")

h["Root.p__Firmicutes.c__Clostridia.o__Clostridiales.f__Clostridiace
ae.Other"] = paste("f__Clostridiaceae (",
format(round(100*mean(y$Root.p__Firmicutes.c__Clostridia.o__Clostrid
iales.f__Clostridiaceae.Other),2), nsmall=2),"%)", sep="")

h["Root.p__Firmicutes.c__Clostridia.o__Clostridiales.f__Clostridiale
sFamilyXI.IncertaeSedis.g__"] =
paste("f__ClostridialesFamilyXI.IncertaeSedis (",
format(round(100*mean(y$Root.p__Firmicutes.c__Clostridia.o__Clostrid
iales.f__ClostridialesFamilyXI.IncertaeSedis.g__),2),
nsmall=2),"%)", sep="")

h["Root.p__Firmicutes.c__Clostridia.o__Clostridiales.f__Clostridiale
sFamilyXI.IncertaeSedis.g__Anaerococcus"] = paste("g__Anaerococcus
(",
format(round(100*mean(y$Root.p__Firmicutes.c__Clostridia.o__Clostrid
iales.f__ClostridialesFamilyXI.IncertaeSedis.g__Anaerococcus),2),
nsmall=2),"%)", sep="")

h["Root.p__Firmicutes.c__Clostridia.o__Clostridiales.f__Clostridiale
sFamilyXI.IncertaeSedis.g__Finegoldia"] = paste("g__Finegoldia (",
format(round(100*mean(y$Root.p__Firmicutes.c__Clostridia.o__Clostrid
iales.f__ClostridialesFamilyXI.IncertaeSedis.g__Finegoldia),2),
nsmall=2),"%)", sep="")

h["Root.p__Firmicutes.c__Clostridia.o__Clostridiales.f__Clostridiale
sFamilyXI.IncertaeSedis.g__Peptoniphilus"] = paste("g__Peptoniphilus
(",
format(round(100*mean(y$Root.p__Firmicutes.c__Clostridia.o__Clostrid
iales.f__ClostridialesFamilyXI.IncertaeSedis.g__Peptoniphilus),2),
nsmall=2),"%)", sep="")

h["Root.p__Firmicutes.c__Clostridia.o__Clostridiales.f__Lachnospirac
eae.g__"] = paste("f__Lachnospiraceae (",
format(round(100*mean(y$Root.p__Firmicutes.c__Clostridia.o__Clostrid
iales.f__Lachnospiraceae.g__),2), nsmall=2),"%)", sep="")

h["Root.p__Firmicutes.c__Clostridia.o__Clostridiales.f__Lachnospirac
eae.g__Blautia"] = paste("g__Blautia (",
format(round(100*mean(y$Root.p__Firmicutes.c__Clostridia.o__Clostrid
iales.f__Lachnospiraceae.g__Blautia),2), nsmall=2),"%)", sep="")

h["Root.p__Firmicutes.c__Clostridia.o__Clostridiales.f__Lachnospirac
eae.g__Catonella"] = paste("g__Catonella (",
format(round(100*mean(y$Root.p__Firmicutes.c__Clostridia.o__Clostrid
iales.f__Lachnospiraceae.g__Catonella),2), nsmall=2),"%)", sep="")

h["Root.p__Firmicutes.c__Clostridia.o__Clostridiales.f__Lachnospirac
eae.g__Clostridium"] = paste("g__Clostridium (",
```

```
format(round(100*mean(y$Root.p__Firmicutes.c__Clostridia.o__Clostrid
iales.f__Lachnospiraceae.g__Clostridium),2), nsmall=2),"%)", sep="")

h["Root.p__Firmicutes.c__Clostridia.o__Clostridiales.f__Lachnospirac
eae.g__Lachnobacterium"] = paste("g__Lachnobacterium (",
format(round(100*mean(y$Root.p__Firmicutes.c__Clostridia.o__Clostrid
iales.f__Lachnospiraceae.g__Lachnobacterium),2), nsmall=2),"%)",
sep="")

h["Root.p__Firmicutes.c__Clostridia.o__Clostridiales.f__Lachnospirac
eae.g__Lachnospira"] = paste("g__Lachnospira (",
format(round(100*mean(y$Root.p__Firmicutes.c__Clostridia.o__Clostrid
iales.f__Lachnospiraceae.g__Lachnospira),2), nsmall=2),"%)", sep="")

h["Root.p__Firmicutes.c__Clostridia.o__Clostridiales.f__Lachnospirac
eae.g__Pseudobutyrivibrio"] = paste("g__Pseudobutyrivibrio (",
format(round(100*mean(y$Root.p__Firmicutes.c__Clostridia.o__Clostrid
iales.f__Lachnospiraceae.g__Pseudobutyrivibrio),2), nsmall=2),"%)",
sep="")

h["Root.p__Firmicutes.c__Clostridia.o__Clostridiales.f__Lachnospirac
eae.g__Ruminococcus"] = paste("g__Ruminococcus (",
format(round(100*mean(y$Root.p__Firmicutes.c__Clostridia.o__Clostrid
iales.f__Lachnospiraceae.g__Ruminococcus),2), nsmall=2),"%)",
sep="")

h["Root.p__Firmicutes.c__Clostridia.o__Clostridiales.f__Lachnospirac
eae.g__Shuttleworthia"] = paste("g__Shuttleworthia (",
format(round(100*mean(y$Root.p__Firmicutes.c__Clostridia.o__Clostrid
iales.f__Lachnospiraceae.g__Shuttleworthia),2), nsmall=2),"%)",
sep="")

h["Root.p__Firmicutes.c__Clostridia.o__Clostridiales.f__Lachnospirac
eae.Other"] = paste("f__Lachnospiraceae (",
format(round(100*mean(y$Root.p__Firmicutes.c__Clostridia.o__Clostrid
iales.f__Lachnospiraceae.Other),2), nsmall=2),"%)", sep="")

h["Root.p__Firmicutes.c__Clostridia.o__Clostridiales.f__Ruminococcac
eae.g__"] = paste("f__Ruminococcaceae (",
format(round(100*mean(y$Root.p__Firmicutes.c__Clostridia.o__Clostrid
iales.f__Ruminococcaceae.g__),2), nsmall=2),"%)", sep="")

h["Root.p__Firmicutes.c__Clostridia.o__Clostridiales.f__Ruminococcac
eae.Other"] = paste("f__Ruminococcaceae (",
format(round(100*mean(y$Root.p__Firmicutes.c__Clostridia.o__Clostrid
iales.f__Ruminococcaceae.Other),2), nsmall=2),"%)", sep="")

h["Root.p__Firmicutes.c__Clostridia.o__Clostridiales.f__Ruminococcac
eae.g__Bacteroides"] = paste("g__Bacteroides (",
format(round(100*mean(y$Root.p__Firmicutes.c__Clostridia.o__Clostrid
iales.f__Ruminococcaceae.g__Bacteroides),2), nsmall=2),"%)", sep="")

h["Root.p__Firmicutes.c__Clostridia.o__Clostridiales.f__Ruminococcac
eae.g__Clostridium"] = paste("g__Clostridium (",
```

```
format(round(100*mean(y$Root.p__Firmicutes.c__Clostridia.o__Clostrid
iales.f__Ruminococcaceae.g__Clostridium),2), nsmall=2),"%)", sep="")

h["Root.p__Firmicutes.c__Clostridia.o__Clostridiales.f__Ruminococcac
eae.g__Eubacterium"] = paste("g__Eubacterium (",
format(round(100*mean(y$Root.p__Firmicutes.c__Clostridia.o__Clostrid
iales.f__Ruminococcaceae.g__Eubacterium),2), nsmall=2),"%)", sep="")

h["Root.p__Firmicutes.c__Clostridia.o__Clostridiales.f__Ruminococcac
eae.g__Faecalibacterium"] = paste("g__Faecalibacterium (",
format(round(100*mean(y$Root.p__Firmicutes.c__Clostridia.o__Clostrid
iales.f__Ruminococcaceae.g__Faecalibacterium),2), nsmall=2),"%)",
sep="")

h["Root.p__Firmicutes.c__Clostridia.o__Clostridiales.f__Ruminococcac
eae.g__Oscillospira"] = paste("g__Oscillospira (",
format(round(100*mean(y$Root.p__Firmicutes.c__Clostridia.o__Clostrid
iales.f__Ruminococcaceae.g__Oscillospira),2), nsmall=2),"%)",
sep="")

h["Root.p__Firmicutes.c__Clostridia.o__Clostridiales.f__Ruminococcac
eae.g__Ruminococcus"] = paste("g__Ruminococcus (",
format(round(100*mean(y$Root.p__Firmicutes.c__Clostridia.o__Clostrid
iales.f__Ruminococcaceae.g__Ruminococcus),2), nsmall=2),"%)",
sep="")

h["Root.p__Firmicutes.c__Clostridia.o__Clostridiales.f__Ruminococcac
eae.g__Subdoligranulum"] = paste("g__Subdoligranulum (",
format(round(100*mean(y$Root.p__Firmicutes.c__Clostridia.o__Clostrid
iales.f__Ruminococcaceae.g__Subdoligranulum),2), nsmall=2),"%)",
sep="")

h["Root.p__Firmicutes.c__Clostridia.o__Clostridiales.f__Veillonellac
eae.g__Dialister"] = paste("g__Dialister (",
format(round(100*mean(y$Root.p__Firmicutes.c__Clostridia.o__Clostrid
iales.f__Veillonellaceae.g__Dialister),2), nsmall=2),"%)", sep="")

h["Root.p__Firmicutes.c__Clostridia.o__Clostridiales.f__Veillonellac
eae.g__Mitsuokella"] = paste("g__Mitsuokella (",
format(round(100*mean(y$Root.p__Firmicutes.c__Clostridia.o__Clostrid
iales.f__Veillonellaceae.g__Mitsuokella),2), nsmall=2),"%)", sep="")

h["Root.p__Firmicutes.c__Clostridia.o__Clostridiales.f__Veillonellac
eae.g__Veillonella"] = paste("g__Veillonella (",
format(round(100*mean(y$Root.p__Firmicutes.c__Clostridia.o__Clostrid
iales.f__Veillonellaceae.g__Veillonella),2), nsmall=2),"%)", sep="")
  h["Root.p__Firmicutes.c__Clostridia.o__Clostridiales.Other.Other"]
= paste("o__Clostridiales (",
format(round(100*mean(y$Root.p__Firmicutes.c__Clostridia.o__Clostrid
iales.Other.Other),2), nsmall=2),"%)", sep="")
  h["Root.p__Firmicutes.c__Clostridia.Other.Other.Other"] =
paste("c__Clostridia (",
format(round(100*mean(y$Root.p__Firmicutes.c__Clostridia.Other.Other
.Other),2), nsmall=2),"%)", sep="")
```

```
h["Root.p__Fusobacteria.c__Fusobacteria.o__Fusobacteriales.f__Fusoba
cteriaceae.g__Fusobacterium"] = paste("g__Fusobacterium (",
format(round(100*mean(y$Root.p__Fusobacteria.c__Fusobacteria.o__Fuso
bacteriales.f__Fusobacteriaceae.g__Fusobacterium),2),
nsmall=2),"%)", sep="")

h["Root.p__Fusobacteria.c__Fusobacteria.o__Fusobacteriales.f__Fusoba
cteriaceae.g__Leptotrichia"] = paste("g__Leptotrichia (",
format(round(100*mean(y$Root.p__Fusobacteria.c__Fusobacteria.o__Fuso
bacteriales.f__Fusobacteriaceae.g__Leptotrichia),2), nsmall=2),"%)",
sep="")

h["Root.p__Fusobacteria.c__Fusobacteria.o__Fusobacteriales.f__Fusoba
cteriaceae.g__Sneathia"] = paste("g__Sneathia (",
format(round(100*mean(y$Root.p__Fusobacteria.c__Fusobacteria.o__Fuso
bacteriales.f__Fusobacteriaceae.g__Sneathia),2), nsmall=2),"%)",
sep="")

h["Root.p__Fusobacteria.c__Fusobacteria.o__Fusobacteriales.f__Fusoba
cteriaceae.Other"] = paste("f__Fusobacteriaceae (",
format(round(100*mean(y$Root.p__Fusobacteria.c__Fusobacteria.o__Fuso
bacteriales.f__Fusobacteriaceae.Other),2), nsmall=2),"%)", sep="")

h["Root.p__Proteobacteria.c__Alphaproteobacteria.o__Caulobacterales.
f__Caulobacteraceae.g__Caulobacter"] = paste("g__Caulobacter (",
format(round(100*mean(y$Root.p__Proteobacteria.c__Alphaproteobacteri
a.o__Caulobacterales.f__Caulobacteraceae.g__Caulobacter),2),
nsmall=2),"%)", sep="")

h["Root.p__Proteobacteria.c__Alphaproteobacteria.o__Caulobacterales.
f__Caulobacteraceae.g__Phenylobacterium"] =
paste("g__Phenylobacterium (",
format(round(100*mean(y$Root.p__Proteobacteria.c__Alphaproteobacteri
a.o__Caulobacterales.f__Caulobacteraceae.g__Phenylobacterium),2),
nsmall=2),"%)", sep="")

h["Root.p__Proteobacteria.c__Alphaproteobacteria.o__Rhizobiales.f__B
radyrhizobiaceae.g__Bradyrhizobium"] = paste("g__Bradyrhizobium (",
format(round(100*mean(y$Root.p__Proteobacteria.c__Alphaproteobacteri
a.o__Rhizobiales.f__Bradyrhizobiaceae.g__Bradyrhizobium),2),
nsmall=2),"%)", sep="")

h["Root.p__Proteobacteria.c__Alphaproteobacteria.o__Rhizobiales.f__H
yphomicrobiaceae.g__Devosia"] = paste("g__Devosia (",
format(round(100*mean(y$Root.p__Proteobacteria.c__Alphaproteobacteri
a.o__Rhizobiales.f__Hyphomicrobiaceae.g__Devosia),2),
nsmall=2),"%)", sep="")

h["Root.p__Proteobacteria.c__Alphaproteobacteria.o__Rhodobacterales.
f__Rhodobacteraceae.g__Thioclava"] = paste("g__Thioclava (",
format(round(100*mean(y$Root.p__Proteobacteria.c__Alphaproteobacteri
a.o__Rhodobacterales.f__Rhodobacteraceae.g__Thioclava),2),
nsmall=2),"%)", sep="")
```

```
h["Root.p__Proteobacteria.c__Alphaproteobacteria.o__Sphingomonadales
.f__Sphingomonadaceae.Other"] = paste("f__Sphingomonadaceae (",
format(round(100*mean(y$Root.p__Proteobacteria.c__Alphaproteobacteri
a.o__Sphingomonadales.f__Sphingomonadaceae.Other),2),
nsmall=2),"%)", sep="")

h["Root.p__Proteobacteria.c__Alphaproteobacteria.o__Sphingomonadales
.Other.Other"] = paste("o__Sphingomonadales (",
format(round(100*mean(y$Root.p__Proteobacteria.c__Alphaproteobacteri
a.o__Sphingomonadales.Other.Other),2), nsmall=2),"%)", sep="")

h["Root.p__Proteobacteria.c__Alphaproteobacteria.Other.Other.Other"]
= paste("c__Alphaproteobacteria (",
format(round(100*mean(y$Root.p__Proteobacteria.c__Alphaproteobacteri
a.Other.Other.Other),2), nsmall=2),"%)", sep="")

h["Root.p__Proteobacteria.c__Betaproteobacteria.o__Burkholderiales.f
__Alcaligenaceae.g__Oligella"] = paste("g__Oligella (",
format(round(100*mean(y$Root.p__Proteobacteria.c__Betaproteobacteria
.o__Burkholderiales.f__Alcaligenaceae.g__Oligella),2),
nsmall=2),"%)", sep="")

h["Root.p__Proteobacteria.c__Betaproteobacteria.o__Burkholderiales.f
__Burkholderiaceae.g__Burkholderia"] = paste("g__Burkholderia (",
format(round(100*mean(y$Root.p__Proteobacteria.c__Betaproteobacteria
.o__Burkholderiales.f__Burkholderiaceae.g__Burkholderia),2),
nsmall=2),"%)", sep="")

h["Root.p__Proteobacteria.c__Betaproteobacteria.o__Burkholderiales.f
__Burkholderiaceae.g__Ralstonia"] = paste("g__Ralstonia (",
format(round(100*mean(y$Root.p__Proteobacteria.c__Betaproteobacteria
.o__Burkholderiales.f__Burkholderiaceae.g__Ralstonia),2),
nsmall=2),"%)", sep="")

h["Root.p__Proteobacteria.c__Betaproteobacteria.o__Burkholderiales.f
__Comamonadaceae.g__Acidovorax"] = paste("g__Acidovorax (",
format(round(100*mean(y$Root.p__Proteobacteria.c__Betaproteobacteria
.o__Burkholderiales.f__Comamonadaceae.g__Acidovorax),2),
nsmall=2),"%)", sep="")

h["Root.p__Proteobacteria.c__Betaproteobacteria.o__Burkholderiales.f
__Oxalobacteraceae.g__Janthinobacterium"] =
paste("g__Janthinobacterium (",
format(round(100*mean(y$Root.p__Proteobacteria.c__Betaproteobacteria
.o__Burkholderiales.f__Oxalobacteraceae.g__Janthinobacterium),2),
nsmall=2),"%)", sep="")

h["Root.p__Proteobacteria.c__Betaproteobacteria.o__Burkholderiales.f
__Oxalobacteraceae.Other"] = paste("f__Oxalobacteraceae (",
format(round(100*mean(y$Root.p__Proteobacteria.c__Betaproteobacteria
.o__Burkholderiales.f__Oxalobacteraceae.Other),2), nsmall=2),"%)",
sep="")
```

```
h["Root.p__Proteobacteria.c__Betaproteobacteria.o__Neisseriales.f__N
eisseriaceae.g__"] = paste("f__Neisseriaceae (",
format(round(100*mean(y$Root.p__Proteobacteria.c__Betaproteobacteria
.o__Neisseriales.f__Neisseriaceae.g__),2), nsmall=2),"%)", sep="")

h["Root.p__Proteobacteria.c__Betaproteobacteria.o__Neisseriales.f__N
eisseriaceae.g__Neisseria"] = paste("g__Neisseria (",
format(round(100*mean(y$Root.p__Proteobacteria.c__Betaproteobacteria
.o__Neisseriales.f__Neisseriaceae.g__Neisseria),2), nsmall=2),"%)",
sep="")

h["Root.p__Proteobacteria.c__Betaproteobacteria.o__Neisseriales.f__N
eisseriaceae.g__Simonsiella"] = paste("g__Simonsiella (",
format(round(100*mean(y$Root.p__Proteobacteria.c__Betaproteobacteria
.o__Neisseriales.f__Neisseriaceae.g__Simonsiella),2),
nsmall=2),"%)", sep="")

h["Root.p__Proteobacteria.c__Epsilonproteobacteria.o__Campylobactera
les.f__Campylobacteraceae.g__Campylobacter"] =
paste("g__Campylobacter (",
format(round(100*mean(y$Root.p__Proteobacteria.c__Epsilonproteobacte
ria.o__Campylobacterales.f__Campylobacteraceae.g__Campylobacter),2),
nsmall=2),"%)", sep="")

h["Root.p__Proteobacteria.c__Gammaproteobacteria.o__Aeromonadales.f_
_Aeromonadaceae.g__Aeromonas"] = paste("g__Aeromonas (",
format(round(100*mean(y$Root.p__Proteobacteria.c__Gammaproteobacteri
a.o__Aeromonadales.f__Aeromonadaceae.g__Aeromonas),2),
nsmall=2),"%)", sep="")

h["Root.p__Proteobacteria.c__Gammaproteobacteria.o__Alteromonadales.
f__Pseudoalteromonadaceae.g__Pseudoalteromonas"] =
paste("g__Pseudoalteromonas (",
format(round(100*mean(y$Root.p__Proteobacteria.c__Gammaproteobacteri
a.o__Alteromonadales.f__Pseudoalteromonadaceae.g__Pseudoalteromonas)
,2), nsmall=2),"%)", sep="")

h["Root.p__Proteobacteria.c__Gammaproteobacteria.o__Enterobacteriale
s.f__Enterobacteriaceae.g__Averyella"] = paste("g__Averyella (",
format(round(100*mean(y$Root.p__Proteobacteria.c__Gammaproteobacteri
a.o__Enterobacteriales.f__Enterobacteriaceae.g__Averyella),2),
nsmall=2),"%)", sep="")

h["Root.p__Proteobacteria.c__Gammaproteobacteria.o__Enterobacteriale
s.f__Enterobacteriaceae.g__Citrobacter"] = paste("g__Citrobacter (",
format(round(100*mean(y$Root.p__Proteobacteria.c__Gammaproteobacteri
a.o__Enterobacteriales.f__Enterobacteriaceae.g__Citrobacter),2),
nsmall=2),"%)", sep="")

h["Root.p__Proteobacteria.c__Gammaproteobacteria.o__Enterobacteriale
s.f__Enterobacteriaceae.g__Escherichia"] = paste("g__Escherichia (",
format(round(100*mean(y$Root.p__Proteobacteria.c__Gammaproteobacteri
a.o__Enterobacteriales.f__Enterobacteriaceae.g__Escherichia),2),
nsmall=2),"%)", sep="")
```

```
h["Root.p__Proteobacteria.c__Gammaproteobacteria.o__Enterobacteriale
s.f__Enterobacteriaceae.g__Klebsiella"] = paste("g__Klebsiella (",
format(round(100*mean(y$Root.p__Proteobacteria.c__Gammaproteobacteri
a.o__Enterobacteriales.f__Enterobacteriaceae.g__Klebsiella),2),
nsmall=2),"%)", sep="")

h["Root.p__Proteobacteria.c__Gammaproteobacteria.o__Enterobacteriale
s.f__Enterobacteriaceae.g__Leclercia"] = paste("g__Leclercia (",
format(round(100*mean(y$Root.p__Proteobacteria.c__Gammaproteobacteri
a.o__Enterobacteriales.f__Enterobacteriaceae.g__Leclercia),2),
nsmall=2),"%)", sep="")

h["Root.p__Proteobacteria.c__Gammaproteobacteria.o__Enterobacteriale
s.f__Enterobacteriaceae.g__Proteus"] = paste("g__Proteus (",
format(round(100*mean(y$Root.p__Proteobacteria.c__Gammaproteobacteri
a.o__Enterobacteriales.f__Enterobacteriaceae.g__Proteus),2),
nsmall=2),"%)", sep="")

h["Root.p__Proteobacteria.c__Gammaproteobacteria.o__Enterobacteriale
s.f__Enterobacteriaceae.g__Serratia"] = paste("g__Serratia (",
format(round(100*mean(y$Root.p__Proteobacteria.c__Gammaproteobacteri
a.o__Enterobacteriales.f__Enterobacteriaceae.g__Serratia),2),
nsmall=2),"%)", sep="")

h["Root.p__Proteobacteria.c__Gammaproteobacteria.o__Enterobacteriale
s.f__Enterobacteriaceae.Other"] = paste("f__Enterobacteriaceae (",
format(round(100*mean(y$Root.p__Proteobacteria.c__Gammaproteobacteri
a.o__Enterobacteriales.f__Enterobacteriaceae.Other),2),
nsmall=2),"%)", sep="")

h["Root.p__Proteobacteria.c__Gammaproteobacteria.o__Oceanospirillale
s.f__Halomonadaceae.g__Halomonas"] = paste("g__Halomonas (",
format(round(100*mean(y$Root.p__Proteobacteria.c__Gammaproteobacteri
a.o__Oceanospirillales.f__Halomonadaceae.g__Halomonas),2),
nsmall=2),"%)", sep="")

h["Root.p__Proteobacteria.c__Gammaproteobacteria.o__Pasteurellales.f
__Pasteurellaceae.g__Actinobacillus"] = paste("g__Actinobacillus (",
format(round(100*mean(y$Root.p__Proteobacteria.c__Gammaproteobacteri
a.o__Pasteurellales.f__Pasteurellaceae.g__Actinobacillus),2),
nsmall=2),"%)", sep="")

h["Root.p__Proteobacteria.c__Gammaproteobacteria.o__Pasteurellales.f
__Pasteurellaceae.g__Haemophilus"] = paste("g__Haemophilus (",
format(round(100*mean(y$Root.p__Proteobacteria.c__Gammaproteobacteri
a.o__Pasteurellales.f__Pasteurellaceae.g__Haemophilus),2),
nsmall=2),"%)", sep="")

h["Root.p__Proteobacteria.c__Gammaproteobacteria.o__Pasteurellales.f
__Pasteurellaceae.g__Pasteurella"] = paste("g__Pasteurella (",
format(round(100*mean(y$Root.p__Proteobacteria.c__Gammaproteobacteri
a.o__Pasteurellales.f__Pasteurellaceae.g__Pasteurella),2),
nsmall=2),"%)", sep="")
```

```
h["Root.p__Proteobacteria.c__Gammaproteobacteria.o__Pseudomonadales.
f__Moraxellaceae.g__"] = paste("f__Moraxellaceae (",
format(round(100*mean(y$Root.p__Proteobacteria.c__Gammaproteobacteri
a.o__Pseudomonadales.f__Moraxellaceae.g__),2), nsmall=2),"%)",
sep="")

h["Root.p__Proteobacteria.c__Gammaproteobacteria.o__Pseudomonadales.
f__Moraxellaceae.g__Acinetobacter"] = paste("g__Acinetobacter (",
format(round(100*mean(y$RRoot.p__Proteobacteria.c__Gammaproteobacter
ia.o__Pseudomonadales.f__Moraxellaceae.g__Acinetobacter),2),
nsmall=2),"%)", sep="")

h["Root.p__Proteobacteria.c__Gammaproteobacteria.o__Pseudomonadales.
f__Moraxellaceae.g__Moraxella"] = paste("g__Moraxella (",
format(round(100*mean(y$Root.p__Proteobacteria.c__Gammaproteobacteri
a.o__Pseudomonadales.f__Moraxellaceae.g__Moraxella),2),
nsmall=2),"%)", sep="")

h["Root.p__Proteobacteria.c__Gammaproteobacteria.o__Pseudomonadales.
f__Pseudomonadaceae.g__Pseudomonas"] = paste("g__Pseudomonas (",
format(round(100*mean(y$Root.p__Proteobacteria.c__Gammaproteobacteri
a.o__Pseudomonadales.f__Pseudomonadaceae.g__Pseudomonas),2),
nsmall=2),"%)", sep="")

h["Root.p__Proteobacteria.c__Gammaproteobacteria.o__Pseudomonadales.
f__Pseudomonadaceae.Other"] = paste("f__Pseudomonadaceae (",
format(round(100*mean(y$Root.p__Proteobacteria.c__Gammaproteobacteri
a.o__Pseudomonadales.f__Pseudomonadaceae.Other),2), nsmall=2),"%)",
sep="")

h["Root.p__Proteobacteria.c__Gammaproteobacteria.o__Vibrionales.f__V
ibrionaceae.g__Photobacterium"] = paste("g__Photobacterium (",
format(round(100*mean(y$Root.p__Proteobacteria.c__Gammaproteobacteri
a.o__Vibrionales.f__Vibrionaceae.g__Photobacterium),2),
nsmall=2),"%)", sep="")

h["Root.p__Proteobacteria.c__Gammaproteobacteria.o__Vibrionales.f__V
ibrionaceae.g__Vibrio"] = paste("g__Vibrio (",
format(round(100*mean(y$Root.p__Proteobacteria.c__Gammaproteobacteri
a.o__Vibrionales.f__Vibrionaceae.g__Vibrio),2), nsmall=2),"%)",
sep="")

h["Root.p__Proteobacteria.c__Gammaproteobacteria.o__Vibrionales.f__V
ibrionaceae.Other"] = paste("f__Vibrionaceae (",
format(round(100*mean(y$Root.p__Proteobacteria.c__Gammaproteobacteri
a.o__Vibrionales.f__Vibrionaceae.Other),2), nsmall=2),"%)", sep="")

h["Root.p__Proteobacteria.c__Gammaproteobacteria.o__Xanthomonadales.
f__Xanthomonadaceae.g__Stenotrophomonas"] =
paste("g__Stenotrophomonas (",
format(round(100*mean(y$Root.p__Proteobacteria.c__Gammaproteobacteri
a.o__Xanthomonadales.f__Xanthomonadaceae.g__Stenotrophomonas),2),
nsmall=2),"%)", sep="")
```

```
h["Root.p__Tenericutes.c__Erysipelotrichi.o__Erysipelotrichales.f__E
rysipelotrichaceae.g__"] = paste("f__Erysipelotrichaceae (",
format(round(100*mean(y$Root.p__Tenericutes.c__Erysipelotrichi.o__Er
ysipelotrichales.f__Erysipelotrichaceae.g__),2), nsmall=2),"%)",
sep="")

h["Root.p__Tenericutes.c__Erysipelotrichi.o__Erysipelotrichales.f__E
rysipelotrichaceae.g__Catenibacterium"] = paste("g__Catenibacterium
(",
format(round(100*mean(y$Root.p__Tenericutes.c__Erysipelotrichi.o__Er
ysipelotrichales.f__Erysipelotrichaceae.g__Catenibacterium),2),
nsmall=2),"%)", sep="")

h["Root.p__Tenericutes.c__Erysipelotrichi.o__Erysipelotrichales.f__E
rysipelotrichaceae.g__Clostridium"] = paste("g__Clostridium (",
format(round(100*mean(y$Root.p__Tenericutes.c__Erysipelotrichi.o__Er
ysipelotrichales.f__Erysipelotrichaceae.g__Clostridium),2),
nsmall=2),"%)", sep="")

h["Root.p__Tenericutes.c__Erysipelotrichi.o__Erysipelotrichales.f__E
rysipelotrichaceae.g__Coprobacillus"] = paste("g__Coprobacillus (",
format(round(100*mean(y$Root.p__Tenericutes.c__Erysipelotrichi.o__Er
ysipelotrichales.f__Erysipelotrichaceae.g__Coprobacillus),2),
nsmall=2),"%)", sep="")

h["Root.p__Tenericutes.c__Mollicutes.o__Mycoplasmatales.f__Mycoplasm
ataceae.g__Mycoplasma"] = paste("g__Mycoplasma (",
format(round(100*mean(y$Root.p__Tenericutes.c__Mollicutes.o__Mycopla
smatales.f__Mycoplasmataceae.g__Mycoplasma),2), nsmall=2),"%)",
sep="")

h["Root.p__Thermi.c__Deinococci.o__Deinococcales.f__Deinococcaceae.g
__Deinococcus"] = paste("g__Deinococcus (",
format(round(100*mean(y$Root.p__Thermi.c__Deinococci.o__Deinococcale
s.f__Deinococcaceae.g__Deinococcus),2), nsmall=2),"%)", sep="")

h["Root.p__Verrucomicrobia.c__Verrucomicrobiae.o__Verrucomicrobiales
.f__Verrucomicrobiaceae.g__Akkermansia"] = paste("g__Akkermansia (",
format(round(100*mean(y$Root.p__Verrucomicrobia.c__Verrucomicrobiae.
o__Verrucomicrobiales.f__Verrucomicrobiaceae.g__Akkermansia),2),
nsmall=2),"%)", sep="")
  h["Root.Other.Other.Other.Other.Other"] = paste("Root (",
format(round(100*mean(y$Root.Other.Other.Other.Other.Other),2),
nsmall=2),"%)", sep="")
  h["Taxa < 1%"] = paste("Taxa < 1% (",
format(round(100*mean(y$"Taxa < 1%"),2), nsmall=2),"%)", sep="")
  h["other"] = paste("Other (", format(round(100*mean(y$other),2),
nsmall=2),"%)", sep="")
  #Make a vector of colours to return
  labels <- character(0)
  for (i in 1:length(x)){
      col <- h[[x[i]]]
      #print(col)
```

````
      if (is.null(col)) {
        print(x[i])
      }
      labels <- c(labels, col)
  }
  return(labels)
}
```

Setup: define colours & labels for L7 Corynebacterium taxa
(dada2_L7_Coryne function)
```{r colours, include=FALSE}
dada2_L7_Coryne <- function(x) {
  #Setup hash
  h <- hash()
  #h["Corynebacterium_1"] = "#d15919" #009ce4"

h["CACGTAGTTAGCCGGTGCTTCTTATACAGGTACCGTCACAAAAAGCTTCGTCCCTGTCGAAAGGA
GTTTACAACCCGAAGGCCGTCATCCCCCACGCGGCGTCGCTGCATCAGGCTTCCGCCCATTGTGCAAT
ATTCCCCA_L6_Corynebacterium"] = "#c76b5f"

h["CACGTAGTTAGCCGGTGCTTCTTATCCAGGTACCGTCAAACAAACTTCGTCCCTGACGAAAGGAG
TTTACAACCCGAAGGCCTTCATCCCCCACACGGCGTCGCTGCATCAGGCTTCCGCCCATTGTGCAAAA
TTCCCCA_L6_Corynebacterium"] = "#b43227"

h["CACGTAGTTAGCCGGTGCTTCTTATACAGGTACCGTCACAAAAGCTTCGTCCCTGTCGAAAGGAG
TTTACAACCCGAAGGCCTTCATCCCCCACGCGGCGTCGCTGCATCAGGCTTCCGCCCATTGTGCAATA
TTCCCCA_L6_Corynebacterium"] = "#e25a3c"

h["CACGTAGTTAGCCGGTGCTTCTTATACAGGTACCGTCACCAAAGCTTCGTCCCTGTCGAAAGAGG
TTTACAACCCGAAGGCCGTCATCCCCCACGCGGCGTCGCTGCATCAGGCTTGCGCCCATTGTGCAATA
TTCCCCA_L6_Corynebacterium"] = "#d08e15"

h["CACGTAGTTAGCCGGTGCTTCTTCTCCAGGTACCGTCACAAAACGCTTCGTCCCTGGCGAAAGGA
GTTTACAACCCGAAGGCCGTCATCCCCCACGCGGCGTCGCTGCATCAGGCTTGCGCCCATTGTGCAAT
ATTCCCCA_L6_Corynebacterium_1"] = "#9e5f00"

h["CACGTAGTTAGCCGGTGCTTCTTATCTAGGTACCGTCACAAAAAGCTTCGTCCCTAGCGAAAGGA
GTTTACAACCCGAAGGCCTTCATCCCCCACGCGGCGTCGCTGCATCAGGCTTGCGCCCATTGTGCAAT
ATTCCCCA_L6_Corynebacterium_1"] = "#ff9971"

h["CACGTAGTTAGCCGGTGCTTCTTATCTAGGTACCGTCACAAAAAAGCTTCGTCCCTAGCGAAAGG
AGTTTACAACCCGAAGGCCTTCATCCCCCACGCGGCGTCGCTGCATCAGGCTTGCGCCCATTGTGCAA
TATTCCCCA_L6_Corynebacterium_1"] = "#915950"

h["CACGTAGTTAGCCGGTGCTTCTTATCTAGGTACCGTCACACAAAGCTTCGTCCCTAGCGAAAGGA
GTTTACAACCCGAAGGCCTTCATCCCCCACGCGGCGTCGCTGCATCAGGCTTGCGCCCATTGTGCAAT
ATTCCCCA_L6_Corynebacterium_1"] = "#bd7300"

h["CACGTAGTTAGCCGGTGCTTCTTATCTAGGTACCGTCACAAAAAGCTTCGTCCCTAGCGAAAGGA
GTTTACAACCCGAAGGCCGTCATCCCCCACGCGGCGTCGCTGCATCAGGCTTGCGCCCATTGTGCAAT
ATTCCCCA_L6_Corynebacterium_1"] = "#917220"

h["CACGTAGTTAGCCGGTGCTTCTTATCTAGGTACCGTCACAACAAAGCTTCGTCCCTAGCGAAAGG
````

AGTTTACAACCCGAAGGCCTTCATCCCCCACGCGGCGTCGCTGCATCAGGCTTGCGCCCATTGTGCAA
TATTCCCCA_L6_Corynebacterium_1"] = "#ffc870"

h["CACGTAGTTAGCCGGTGCTTCTTCTCCAGGTACCGTCACAAAACGCTTCGTCCCTGGCGAAAGGA
GTTTACAACCCGAAGGTCGTCATCCCCCACGCGGCGTCGCTGCATCAGGCTTGCGCCCATTGTGCAAT
ATTCCCCA_L6_Corynebacterium_1"] = "#e2b53d"

h["CACGTAGTTAGCCGGTGCTTCTTCTACCACTACCGTCACCACAAGGGCTTCGTCATGGCTGAAAG
GAGTTTACAACCCGAAGGCCGTCATCCCCCACGCGGCGTCGCTGCATCAGGCTTGCGCCCATTGTGCA
ATATTCCCCA_L6_Corynebacterium_1"] = "#e2b53d"

h["CACGTAGTTAGCCGGTGCTTCTTCTCCATCTACCGTCAGAAACCCTTCGTCGATGGTGAAAGGAG
TTTACAACCCGAAGGCCGTCATCCCCCACGCGGCGTCGCTGCATCAGGCTTCCGCCCATTGTGCAATA
TTCCCCA_L6_Corynebacterium_1"] = "#805b2b"

h["CACGTAGTTAGCCGGTGCTTCTTATACAGGTACCGTCACTTACGCTTCGTCCCTGTCGAAAGGAG
TTTACAACCCGAAGGCCTTCATCCCCCACGCGGCGTCGCTGCATCAGGCTTCCGCCCATTGTGCAATA
TTCCCCA_L6_Corynebacterium_1"] = "#ecd4aa"

h["CACGTAGTTAGCCGGTGCTTCTTATCTAGGTACCGTCACAAAAGCTTCGTCCCTAGCGAAAGGAG
TTTACAACCCGAAGGCCGTCATCCCCCACGCGGCGTCGCTGCATCAGGCTTGCGCCCATTGTGCAATA
TTCCCCA_L6_Corynebacterium_1"] = "#c29088"

h["CACGTAGTTAGCCGGTGCTTCTTATCTAGGTACCGTCACCAAAAAGCTTCGTCCCTAGCGAAAGG
AGTTTACAACCCGAAGGCCTTCATCCCCCACGCGGCGTCGCTGCATCAGGCTTGCGCCCATTGTGCAA
TATTCCCCA_L6_Corynebacterium_1"] = "#ad4e44"

h["CACGTAGTTAGCCGGTGCTTCTTATCTAGGTACCGTCACAAAAGCTTCGTCCCTAGCGAAAGGAG
TTTACAACCCGAAGGCCTTCATCCCCCACGCGGCGTCGCTGCATCAGGCTTGCGCCCATTGTGCAATA
TTCCCCA_L6_Corynebacterium_1"] = "#dcc089"

h["CACGTAGTTAGCCGGTGCTTCTTCTCTAGGTACCGTCACATAACGCTTCGTCCCTAGCGAAAGGA
GTTTACAACCCGAAGGCCGTCATCCCCCACGCGGCGTCGCTGCATCAGGCTTGCGCCCATTGTGCAAT
ATTCCCCA_L6_Corynebacterium_1"] = "#8f6c57"

h["CACGTAGTTAGCCGGTGCTTCTTCTCCAGGTACCGTCACAAAAACGCTTCGTCCCTAGCGAAAGG
AGTTTACAACCCGAAGGCCGTCATCCCCCACGCGGCGTCGCTGCATCAGGCTTGCGCCCATTGTGCAA
TATTCCCCA_L6_Corynebacterium_1"] = "#ffd599"

h["CACGTAGTTAGCCGGTGCTTCTTCTCCAGGTACCGTCACAAAAAGCTTCGTCCCTAGCGAAAGGA
GTTTACAACCCGAAGGCCTTCATCCCCCACGCGGCGTCGCTGCATCAGGGTTTCCCCCATTGTGCAAT
ATTCCCCA_L6_Corynebacterium_1"] = "#ef9234"

h["CACGTAGTTAGCCGGTGCTTCTTCTCCAGGTACCGTCACAAAACGCTTCGTCCCTGGCGAAAGGA
GTTTACAACCCGAAGGCCGTCGTCCCCCACGCGGCGTCGCTGCATCAGGCTTGCGCCCATTGTGCAAT
ATTCCCCA_L6_Corynebacterium_1"] = "#ce5247"

h["CACGTAGTTAGCCGGTGCTTCTTCTCTAGGTACCGTCACTTGCGCTTCGTCCCTAGCGAAAGGAG
TTTACAACCCGAAGGCCGTCATCCCCCACGCGGCGTCGCTGCATCAGGCTTGCGCCCATTGTGCAATA
TTCCCCA_L6_Corynebacterium_1"] = "#7a5c3c"

h["CACGTAGTTAGCCGGTGCTTCTTCTCCAAATACCGTCAAAATTCGTCTTTGGCGAAAGGAGTTTA
CAACCCGAAGGCCGTCATCCCCCACGCGGCGTCGCTGCATCAGGCTTCCGCCCATTGTGCAATATTCC
CCA_L6_Corynebacterium_1"] = "#a17e13"

```
h["CACGTAGTTAGCCGGTGCTTCTTATCCAGGTACCGTCACCAAAAGGCTTCTTCCCTAGCGAAAGG
AGTTTACAACCCGAAGGCCGTCATCCCCCACGCGGCGTCGCTGCATCAGGCTTGCGCCCATTGTGCAA
TATTCCCCA_L6_Corynebacterium_1"] = "#ff856e"

h["CACGTAGTTAGCCGGTGCTTCTTCTCCAGGTACCGTCACAAAACGCTTCGTCCCTGGCGAAAGGA
GTTTACAACCCGAAGGCCGTCATCCCCCACGCAGCGTCGCTGCATCAGGCTTGCGCCCATTGTGCAAT
ATTCCCCA_L6_Corynebacterium_1"] = "#c5a461"

h["CACGTAGTTAGCCGGTGCTTCTTATCTAGGTACCGTCACAACAAAGCTTCGTCCCTAGCGAAAGG
AGTTTACAACCCGAAGGCCTTCATCCCCCACGCGGCGTCGCTGCATCAGGCTTGCGCCCATTGTGCAA
TATTCCCCG_L6_Corynebacterium_1"] = "#fa7062"

h["CACGTAGTTAGCCGGTGCTTCTTATACAGGTACCGTCACTTACGCTTCGTCCCTGTCGAAAGGAG
TTTACAACCCGAAGGCCGTCATCCCCCACGCGGCGTCGCTGCATCAGGCTTCCGCCCATTGTGCAATA
TTCCCCA_L6_Corynebacterium_1"] = "#805b1d"

h["CACGTAGTTAGCCGGTGCTTCTTATCCAGGTACCGTCACTTACGCTTCGTCCCTGGCGAAAGGAG
TTTACAACCCGAAGGCCGTCATCCCCCACGCGGCGTCGCTGCATCAGGCTTGCGCCCATTGTGCAATA
TTCCCCA_L6_Corynebacterium_1"] = "#de8e82"

h["CACGTAGTTAGCCGGTGCTTCTTCTACCACTACCGTCACCCGAAGGCTTCGTCATGGCTGAAAGG
AGTTTACAACCCGAAGGCCGTCATCCCCCACGCGGCGTCGCTGCATCAGGCTTGCGCCCATTGTGCAA
TATTCCCCA_L6_Corynebacterium_1"] = "#ffb18f"

h["CACGTAGTTAGCCGGTGCTTCTTATCTAGGTACCGTCACAAAAAAGCTTCGTCCCTAGCGAAAGG
AGTTTACAACCCGAAGGCCTTCATCCCCCACGCGGCGTCGCTGCATCAGGCTTGCGCCCATTGTACAA
TATTCCCCA_L6_Corynebacterium_1"] = "#a08b6b"

h["CACGTAGTTAGCCGGTACTTCTTATCTAGGTACCGTCACAAAAAGCTTCGTCCCTAGCGAAAGGA
GTTTACAACCCGAAGGCCTTCATCCCCCACGCGGCGTCGCTGCATCAGGCTTGCGCCCATTGTGCAAT
ATTCCCCA_L6_Corynebacterium_1"] = "#ea6c3b"

h["CACGTAGTTAGCCGGTGCTTCTTATCTAGATACCGTCACAAAAAGCTTCGTCCCTAGCGAAAGGA
GTTTACAACCCGAAGGCCGTCATCCCCCACGCGGCGTCGCTGCATCAGGCTTGCGCCCATTGTGCAAT
ATTCCCCA_L6_Corynebacterium_1"] = "#a54412"

h["CACGTAGTTAGCCGGTGCTTCTTATCCAGGTACCGTCACAAAACGCTTCGTCCCTGGCGAAAGGA
GTTTACAACCCGAAGGCCGTCATCCCCCACGCGGCGTCGCTGCATCAGGCTTGCGCCCATTGTGCAAT
ATTCCCCA_L6_Corynebacterium_1"] = "#e9bbab"
  h["Taxa < 1%"] = "#808080"
  h["other"] = "#808080"
  h["NA"] = "#181818"
  #Make a vector of colours to return
  colours <- character(0)
  for (i in 1:length(x)){
    if (x[i] == "Taxa < 1%") {
      col <- h[[x[i]]]
    } else {
      x.sub  <- str_split(x[i], "_L6_")[[1]][2]
      if (!str_detect(x.sub, "Corynebacterium")) {
        col <- "#808080" #181818"
      } else {
        col <- h[[x[i]]]
```

```
      }
    }
      if (is.null(col)) {
        print(x[i])
      }
      colours <- c(colours, col)
  }
  return(colours)
}

#myLabels_L6 function
myLabels_L6 <- function(x,y) {
  #Setup hash
  h <- hash()

h["Root.p__Actinobacteria.c__Actinobacteria.o__Actinomycetales.f__Ac
tinomycetaceae.g__Actinobaculum"] = paste("g__Actinobaculum (",
format(round(100*mean(y$Root.p__Actinobacteria.c__Actinobacteria.o__
Actinomycetales.f__Actinomycetaceae.g__Actinobaculum),2),
nsmall=2),"%)", sep="")

h["Root.p__Actinobacteria.c__Actinobacteria.o__Actinomycetales.f__Ac
tinomycetaceae.g__Actinomyces"] = paste("g__Actinomyces (",
format(round(100*mean(y$Root.p__Actinobacteria.c__Actinobacteria.o__
Actinomycetales.f__Actinomycetaceae.g__Actinomyces),2),
nsmall=2),"%)", sep="")

h["Root.p__Actinobacteria.c__Actinobacteria.o__Coriobacteriales.f__C
oriobacteriaceae.g__Adlercreutzia"] = paste("g__Adlercreutzia (",
format(round(100*mean(y$Root.p__Actinobacteria.c__Actinobacteria.o__
Actinomycetales.f__Actinomycetaceae.g__Adlercreutzia),2),
nsmall=2),"%)", sep="")

h["Root.p__Actinobacteria.c__Actinobacteria.o__Actinomycetales.f__Ac
tinomycetaceae.g__Arcanobacterium"] = paste("g__Arcanobacterium (",
format(round(100*mean(y$Root.p__Actinobacteria.c__Actinobacteria.o__
Actinomycetales.f__Actinomycetaceae.g__Arcanobacterium),2),
nsmall=2),"%)", sep="")

h["Root.p__Actinobacteria.c__Actinobacteria.o__Actinomycetales.f__Br
evibacteriaceae.g__Brevibacterium"] = paste("g__Brevibacterium (",
format(round(100*mean(y$Root.p__Actinobacteria.c__Actinobacteria.o__
Actinomycetales.f__Brevibacteriaceae.g__Brevibacterium),2),
nsmall=2),"%)", sep="")

h["Root.p__Actinobacteria.c__Actinobacteria.o__Actinomycetales.f__Co
rynebacteriaceae.g__"] = paste("f__Corynebacteriaceae (",
format(round(100*mean(y$Root.p__Actinobacteria.c__Actinobacteria.o__
Actinomycetales.f__Corynebacteriaceae.g__),2), nsmall=2),"%)",
sep="")

h["Root.p__Actinobacteria.c__Actinobacteria.o__Actinomycetales.f__Co
rynebacteriaceae.g__Corynebacterium"] = paste("g__Corynebacterium
(",
```

```
format(round(100*mean(y$Root.p__Actinobacteria.c__Actinobacteria.o__
Actinomycetales.f__Corynebacteriaceae.g__Corynebacterium),2),
nsmall=2),"%)", sep="")

h["Root.p__Actinobacteria.c__Actinobacteria.o__Actinomycetales.f__In
trasporangiaceae.g__"] = paste("f__Intrasporangiaceae (",
format(round(100*mean(y$Root.p__Actinobacteria.c__Actinobacteria.o__
Actinomycetales.f__Intrasporangiaceae.g__),2), nsmall=2),"%)",
sep="")

h["Root.p__Actinobacteria.c__Actinobacteria.o__Actinomycetales.f__Mi
crococcaceae.g__Rothia"] = paste("g__Rothia (",
format(round(100*mean(y$Root.p__Actinobacteria.c__Actinobacteria.o__
Actinomycetales.f__Micrococcaceae.g__Rothia),2), nsmall=2),"%)",
sep="")

h["Root.p__Actinobacteria.c__Actinobacteria.o__Actinomycetales.f__Mi
cromonosporaceae.Other"] = paste("f__Micromonosporaceae (",
format(round(100*mean(y$Root.p__Actinobacteria.c__Actinobacteria.o__
Actinomycetales.f__Micromonosporaceae.Other),2), nsmall=2),"%)",
sep="")

h["Root.p__Actinobacteria.c__Actinobacteria.o__Actinomycetales.f__Pr
omicromonosporaceae.g__Cellulosimicrobium"] =
paste("g__Cellulosimicrobium (",
format(round(100*mean(y$Root.p__Actinobacteria.c__Actinobacteria.o__
Actinomycetales.f__Promicromonosporaceae.g__Cellulosimicrobium),2),
nsmall=2),"%)", sep="")

h["Root.p__Actinobacteria.c__Actinobacteria.o__Actinomycetales.f__Pr
opionibacteriaceae.g__Propionibacterium"] =
paste("g__Propionibacterium (",
format(round(100*mean(y$Root.p__Actinobacteria.c__Actinobacteria.o__
Actinomycetales.f__Propionibacteriaceae.g__Propionibacterium),2),
nsmall=2),"%)", sep="")

h["Root.p__Actinobacteria.c__Actinobacteria.o__Actinomycetales.f__Pr
opionibacteriaceae.Other"] = paste("f__Propionibacteriaceae (",
format(round(100*mean(y$Root.p__Actinobacteria.c__Actinobacteria.o__
Actinomycetales.f__Propionibacteriaceae.Other),2), nsmall=2),"%)",
sep="")

h["Root.p__Actinobacteria.c__Actinobacteria.o__Actinomycetales.Other
.Other"] = paste("o__Actinomycetales (",
format(round(100*mean(y$Root.p__Actinobacteria.c__Actinobacteria.o__
Actinomycetales.Other.Other),2), nsmall=2),"%)", sep="")

h["Root.p__Actinobacteria.c__Actinobacteria.o__Bifidobacteriales.f__
Bifidobacteriaceae.g__Bifidobacterium"] = paste("g__Bifidobacterium
(",
format(round(100*mean(y$Root.p__Actinobacteria.c__Actinobacteria.o__
Bifidobacteriales.f__Bifidobacteriaceae.g__Bifidobacterium),2),
nsmall=2),"%)", sep="")
```

```
h["Root.p__Actinobacteria.c__Actinobacteria.o__Bifidobacteriales.f__
Bifidobacteriaceae.g__Gardnerella"] = paste("g__Gardnerella (",
format(round(100*mean(y$Root.p__Actinobacteria.c__Actinobacteria.o__
Bifidobacteriales.f__Bifidobacteriaceae.g__Gardnerella),2),
nsmall=2),"%)", sep="")

h["Root.p__Actinobacteria.c__Actinobacteria.o__Bifidobacteriales.f__
Bifidobacteriaceae.g__Scardovia"] = paste("g__Scardovia (",
format(round(100*mean(y$Root.p__Actinobacteria.c__Actinobacteria.o__
Bifidobacteriales.f__Bifidobacteriaceae.g__Scardovia),2),
nsmall=2),"%)", sep="")

h["Root.p__Actinobacteria.c__Actinobacteria.o__Coriobacteriales.f__C
oriobacteriaceae.g__Collinsella"] = paste("g__Collinsella (",
format(round(100*mean(y$Root.p__Actinobacteria.c__Actinobacteria.o__
Coriobacteriales.f__Coriobacteriaceae.g__Collinsella),2),
nsmall=2),"%)", sep="")
  h["Root.p__Bacteroidetes.c__Bacteroidia.o__Bacteroidales.f__.g__"]
= paste("o__Bacteroidales (",
format(round(100*mean(y$Root.p__Bacteroidetes.c__Bacteroidia.o__Bact
eroidales.f__.g__),2), nsmall=2),"%)", sep="")

h["Root.p__Bacteroidetes.c__Bacteroidia.o__Bacteroidales.f__Bacteroi
daceae.g__Bacteroides"] = paste("g__Bacteroides (",
format(round(100*mean(y$Root.p__Bacteroidetes.c__Bacteroidia.o__Bact
eroidales.f__Bacteroidaceae.g__Bacteroides),2), nsmall=2),"%)",
sep="")

h["Root.p__Bacteroidetes.c__Bacteroidia.o__Bacteroidales.f__Porphyro
monadaceae.g__"] = paste("f__Porphyromonadaceae (",
format(round(100*mean(y$Root.p__Bacteroidetes.c__Bacteroidia.o__Bact
eroidales.f__Porphyromonadaceae.g__),2), nsmall=2),"%)", sep="")

h["Root.p__Bacteroidetes.c__Bacteroidia.o__Bacteroidales.f__Porphyro
monadaceae.g__Parabacteroides"] = paste("g__Parabacteroides (",
format(round(100*mean(y$Root.p__Bacteroidetes.c__Bacteroidia.o__Bact
eroidales.f__Porphyromonadaceae.g__Parabacteroides),2),
nsmall=2),"%)", sep="")

h["Root.p__Bacteroidetes.c__Bacteroidia.o__Bacteroidales.f__Porphyro
monadaceae.g__Porphyromonas"] = paste("g__Porphyromonas (",
format(round(100*mean(y$Root.p__Bacteroidetes.c__Bacteroidia.o__Bact
eroidales.f__Porphyromonadaceae.g__Porphyromonas),2),
nsmall=2),"%)", sep="")

h["Root.p__Bacteroidetes.c__Bacteroidia.o__Bacteroidales.f__Prevotel
laceae.g__Prevotella"] = paste("g__Prevotella (",
format(round(100*mean(y$Root.p__Bacteroidetes.c__Bacteroidia.o__Bact
eroidales.f__Prevotellaceae.g__Prevotella),2), nsmall=2),"%)",
sep="")

h["Root.p__Bacteroidetes.c__Bacteroidia.o__Bacteroidales.f__Rikenell
aceae.g__Alistipes"] = paste("g__Alistipes (",
format(round(100*mean(y$Root.p__Bacteroidetes.c__Bacteroidia.o__Bact
```

```
eroidales.f__Rikenellaceae.g__Alistipes),2), nsmall=2),"%)", sep="")

h["Root.p__Bacteroidetes.c__Flavobacteria.o__Flavobacteriales.f__Fla
vobacteriaceae.g__Capnocytophaga"] = paste("g__Capnocytophaga (",
format(round(100*mean(y$Root.p__Bacteroidetes.c__Flavobacteria.o__Fl
avobacteriales.f__Flavobacteriaceae.g__Capnocytophaga),2),
nsmall=2),"%)", sep="")

h["Root.p__Bacteroidetes.c__Flavobacteria.o__Flavobacteriales.f__Fla
vobacteriaceae.g__Chryseobacterium"] = paste("g__Chryseobacterium
(",
format(round(100*mean(y$Root.p__Bacteroidetes.c__Flavobacteria.o__Fl
avobacteriales.f__Flavobacteriaceae.g__Chryseobacterium),2),
nsmall=2),"%)", sep="")

h["Root.p__Bacteroidetes.c__Sphingobacteria.o__Sphingobacteriales.f_
_Flexibacteraceae.g__"] = paste("f__Flexibacteraceae (",
format(round(100*mean(y$Root.p__Bacteroidetes.c__Sphingobacteria.o__
Sphingobacteriales.f__Flexibacteraceae.g__),2), nsmall=2),"%)",
sep="")

h["Root.p__Bacteroidetes.c__Sphingobacteria.o__Sphingobacteriales.f_
_Sphingobacteriaceae.g__Sphingobacterium"] =
paste("g__Sphingobacterium (",
format(round(100*mean(y$Root.p__Bacteroidetes.c__Sphingobacteria.o__
Sphingobacteriales.f__Sphingobacteriaceae.g__Sphingobacterium),2),
nsmall=2),"%)", sep="")
  h["Root.p__Chloroflexi.c__SOGA31.o__.f__.g__"] = paste("c__SOGA31
(",
format(round(100*mean(y$Root.p__Chloroflexi.c__SOGA31.o__.f__.g__),2
), nsmall=2),"%)", sep="")
  h["Root.p__Crenarchaeota.Other.Other.Other.Other"] =
paste("p__Crenarchaeota (",
format(round(100*mean(y$Root.p__Crenarchaeota.Other.Other.Other.Othe
r),2), nsmall=2),"%)", sep="")
  h["Root.p__Cyanobacteria.c__Chloroplast.o__Streptophyta.f__.g__"]
= paste("o__Streptophyta (",
format(round(100*mean(y$Root.p__Cyanobacteria.c__Chloroplast.o__Stre
ptophyta.f__.g__),2), nsmall=2),"%)", sep="")

h["Root.p__Cyanobacteria.c__Nostocophycideae.o__Nostocales.f__Nostoc
aceae.Other"] = paste("f__Nostocaceae (",
format(round(100*mean(y$Root.p__Cyanobacteria.c__Nostocophycideae.o_
_Nostocales.f__Nostocaceae.Other),2), nsmall=2),"%)", sep="")

h["Root.p__Cyanobacteria.c__Synechococcophycideae.o__Pseudanabaenale
s.f__Pseudanabaenaceae.g__Halomicronema"] = paste("g__Halomicronema
(",
format(round(100*mean(y$Root.p__Cyanobacteria.c__Synechococcophycide
ae.o__Pseudanabaenales.f__Pseudanabaenaceae.g__Halomicronema),2),
nsmall=2),"%)", sep="")

h["Root.p__Firmicutes.c__Bacilli.o__Bacillales.f__Bacillaceae.g__Ano
xybacillus"] = paste("g__Anoxybacillus (",
```

```
format(round(100*mean(y$Root.p__Firmicutes.c__Bacilli.o__Bacillales.
f__Bacillaceae.g__Anoxybacillus),2), nsmall=2),"%)", sep="")

h["Root.p__Firmicutes.c__Bacilli.o__Bacillales.f__Bacillaceae.g__Bac
illus"] = paste("g__Bacillus (",
format(round(100*mean(y$Root.p__Firmicutes.c__Bacilli.o__Bacillales.
f__Bacillaceae.g__Bacillus),2), nsmall=2),"%)", sep="")

h["Root.p__Firmicutes.c__Bacilli.o__Bacillales.f__Paenibacillaceae.g
__Paenibacillus"] = paste("g__Paenibacillus (",
format(round(100*mean(y$Root.p__Firmicutes.c__Bacilli.o__Bacillales.
f__Paenibacillaceae.g__Paenibacillus),2), nsmall=2),"%)", sep="")

h["Root.p__Firmicutes.c__Bacilli.o__Bacillales.f__Staphylococcaceae.
g__Staphylococcus"] = paste("g__Staphylococcus (",
format(round(100*mean(y$Root.p__Firmicutes.c__Bacilli.o__Bacillales.
f__Staphylococcaceae.g__Staphylococcus),2), nsmall=2),"%)", sep="")

h["Root.p__Firmicutes.c__Bacilli.o__Gemellales.f__Gemellaceae.g__Gem
ella"] = paste("g__Gemella (",
format(round(100*mean(y$Root.p__Firmicutes.c__Bacilli.o__Gemellales.
f__Gemellaceae.g__Gemella),2), nsmall=2),"%)", sep="")

h["Root.p__Firmicutes.c__Bacilli.o__Lactobacillales.f__Aerococcaceae
.g__Aerococcus"] = paste("g__Aerococcus (",
format(round(100*mean(y$Root.p__Firmicutes.c__Bacilli.o__Lactobacill
ales.f__Aerococcaceae.g__Aerococcus),2), nsmall=2),"%)", sep="")

h["Root.p__Firmicutes.c__Bacilli.o__Lactobacillales.f__Aerococcaceae
.g__Facklamia"] = paste("g__Facklamia (",
format(round(100*mean(y$Root.p__Firmicutes.c__Bacilli.o__Lactobacill
ales.f__Aerococcaceae.g__Facklamia),2), nsmall=2),"%)", sep="")

h["Root.p__Firmicutes.c__Bacilli.o__Lactobacillales.f__Carnobacteria
ceae.g__"] = paste("f__Carnobacteriaceae (",
format(round(100*mean(y$Root.p__Firmicutes.c__Bacilli.o__Lactobacill
ales.f__Carnobacteriaceae.g__),2), nsmall=2),"%)", sep="")

h["Root.p__Firmicutes.c__Bacilli.o__Lactobacillales.f__Carnobacteria
ceae.g__Granulicatella"] = paste("g__Granulicatella (",
format(round(100*mean(y$Root.p__Firmicutes.c__Bacilli.o__Lactobacill
ales.f__Carnobacteriaceae.g__Granulicatella),2), nsmall=2),"%)",
sep="")

h["Root.p__Firmicutes.c__Bacilli.o__Lactobacillales.f__Enterococcace
ae.g__Enterococcus"] = paste("g__Enterococcus (",
format(round(100*mean(y$Root.p__Firmicutes.c__Bacilli.o__Lactobacill
ales.f__Enterococcaceae.g__Enterococcus),2), nsmall=2),"%)", sep="")

h["Root.p__Firmicutes.c__Bacilli.o__Lactobacillales.f__Lactobacillac
eae.g__Lactobacillus"] = paste("g__Lactobacillus (",
format(round(100*mean(y$Root.p__Firmicutes.c__Bacilli.o__Lactobacill
ales.f__Lactobacillaceae.g__Lactobacillus),2), nsmall=2),"%)",
sep="")
```

```
h["Root.p__Firmicutes.c__Bacilli.o__Lactobacillales.f__Leuconostocac
eae.g__Weissella"] = paste("g__Weissella (",
format(round(100*mean(y$Root.p__Firmicutes.c__Bacilli.o__Lactobacill
ales.f__Leuconostocaceae.g__Weissella),2), nsmall=2),"%)", sep="")

h["Root.p__Firmicutes.c__Bacilli.o__Lactobacillales.f__Streptococcac
eae.g__Lactococcus"] = paste("g__Lactococcus (",
format(round(100*mean(y$Root.p__Firmicutes.c__Bacilli.o__Lactobacill
ales.f__Streptococcaceae.g__Lactococcus),2), nsmall=2),"%)", sep="")

h["Root.p__Firmicutes.c__Bacilli.o__Lactobacillales.f__Streptococcac
eae.g__Streptococcus"] = paste("g__Streptococcus (",
format(round(100*mean(y$Root.p__Firmicutes.c__Bacilli.o__Lactobacill
ales.f__Streptococcaceae.g__Streptococcus),2), nsmall=2),"%)",
sep="")

h["Root.p__Firmicutes.c__Bacilli.o__Lactobacillales.f__Streptococcac
eae.Other"] = paste("f__Streptococcaceae (",
format(round(100*mean(y$Root.p__Firmicutes.c__Bacilli.o__Lactobacill
ales.f__Streptococcaceae.Other),2), nsmall=2),"%)", sep="")

h["Root.p__Firmicutes.c__Bacilli.o__Turicibacterales.f__Turicibacter
aceae.g__"] = paste("f__Turicibacteraceae (",
format(round(100*mean(y$Root.p__Firmicutes.c__Bacilli.o__Turicibacte
rales.f__Turicibacteraceae.g__),2), nsmall=2),"%)", sep="")

h["Root.p__Firmicutes.c__Bacilli.o__Turicibacterales.f__Turicibacter
aceae.g__Turicibacter"] = paste("g__Turicibacter (",
format(round(100*mean(y$Root.p__Firmicutes.c__Bacilli.o__Turicibacte
rales.f__Turicibacteraceae.g__Turicibacter),2), nsmall=2),"%)",
sep="")
  h["Root.p__Firmicutes.c__Clostridia.o__Clostridiales.f__.g__"] =
paste("o__Clostridiales (",
format(round(100*mean(y$Root.p__Firmicutes.c__Clostridia.o__Clostrid
iales.f__.g__),2), nsmall=2),"%)", sep="")

h["Root.p__Firmicutes.c__Clostridia.o__Clostridiales.f__Catabacteria
ceae.g__"] = paste("f__Catabacteriaceae (",
format(round(100*mean(y$Root.p__Firmicutes.c__Clostridia.o__Clostrid
iales.f__Catabacteriaceae.g__),2), nsmall=2),"%)", sep="")

h["Root.p__Firmicutes.c__Clostridia.o__Clostridiales.f__Clostridiace
ae.g__Clostridium"] = paste("g__Clostridium (",
format(round(100*mean(y$Root.p__Firmicutes.c__Clostridia.o__Clostrid
iales.f__Clostridiaceae.g__Clostridium),2), nsmall=2),"%)", sep="")

h["Root.p__Firmicutes.c__Clostridia.o__Clostridiales.f__Clostridiace
ae.Other"] = paste("f__Clostridiaceae (",
format(round(100*mean(y$Root.p__Firmicutes.c__Clostridia.o__Clostrid
iales.f__Clostridiaceae.Other),2), nsmall=2),"%)", sep="")

h["Root.p__Firmicutes.c__Clostridia.o__Clostridiales.f__Clostridiale
sFamilyXI.IncertaeSedis.g__"] =
```

```
paste("f__ClostridialesFamilyXI.IncertaeSedis (",
format(round(100*mean(y$Root.p__Firmicutes.c__Clostridia.o__Clostrid
iales.f__ClostridialesFamilyXI.IncertaeSedis.g__),2),
nsmall=2),"%)", sep="")

h["Root.p__Firmicutes.c__Clostridia.o__Clostridiales.f__Clostridiale
sFamilyXI.IncertaeSedis.g__Anaerococcus"] = paste("g__Anaerococcus
(",
format(round(100*mean(y$Root.p__Firmicutes.c__Clostridia.o__Clostrid
iales.f__ClostridialesFamilyXI.IncertaeSedis.g__Anaerococcus),2),
nsmall=2),"%)", sep="")

h["Root.p__Firmicutes.c__Clostridia.o__Clostridiales.f__Clostridiale
sFamilyXI.IncertaeSedis.g__Finegoldia"] = paste("g__Finegoldia (",
format(round(100*mean(y$Root.p__Firmicutes.c__Clostridia.o__Clostrid
iales.f__ClostridialesFamilyXI.IncertaeSedis.g__Finegoldia),2),
nsmall=2),"%)", sep="")

h["Root.p__Firmicutes.c__Clostridia.o__Clostridiales.f__Clostridiale
sFamilyXI.IncertaeSedis.g__Peptoniphilus"] = paste("g__Peptoniphilus
(",
format(round(100*mean(y$Root.p__Firmicutes.c__Clostridia.o__Clostrid
iales.f__ClostridialesFamilyXI.IncertaeSedis.g__Peptoniphilus),2),
nsmall=2),"%)", sep="")

h["Root.p__Firmicutes.c__Clostridia.o__Clostridiales.f__Lachnospirac
eae.g__"] = paste("f__Lachnospiraceae (",
format(round(100*mean(y$Root.p__Firmicutes.c__Clostridia.o__Clostrid
iales.f__Lachnospiraceae.g__),2), nsmall=2),"%)", sep="")

h["Root.p__Firmicutes.c__Clostridia.o__Clostridiales.f__Lachnospirac
eae.g__Blautia"] = paste("g__Blautia (",
format(round(100*mean(y$Root.p__Firmicutes.c__Clostridia.o__Clostrid
iales.f__Lachnospiraceae.g__Blautia),2), nsmall=2),"%)", sep="")

h["Root.p__Firmicutes.c__Clostridia.o__Clostridiales.f__Lachnospirac
eae.g__Catonella"] = paste("g__Catonella (",
format(round(100*mean(y$Root.p__Firmicutes.c__Clostridia.o__Clostrid
iales.f__Lachnospiraceae.g__Catonella),2), nsmall=2),"%)", sep="")

h["Root.p__Firmicutes.c__Clostridia.o__Clostridiales.f__Lachnospirac
eae.g__Clostridium"] = paste("g__Clostridium (",
format(round(100*mean(y$Root.p__Firmicutes.c__Clostridia.o__Clostrid
iales.f__Lachnospiraceae.g__Clostridium),2), nsmall=2),"%)", sep="")

h["Root.p__Firmicutes.c__Clostridia.o__Clostridiales.f__Lachnospirac
eae.g__Lachnobacterium"] = paste("g__Lachnobacterium (",
format(round(100*mean(y$Root.p__Firmicutes.c__Clostridia.o__Clostrid
iales.f__Lachnospiraceae.g__Lachnobacterium),2), nsmall=2),"%)",
sep="")

h["Root.p__Firmicutes.c__Clostridia.o__Clostridiales.f__Lachnospirac
eae.g__Lachnospira"] = paste("g__Lachnospira (",
format(round(100*mean(y$Root.p__Firmicutes.c__Clostridia.o__Clostrid
```

```
iales.f__Lachnospiraceae.g__Lachnospira),2), nsmall=2),"%)", sep="")

h["Root.p__Firmicutes.c__Clostridia.o__Clostridiales.f__Lachnospirac
eae.g__Pseudobutyrivibrio"] = paste("g__Pseudobutyrivibrio (",
format(round(100*mean(y$Root.p__Firmicutes.c__Clostridia.o__Clostrid
iales.f__Lachnospiraceae.g__Pseudobutyrivibrio),2), nsmall=2),"%)",
sep="")

h["Root.p__Firmicutes.c__Clostridia.o__Clostridiales.f__Lachnospirac
eae.g__Ruminococcus"] = paste("g__Ruminococcus (",
format(round(100*mean(y$Root.p__Firmicutes.c__Clostridia.o__Clostrid
iales.f__Lachnospiraceae.g__Ruminococcus),2), nsmall=2),"%)",
sep="")

h["Root.p__Firmicutes.c__Clostridia.o__Clostridiales.f__Lachnospirac
eae.g__Shuttleworthia"] = paste("g__Shuttleworthia (",
format(round(100*mean(y$Root.p__Firmicutes.c__Clostridia.o__Clostrid
iales.f__Lachnospiraceae.g__Shuttleworthia),2), nsmall=2),"%)",
sep="")

h["Root.p__Firmicutes.c__Clostridia.o__Clostridiales.f__Lachnospirac
eae.Other"] = paste("f__Lachnospiraceae (",
format(round(100*mean(y$Root.p__Firmicutes.c__Clostridia.o__Clostrid
iales.f__Lachnospiraceae.Other),2), nsmall=2),"%)", sep="")

h["Root.p__Firmicutes.c__Clostridia.o__Clostridiales.f__Ruminococcac
eae.g__"] = paste("f__Ruminococcaceae (",
format(round(100*mean(y$Root.p__Firmicutes.c__Clostridia.o__Clostrid
iales.f__Ruminococcaceae.g__),2), nsmall=2),"%)", sep="")

h["Root.p__Firmicutes.c__Clostridia.o__Clostridiales.f__Ruminococcac
eae.Other"] = paste("f__Ruminococcaceae (",
format(round(100*mean(y$Root.p__Firmicutes.c__Clostridia.o__Clostrid
iales.f__Ruminococcaceae.Other),2), nsmall=2),"%)", sep="")

h["Root.p__Firmicutes.c__Clostridia.o__Clostridiales.f__Ruminococcac
eae.g__Bacteroides"] = paste("g__Bacteroides (",
format(round(100*mean(y$Root.p__Firmicutes.c__Clostridia.o__Clostrid
iales.f__Ruminococcaceae.g__Bacteroides),2), nsmall=2),"%)", sep="")

h["Root.p__Firmicutes.c__Clostridia.o__Clostridiales.f__Ruminococcac
eae.g__Clostridium"] = paste("g__Clostridium (",
format(round(100*mean(y$Root.p__Firmicutes.c__Clostridia.o__Clostrid
iales.f__Ruminococcaceae.g__Clostridium),2), nsmall=2),"%)", sep="")

h["Root.p__Firmicutes.c__Clostridia.o__Clostridiales.f__Ruminococcac
eae.g__Eubacterium"] = paste("g__Eubacterium (",
format(round(100*mean(y$Root.p__Firmicutes.c__Clostridia.o__Clostrid
iales.f__Ruminococcaceae.g__Eubacterium),2), nsmall=2),"%)", sep="")

h["Root.p__Firmicutes.c__Clostridia.o__Clostridiales.f__Ruminococcac
eae.g__Faecalibacterium"] = paste("g__Faecalibacterium (",
format(round(100*mean(y$Root.p__Firmicutes.c__Clostridia.o__Clostrid
iales.f__Ruminococcaceae.g__Faecalibacterium),2), nsmall=2),"%)",
```

```
sep="")

h["Root.p__Firmicutes.c__Clostridia.o__Clostridiales.f__Ruminococcac
eae.g__Oscillospira"] = paste("g__Oscillospira (",
format(round(100*mean(y$Root.p__Firmicutes.c__Clostridia.o__Clostrid
iales.f__Ruminococcaceae.g__Oscillospira),2), nsmall=2),"%)",
sep="")

h["Root.p__Firmicutes.c__Clostridia.o__Clostridiales.f__Ruminococcac
eae.g__Ruminococcus"] = paste("g__Ruminococcus (",
format(round(100*mean(y$Root.p__Firmicutes.c__Clostridia.o__Clostrid
iales.f__Ruminococcaceae.g__Ruminococcus),2), nsmall=2),"%)",
sep="")

h["Root.p__Firmicutes.c__Clostridia.o__Clostridiales.f__Ruminococcac
eae.g__Subdoligranulum"] = paste("g__Subdoligranulum (",
format(round(100*mean(y$Root.p__Firmicutes.c__Clostridia.o__Clostrid
iales.f__Ruminococcaceae.g__Subdoligranulum),2), nsmall=2),"%)",
sep="")

h["Root.p__Firmicutes.c__Clostridia.o__Clostridiales.f__Veillonellac
eae.g__Dialister"] = paste("g__Dialister (",
format(round(100*mean(y$Root.p__Firmicutes.c__Clostridia.o__Clostrid
iales.f__Veillonellaceae.g__Dialister),2), nsmall=2),"%)", sep="")

h["Root.p__Firmicutes.c__Clostridia.o__Clostridiales.f__Veillonellac
eae.g__Mitsuokella"] = paste("g__Mitsuokella (",
format(round(100*mean(y$Root.p__Firmicutes.c__Clostridia.o__Clostrid
iales.f__Veillonellaceae.g__Mitsuokella),2), nsmall=2),"%)", sep="")

h["Root.p__Firmicutes.c__Clostridia.o__Clostridiales.f__Veillonellac
eae.g__Veillonella"] = paste("g__Veillonella (",
format(round(100*mean(y$Root.p__Firmicutes.c__Clostridia.o__Clostrid
iales.f__Veillonellaceae.g__Veillonella),2), nsmall=2),"%)", sep="")
  h["Root.p__Firmicutes.c__Clostridia.o__Clostridiales.Other.Other"]
= paste("o__Clostridiales (",
format(round(100*mean(y$Root.p__Firmicutes.c__Clostridia.o__Clostrid
iales.Other.Other),2), nsmall=2),"%)", sep="")
  h["Root.p__Firmicutes.c__Clostridia.Other.Other.Other"] =
paste("c__Clostridia (",
format(round(100*mean(y$Root.p__Firmicutes.c__Clostridia.Other.Other
.Other),2), nsmall=2),"%)", sep="")

h["Root.p__Fusobacteria.c__Fusobacteria.o__Fusobacteriales.f__Fusoba
cteriaceae.g__Fusobacterium"] = paste("g__Fusobacterium (",
format(round(100*mean(y$Root.p__Fusobacteria.c__Fusobacteria.o__Fuso
bacteriales.f__Fusobacteriaceae.g__Fusobacterium),2),
nsmall=2),"%)", sep="")

h["Root.p__Fusobacteria.c__Fusobacteria.o__Fusobacteriales.f__Fusoba
cteriaceae.g__Leptotrichia"] = paste("g__Leptotrichia (",
format(round(100*mean(y$Root.p__Fusobacteria.c__Fusobacteria.o__Fuso
bacteriales.f__Fusobacteriaceae.g__Leptotrichia),2), nsmall=2),"%)",
sep="")
```

```
h["Root.p__Fusobacteria.c__Fusobacteria.o__Fusobacteriales.f__Fusoba
cteriaceae.g__Sneathia"] = paste("g__Sneathia (",
format(round(100*mean(y$Root.p__Fusobacteria.c__Fusobacteria.o__Fuso
bacteriales.f__Fusobacteriaceae.g__Sneathia),2), nsmall=2),"%)",
sep="")

h["Root.p__Fusobacteria.c__Fusobacteria.o__Fusobacteriales.f__Fusoba
cteriaceae.Other"] = paste("f__Fusobacteriaceae (",
format(round(100*mean(y$Root.p__Fusobacteria.c__Fusobacteria.o__Fuso
bacteriales.f__Fusobacteriaceae.Other),2), nsmall=2),"%)", sep="")

h["Root.p__Proteobacteria.c__Alphaproteobacteria.o__Caulobacterales.
f__Caulobacteraceae.g__Caulobacter"] = paste("g__Caulobacter (",
format(round(100*mean(y$Root.p__Proteobacteria.c__Alphaproteobacteri
a.o__Caulobacterales.f__Caulobacteraceae.g__Caulobacter),2),
nsmall=2),"%)", sep="")

h["Root.p__Proteobacteria.c__Alphaproteobacteria.o__Caulobacterales.
f__Caulobacteraceae.g__Phenylobacterium"] =
paste("g__Phenylobacterium (",
format(round(100*mean(y$Root.p__Proteobacteria.c__Alphaproteobacteri
a.o__Caulobacterales.f__Caulobacteraceae.g__Phenylobacterium),2),
nsmall=2),"%)", sep="")

h["Root.p__Proteobacteria.c__Alphaproteobacteria.o__Rhizobiales.f__B
radyrhizobiaceae.g__Bradyrhizobium"] = paste("g__Bradyrhizobium (",
format(round(100*mean(y$Root.p__Proteobacteria.c__Alphaproteobacteri
a.o__Rhizobiales.f__Bradyrhizobiaceae.g__Bradyrhizobium),2),
nsmall=2),"%)", sep="")

h["Root.p__Proteobacteria.c__Alphaproteobacteria.o__Rhizobiales.f__H
yphomicrobiaceae.g__Devosia"] = paste("g__Devosia (",
format(round(100*mean(y$Root.p__Proteobacteria.c__Alphaproteobacteri
a.o__Rhizobiales.f__Hyphomicrobiaceae.g__Devosia),2),
nsmall=2),"%)", sep="")

h["Root.p__Proteobacteria.c__Alphaproteobacteria.o__Rhodobacterales.
f__Rhodobacteraceae.g__Thioclava"] = paste("g__Thioclava (",
format(round(100*mean(y$Root.p__Proteobacteria.c__Alphaproteobacteri
a.o__Rhodobacterales.f__Rhodobacteraceae.g__Thioclava),2),
nsmall=2),"%)", sep="")

h["Root.p__Proteobacteria.c__Alphaproteobacteria.o__Sphingomonadales
.f__Sphingomonadaceae.Other"] = paste("f__Sphingomonadaceae (",
format(round(100*mean(y$Root.p__Proteobacteria.c__Alphaproteobacteri
a.o__Sphingomonadales.f__Sphingomonadaceae.Other),2),
nsmall=2),"%)", sep="")

h["Root.p__Proteobacteria.c__Alphaproteobacteria.o__Sphingomonadales
.Other.Other"] = paste("o__Sphingomonadales (",
format(round(100*mean(y$Root.p__Proteobacteria.c__Alphaproteobacteri
a.o__Sphingomonadales.Other.Other),2), nsmall=2),"%)", sep="")
```

```
h["Root.p__Proteobacteria.c__Alphaproteobacteria.Other.Other.Other"]
= paste("c__Alphaproteobacteria (",
format(round(100*mean(y$Root.p__Proteobacteria.c__Alphaproteobacteri
a.Other.Other.Other),2), nsmall=2),"%)", sep="")

h["Root.p__Proteobacteria.c__Betaproteobacteria.o__Burkholderiales.f
__Alcaligenaceae.g__Oligella"] = paste("g__Oligella (",
format(round(100*mean(y$Root.p__Proteobacteria.c__Betaproteobacteria
.o__Burkholderiales.f__Alcaligenaceae.g__Oligella),2),
nsmall=2),"%)", sep="")

h["Root.p__Proteobacteria.c__Betaproteobacteria.o__Burkholderiales.f
__Burkholderiaceae.g__Burkholderia"] = paste("g__Burkholderia (",
format(round(100*mean(y$Root.p__Proteobacteria.c__Betaproteobacteria
.o__Burkholderiales.f__Burkholderiaceae.g__Burkholderia),2),
nsmall=2),"%)", sep="")

h["Root.p__Proteobacteria.c__Betaproteobacteria.o__Burkholderiales.f
__Burkholderiaceae.g__Ralstonia"] = paste("g__Ralstonia (",
format(round(100*mean(y$Root.p__Proteobacteria.c__Betaproteobacteria
.o__Burkholderiales.f__Burkholderiaceae.g__Ralstonia),2),
nsmall=2),"%)", sep="")

h["Root.p__Proteobacteria.c__Betaproteobacteria.o__Burkholderiales.f
__Comamonadaceae.g__Acidovorax"] = paste("g__Acidovorax (",
format(round(100*mean(y$Root.p__Proteobacteria.c__Betaproteobacteria
.o__Burkholderiales.f__Comamonadaceae.g__Acidovorax),2),
nsmall=2),"%)", sep="")

h["Root.p__Proteobacteria.c__Betaproteobacteria.o__Burkholderiales.f
__Oxalobacteraceae.g__Janthinobacterium"] =
paste("g__Janthinobacterium (",
format(round(100*mean(y$Root.p__Proteobacteria.c__Betaproteobacteria
.o__Burkholderiales.f__Oxalobacteraceae.g__Janthinobacterium),2),
nsmall=2),"%)", sep="")

h["Root.p__Proteobacteria.c__Betaproteobacteria.o__Burkholderiales.f
__Oxalobacteraceae.Other"] = paste("f__Oxalobacteraceae (",
format(round(100*mean(y$Root.p__Proteobacteria.c__Betaproteobacteria
.o__Burkholderiales.f__Oxalobacteraceae.Other),2), nsmall=2),"%)",
sep="")

h["Root.p__Proteobacteria.c__Betaproteobacteria.o__Neisseriales.f__N
eisseriaceae.g__"] = paste("f__Neisseriaceae (",
format(round(100*mean(y$Root.p__Proteobacteria.c__Betaproteobacteria
.o__Neisseriales.f__Neisseriaceae.g__),2), nsmall=2),"%)", sep="")

h["Root.p__Proteobacteria.c__Betaproteobacteria.o__Neisseriales.f__N
eisseriaceae.g__Neisseria"] = paste("g__Neisseria (",
format(round(100*mean(y$Root.p__Proteobacteria.c__Betaproteobacteria
.o__Neisseriales.f__Neisseriaceae.g__Neisseria),2), nsmall=2),"%)",
sep="")

h["Root.p__Proteobacteria.c__Betaproteobacteria.o__Neisseriales.f__N
```

```
eisseriaceae.g__Simonsiella"] = paste("g__Simonsiella (",
format(round(100*mean(y$Root.p__Proteobacteria.c__Betaproteobacteria
.o__Neisseriales.f__Neisseriaceae.g__Simonsiella),2),
nsmall=2),"%)", sep="")

h["Root.p__Proteobacteria.c__Epsilonproteobacteria.o__Campylobactera
les.f__Campylobacteraceae.g__Campylobacter"] =
paste("g__Campylobacter (",
format(round(100*mean(y$Root.p__Proteobacteria.c__Epsilonproteobacte
ria.o__Campylobacterales.f__Campylobacteraceae.g__Campylobacter),2),
nsmall=2),"%)", sep="")

h["Root.p__Proteobacteria.c__Gammaproteobacteria.o__Aeromonadales.f_
_Aeromonadaceae.g__Aeromonas"] = paste("g__Aeromonas (",
format(round(100*mean(y$Root.p__Proteobacteria.c__Gammaproteobacteri
a.o__Aeromonadales.f__Aeromonadaceae.g__Aeromonas),2),
nsmall=2),"%)", sep="")

h["Root.p__Proteobacteria.c__Gammaproteobacteria.o__Alteromonadales.
f__Pseudoalteromonadaceae.g__Pseudoalteromonas"] =
paste("g__Pseudoalteromonas (",
format(round(100*mean(y$Root.p__Proteobacteria.c__Gammaproteobacteri
a.o__Alteromonadales.f__Pseudoalteromonadaceae.g__Pseudoalteromonas)
,2), nsmall=2),"%)", sep="")

h["Root.p__Proteobacteria.c__Gammaproteobacteria.o__Enterobacteriale
s.f__Enterobacteriaceae.g__Averyella"] = paste("g__Averyella (",
format(round(100*mean(y$Root.p__Proteobacteria.c__Gammaproteobacteri
a.o__Enterobacteriales.f__Enterobacteriaceae.g__Averyella),2),
nsmall=2),"%)", sep="")

h["Root.p__Proteobacteria.c__Gammaproteobacteria.o__Enterobacteriale
s.f__Enterobacteriaceae.g__Citrobacter"] = paste("g__Citrobacter (",
format(round(100*mean(y$Root.p__Proteobacteria.c__Gammaproteobacteri
a.o__Enterobacteriales.f__Enterobacteriaceae.g__Citrobacter),2),
nsmall=2),"%)", sep="")

h["Root.p__Proteobacteria.c__Gammaproteobacteria.o__Enterobacteriale
s.f__Enterobacteriaceae.g__Escherichia"] = paste("g__Escherichia (",
format(round(100*mean(y$Root.p__Proteobacteria.c__Gammaproteobacteri
a.o__Enterobacteriales.f__Enterobacteriaceae.g__Escherichia),2),
nsmall=2),"%)", sep="")

h["Root.p__Proteobacteria.c__Gammaproteobacteria.o__Enterobacteriale
s.f__Enterobacteriaceae.g__Klebsiella"] = paste("g__Klebsiella (",
format(round(100*mean(y$Root.p__Proteobacteria.c__Gammaproteobacteri
a.o__Enterobacteriales.f__Enterobacteriaceae.g__Klebsiella),2),
nsmall=2),"%)", sep="")

h["Root.p__Proteobacteria.c__Gammaproteobacteria.o__Enterobacteriale
s.f__Enterobacteriaceae.g__Leclercia"] = paste("g__Leclercia (",
format(round(100*mean(y$Root.p__Proteobacteria.c__Gammaproteobacteri
a.o__Enterobacteriales.f__Enterobacteriaceae.g__Leclercia),2),
nsmall=2),"%)", sep="")
```

```
h["Root.p__Proteobacteria.c__Gammaproteobacteria.o__Enterobacteriale
s.f__Enterobacteriaceae.g__Proteus"] = paste("g__Proteus (",
format(round(100*mean(y$Root.p__Proteobacteria.c__Gammaproteobacteri
a.o__Enterobacteriales.f__Enterobacteriaceae.g__Proteus),2),
nsmall=2),"%)", sep="")

h["Root.p__Proteobacteria.c__Gammaproteobacteria.o__Enterobacteriale
s.f__Enterobacteriaceae.g__Serratia"] = paste("g__Serratia (",
format(round(100*mean(y$Root.p__Proteobacteria.c__Gammaproteobacteri
a.o__Enterobacteriales.f__Enterobacteriaceae.g__Serratia),2),
nsmall=2),"%)", sep="")

h["Root.p__Proteobacteria.c__Gammaproteobacteria.o__Enterobacteriale
s.f__Enterobacteriaceae.Other"] = paste("f__Enterobacteriaceae (",
format(round(100*mean(y$Root.p__Proteobacteria.c__Gammaproteobacteri
a.o__Enterobacteriales.f__Enterobacteriaceae.Other),2),
nsmall=2),"%)", sep="")

h["Root.p__Proteobacteria.c__Gammaproteobacteria.o__Oceanospirillale
s.f__Halomonadaceae.g__Halomonas"] = paste("g__Halomonas (",
format(round(100*mean(y$Root.p__Proteobacteria.c__Gammaproteobacteri
a.o__Oceanospirillales.f__Halomonadaceae.g__Halomonas),2),
nsmall=2),"%)", sep="")

h["Root.p__Proteobacteria.c__Gammaproteobacteria.o__Pasteurellales.f
__Pasteurellaceae.g__Actinobacillus"] = paste("g__Actinobacillus (",
format(round(100*mean(y$Root.p__Proteobacteria.c__Gammaproteobacteri
a.o__Pasteurellales.f__Pasteurellaceae.g__Actinobacillus),2),
nsmall=2),"%)", sep="")

h["Root.p__Proteobacteria.c__Gammaproteobacteria.o__Pasteurellales.f
__Pasteurellaceae.g__Haemophilus"] = paste("g__Haemophilus (",
format(round(100*mean(y$Root.p__Proteobacteria.c__Gammaproteobacteri
a.o__Pasteurellales.f__Pasteurellaceae.g__Haemophilus),2),
nsmall=2),"%)", sep="")

h["Root.p__Proteobacteria.c__Gammaproteobacteria.o__Pasteurellales.f
__Pasteurellaceae.g__Pasteurella"] = paste("g__Pasteurella (",
format(round(100*mean(y$Root.p__Proteobacteria.c__Gammaproteobacteri
a.o__Pasteurellales.f__Pasteurellaceae.g__Pasteurella),2),
nsmall=2),"%)", sep="")

h["Root.p__Proteobacteria.c__Gammaproteobacteria.o__Pseudomonadales.
f__Moraxellaceae.g__"] = paste("f__Moraxellaceae (",
format(round(100*mean(y$Root.p__Proteobacteria.c__Gammaproteobacteri
a.o__Pseudomonadales.f__Moraxellaceae.g__),2), nsmall=2),"%)",
sep="")

h["Root.p__Proteobacteria.c__Gammaproteobacteria.o__Pseudomonadales.
f__Moraxellaceae.g__Acinetobacter"] = paste("g__Acinetobacter (",
format(round(100*mean(y$RRoot.p__Proteobacteria.c__Gammaproteobacter
ia.o__Pseudomonadales.f__Moraxellaceae.g__Acinetobacter),2),
nsmall=2),"%)", sep="")
```

```
h["Root.p__Proteobacteria.c__Gammaproteobacteria.o__Pseudomonadales.
f__Moraxellaceae.g__Moraxella"] = paste("g__Moraxella (",
format(round(100*mean(y$Root.p__Proteobacteria.c__Gammaproteobacteri
a.o__Pseudomonadales.f__Moraxellaceae.g__Moraxella),2),
nsmall=2),"%)", sep="")

h["Root.p__Proteobacteria.c__Gammaproteobacteria.o__Pseudomonadales.
f__Pseudomonadaceae.g__Pseudomonas"] = paste("g__Pseudomonas (",
format(round(100*mean(y$Root.p__Proteobacteria.c__Gammaproteobacteri
a.o__Pseudomonadales.f__Pseudomonadaceae.g__Pseudomonas),2),
nsmall=2),"%)", sep="")

h["Root.p__Proteobacteria.c__Gammaproteobacteria.o__Pseudomonadales.
f__Pseudomonadaceae.Other"] = paste("f__Pseudomonadaceae (",
format(round(100*mean(y$Root.p__Proteobacteria.c__Gammaproteobacteri
a.o__Pseudomonadales.f__Pseudomonadaceae.Other),2), nsmall=2),"%)",
sep="")

h["Root.p__Proteobacteria.c__Gammaproteobacteria.o__Vibrionales.f__V
ibrionaceae.g__Photobacterium"] = paste("g__Photobacterium (",
format(round(100*mean(y$Root.p__Proteobacteria.c__Gammaproteobacteri
a.o__Vibrionales.f__Vibrionaceae.g__Photobacterium),2),
nsmall=2),"%)", sep="")

h["Root.p__Proteobacteria.c__Gammaproteobacteria.o__Vibrionales.f__V
ibrionaceae.g__Vibrio"] = paste("g__Vibrio (",
format(round(100*mean(y$Root.p__Proteobacteria.c__Gammaproteobacteri
a.o__Vibrionales.f__Vibrionaceae.g__Vibrio),2), nsmall=2),"%)",
sep="")

h["Root.p__Proteobacteria.c__Gammaproteobacteria.o__Vibrionales.f__V
ibrionaceae.Other"] = paste("f__Vibrionaceae (",
format(round(100*mean(y$Root.p__Proteobacteria.c__Gammaproteobacteri
a.o__Vibrionales.f__Vibrionaceae.Other),2), nsmall=2),"%)", sep="")

h["Root.p__Proteobacteria.c__Gammaproteobacteria.o__Xanthomonadales.
f__Xanthomonadaceae.g__Stenotrophomonas"] =
paste("g__Stenotrophomonas (",
format(round(100*mean(y$Root.p__Proteobacteria.c__Gammaproteobacteri
a.o__Xanthomonadales.f__Xanthomonadaceae.g__Stenotrophomonas),2),
nsmall=2),"%)", sep="")

h["Root.p__Tenericutes.c__Erysipelotrichi.o__Erysipelotrichales.f__E
rysipelotrichaceae.g__"] = paste("f__Erysipelotrichaceae (",
format(round(100*mean(y$Root.p__Tenericutes.c__Erysipelotrichi.o__Er
ysipelotrichales.f__Erysipelotrichaceae.g__),2), nsmall=2),"%)",
sep="")

h["Root.p__Tenericutes.c__Erysipelotrichi.o__Erysipelotrichales.f__E
rysipelotrichaceae.g__Catenibacterium"] = paste("g__Catenibacterium
(",
format(round(100*mean(y$Root.p__Tenericutes.c__Erysipelotrichi.o__Er
ysipelotrichales.f__Erysipelotrichaceae.g__Catenibacterium),2),
```

```
nsmall=2),"%)", sep="")

h["Root.p__Tenericutes.c__Erysipelotrichi.o__Erysipelotrichales.f__E
rysipelotrichaceae.g__Clostridium"] = paste("g__Clostridium (",
format(round(100*mean(y$Root.p__Tenericutes.c__Erysipelotrichi.o__Er
ysipelotrichales.f__Erysipelotrichaceae.g__Clostridium),2),
nsmall=2),"%)", sep="")

h["Root.p__Tenericutes.c__Erysipelotrichi.o__Erysipelotrichales.f__E
rysipelotrichaceae.g__Coprobacillus"] = paste("g__Coprobacillus (",
format(round(100*mean(y$Root.p__Tenericutes.c__Erysipelotrichi.o__Er
ysipelotrichales.f__Erysipelotrichaceae.g__Coprobacillus),2),
nsmall=2),"%)", sep="")

h["Root.p__Tenericutes.c__Mollicutes.o__Mycoplasmatales.f__Mycoplasm
ataceae.g__Mycoplasma"] = paste("g__Mycoplasma (",
format(round(100*mean(y$Root.p__Tenericutes.c__Mollicutes.o__Mycopla
smatales.f__Mycoplasmataceae.g__Mycoplasma),2), nsmall=2),"%)",
sep="")

h["Root.p__Thermi.c__Deinococci.o__Deinococcales.f__Deinococcaceae.g
__Deinococcus"] = paste("g__Deinococcus (",
format(round(100*mean(y$Root.p__Thermi.c__Deinococci.o__Deinococcale
s.f__Deinococcaceae.g__Deinococcus),2), nsmall=2),"%)", sep="")

h["Root.p__Verrucomicrobia.c__Verrucomicrobiae.o__Verrucomicrobiales
.f__Verrucomicrobiaceae.g__Akkermansia"] = paste("g__Akkermansia (",
format(round(100*mean(y$Root.p__Verrucomicrobia.c__Verrucomicrobiae.
o__Verrucomicrobiales.f__Verrucomicrobiaceae.g__Akkermansia),2),
nsmall=2),"%)", sep="")
  h["Root.Other.Other.Other.Other.Other"] = paste("Root (",
format(round(100*mean(y$Root.Other.Other.Other.Other.Other),2),
nsmall=2),"%)", sep="")
  h["Taxa < 1%"] = paste("Taxa < 1% (",
format(round(100*mean(y$"Taxa < 1%"),2), nsmall=2),"%)", sep="")
  h["other"] = paste("Other (", format(round(100*mean(y$other),2),
nsmall=2),"%)", sep="")
  #Make a vector of colours to return
  labels <- character(0)
  for (i in 1:length(x)){
      col <- h[[x[i]]]
      #print(col)
      if (is.null(col)) {
        print(x[i])
      }
      labels <- c(labels, col)
  }
  return(labels)
}
```

Setup: define colours & labels for L7 Moraxella taxa (dada2_L7_Morax
function)

```{r colours, include=FALSE}
```

```
dada2_L7_Morax <- function(x) {
  #Setup hash
  h <- hash()
  #h["Moraxella"] = "#005b9e"

h["CACAGAGTTAGCCGGTGCTTATTCTGTGGGTAACGTCAGGGCTTATGGGTATTAACCATAAGCTT
TTCCTCCCCACTTAAAGTGCTTTACAACCAAAAGGCCTTCTTCACACACGCGGCATGGCTGGATCAGG
CTTTCGCCCATTGTCCAATATTCCCCA_L6_Moraxella"] = "#7398d8"

h["CACAGAGTTAGCCGGTGCTTATTCTGTGGGTAACGTCAGAACCTATGGGTGTTATCCATAAGCTT
TTCCTCCCCACTTAAAGTGCTTTACAACCATAAGGCCTTCTTCACACACGCGGCATGGCTGGATCAGG
CTTTCGCCCATTGTCCAATATTCCCCA_L6_Moraxella"] = "#162956"

h["CACAGAGTTAGCCGGTGCTTATTCTGTGGGTAACGTCAGGGCTTATGGGTATTAACCACAAGCTT
TTCCTCCCCACTTAAAGTGCTTTACAACCAAAAGGCCTTCTTCACACACGCGGCATGGCTGGATCAGG
CTTTCGCCCATTGTCCAATATTCCCCA_L6_Moraxella"] = "#3fa2c8"

h["CACAGAGTTAGCCGGTGCTTATTCTGTGGGTAACGTCAGAACCTATGGGTATTATCCATAAGCTT
TTCCTCCCCACTTAAAGTGCTTTACAACCATAAGGCCTTCTTCACACACGCGGCATGGCTGGATCAGG
CTTTCGCCCATTGTCCAATATTCCCCA_L6_Moraxella"] = "#3f7cde"

h["CACAGAGTTAGCCGGTGCTTATTCTGTGGGTAACGTCAGGGCTTATGGGTATTAACCATAAACTT
TTCCTCCCCACTTAAAGTGCTTTACAACCAAAAGGCCTTCTTCACACACGCGGCATGGCTGGATCAGG
CTTTCGCCCATTGTCCAATATTCCCCA_L6_Moraxella"] = "#29477d"

h["CACAGAGTTAGCCGGTGCTTATTCTGTGGGTAACGTCAGGGCTTGTGGGTATTAACCACAAGCTT
TTCCTCCCCACTTAAAGTGCTTTACAACCAAAAGGCCTTCTTCACACACGCGGCATGGCTGGATCAGG
CTTTCGCCCATTGTCCAATATTCCCCA_L6_Moraxella"] = "#456ba4"
  h["Taxa < 1%"] = "#808080"
  h["other"] = "#808080"
  h["NA"] = "#181818"
  #Make a vector of colours to return
  colours <- character(0)
  for (i in 1:length(x)){
    if (x[i] == "Taxa < 1%") {
      col <- h[[x[i]]]
    } else {
      x.sub  <- str_split(x[i], "_L6_")[[1]][2]
      if (!str_detect(x.sub, "Moraxella")) {
        col <- "#808080" #181818"
      } else {
        col <- h[[x[i]]]
      }
    }
      if (is.null(col)) {
        print(x[i])
      }
      colours <- c(colours, col)
  }
  return(colours)
}

#myLabels_L6 function
myLabels_L6 <- function(x,y) {
```

```
  #Setup hash
  h <- hash()

h["Root.p__Actinobacteria.c__Actinobacteria.o__Actinomycetales.f__Ac
tinomycetaceae.g__Actinobaculum"] = paste("g__Actinobaculum (",
format(round(100*mean(y$Root.p__Actinobacteria.c__Actinobacteria.o__
Actinomycetales.f__Actinomycetaceae.g__Actinobaculum),2),
nsmall=2),"%)", sep="")

h["Root.p__Actinobacteria.c__Actinobacteria.o__Actinomycetales.f__Ac
tinomycetaceae.g__Actinomyces"] = paste("g__Actinomyces (",
format(round(100*mean(y$Root.p__Actinobacteria.c__Actinobacteria.o__
Actinomycetales.f__Actinomycetaceae.g__Actinomyces),2),
nsmall=2),"%)", sep="")

h["Root.p__Actinobacteria.c__Actinobacteria.o__Coriobacteriales.f__C
oriobacteriaceae.g__Adlercreutzia"] = paste("g__Adlercreutzia (",
format(round(100*mean(y$Root.p__Actinobacteria.c__Actinobacteria.o__
Actinomycetales.f__Actinomycetaceae.g__Adlercreutzia),2),
nsmall=2),"%)", sep="")

h["Root.p__Actinobacteria.c__Actinobacteria.o__Actinomycetales.f__Ac
tinomycetaceae.g__Arcanobacterium"] = paste("g__Arcanobacterium (",
format(round(100*mean(y$Root.p__Actinobacteria.c__Actinobacteria.o__
Actinomycetales.f__Actinomycetaceae.g__Arcanobacterium),2),
nsmall=2),"%)", sep="")

h["Root.p__Actinobacteria.c__Actinobacteria.o__Actinomycetales.f__Br
evibacteriaceae.g__Brevibacterium"] = paste("g__Brevibacterium (",
format(round(100*mean(y$Root.p__Actinobacteria.c__Actinobacteria.o__
Actinomycetales.f__Brevibacteriaceae.g__Brevibacterium),2),
nsmall=2),"%)", sep="")

h["Root.p__Actinobacteria.c__Actinobacteria.o__Actinomycetales.f__Co
rynebacteriaceae.g__"] = paste("f__Corynebacteriaceae (",
format(round(100*mean(y$Root.p__Actinobacteria.c__Actinobacteria.o__
Actinomycetales.f__Corynebacteriaceae.g__),2), nsmall=2),"%)",
sep="")

h["Root.p__Actinobacteria.c__Actinobacteria.o__Actinomycetales.f__Co
rynebacteriaceae.g__Corynebacterium"] = paste("g__Corynebacterium
(",
format(round(100*mean(y$Root.p__Actinobacteria.c__Actinobacteria.o__
Actinomycetales.f__Corynebacteriaceae.g__Corynebacterium),2),
nsmall=2),"%)", sep="")

h["Root.p__Actinobacteria.c__Actinobacteria.o__Actinomycetales.f__In
trasporangiaceae.g__"] = paste("f__Intrasporangiaceae (",
format(round(100*mean(y$Root.p__Actinobacteria.c__Actinobacteria.o__
Actinomycetales.f__Intrasporangiaceae.g__),2), nsmall=2),"%)",
sep="")

h["Root.p__Actinobacteria.c__Actinobacteria.o__Actinomycetales.f__Mi
crococcaceae.g__Rothia"] = paste("g__Rothia (",
```

```
format(round(100*mean(y$Root.p__Actinobacteria.c__Actinobacteria.o__
Actinomycetales.f__Micrococcaceae.g__Rothia),2), nsmall=2),"%)",
sep="")

h["Root.p__Actinobacteria.c__Actinobacteria.o__Actinomycetales.f__Mi
cromonosporaceae.Other"] = paste("f__Micromonosporaceae (",
format(round(100*mean(y$Root.p__Actinobacteria.c__Actinobacteria.o__
Actinomycetales.f__Micromonosporaceae.Other),2), nsmall=2),"%)",
sep="")

h["Root.p__Actinobacteria.c__Actinobacteria.o__Actinomycetales.f__Pr
omicromonosporaceae.g__Cellulosimicrobium"] =
paste("g__Cellulosimicrobium (",
format(round(100*mean(y$Root.p__Actinobacteria.c__Actinobacteria.o__
Actinomycetales.f__Promicromonosporaceae.g__Cellulosimicrobium),2),
nsmall=2),"%)", sep="")

h["Root.p__Actinobacteria.c__Actinobacteria.o__Actinomycetales.f__Pr
opionibacteriaceae.g__Propionibacterium"] =
paste("g__Propionibacterium (",
format(round(100*mean(y$Root.p__Actinobacteria.c__Actinobacteria.o__
Actinomycetales.f__Propionibacteriaceae.g__Propionibacterium),2),
nsmall=2),"%)", sep="")

h["Root.p__Actinobacteria.c__Actinobacteria.o__Actinomycetales.f__Pr
opionibacteriaceae.Other"] = paste("f__Propionibacteriaceae (",
format(round(100*mean(y$Root.p__Actinobacteria.c__Actinobacteria.o__
Actinomycetales.f__Propionibacteriaceae.Other),2), nsmall=2),"%)",
sep="")

h["Root.p__Actinobacteria.c__Actinobacteria.o__Actinomycetales.Other
.Other"] = paste("o__Actinomycetales (",
format(round(100*mean(y$Root.p__Actinobacteria.c__Actinobacteria.o__
Actinomycetales.Other.Other),2), nsmall=2),"%)", sep="")

h["Root.p__Actinobacteria.c__Actinobacteria.o__Bifidobacteriales.f__
Bifidobacteriaceae.g__Bifidobacterium"] = paste("g__Bifidobacterium
(",
format(round(100*mean(y$Root.p__Actinobacteria.c__Actinobacteria.o__
Bifidobacteriales.f__Bifidobacteriaceae.g__Bifidobacterium),2),
nsmall=2),"%)", sep="")

h["Root.p__Actinobacteria.c__Actinobacteria.o__Bifidobacteriales.f__
Bifidobacteriaceae.g__Gardnerella"] = paste("g__Gardnerella (",
format(round(100*mean(y$Root.p__Actinobacteria.c__Actinobacteria.o__
Bifidobacteriales.f__Bifidobacteriaceae.g__Gardnerella),2),
nsmall=2),"%)", sep="")

h["Root.p__Actinobacteria.c__Actinobacteria.o__Bifidobacteriales.f__
Bifidobacteriaceae.g__Scardovia"] = paste("g__Scardovia (",
format(round(100*mean(y$Root.p__Actinobacteria.c__Actinobacteria.o__
Bifidobacteriales.f__Bifidobacteriaceae.g__Scardovia),2),
nsmall=2),"%)", sep="")
```

```
h["Root.p__Actinobacteria.c__Actinobacteria.o__Coriobacteriales.f__C
oriobacteriaceae.g__Collinsella"] = paste("g__Collinsella (",
format(round(100*mean(y$Root.p__Actinobacteria.c__Actinobacteria.o__
Coriobacteriales.f__Coriobacteriaceae.g__Collinsella),2),
nsmall=2),"%)", sep="")
  h["Root.p__Bacteroidetes.c__Bacteroidia.o__Bacteroidales.f__.g__"]
= paste("o__Bacteroidales (",
format(round(100*mean(y$Root.p__Bacteroidetes.c__Bacteroidia.o__Bact
eroidales.f__.g__),2), nsmall=2),"%)", sep="")

h["Root.p__Bacteroidetes.c__Bacteroidia.o__Bacteroidales.f__Bacteroi
daceae.g__Bacteroides"] = paste("g__Bacteroides (",
format(round(100*mean(y$Root.p__Bacteroidetes.c__Bacteroidia.o__Bact
eroidales.f__Bacteroidaceae.g__Bacteroides),2), nsmall=2),"%)",
sep="")

h["Root.p__Bacteroidetes.c__Bacteroidia.o__Bacteroidales.f__Porphyro
monadaceae.g__"] = paste("f__Porphyromonadaceae (",
format(round(100*mean(y$Root.p__Bacteroidetes.c__Bacteroidia.o__Bact
eroidales.f__Porphyromonadaceae.g__),2), nsmall=2),"%)", sep="")

h["Root.p__Bacteroidetes.c__Bacteroidia.o__Bacteroidales.f__Porphyro
monadaceae.g__Parabacteroides"] = paste("g__Parabacteroides (",
format(round(100*mean(y$Root.p__Bacteroidetes.c__Bacteroidia.o__Bact
eroidales.f__Porphyromonadaceae.g__Parabacteroides),2),
nsmall=2),"%)", sep="")

h["Root.p__Bacteroidetes.c__Bacteroidia.o__Bacteroidales.f__Porphyro
monadaceae.g__Porphyromonas"] = paste("g__Porphyromonas (",
format(round(100*mean(y$Root.p__Bacteroidetes.c__Bacteroidia.o__Bact
eroidales.f__Porphyromonadaceae.g__Porphyromonas),2),
nsmall=2),"%)", sep="")

h["Root.p__Bacteroidetes.c__Bacteroidia.o__Bacteroidales.f__Prevotel
laceae.g__Prevotella"] = paste("g__Prevotella (",
format(round(100*mean(y$Root.p__Bacteroidetes.c__Bacteroidia.o__Bact
eroidales.f__Prevotellaceae.g__Prevotella),2), nsmall=2),"%)",
sep="")

h["Root.p__Bacteroidetes.c__Bacteroidia.o__Bacteroidales.f__Rikenell
aceae.g__Alistipes"] = paste("g__Alistipes (",
format(round(100*mean(y$Root.p__Bacteroidetes.c__Bacteroidia.o__Bact
eroidales.f__Rikenellaceae.g__Alistipes),2), nsmall=2),"%)", sep="")

h["Root.p__Bacteroidetes.c__Flavobacteria.o__Flavobacteriales.f__Fla
vobacteriaceae.g__Capnocytophaga"] = paste("g__Capnocytophaga (",
format(round(100*mean(y$Root.p__Bacteroidetes.c__Flavobacteria.o__Fl
avobacteriales.f__Flavobacteriaceae.g__Capnocytophaga),2),
nsmall=2),"%)", sep="")

h["Root.p__Bacteroidetes.c__Flavobacteria.o__Flavobacteriales.f__Fla
vobacteriaceae.g__Chryseobacterium"] = paste("g__Chryseobacterium
(",
format(round(100*mean(y$Root.p__Bacteroidetes.c__Flavobacteria.o__Fl
```

```
avobacteriales.f__Flavobacteriaceae.g__Chryseobacterium),2),
nsmall=2),"%)", sep="")

h["Root.p__Bacteroidetes.c__Sphingobacteria.o__Sphingobacteriales.f_
_Flexibacteraceae.g__"] = paste("f__Flexibacteraceae (",
format(round(100*mean(y$Root.p__Bacteroidetes.c__Sphingobacteria.o__
Sphingobacteriales.f__Flexibacteraceae.g__),2), nsmall=2),"%)",
sep="")

h["Root.p__Bacteroidetes.c__Sphingobacteria.o__Sphingobacteriales.f_
_Sphingobacteriaceae.g__Sphingobacterium"] =
paste("g__Sphingobacterium (",
format(round(100*mean(y$Root.p__Bacteroidetes.c__Sphingobacteria.o__
Sphingobacteriales.f__Sphingobacteriaceae.g__Sphingobacterium),2),
nsmall=2),"%)", sep="")
  h["Root.p__Chloroflexi.c__SOGA31.o__.f__.g__"] = paste("c__SOGA31
(",
format(round(100*mean(y$Root.p__Chloroflexi.c__SOGA31.o__.f__.g__),2
), nsmall=2),"%)", sep="")
  h["Root.p__Crenarchaeota.Other.Other.Other.Other"] =
paste("p__Crenarchaeota (",
format(round(100*mean(y$Root.p__Crenarchaeota.Other.Other.Other.Othe
r),2), nsmall=2),"%)", sep="")
  h["Root.p__Cyanobacteria.c__Chloroplast.o__Streptophyta.f__.g__"]
= paste("o__Streptophyta (",
format(round(100*mean(y$Root.p__Cyanobacteria.c__Chloroplast.o__Stre
ptophyta.f__.g__),2), nsmall=2),"%)", sep="")

h["Root.p__Cyanobacteria.c__Nostocophycideae.o__Nostocales.f__Nostoc
aceae.Other"] = paste("f__Nostocaceae (",
format(round(100*mean(y$Root.p__Cyanobacteria.c__Nostocophycideae.o_
_Nostocales.f__Nostocaceae.Other),2), nsmall=2),"%)", sep="")

h["Root.p__Cyanobacteria.c__Synechococcophycideae.o__Pseudanabaenale
s.f__Pseudanabaenaceae.g__Halomicronema"] = paste("g__Halomicronema
(",
format(round(100*mean(y$Root.p__Cyanobacteria.c__Synechococcophycide
ae.o__Pseudanabaenales.f__Pseudanabaenaceae.g__Halomicronema),2),
nsmall=2),"%)", sep="")

h["Root.p__Firmicutes.c__Bacilli.o__Bacillales.f__Bacillaceae.g__Ano
xybacillus"] = paste("g__Anoxybacillus (",
format(round(100*mean(y$Root.p__Firmicutes.c__Bacilli.o__Bacillales.
f__Bacillaceae.g__Anoxybacillus),2), nsmall=2),"%)", sep="")

h["Root.p__Firmicutes.c__Bacilli.o__Bacillales.f__Bacillaceae.g__Bac
illus"] = paste("g__Bacillus (",
format(round(100*mean(y$Root.p__Firmicutes.c__Bacilli.o__Bacillales.
f__Bacillaceae.g__Bacillus),2), nsmall=2),"%)", sep="")

h["Root.p__Firmicutes.c__Bacilli.o__Bacillales.f__Paenibacillaceae.g
__Paenibacillus"] = paste("g__Paenibacillus (",
format(round(100*mean(y$Root.p__Firmicutes.c__Bacilli.o__Bacillales.
f__Paenibacillaceae.g__Paenibacillus),2), nsmall=2),"%)", sep="")
```

```
h["Root.p__Firmicutes.c__Bacilli.o__Bacillales.f__Staphylococcaceae.
g__Staphylococcus"] = paste("g__Staphylococcus (",
format(round(100*mean(y$Root.p__Firmicutes.c__Bacilli.o__Bacillales.
f__Staphylococcaceae.g__Staphylococcus),2), nsmall=2),"%)", sep="")

h["Root.p__Firmicutes.c__Bacilli.o__Gemellales.f__Gemellaceae.g__Gem
ella"] = paste("g__Gemella (",
format(round(100*mean(y$Root.p__Firmicutes.c__Bacilli.o__Gemellales.
f__Gemellaceae.g__Gemella),2), nsmall=2),"%)", sep="")

h["Root.p__Firmicutes.c__Bacilli.o__Lactobacillales.f__Aerococcaceae
.g__Aerococcus"] = paste("g__Aerococcus (",
format(round(100*mean(y$Root.p__Firmicutes.c__Bacilli.o__Lactobacill
ales.f__Aerococcaceae.g__Aerococcus),2), nsmall=2),"%)", sep="")

h["Root.p__Firmicutes.c__Bacilli.o__Lactobacillales.f__Aerococcaceae
.g__Facklamia"] = paste("g__Facklamia (",
format(round(100*mean(y$Root.p__Firmicutes.c__Bacilli.o__Lactobacill
ales.f__Aerococcaceae.g__Facklamia),2), nsmall=2),"%)", sep="")

h["Root.p__Firmicutes.c__Bacilli.o__Lactobacillales.f__Carnobacteria
ceae.g__"] = paste("f__Carnobacteriaceae (",
format(round(100*mean(y$Root.p__Firmicutes.c__Bacilli.o__Lactobacill
ales.f__Carnobacteriaceae.g__),2), nsmall=2),"%)", sep="")

h["Root.p__Firmicutes.c__Bacilli.o__Lactobacillales.f__Carnobacteria
ceae.g__Granulicatella"] = paste("g__Granulicatella (",
format(round(100*mean(y$Root.p__Firmicutes.c__Bacilli.o__Lactobacill
ales.f__Carnobacteriaceae.g__Granulicatella),2), nsmall=2),"%)",
sep="")

h["Root.p__Firmicutes.c__Bacilli.o__Lactobacillales.f__Enterococcace
ae.g__Enterococcus"] = paste("g__Enterococcus (",
format(round(100*mean(y$Root.p__Firmicutes.c__Bacilli.o__Lactobacill
ales.f__Enterococcaceae.g__Enterococcus),2), nsmall=2),"%)", sep="")

h["Root.p__Firmicutes.c__Bacilli.o__Lactobacillales.f__Lactobacillac
eae.g__Lactobacillus"] = paste("g__Lactobacillus (",
format(round(100*mean(y$Root.p__Firmicutes.c__Bacilli.o__Lactobacill
ales.f__Lactobacillaceae.g__Lactobacillus),2), nsmall=2),"%)",
sep="")

h["Root.p__Firmicutes.c__Bacilli.o__Lactobacillales.f__Leuconostocac
eae.g__Weissella"] = paste("g__Weissella (",
format(round(100*mean(y$Root.p__Firmicutes.c__Bacilli.o__Lactobacill
ales.f__Leuconostocaceae.g__Weissella),2), nsmall=2),"%)", sep="")

h["Root.p__Firmicutes.c__Bacilli.o__Lactobacillales.f__Streptococcac
eae.g__Lactococcus"] = paste("g__Lactococcus (",
format(round(100*mean(y$Root.p__Firmicutes.c__Bacilli.o__Lactobacill
ales.f__Streptococcaceae.g__Lactococcus),2), nsmall=2),"%)", sep="")

h["Root.p__Firmicutes.c__Bacilli.o__Lactobacillales.f__Streptococcac
```

```
eae.g__Streptococcus"] = paste("g__Streptococcus (",
format(round(100*mean(y$Root.p__Firmicutes.c__Bacilli.o__Lactobacill
ales.f__Streptococcaceae.g__Streptococcus),2), nsmall=2),"%)",
sep="")

h["Root.p__Firmicutes.c__Bacilli.o__Lactobacillales.f__Streptococcac
eae.Other"] = paste("f__Streptococcaceae (",
format(round(100*mean(y$Root.p__Firmicutes.c__Bacilli.o__Lactobacill
ales.f__Streptococcaceae.Other),2), nsmall=2),"%)", sep="")

h["Root.p__Firmicutes.c__Bacilli.o__Turicibacterales.f__Turicibacter
aceae.g__"] = paste("f__Turicibacteraceae (",
format(round(100*mean(y$Root.p__Firmicutes.c__Bacilli.o__Turicibacte
rales.f__Turicibacteraceae.g__),2), nsmall=2),"%)", sep="")

h["Root.p__Firmicutes.c__Bacilli.o__Turicibacterales.f__Turicibacter
aceae.g__Turicibacter"] = paste("g__Turicibacter (",
format(round(100*mean(y$Root.p__Firmicutes.c__Bacilli.o__Turicibacte
rales.f__Turicibacteraceae.g__Turicibacter),2), nsmall=2),"%)",
sep="")
  h["Root.p__Firmicutes.c__Clostridia.o__Clostridiales.f__.g__"] =
paste("o__Clostridiales (",
format(round(100*mean(y$Root.p__Firmicutes.c__Clostridia.o__Clostrid
iales.f__.g__),2), nsmall=2),"%)", sep="")

h["Root.p__Firmicutes.c__Clostridia.o__Clostridiales.f__Catabacteria
ceae.g__"] = paste("f__Catabacteriaceae (",
format(round(100*mean(y$Root.p__Firmicutes.c__Clostridia.o__Clostrid
iales.f__Catabacteriaceae.g__),2), nsmall=2),"%)", sep="")

h["Root.p__Firmicutes.c__Clostridia.o__Clostridiales.f__Clostridiace
ae.g__Clostridium"] = paste("g__Clostridium (",
format(round(100*mean(y$Root.p__Firmicutes.c__Clostridia.o__Clostrid
iales.f__Clostridiaceae.g__Clostridium),2), nsmall=2),"%)", sep="")

h["Root.p__Firmicutes.c__Clostridia.o__Clostridiales.f__Clostridiace
ae.Other"] = paste("f__Clostridiaceae (",
format(round(100*mean(y$Root.p__Firmicutes.c__Clostridia.o__Clostrid
iales.f__Clostridiaceae.Other),2), nsmall=2),"%)", sep="")

h["Root.p__Firmicutes.c__Clostridia.o__Clostridiales.f__Clostridiale
sFamilyXI.IncertaeSedis.g__"] =
paste("f__ClostridialesFamilyXI.IncertaeSedis (",
format(round(100*mean(y$Root.p__Firmicutes.c__Clostridia.o__Clostrid
iales.f__ClostridialesFamilyXI.IncertaeSedis.g__),2),
nsmall=2),"%)", sep="")

h["Root.p__Firmicutes.c__Clostridia.o__Clostridiales.f__Clostridiale
sFamilyXI.IncertaeSedis.g__Anaerococcus"] = paste("g__Anaerococcus
(",
format(round(100*mean(y$Root.p__Firmicutes.c__Clostridia.o__Clostrid
iales.f__ClostridialesFamilyXI.IncertaeSedis.g__Anaerococcus),2),
nsmall=2),"%)", sep="")
```

```
h["Root.p__Firmicutes.c__Clostridia.o__Clostridiales.f__Clostridiale
sFamilyXI.IncertaeSedis.g__Finegoldia"] = paste("g__Finegoldia (",
format(round(100*mean(y$Root.p__Firmicutes.c__Clostridia.o__Clostrid
iales.f__ClostridialesFamilyXI.IncertaeSedis.g__Finegoldia),2),
nsmall=2),"%)", sep="")

h["Root.p__Firmicutes.c__Clostridia.o__Clostridiales.f__Clostridiale
sFamilyXI.IncertaeSedis.g__Peptoniphilus"] = paste("g__Peptoniphilus
(",
format(round(100*mean(y$Root.p__Firmicutes.c__Clostridia.o__Clostrid
iales.f__ClostridialesFamilyXI.IncertaeSedis.g__Peptoniphilus),2),
nsmall=2),"%)", sep="")

h["Root.p__Firmicutes.c__Clostridia.o__Clostridiales.f__Lachnospirac
eae.g__"] = paste("f__Lachnospiraceae (",
format(round(100*mean(y$Root.p__Firmicutes.c__Clostridia.o__Clostrid
iales.f__Lachnospiraceae.g__),2), nsmall=2),"%)", sep="")

h["Root.p__Firmicutes.c__Clostridia.o__Clostridiales.f__Lachnospirac
eae.g__Blautia"] = paste("g__Blautia (",
format(round(100*mean(y$Root.p__Firmicutes.c__Clostridia.o__Clostrid
iales.f__Lachnospiraceae.g__Blautia),2), nsmall=2),"%)", sep="")

h["Root.p__Firmicutes.c__Clostridia.o__Clostridiales.f__Lachnospirac
eae.g__Catonella"] = paste("g__Catonella (",
format(round(100*mean(y$Root.p__Firmicutes.c__Clostridia.o__Clostrid
iales.f__Lachnospiraceae.g__Catonella),2), nsmall=2),"%)", sep="")

h["Root.p__Firmicutes.c__Clostridia.o__Clostridiales.f__Lachnospirac
eae.g__Clostridium"] = paste("g__Clostridium (",
format(round(100*mean(y$Root.p__Firmicutes.c__Clostridia.o__Clostrid
iales.f__Lachnospiraceae.g__Clostridium),2), nsmall=2),"%)", sep="")

h["Root.p__Firmicutes.c__Clostridia.o__Clostridiales.f__Lachnospirac
eae.g__Lachnobacterium"] = paste("g__Lachnobacterium (",
format(round(100*mean(y$Root.p__Firmicutes.c__Clostridia.o__Clostrid
iales.f__Lachnospiraceae.g__Lachnobacterium),2), nsmall=2),"%)",
sep="")

h["Root.p__Firmicutes.c__Clostridia.o__Clostridiales.f__Lachnospirac
eae.g__Lachnospira"] = paste("g__Lachnospira (",
format(round(100*mean(y$Root.p__Firmicutes.c__Clostridia.o__Clostrid
iales.f__Lachnospiraceae.g__Lachnospira),2), nsmall=2),"%)", sep="")

h["Root.p__Firmicutes.c__Clostridia.o__Clostridiales.f__Lachnospirac
eae.g__Pseudobutyrivibrio"] = paste("g__Pseudobutyrivibrio (",
format(round(100*mean(y$Root.p__Firmicutes.c__Clostridia.o__Clostrid
iales.f__Lachnospiraceae.g__Pseudobutyrivibrio),2), nsmall=2),"%)",
sep="")

h["Root.p__Firmicutes.c__Clostridia.o__Clostridiales.f__Lachnospirac
eae.g__Ruminococcus"] = paste("g__Ruminococcus (",
format(round(100*mean(y$Root.p__Firmicutes.c__Clostridia.o__Clostrid
iales.f__Lachnospiraceae.g__Ruminococcus),2), nsmall=2),"%)",
```

```
sep="")

h["Root.p__Firmicutes.c__Clostridia.o__Clostridiales.f__Lachnospirac
eae.g__Shuttleworthia"] = paste("g__Shuttleworthia (",
format(round(100*mean(y$Root.p__Firmicutes.c__Clostridia.o__Clostrid
iales.f__Lachnospiraceae.g__Shuttleworthia),2), nsmall=2),"%)",
sep="")

h["Root.p__Firmicutes.c__Clostridia.o__Clostridiales.f__Lachnospirac
eae.Other"] = paste("f__Lachnospiraceae (",
format(round(100*mean(y$Root.p__Firmicutes.c__Clostridia.o__Clostrid
iales.f__Lachnospiraceae.Other),2), nsmall=2),"%)", sep="")

h["Root.p__Firmicutes.c__Clostridia.o__Clostridiales.f__Ruminococcac
eae.g__"] = paste("f__Ruminococcaceae (",
format(round(100*mean(y$Root.p__Firmicutes.c__Clostridia.o__Clostrid
iales.f__Ruminococcaceae.g__),2), nsmall=2),"%)", sep="")

h["Root.p__Firmicutes.c__Clostridia.o__Clostridiales.f__Ruminococcac
eae.Other"] = paste("f__Ruminococcaceae (",
format(round(100*mean(y$Root.p__Firmicutes.c__Clostridia.o__Clostrid
iales.f__Ruminococcaceae.Other),2), nsmall=2),"%)", sep="")

h["Root.p__Firmicutes.c__Clostridia.o__Clostridiales.f__Ruminococcac
eae.g__Bacteroides"] = paste("g__Bacteroides (",
format(round(100*mean(y$Root.p__Firmicutes.c__Clostridia.o__Clostrid
iales.f__Ruminococcaceae.g__Bacteroides),2), nsmall=2),"%)", sep="")

h["Root.p__Firmicutes.c__Clostridia.o__Clostridiales.f__Ruminococcac
eae.g__Clostridium"] = paste("g__Clostridium (",
format(round(100*mean(y$Root.p__Firmicutes.c__Clostridia.o__Clostrid
iales.f__Ruminococcaceae.g__Clostridium),2), nsmall=2),"%)", sep="")

h["Root.p__Firmicutes.c__Clostridia.o__Clostridiales.f__Ruminococcac
eae.g__Eubacterium"] = paste("g__Eubacterium (",
format(round(100*mean(y$Root.p__Firmicutes.c__Clostridia.o__Clostrid
iales.f__Ruminococcaceae.g__Eubacterium),2), nsmall=2),"%)", sep="")

h["Root.p__Firmicutes.c__Clostridia.o__Clostridiales.f__Ruminococcac
eae.g__Faecalibacterium"] = paste("g__Faecalibacterium (",
format(round(100*mean(y$Root.p__Firmicutes.c__Clostridia.o__Clostrid
iales.f__Ruminococcaceae.g__Faecalibacterium),2), nsmall=2),"%)",
sep="")

h["Root.p__Firmicutes.c__Clostridia.o__Clostridiales.f__Ruminococcac
eae.g__Oscillospira"] = paste("g__Oscillospira (",
format(round(100*mean(y$Root.p__Firmicutes.c__Clostridia.o__Clostrid
iales.f__Ruminococcaceae.g__Oscillospira),2), nsmall=2),"%)",
sep="")

h["Root.p__Firmicutes.c__Clostridia.o__Clostridiales.f__Ruminococcac
eae.g__Ruminococcus"] = paste("g__Ruminococcus (",
format(round(100*mean(y$Root.p__Firmicutes.c__Clostridia.o__Clostrid
iales.f__Ruminococcaceae.g__Ruminococcus),2), nsmall=2),"%)",
```

```
sep="")

h["Root.p__Firmicutes.c__Clostridia.o__Clostridiales.f__Ruminococcac
eae.g__Subdoligranulum"] = paste("g__Subdoligranulum (",
format(round(100*mean(y$Root.p__Firmicutes.c__Clostridia.o__Clostrid
iales.f__Ruminococcaceae.g__Subdoligranulum),2), nsmall=2),"%)",
sep="")

h["Root.p__Firmicutes.c__Clostridia.o__Clostridiales.f__Veillonellac
eae.g__Dialister"] = paste("g__Dialister (",
format(round(100*mean(y$Root.p__Firmicutes.c__Clostridia.o__Clostrid
iales.f__Veillonellaceae.g__Dialister),2), nsmall=2),"%)", sep="")

h["Root.p__Firmicutes.c__Clostridia.o__Clostridiales.f__Veillonellac
eae.g__Mitsuokella"] = paste("g__Mitsuokella (",
format(round(100*mean(y$Root.p__Firmicutes.c__Clostridia.o__Clostrid
iales.f__Veillonellaceae.g__Mitsuokella),2), nsmall=2),"%)", sep="")

h["Root.p__Firmicutes.c__Clostridia.o__Clostridiales.f__Veillonellac
eae.g__Veillonella"] = paste("g__Veillonella (",
format(round(100*mean(y$Root.p__Firmicutes.c__Clostridia.o__Clostrid
iales.f__Veillonellaceae.g__Veillonella),2), nsmall=2),"%)", sep="")
  h["Root.p__Firmicutes.c__Clostridia.o__Clostridiales.Other.Other"]
= paste("o__Clostridiales (",
format(round(100*mean(y$Root.p__Firmicutes.c__Clostridia.o__Clostrid
iales.Other.Other),2), nsmall=2),"%)", sep="")
  h["Root.p__Firmicutes.c__Clostridia.Other.Other.Other"] =
paste("c__Clostridia (",
format(round(100*mean(y$Root.p__Firmicutes.c__Clostridia.Other.Other
.Other),2), nsmall=2),"%)", sep="")

h["Root.p__Fusobacteria.c__Fusobacteria.o__Fusobacteriales.f__Fusoba
cteriaceae.g__Fusobacterium"] = paste("g__Fusobacterium (",
format(round(100*mean(y$Root.p__Fusobacteria.c__Fusobacteria.o__Fuso
bacteriales.f__Fusobacteriaceae.g__Fusobacterium),2),
nsmall=2),"%)", sep="")

h["Root.p__Fusobacteria.c__Fusobacteria.o__Fusobacteriales.f__Fusoba
cteriaceae.g__Leptotrichia"] = paste("g__Leptotrichia (",
format(round(100*mean(y$Root.p__Fusobacteria.c__Fusobacteria.o__Fuso
bacteriales.f__Fusobacteriaceae.g__Leptotrichia),2), nsmall=2),"%)",
sep="")

h["Root.p__Fusobacteria.c__Fusobacteria.o__Fusobacteriales.f__Fusoba
cteriaceae.g__Sneathia"] = paste("g__Sneathia (",
format(round(100*mean(y$Root.p__Fusobacteria.c__Fusobacteria.o__Fuso
bacteriales.f__Fusobacteriaceae.g__Sneathia),2), nsmall=2),"%)",
sep="")

h["Root.p__Fusobacteria.c__Fusobacteria.o__Fusobacteriales.f__Fusoba
cteriaceae.Other"] = paste("f__Fusobacteriaceae (",
format(round(100*mean(y$Root.p__Fusobacteria.c__Fusobacteria.o__Fuso
bacteriales.f__Fusobacteriaceae.Other),2), nsmall=2),"%)", sep="")
```

```
h["Root.p__Proteobacteria.c__Alphaproteobacteria.o__Caulobacterales.
f__Caulobacteraceae.g__Caulobacter"] = paste("g__Caulobacter (",
format(round(100*mean(y$Root.p__Proteobacteria.c__Alphaproteobacteri
a.o__Caulobacterales.f__Caulobacteraceae.g__Caulobacter),2),
nsmall=2),"%)", sep="")

h["Root.p__Proteobacteria.c__Alphaproteobacteria.o__Caulobacterales.
f__Caulobacteraceae.g__Phenylobacterium"] =
paste("g__Phenylobacterium (",
format(round(100*mean(y$Root.p__Proteobacteria.c__Alphaproteobacteri
a.o__Caulobacterales.f__Caulobacteraceae.g__Phenylobacterium),2),
nsmall=2),"%)", sep="")

h["Root.p__Proteobacteria.c__Alphaproteobacteria.o__Rhizobiales.f__B
radyrhizobiaceae.g__Bradyrhizobium"] = paste("g__Bradyrhizobium (",
format(round(100*mean(y$Root.p__Proteobacteria.c__Alphaproteobacteri
a.o__Rhizobiales.f__Bradyrhizobiaceae.g__Bradyrhizobium),2),
nsmall=2),"%)", sep="")

h["Root.p__Proteobacteria.c__Alphaproteobacteria.o__Rhizobiales.f__H
yphomicrobiaceae.g__Devosia"] = paste("g__Devosia (",
format(round(100*mean(y$Root.p__Proteobacteria.c__Alphaproteobacteri
a.o__Rhizobiales.f__Hyphomicrobiaceae.g__Devosia),2),
nsmall=2),"%)", sep="")

h["Root.p__Proteobacteria.c__Alphaproteobacteria.o__Rhodobacterales.
f__Rhodobacteraceae.g__Thioclava"] = paste("g__Thioclava (",
format(round(100*mean(y$Root.p__Proteobacteria.c__Alphaproteobacteri
a.o__Rhodobacterales.f__Rhodobacteraceae.g__Thioclava),2),
nsmall=2),"%)", sep="")

h["Root.p__Proteobacteria.c__Alphaproteobacteria.o__Sphingomonadales
.f__Sphingomonadaceae.Other"] = paste("f__Sphingomonadaceae (",
format(round(100*mean(y$Root.p__Proteobacteria.c__Alphaproteobacteri
a.o__Sphingomonadales.f__Sphingomonadaceae.Other),2),
nsmall=2),"%)", sep="")

h["Root.p__Proteobacteria.c__Alphaproteobacteria.o__Sphingomonadales
.Other.Other"] = paste("o__Sphingomonadales (",
format(round(100*mean(y$Root.p__Proteobacteria.c__Alphaproteobacteri
a.o__Sphingomonadales.Other.Other),2), nsmall=2),"%)", sep="")

h["Root.p__Proteobacteria.c__Alphaproteobacteria.Other.Other.Other"]
= paste("c__Alphaproteobacteria (",
format(round(100*mean(y$Root.p__Proteobacteria.c__Alphaproteobacteri
a.Other.Other.Other),2), nsmall=2),"%)", sep="")

h["Root.p__Proteobacteria.c__Betaproteobacteria.o__Burkholderiales.f
__Alcaligenaceae.g__Oligella"] = paste("g__Oligella (",
format(round(100*mean(y$Root.p__Proteobacteria.c__Betaproteobacteria
.o__Burkholderiales.f__Alcaligenaceae.g__Oligella),2),
nsmall=2),"%)", sep="")

h["Root.p__Proteobacteria.c__Betaproteobacteria.o__Burkholderiales.f
```

```
__Burkholderiaceae.g__Burkholderia"] = paste("g__Burkholderia (",
format(round(100*mean(y$Root.p__Proteobacteria.c__Betaproteobacteria
.o__Burkholderiales.f__Burkholderiaceae.g__Burkholderia),2),
nsmall=2),"%)", sep="")

h["Root.p__Proteobacteria.c__Betaproteobacteria.o__Burkholderiales.f
__Burkholderiaceae.g__Ralstonia"] = paste("g__Ralstonia (",
format(round(100*mean(y$Root.p__Proteobacteria.c__Betaproteobacteria
.o__Burkholderiales.f__Burkholderiaceae.g__Ralstonia),2),
nsmall=2),"%)", sep="")

h["Root.p__Proteobacteria.c__Betaproteobacteria.o__Burkholderiales.f
__Comamonadaceae.g__Acidovorax"] = paste("g__Acidovorax (",
format(round(100*mean(y$Root.p__Proteobacteria.c__Betaproteobacteria
.o__Burkholderiales.f__Comamonadaceae.g__Acidovorax),2),
nsmall=2),"%)", sep="")

h["Root.p__Proteobacteria.c__Betaproteobacteria.o__Burkholderiales.f
__Oxalobacteraceae.g__Janthinobacterium"] =
paste("g__Janthinobacterium (",
format(round(100*mean(y$Root.p__Proteobacteria.c__Betaproteobacteria
.o__Burkholderiales.f__Oxalobacteraceae.g__Janthinobacterium),2),
nsmall=2),"%)", sep="")

h["Root.p__Proteobacteria.c__Betaproteobacteria.o__Burkholderiales.f
__Oxalobacteraceae.Other"] = paste("f__Oxalobacteraceae (",
format(round(100*mean(y$Root.p__Proteobacteria.c__Betaproteobacteria
.o__Burkholderiales.f__Oxalobacteraceae.Other),2), nsmall=2),"%)",
sep="")

h["Root.p__Proteobacteria.c__Betaproteobacteria.o__Neisseriales.f__N
eisseriaceae.g__"] = paste("f__Neisseriaceae (",
format(round(100*mean(y$Root.p__Proteobacteria.c__Betaproteobacteria
.o__Neisseriales.f__Neisseriaceae.g__),2), nsmall=2),"%)", sep="")

h["Root.p__Proteobacteria.c__Betaproteobacteria.o__Neisseriales.f__N
eisseriaceae.g__Neisseria"] = paste("g__Neisseria (",
format(round(100*mean(y$Root.p__Proteobacteria.c__Betaproteobacteria
.o__Neisseriales.f__Neisseriaceae.g__Neisseria),2), nsmall=2),"%)",
sep="")

h["Root.p__Proteobacteria.c__Betaproteobacteria.o__Neisseriales.f__N
eisseriaceae.g__Simonsiella"] = paste("g__Simonsiella (",
format(round(100*mean(y$Root.p__Proteobacteria.c__Betaproteobacteria
.o__Neisseriales.f__Neisseriaceae.g__Simonsiella),2),
nsmall=2),"%)", sep="")

h["Root.p__Proteobacteria.c__Epsilonproteobacteria.o__Campylobactera
les.f__Campylobacteraceae.g__Campylobacter"] =
paste("g__Campylobacter (",
format(round(100*mean(y$Root.p__Proteobacteria.c__Epsilonproteobacte
ria.o__Campylobacterales.f__Campylobacteraceae.g__Campylobacter),2),
nsmall=2),"%)", sep="")
```

```
h["Root.p__Proteobacteria.c__Gammaproteobacteria.o__Aeromonadales.f_
_Aeromonadaceae.g__Aeromonas"] = paste("g__Aeromonas (",
format(round(100*mean(y$Root.p__Proteobacteria.c__Gammaproteobacteri
a.o__Aeromonadales.f__Aeromonadaceae.g__Aeromonas),2),
nsmall=2),"%)", sep="")

h["Root.p__Proteobacteria.c__Gammaproteobacteria.o__Alteromonadales.
f__Pseudoalteromonadaceae.g__Pseudoalteromonas"] =
paste("g__Pseudoalteromonas (",
format(round(100*mean(y$Root.p__Proteobacteria.c__Gammaproteobacteri
a.o__Alteromonadales.f__Pseudoalteromonadaceae.g__Pseudoalteromonas)
,2), nsmall=2),"%)", sep="")

h["Root.p__Proteobacteria.c__Gammaproteobacteria.o__Enterobacteriale
s.f__Enterobacteriaceae.g__Averyella"] = paste("g__Averyella (",
format(round(100*mean(y$Root.p__Proteobacteria.c__Gammaproteobacteri
a.o__Enterobacteriales.f__Enterobacteriaceae.g__Averyella),2),
nsmall=2),"%)", sep="")

h["Root.p__Proteobacteria.c__Gammaproteobacteria.o__Enterobacteriale
s.f__Enterobacteriaceae.g__Citrobacter"] = paste("g__Citrobacter (",
format(round(100*mean(y$Root.p__Proteobacteria.c__Gammaproteobacteri
a.o__Enterobacteriales.f__Enterobacteriaceae.g__Citrobacter),2),
nsmall=2),"%)", sep="")

h["Root.p__Proteobacteria.c__Gammaproteobacteria.o__Enterobacteriale
s.f__Enterobacteriaceae.g__Escherichia"] = paste("g__Escherichia (",
format(round(100*mean(y$Root.p__Proteobacteria.c__Gammaproteobacteri
a.o__Enterobacteriales.f__Enterobacteriaceae.g__Escherichia),2),
nsmall=2),"%)", sep="")

h["Root.p__Proteobacteria.c__Gammaproteobacteria.o__Enterobacteriale
s.f__Enterobacteriaceae.g__Klebsiella"] = paste("g__Klebsiella (",
format(round(100*mean(y$Root.p__Proteobacteria.c__Gammaproteobacteri
a.o__Enterobacteriales.f__Enterobacteriaceae.g__Klebsiella),2),
nsmall=2),"%)", sep="")

h["Root.p__Proteobacteria.c__Gammaproteobacteria.o__Enterobacteriale
s.f__Enterobacteriaceae.g__Leclercia"] = paste("g__Leclercia (",
format(round(100*mean(y$Root.p__Proteobacteria.c__Gammaproteobacteri
a.o__Enterobacteriales.f__Enterobacteriaceae.g__Leclercia),2),
nsmall=2),"%)", sep="")

h["Root.p__Proteobacteria.c__Gammaproteobacteria.o__Enterobacteriale
s.f__Enterobacteriaceae.g__Proteus"] = paste("g__Proteus (",
format(round(100*mean(y$Root.p__Proteobacteria.c__Gammaproteobacteri
a.o__Enterobacteriales.f__Enterobacteriaceae.g__Proteus),2),
nsmall=2),"%)", sep="")

h["Root.p__Proteobacteria.c__Gammaproteobacteria.o__Enterobacteriale
s.f__Enterobacteriaceae.g__Serratia"] = paste("g__Serratia (",
format(round(100*mean(y$Root.p__Proteobacteria.c__Gammaproteobacteri
a.o__Enterobacteriales.f__Enterobacteriaceae.g__Serratia),2),
nsmall=2),"%)", sep="")
```

```
h["Root.p__Proteobacteria.c__Gammaproteobacteria.o__Enterobacteriale
s.f__Enterobacteriaceae.Other"] = paste("f__Enterobacteriaceae (",
format(round(100*mean(y$Root.p__Proteobacteria.c__Gammaproteobacteri
a.o__Enterobacteriales.f__Enterobacteriaceae.Other),2),
nsmall=2),"%)", sep="")

h["Root.p__Proteobacteria.c__Gammaproteobacteria.o__Oceanospirillale
s.f__Halomonadaceae.g__Halomonas"] = paste("g__Halomonas (",
format(round(100*mean(y$Root.p__Proteobacteria.c__Gammaproteobacteri
a.o__Oceanospirillales.f__Halomonadaceae.g__Halomonas),2),
nsmall=2),"%)", sep="")

h["Root.p__Proteobacteria.c__Gammaproteobacteria.o__Pasteurellales.f
__Pasteurellaceae.g__Actinobacillus"] = paste("g__Actinobacillus (",
format(round(100*mean(y$Root.p__Proteobacteria.c__Gammaproteobacteri
a.o__Pasteurellales.f__Pasteurellaceae.g__Actinobacillus),2),
nsmall=2),"%)", sep="")

h["Root.p__Proteobacteria.c__Gammaproteobacteria.o__Pasteurellales.f
__Pasteurellaceae.g__Haemophilus"] = paste("g__Haemophilus (",
format(round(100*mean(y$Root.p__Proteobacteria.c__Gammaproteobacteri
a.o__Pasteurellales.f__Pasteurellaceae.g__Haemophilus),2),
nsmall=2),"%)", sep="")

h["Root.p__Proteobacteria.c__Gammaproteobacteria.o__Pasteurellales.f
__Pasteurellaceae.g__Pasteurella"] = paste("g__Pasteurella (",
format(round(100*mean(y$Root.p__Proteobacteria.c__Gammaproteobacteri
a.o__Pasteurellales.f__Pasteurellaceae.g__Pasteurella),2),
nsmall=2),"%)", sep="")

h["Root.p__Proteobacteria.c__Gammaproteobacteria.o__Pseudomonadales.
f__Moraxellaceae.g__"] = paste("f__Moraxellaceae (",
format(round(100*mean(y$Root.p__Proteobacteria.c__Gammaproteobacteri
a.o__Pseudomonadales.f__Moraxellaceae.g__),2), nsmall=2),"%)",
sep="")

h["Root.p__Proteobacteria.c__Gammaproteobacteria.o__Pseudomonadales.
f__Moraxellaceae.g__Acinetobacter"] = paste("g__Acinetobacter (",
format(round(100*mean(y$RRoot.p__Proteobacteria.c__Gammaproteobacter
ia.o__Pseudomonadales.f__Moraxellaceae.g__Acinetobacter),2),
nsmall=2),"%)", sep="")

h["Root.p__Proteobacteria.c__Gammaproteobacteria.o__Pseudomonadales.
f__Moraxellaceae.g__Moraxella"] = paste("g__Moraxella (",
format(round(100*mean(y$Root.p__Proteobacteria.c__Gammaproteobacteri
a.o__Pseudomonadales.f__Moraxellaceae.g__Moraxella),2),
nsmall=2),"%)", sep="")

h["Root.p__Proteobacteria.c__Gammaproteobacteria.o__Pseudomonadales.
f__Pseudomonadaceae.g__Pseudomonas"] = paste("g__Pseudomonas (",
format(round(100*mean(y$Root.p__Proteobacteria.c__Gammaproteobacteri
a.o__Pseudomonadales.f__Pseudomonadaceae.g__Pseudomonas),2),
nsmall=2),"%)", sep="")
```

```
h["Root.p__Proteobacteria.c__Gammaproteobacteria.o__Pseudomonadales.
f__Pseudomonadaceae.Other"] = paste("f__Pseudomonadaceae (",
format(round(100*mean(y$Root.p__Proteobacteria.c__Gammaproteobacteri
a.o__Pseudomonadales.f__Pseudomonadaceae.Other),2), nsmall=2),"%)",
sep="")

h["Root.p__Proteobacteria.c__Gammaproteobacteria.o__Vibrionales.f__V
ibrionaceae.g__Photobacterium"] = paste("g__Photobacterium (",
format(round(100*mean(y$Root.p__Proteobacteria.c__Gammaproteobacteri
a.o__Vibrionales.f__Vibrionaceae.g__Photobacterium),2),
nsmall=2),"%)", sep="")

h["Root.p__Proteobacteria.c__Gammaproteobacteria.o__Vibrionales.f__V
ibrionaceae.g__Vibrio"] = paste("g__Vibrio (",
format(round(100*mean(y$Root.p__Proteobacteria.c__Gammaproteobacteri
a.o__Vibrionales.f__Vibrionaceae.g__Vibrio),2), nsmall=2),"%)",
sep="")

h["Root.p__Proteobacteria.c__Gammaproteobacteria.o__Vibrionales.f__V
ibrionaceae.Other"] = paste("f__Vibrionaceae (",
format(round(100*mean(y$Root.p__Proteobacteria.c__Gammaproteobacteri
a.o__Vibrionales.f__Vibrionaceae.Other),2), nsmall=2),"%)", sep="")

h["Root.p__Proteobacteria.c__Gammaproteobacteria.o__Xanthomonadales.
f__Xanthomonadaceae.g__Stenotrophomonas"] =
paste("g__Stenotrophomonas (",
format(round(100*mean(y$Root.p__Proteobacteria.c__Gammaproteobacteri
a.o__Xanthomonadales.f__Xanthomonadaceae.g__Stenotrophomonas),2),
nsmall=2),"%)", sep="")

h["Root.p__Tenericutes.c__Erysipelotrichi.o__Erysipelotrichales.f__E
rysipelotrichaceae.g__"] = paste("f__Erysipelotrichaceae (",
format(round(100*mean(y$Root.p__Tenericutes.c__Erysipelotrichi.o__Er
ysipelotrichales.f__Erysipelotrichaceae.g__),2), nsmall=2),"%)",
sep="")

h["Root.p__Tenericutes.c__Erysipelotrichi.o__Erysipelotrichales.f__E
rysipelotrichaceae.g__Catenibacterium"] = paste("g__Catenibacterium
(",
format(round(100*mean(y$Root.p__Tenericutes.c__Erysipelotrichi.o__Er
ysipelotrichales.f__Erysipelotrichaceae.g__Catenibacterium),2),
nsmall=2),"%)", sep="")

h["Root.p__Tenericutes.c__Erysipelotrichi.o__Erysipelotrichales.f__E
rysipelotrichaceae.g__Clostridium"] = paste("g__Clostridium (",
format(round(100*mean(y$Root.p__Tenericutes.c__Erysipelotrichi.o__Er
ysipelotrichales.f__Erysipelotrichaceae.g__Clostridium),2),
nsmall=2),"%)", sep="")

h["Root.p__Tenericutes.c__Erysipelotrichi.o__Erysipelotrichales.f__E
rysipelotrichaceae.g__Coprobacillus"] = paste("g__Coprobacillus (",
format(round(100*mean(y$Root.p__Tenericutes.c__Erysipelotrichi.o__Er
ysipelotrichales.f__Erysipelotrichaceae.g__Coprobacillus),2),
```

````
nsmall=2),"%)", sep="")

h["Root.p__Tenericutes.c__Mollicutes.o__Mycoplasmatales.f__Mycoplasm
ataceae.g__Mycoplasma"] = paste("g__Mycoplasma (",
format(round(100*mean(y$Root.p__Tenericutes.c__Mollicutes.o__Mycopla
smatales.f__Mycoplasmataceae.g__Mycoplasma),2), nsmall=2),"%)",
sep="")

h["Root.p__Thermi.c__Deinococci.o__Deinococcales.f__Deinococcaceae.g
__Deinococcus"] = paste("g__Deinococcus (",
format(round(100*mean(y$Root.p__Thermi.c__Deinococci.o__Deinococcale
s.f__Deinococcaceae.g__Deinococcus),2), nsmall=2),"%)", sep="")

h["Root.p__Verrucomicrobia.c__Verrucomicrobiae.o__Verrucomicrobiales
.f__Verrucomicrobiaceae.g__Akkermansia"] = paste("g__Akkermansia (",
format(round(100*mean(y$Root.p__Verrucomicrobia.c__Verrucomicrobiae.
o__Verrucomicrobiales.f__Verrucomicrobiaceae.g__Akkermansia),2),
nsmall=2),"%)", sep="")
  h["Root.Other.Other.Other.Other.Other"] = paste("Root (",
format(round(100*mean(y$Root.Other.Other.Other.Other.Other),2),
nsmall=2),"%)", sep="")
  h["Taxa < 1%"] = paste("Taxa < 1% (",
format(round(100*mean(y$"Taxa < 1%"),2), nsmall=2),"%)", sep="")
  h["other"] = paste("Other (", format(round(100*mean(y$other),2),
nsmall=2),"%)", sep="")
  #Make a vector of colours to return
  labels <- character(0)
  for (i in 1:length(x)){
      col <- h[[x[i]]]
      #print(col)
      if (is.null(col)) {
        print(x[i])
      }
      labels <- c(labels, col)
  }
  return(labels)
}
```

Setup: function to convert to relative abundance (normf function)
```{r}
#Convert to relative abundance data
  normf = function(x) {
  x/sum(x)
  }
```

# Load dataset

Load dataset: define working directory; load phyloseq object
```{r load, include=FALSE}
setwd(<set to your own working directory>)
otufile = read.csv("seqtab_dada2_output.csv", quote='"',
row.names=1)
````

````
OTU = otu_table(otufile, taxa_are_rows = TRUE)
taxfile = as.matrix(read.csv("taxa_dada2_output.csv", quote='"',
row.names=1))
TAX = tax_table(taxfile)
MAP = import_qiime_sample_data("mapping_file.txt")
qd = phyloseq(OTU, TAX, MAP)
```

Load dataset: reorder clusters for aesthetic reasons
```{r}
recluster <- as.character(sample_data(qd)$cluster)
#add heading to differentiate
recluster[recluster=="4"] <- "cluster1"
recluster[recluster=="6"] <- "cluster2"
recluster[recluster=="8"] <- "cluster3"
recluster[recluster=="7"] <- "cluster4"
recluster[recluster=="5"] <- "cluster5"
recluster[recluster=="3"] <- "cluster6"
recluster[recluster=="9"] <- "cluster7"
recluster[recluster=="2"] <- "cluster8"
recluster[recluster=="1"] <- "cluster9"
#now get rid of heading
recluster[recluster=="cluster1"] <- "1"
recluster[recluster=="cluster2"] <- "2"
recluster[recluster=="cluster3"] <- "3"
recluster[recluster=="cluster4"] <- "4"
recluster[recluster=="cluster5"] <- "5"
recluster[recluster=="cluster6"] <- "6"
recluster[recluster=="cluster7"] <- "7"
recluster[recluster=="cluster8"] <- "8"
recluster[recluster=="cluster9"] <- "9"

sample_data(qd)$cluster <- recluster
```

# Process taxa: prune non-bacterial ASVs
```{r}
# qd.BactCounts = subset_taxa(qd, Kingdom=="Bacteria")
# counts <- sample_sums(qd.BactCounts)
# subset(counts, counts < 1000)
# psmelt.qd = psmelt(qd)
# subset.bac <- subset(psmelt.qd, Kingdom == "Bacteria")
# qd.bac = prune_taxa(subset.bac$OTU, qd)
#
# qd=qd.bac
# qd.norm = transform_sample_counts(qd, normf)
```

Load dataset: set variable types correctly (i.e. discrete,
continuous):
```{r}
sample_data(qd)$Age_at_enrollment <-
as.numeric(as.character(sample_data(qd)$Age_at_enrollment))
sample_data(qd)$Num_Medications <-
````

as.numeric(as.character(sample_data(qd)$Num_Medications))
sample_data(qd)$Barthel_bowel <-
as.numeric(as.character(sample_data(qd)$Barthel_bowel))
sample_data(qd)$Barthel_bladder <-
as.numeric(as.character(sample_data(qd)$Barthel_bladder))
sample_data(qd)$Barthel_grooming <-
as.numeric(as.character(sample_data(qd)$Barthel_grooming))
sample_data(qd)$Barthel_toilet_use <-
as.numeric(as.character(sample_data(qd)$Barthel_toilet_use))
sample_data(qd)$Barthel_feeding <-
as.numeric(as.character(sample_data(qd)$Barthel_feeding))
sample_data(qd)$Barthel_transfer <-
as.numeric(as.character(sample_data(qd)$Barthel_transfer))
sample_data(qd)$Barthel_mobility <-
as.numeric(as.character(sample_data(qd)$Barthel_mobility))
sample_data(qd)$Barthel_dressing <-
as.numeric(as.character(sample_data(qd)$Barthel_dressing))
sample_data(qd)$Barthel_stairs <-
as.numeric(as.character(sample_data(qd)$Barthel_stairs))
sample_data(qd)$Barthel_bathing <-
as.numeric(as.character(sample_data(qd)$Barthel_bathing))
sample_data(qd)$Barthel_Total <-
as.numeric(as.character(sample_data(qd)$Barthel_Total))
sample_data(qd)$Barthel_final_score_100 <-
as.numeric(as.character(sample_data(qd)$Barthel_final_score_100))
sample_data(qd)$Num_Comorbidities <-
as.numeric(as.character(sample_data(qd)$Num_Comorbidities))
sample_data(qd)$qPCR_Concentration <-
as.numeric(as.character(sample_data(qd)$qPCR_Concentration))
sample_data(qd)$InflamIndex <-
as.numeric(as.character(sample_data(qd)$InflamIndex))
sample_data(qd)$Year <- as.character(sample_data(qd)$Year)
sample_data(qd)$IL1B <- as.numeric(as.character(sample_data(qd)
$IL1B))
sample_data(qd)$IL6 <- as.numeric(as.character(sample_data(qd)$IL6))
sample_data(qd)$TNFA <- as.numeric(as.character(sample_data(qd)
$TNFA))
sample_data(qd)$cluster <- factor(sample_data(qd)$cluster,
levels=c("1","2","3","4","5","6","7","8","9","10"))
```

Process dataset: remove samples that do not meet quality standards
```{r cull, include=FALSE}
#1Cull samples with a qPCR concentration below the level of
detection
qPCR <- sample_data(qd)$qPCR_Concentration
qPCR <- qPCR[!qPCR %in% '.']
qPCR <- as.numeric(as.character(qPCR))
qPCR <- sort(qPCR)
qPCR.orig <- qPCR
#qPCR concentration is in ng; convert to gs (x/1000000000); c = mol
weight * genome size of standard which is = 660 * 4641652 =
3063490320; moles = x / c; molecules = x *6.0223*10^23; # of 16S
copies = x * 7

```
qPCR <- qPCR/1000000000
qPCR <- qPCR/3063490320
qPCR <- qPCR*6.0223*10^23
qPCR <- qPCR*7
qPCR.df <- as.data.frame(qPCR)
p <- ggplot(qPCR.df, aes(y = qPCR, x = seq(1, length(qPCR))))
p <- p + geom_point() +
  scale_y_log10() + #labels = scales::comma) #to force non-
scientific notation
  theme_bw() +
  theme(axis.title.x=element_blank(),
        axis.text.x=element_blank(),
        axis.ticks.x=element_blank()) +
  ylab("qPCR Concentration") +
  geom_hline(yintercept=1.38*10^3, colour="gray", linetype="dashed")
p

#The level of detection of the qPCR is at 10^3; let's cull the
samples before this.
#Show me the sample IDs with qPCR < 0.001ng
(0.001/1000000000/3063490320*6.0223*10^23*7=1.38*10^3, NTC)
#Also remove samples without a qPCR reading
sd <- sample_data(qd)
sd$SampleID[sd$qPCR_Concentration %in% '.']
qd = subset_samples(qd, SampleID != "ML49")
qd = subset_samples(qd, SampleID != "ML467")
sd <- subset(sd, !sd$qPCR_Concentration %in% '.')
sd$qPCR_Concentration <-
as.numeric(as.character(sd$qPCR_Concentration))
sd$SampleID[sd$qPCR_Concentration < 0.001]
qd = subset_samples(qd, SampleID != "ML377")
qd = subset_samples(qd, SampleID != "ML485")
qd = subset_samples(qd, SampleID != "JJ228")
qd = subset_samples(qd, SampleID != "ML63")
qd = subset_samples(qd, SampleID != "ML66")
qd = subset_samples(qd, SampleID != "ML69")
qd = subset_samples(qd, SampleID != "ML70")
qd = subset_samples(qd, SampleID != "ML71")
qd = subset_samples(qd, SampleID != "ML155")
qd = subset_samples(qd, SampleID != "ML200")
qd = subset_samples(qd, SampleID != "ML216")
qd = subset_samples(qd, SampleID != "ML278")
qd = subset_samples(qd, SampleID != "ML292")
qd = subset_samples(qd, SampleID != "ML297")
qd = subset_samples(qd, SampleID != "ML300")
qd = subset_samples(qd, SampleID != "ML301")
qd = subset_samples(qd, SampleID != "ML305")
qd = subset_samples(qd, SampleID != "JJa310")
```

Process dataset: ensure dataset is loaded properly and matches what we know about the data from the mapping file
i.e. 334 samples, 13463 taxa.

```{r qd-data}
```

qd
```

Setup dataset: split samples into subsets qd.baseline (first sample only), qd.healthy (all non-ILI samples)
```{r subset}
#Pre-define L6 and relabund vars
qd.L6 <- tax_glom(qd, taxrank="Genus")
qd.L6.norm = transform_sample_counts(qd.L6, normf)

qd.norm = transform_sample_counts(qd, normf)

qd.healthy <- subset_samples(qd, Type.B.E.F. != "E")
qd.healthy

qd.baseline = subset_samples(qd, Type.B.E.F. == "B")
qd.baseline
qd.baseline.norm = transform_sample_counts(qd.baseline, normf)
#plyr::count(sample_data(qd.baseline)$ID) #make sure there is only 1 B per participant
```

Pre-analysis: Cluster samples based on composition (qd)

How many clusters are in the data?
```{r}
#Bray curtis, hierarchical clustering
qd.rare = rarefy_even_depth(qd, sample.size = min(sample_sums(qd)), rngseed=1414)
exord = ordinate(qd.rare, method="PCoA", distance="bray")
x = phyloseq:::scores.pcoa(exord, display="sites")
head(x)
dist1 <- phyloseq::distance(qd.rare, method = "bray") #calc dist matrix

nb.hi <- NbClust(data = x, diss = dist1, distance = NULL, min.nc = 2, max.nc = 20, method = "complete", index = "all", alphaBeale = 0.1)
table(nb.hi$Best.partition)
#n=10 with 1 n=1 cluster prior to removing the single sample from the input dataset to make 9 clusters
```

Define df.all, dist.all, iMDS.all for all samples in the dataset to be used for Bray Curtis and Aitchinson work for the rest of the script
```{r}
qd.all.rare = rarefy_even_depth(qd, sample.size = min(sample_sums(qd)), rngseed=1414)
df.all = as(sample_data(qd.all.rare), "data.frame")
dist.all <- phyloseq::distance(qd.all.rare, method = "bray")
iMDS.all <- ordinate(qd.all.rare, "PCoA", distance = dist.all)

qd.all.clr <- microbiome::transform(qd, "clr")

````
df.ait.all = as(sample_data(qd.all.clr), "data.frame")
dist.ait.all <- phyloseq::distance(qd.all.clr, method = "euclidean")
iMDS.ait.all <- ordinate(qd.all.clr, method = "RDA", distance =
dist.ait.all)
```

Cluster samples using hierarchical clustering
```{r}
#Cluster data
qd.rare = rarefy_even_depth(qd, sample.size = min(sample_sums(qd)),
rngseed=1414)
dist1 <- phyloseq::distance(qd.rare, method = "bray")
hier.cluster <- hclust(dist1)
# dend <- as.dendrogram(hier.cluster)
# dend <- color_branches(dend, k=10) # Color the branches based on
the clusters
# plot(dend)

#Define clustering and label order variables for later use
hc.labels <- cutree(hier.cluster, k=9)
hc.order <- labels(hier.cluster)
```

Update mapping file with cluster information; reload, and don't go
through the process of clustering again

Setup clustering vars: qd.clust, qd.baseline.clust,
qd.healthy.clust, #qd.event.clust
```{r}
#Clustering vars (qd)
clustdf <- data.frame(cluster = sample_data(qd)$cluster, #hc.labels,
                    SampleID= sample_data(qd)$SampleID)
clustdf$cluster <- factor(clustdf$cluster,
levels=c("1","2","3","4","5","6","7","8","9"))
sample.data <- as(sample_data(qd), "data.frame")
qd.clust.sam <- merge(clustdf, sample.data, by.x="SampleID",
by.y="SampleID")
#Create df.clust with cluster data in the right format for input
into lm
qd.clust.sam$cluster <- qd.clust.sam$cluster.x
qd.clust <- mutate(qd.clust.sam, Cluster1 = case_when(cluster == '1'
~ 1, TRUE ~ 0))
qd.clust <- mutate(qd.clust, Cluster2 = case_when(cluster == '2' ~
1, TRUE ~ 0))
qd.clust <- mutate(qd.clust, Cluster3 = case_when(cluster == '3' ~
1, TRUE ~ 0))
qd.clust <- mutate(qd.clust, Cluster4 = case_when(cluster == '4' ~
1, TRUE ~ 0))
qd.clust <- mutate(qd.clust, Cluster5 = case_when(cluster == '5' ~
1, TRUE ~ 0))
qd.clust <- mutate(qd.clust, Cluster6 = case_when(cluster == '6' ~
1, TRUE ~ 0))
qd.clust <- mutate(qd.clust, Cluster7 = case_when(cluster == '7' ~
1, TRUE ~ 0))
````

```
qd.clust <- mutate(qd.clust, Cluster8 = case_when(cluster == '8' ~
1, TRUE ~ 0))
qd.clust <- mutate(qd.clust, Cluster9 = case_when(cluster == '9' ~
1, TRUE ~ 0))

#Clustering vars (qd.baseline)
clustdf.b <- data.frame(cluster = sample_data(qd.baseline)$cluster,
                    SampleID= sample_data(qd.baseline)$SampleID)
clustdf.b$cluster <- factor(clustdf.b$cluster,
levels=c("1","2","3","4","5","6","7","8","9"))
sample.data <- as(sample_data(qd.baseline), "data.frame")
qd.baseline.clust.sam <- merge(clustdf.b, sample.data,
by.x="SampleID", by.y="SampleID")
#Create df.clust with cluster data in the right format for input
into lm
qd.baseline.clust.sam$cluster <- qd.baseline.clust.sam$cluster.x
qd.baseline.clust <- mutate(qd.baseline.clust.sam, Cluster1 =
case_when(cluster == '1' ~ 1, TRUE ~ 0))
qd.baseline.clust <- mutate(qd.baseline.clust, Cluster2 =
case_when(cluster == '2' ~ 1, TRUE ~ 0))
qd.baseline.clust <- mutate(qd.baseline.clust, Cluster3 =
case_when(cluster == '3' ~ 1, TRUE ~ 0))
qd.baseline.clust <- mutate(qd.baseline.clust, Cluster4 =
case_when(cluster == '4' ~ 1, TRUE ~ 0))
qd.baseline.clust <- mutate(qd.baseline.clust, Cluster5 =
case_when(cluster == '5' ~ 1, TRUE ~ 0))
qd.baseline.clust <- mutate(qd.baseline.clust, Cluster6 =
case_when(cluster == '6' ~ 1, TRUE ~ 0))
qd.baseline.clust <- mutate(qd.baseline.clust, Cluster7 =
case_when(cluster == '7' ~ 1, TRUE ~ 0))
qd.baseline.clust <- mutate(qd.baseline.clust, Cluster8 =
case_when(cluster == '8' ~ 1, TRUE ~ 0))
qd.baseline.clust <- mutate(qd.baseline.clust, Cluster9 =
case_when(cluster == '9' ~ 1, TRUE ~ 0))

#Clustering vars (qd.healthy)
clustdf.h <- data.frame(cluster = sample_data(qd.healthy)$cluster,
                    SampleID= sample_data(qd.healthy)$SampleID)
clustdf.h$cluster <- factor(clustdf.h$cluster,
levels=c("1","2","3","4","5","6","7","8","9"))
sample.data <- as(sample_data(qd.healthy), "data.frame")
qd.healthy.clust.sam <- merge(clustdf.h, sample.data,
by.x="SampleID", by.y="SampleID")
#Create df.clust with cluster data in the right format for input
into lm
qd.healthy.clust.sam$cluster <- qd.healthy.clust.sam$cluster.x
qd.healthy.clust <- mutate(qd.healthy.clust.sam, Cluster1 =
case_when(cluster == '1' ~ 1, TRUE ~ 0))
qd.healthy.clust <- mutate(qd.healthy.clust, Cluster2 =
case_when(cluster == '2' ~ 1, TRUE ~ 0))
qd.healthy.clust <- mutate(qd.healthy.clust, Cluster3 =
case_when(cluster == '3' ~ 1, TRUE ~ 0))
qd.healthy.clust <- mutate(qd.healthy.clust, Cluster4 =
case_when(cluster == '4' ~ 1, TRUE ~ 0))
```

```
qd.healthy.clust <- mutate(qd.healthy.clust, Cluster5 =
case_when(cluster == '5' ~ 1, TRUE ~ 0))
qd.healthy.clust <- mutate(qd.healthy.clust, Cluster6 =
case_when(cluster == '6' ~ 1, TRUE ~ 0))
qd.healthy.clust <- mutate(qd.healthy.clust, Cluster7 =
case_when(cluster == '7' ~ 1, TRUE ~ 0))
qd.healthy.clust <- mutate(qd.healthy.clust, Cluster8 =
case_when(cluster == '8' ~ 1, TRUE ~ 0))
qd.healthy.clust <- mutate(qd.healthy.clust, Cluster9 =
case_when(cluster == '9' ~ 1, TRUE ~ 0))
```

Supplemental Figure 1: Timeline of sampling events (qd)

Timeline Figure of sample collection dates

```{r}
#Set local time
Sys.setlocale("LC_TIME", "C")
#Get time data out of metadata information
collect.dates <- as.matrix(sample_data(qd)[,c(1,4,11,18,19)])
#SampleID, ID, CollectionDate.Y.M.D., Swab, Type.B.E.F.
collect.dates <- as.data.frame(collect.dates)
#Convert date information to as.Date class
collect.dates[,3] <- as.Date(collect.dates[,3], "%d/%m/%Y")
#Make zoo object
ncol(collect.dates)
ts.A <- zoo(collect.dates[,1:5], order.by = collect.dates[,3])
#Convert zoo object into dataframe
df.A <- data.frame(value = ts.A[,2],
                     time = time(ts.A),
                     sampleid = ts.A[,1],
                     personid = ts.A[,2],
                     sampletype = ts.A[,5])
#Convert data information in zoo object to as.Date class
df.A$time <- as.Date(df.A$time)
#Plot
man.colors <- c("#377eb8","#e41a1c","black")
p1 <- ggplot(df.A, aes(y = value, x = time, group = personid, colour
= sampletype)) +
  geom_line(colour="gray") +
  scale_color_manual(values=man.colors, name = "Sample type", labels
= c("pre-C&F", "C&F", "post-C&F")) +
  theme_classic() +
  theme(axis.text.y =  element_blank(),
        axis.ticks.y = element_blank(),
        axis.title.y = element_blank(),
        axis.text.x = element_text(angle=45, hjust=1),
        legend.position = c(0.95,0.1)) +
  scale_x_date(breaks = as.Date(c("2011-01-01", "2011-05-17",
                                  "2013-11-01", "2014-08-01",
                                  "2014-12-01", "2015-06-01",
                                  "2015-11-01", "2016-06-01",
                                  "2016-11-01", "2017-05-01")),
               minor_breaks = as.Date(c("2015-06-01"))) +
```

```
  xlab("Date of sample collection") +
  geom_point()
p1
pdf("figures/SupFigure1.pdf", height=8, width=12)
plot(p1)
dev.off()
```

Table 1: Some basic stats about the population (qd.baseline)

Age (mean, SDEV),sex ratios etc. of the individuals in the dataset
```{r}
plyr::count(sample_data(qd.baseline)$Sex)
mean(sample_data(qd.baseline)$Age_at_enrollment)
median(sample_data(qd.baseline)$Age_at_enrollment)
sd(sample_data(qd.baseline)$Age_at_enrollment)
plyr::count(sample_data(qd.baseline)$Smoker)
mean(sample_data(qd.baseline)$Num_Medications, na.rm = TRUE)
median(sample_data(qd.baseline)$Num_Medications, na.rm = TRUE)
sd(sample_data(qd.baseline)$Num_Medications, na.rm = TRUE)
mean(sample_data(qd.baseline)$Num_Comorbidities, na.rm = TRUE)
median(sample_data(qd.baseline)$Num_Comorbidities, na.rm = TRUE)
sd(sample_data(qd.baseline)$Num_Comorbidities, na.rm = TRUE)
mean(sample_data(qd.baseline)$Barthel_final_score_100, na.rm = TRUE)
median(sample_data(qd.baseline)$Barthel_final_score_100, na.rm =
TRUE)
sd(sample_data(qd.baseline)$Barthel_final_score_100, na.rm = TRUE)
min(sample_data(qd.baseline)$Barthel_final_score_100)
max(sample_data(qd.baseline)$Barthel_final_score_100)
mean(sample_data(qd.baseline)$Barthel_Total, na.rm = TRUE)
median(sample_data(qd.baseline)$Barthel_Total, na.rm = TRUE)
sd(sample_data(qd.baseline)$Barthel_Total, na.rm = TRUE)
min(sample_data(qd.baseline)$Barthel_Total)
max(sample_data(qd.baseline)$Barthel_Total)
table(sample_data(qd.baseline)$hadEvent)
table(sample_data(qd.event)$Type.B.E.F.)
avg_events_perperson = 57/51
avg_events_perperson
table(subset(cbind(sample_data(qd.event)$Type.B.E.F.,
sample_data(qd.event)$ID), sample_data(qd.event)$Type.B.E.F. ==
"E"))
median(c(1,2,1,2,1,1,1,1,1,1,1,1,1,1,1,1,2,1,1,1,1,1,1,2,2,1,1,1,1,1
,1,1,1,1,1,1,1,1,
         1,1,1,1,1,1,1,2,1,2,1,1))
  plyr::count(sample_data(qd.baseline)$Allocation_Group_Probiotics)
```

Sup Table 4: Basic stats like Table 1 but split by probiotic group
Age (mean, SDEV),sex ratios etc. of the individuals in the dataset
```{r}
qd.event.B <- subset_samples(qd.event, Type.B.E.F. == "B")
qd.event.B
qd.event.B.Active <- subset_samples(qd.event.B,
Allocation_Group_Probiotics == "Active")
```

```
qd.event.B.Placeb <- subset_samples(qd.event.B,
Allocation_Group_Probiotics == "Placebo")
qd.event.B.Active
qd.event.B.Placeb

plyr::count(sample_data(qd.event.B.Active)$Sex)
plyr::count(sample_data(qd.event.B.Placeb)$Sex)

mean(sample_data(qd.event.B.Active)$Age_at_enrollment)
mean(sample_data(qd.event.B)$Age_at_enrollment)

median(sample_data(qd.event.B.Active)$Age_at_enrollment)
median(sample_data(qd.event.B.Placeb)$Age_at_enrollment)

sd(sample_data(qd.event.B.Active)$Age_at_enrollment)
sd(sample_data(qd.event.B.Placeb)$Age_at_enrollment)

plyr::count(sample_data(qd.event.B.Active)$Smoker)
plyr::count(sample_data(qd.event.B.Placeb)$Smoker)

median(sample_data(qd.event.B.Active)$Num_Medications, na.rm = TRUE)
median(sample_data(qd.event.B.Placeb)$Num_Medications, na.rm = TRUE)

sd(sample_data(qd.event.B.Active)$Num_Medications, na.rm = TRUE)
sd(sample_data(qd.event.B.Placeb)$Num_Medications, na.rm = TRUE)

median(sample_data(qd.event.B.Active)$Num_Comorbidities, na.rm =
TRUE)
median(sample_data(qd.event.B.Placeb)$Num_Comorbidities, na.rm =
TRUE)

sd(sample_data(qd.event.B.Active)$Num_Comorbidities, na.rm = TRUE)
sd(sample_data(qd.event.B.Placeb)$Num_Comorbidities, na.rm = TRUE)

median(sample_data(qd.event.B.Active)$Barthel_final_score_100, na.rm
= TRUE)
median(sample_data(qd.event.B.Placeb)$Barthel_final_score_100, na.rm
= TRUE)

sd(sample_data(qd.event.B.Active)$Barthel_final_score_100, na.rm =
TRUE)
sd(sample_data(qd.event.B.Placeb)$Barthel_final_score_100, na.rm =
TRUE)

table(sample_data(qd.event.B.Active)$hadEvent)
table(sample_data(qd.event.B.Placeb)$hadEvent)
```

Figure 1: Details of the clustering results of the population (qd)

Cluster colour definition

```{r}
cluster.colours.k10 <- c("9" = "#d7663a",
                         "10" = "#8765e2",
```

````
                         "6" = "#8dd94f",
                         "7" = "#d44bc4",
                         "8" = "#6b9b4d",
                         "2" = "#c97ebe",
                         "3" = "#65d2ad",
                         "4" = "#d75b74",
                         "5" = "#6e94d5",
                         "1"= "#c9aa4e")
```

A. Draw dendrogram based on hierarchical clustering
```{r}
library("ggtree")
#Plot clustering results
g <- split(names(hc.labels), hc.labels)
p <- ggtree(hier.cluster, ladderize=FALSE)
clades <- sapply(g, function(n) MRCA(p, n))
p <- groupClade(p, clades, group_name='subtree')
d <- data.frame(label = names(hc.labels),
                  clust = sample_data(qd)$cluster)
p.tree <- p %<+% d +
  layout_dendrogram() +
  geom_tippoint(size=2, shape=15, aes(colour=factor(clust))) +
  scale_colour_manual(values = cluster.colours.k10) +
  theme(legend.position="none")
p.tree
detach("package:ggtree", unload=TRUE)
```

A1. PCOA by cluster data
```{r}
qd.rare = rarefy_even_depth(qd, sample.size = min(sample_sums(qd)),
rngseed=1414)
dist <- phyloseq::distance(qd.rare, method = "bray")
iMDS <- ordinate(qd.rare, "PCoA", distance = dist)

qd.clr = microbiome::transform(qd, "clr")
dist.ait = phyloseq::distance(qd.clr, method="euclidean")
iMDS.ait <- ordinate(qd.clr, "PCoA", distance = dist.ait)

clust.pcoa12 <- plot_ordination(qd.rare, axes=c(1,2), iMDS) +
  theme_bw() +
  theme(legend.title=element_blank(),
        legend.position="none") +
  geom_point(size = 2, shape=21, colour="black",
aes(fill=clustdf$cluster)) +
  scale_fill_manual(values=c(cluster.colours.k10))
clust.pcoa12

clust.pcoa12.leg <- plot_ordination(qd.rare, axes=c(1,2), iMDS) +
  theme_bw() +
  theme(legend.title=element_blank()) +
  geom_point(size = 2, shape=21, colour="black",
aes(fill=clustdf$cluster)) +
````

```
  scale_fill_manual(values=c(cluster.colours.k10))

clust.pcoa13 <- plot_ordination(qd.rare, axes=c(1,3), iMDS) +
  theme_bw() +
  theme(legend.title=element_blank(),
        legend.position="none") +
  geom_point(size = 2, shape=21, colour="black",
aes(fill=clustdf$cluster)) +
  scale_fill_manual(values=c(cluster.colours.k10))

clust.pcoa23 <- plot_ordination(qd.rare, axes=c(2,3), iMDS) +
  theme_bw() +
  theme(legend.title=element_blank(),
        legend.position="none") +
  geom_point(size = 2, shape=21, colour="black",
aes(fill=clustdf$cluster)) +
  scale_fill_manual(values=c(cluster.colours.k10))

clust.pcoa14 <- plot_ordination(qd.rare, axes=c(1,4), iMDS) +
  theme_bw() +
  theme(legend.title=element_blank(),
        legend.position="none") +
  geom_point(size = 2, shape=21, colour="black",
aes(fill=clustdf$cluster)) +
  scale_fill_manual(values=c(cluster.colours.k10))

clust.pcoa24 <- plot_ordination(qd.rare, axes=c(2,4), iMDS) +
  theme_bw() +
  theme(legend.title=element_blank(),
        legend.position="none") +
  geom_point(size = 2, shape=21, colour="black",
aes(fill=clustdf$cluster)) +
  scale_fill_manual(values=c(cluster.colours.k10))

clust.pcoa34 <- plot_ordination(qd.rare, axes=c(3,4), iMDS) +
  theme_bw() +
  theme(legend.title=element_blank(),
        legend.position="none") +
  geom_point(size = 2, shape=21, colour="black",
aes(fill=clustdf$cluster)) +
  scale_fill_manual(values=c(cluster.colours.k10))
```

A2. Cluster dispersion statistics
Work modelled off of this tutorial: https://cran.r-project.org/web/packages/hagis/vignettes/betadiversity.html

```{r}
table(clustdf$cluster)

disp <- betadisper(dist, clustdf$cluster)
disp.anova <- anova(disp)
disp.anova #p=< 2.2e-16*** clusters are significantly dispersed
disp.TukeyHSD <- TukeyHSD(disp) # test significance between each
group
```

````
#disp.TukeyHSD
plot(disp, hull = FALSE, ellipse = TRUE)
plot(disp.TukeyHSD)
#PERMANOVA tests if the centroids, similar to means, of each group
are significantly different from each other.
adonis.out <- adonis(dist ~ clustdf$cluster)
adonis.out #p=0.001***
#Analysis of Similarity (ANOSIM)
anosim.out <- anosim(dist, grouping = clustdf$cluster)
anosim.out
#R = 0.6896; Positive numbers suggest that there is more similarity
within groups than there is between groups.
#signif = 0.001; so there is a significant difference between
groups' similiarities.
```

A3. Within and between beta-diversity per cluster
```{r}
#Used a bit of Python code to define, for each distance between 2
samples, which clusters they were part of.
dists = read.csv(file="qd_bray_curtis_distance_byclust.csv",
quote="")
dists$clust1vsclust2 <- factor(dists$clust1vsclust2, levels =
c("1vs1","1vs2","1vs3","1vs4","1vs5","1vs6","1vs7","1vs8","1vs9","1v
s10","2vs2","2vs3","2vs4","2vs5","2vs6",
"2vs7","2vs8","2vs9","2vs10","3vs3","3vs4","3vs5","3vs6","3vs7","3vs
8","3vs9","3vs10","4vs4",
"4vs5","4vs6","4vs7","4vs8","4vs9","4vs10","5vs5","5vs6","5vs7","5vs
8","5vs9","5vs10","6vs6","6vs7",
"6vs8","6vs9","6vs10","7vs7","7vs8","7vs9","7vs10","8vs8","8vs9","8v
s10","9vs9","9vs10","10vs10"))

#reorder
colour.array <- c("1vs1" = "black",
                  "1vs2" = "gray",
                  "1vs3" = "gray",
                  "1vs4" = "gray",
                  "1vs5" = "gray",
                  "1vs6" = "gray",
                  "1vs7" = "gray",
                  "1vs8" = "gray",
                  "1vs9" = "gray",
                  "1vs10" = "gray",
                  "2vs2" = "black",
                  "2vs3" = "gray",
                  "2vs4" = "gray",
                  "2vs5" = "gray",
                  "2vs6" = "gray",
                  "2vs7" = "gray",
                  "2vs8" = "gray",
                  "2vs9" = "gray",
                  "2vs10" = "gray",
                  "3vs3" = "black",
                  "3vs4" = "gray",
````

~~~
                   "3vs5" = "gray",
                   "3vs6" = "gray",
                   "3vs7" = "gray",
                   "3vs8" = "gray",
                   "3vs9" = "gray",
                   "3vs10" = "gray",
                   "4vs4" = "black",
                   "4vs5" = "gray",
                   "4vs6" = "gray",
                   "4vs7" = "gray",
                   "4vs8" = "gray",
                   "4vs9" = "gray",
                   "4vs10" = "gray",
                   "5vs5" = "black",
                   "5vs6" = "gray",
                   "5vs7" = "gray",
                   "5vs8" = "gray",
                   "5vs9" = "gray",
                   "5vs10" = "gray",
                   "6vs6" = "black",
                   "6vs7" = "gray",
                   "6vs8" = "gray",
                   "6vs9" = "gray",
                   "6vs10" = "gray",
                   "7vs7" = "black",
                   "7vs8" = "gray",
                   "7vs9" = "gray",
                   "7vs10" = "gray",
                   "8vs8" = "black",
                   "8vs9" = "gray",
                   "8vs10" = "gray",
                   "9vs9" = "black",
                   "9vs10" = "gray",
                   "10vs10" = "black")

p.wnbtw <- ggplot(data=dists, aes(x=clust1vsclust2, y=dist,
colour=clust1vsclust2)) +
  geom_boxplot() +
  scale_colour_manual(values=colour.array) +
  xlab("Pairwise comparison of clusters") + ylab("Bray curtis
distance metric") +
  theme(legend.position="none",
        axis.text.x = element_text(angle = 90, vjust = 0.5,
hjust=1))
p.wnbtw
```

A4. Beta-diversity as a heatmap
```{r}
betas <- as.data.frame(as.matrix(phyloseq::distance(qd.rare,
"bray")))
betas$x <- rownames(betas)
betas.melt <- melt(betas, variable="y")
betas.melt$x <- factor(betas.melt$x, levels = hc.order)
~~~

```
betas.melt$y <- factor(betas.melt$y, levels = hc.order)
head(betas.melt)
p.beta <- ggplot(betas.melt, aes(x=x, y=y, fill=value)) +
  geom_tile() +
  scale_fill_gradient(low="#636363", high="white") +
  theme(axis.text.x = element_blank(),
        axis.text.y = element_blank(),
        axis.title.x = element_blank(),
        axis.title.y = element_blank(),
        axis.ticks.x = element_blank(),
        axis.ticks.y = element_blank(),
        legend.position = "none")
p.beta
```

B. Draw taxa summary ordered by hierarchical clustering

```{r}
#Output taxa summary ordered by phylogeny
#Subset to genera > 0.1% abundance
L6.otu <-
subset(otu_table(qd.L6.norm),rowSums(otu_table(qd.L6.norm)) > 0.001)
#Merge OTU and TAX information
L6 <- merge(L6.otu, tax_table(qd.L6), by = "row.names", all.x =
TRUE)
#Set rownames to the Genus taxon assignments
rownames(L6) <- L6$Genus
L6[,1] <- NULL
#Transpose data.frame
L6.t <- t(L6)
#Mapfile read in
mapfile <- read.table("mapping_file.txt",
                         na.strings="#NULL!", header=TRUE,
fill=TRUE, sep="\t", row.names = 1)
#Ensure samples are ordered properly by mapfile
L6.map <- mapfile[rownames(L6.t),]
taxa <- data.frame(L6.map, L6.t)
L6.map <- subset(L6.map, rownames(L6.map) %in%
rownames(sample_data(qd)))
L6 <- subset(L6.t, rownames(L6.t) %in% rownames(L6.map))
#There are a few samples that aren't in the mapfile; pull them
L6.map <- subset(L6.map, rownames(L6.map) %in% rownames(L6))
taxa <- data.frame(L6.map, L6)
#Order rows in taxa by dendrogram
taxa <- taxa[match(hc.order, rownames(taxa)),]
#Reorder by most abundant taxa
x <- taxa[,114:length(taxa)]
#Make numeric
x[] <- lapply(x, as.character)
x[] <- lapply(x, as.numeric)
#Order by abundance
x <- x[,order(colSums(x),decreasing=TRUE)]
#Change any individual value < 1.0% to 0
new_x <- x
new_x[new_x<0.01] <- 0
```

```
#Remove any taxa that now sum to 0
new_x <- new_x[,colSums(new_x)>0]
#Add an Others column
new_x[,"Taxa < 1%"] <- 1-rowSums(new_x)
#Decide on colour vector
colours <- dada2_L6(colnames(new_x))
#Plot
df<-NULL
for (i in 1:dim(new_x)[2]){
  tmp<-
data.frame(row.names=NULL,Sample=rownames(new_x),Taxa=rep(colnames(n
ew_x)[i],dim(new_x)[1]),Value=new_x[,i])
  if(i==1){df<-tmp} else {df<-rbind(df,tmp)}
}
df$Sample <- factor(df$Sample, levels=unique(df$Sample))
df$Taxa <- factor(df$Taxa, levels=unique(df$Taxa))
taxa.plot <- ggplot(df,aes(Sample,Value,fill=Taxa))
+geom_bar(stat="identity") +
  scale_fill_manual(values=colours) +
  ylab("Relative abundance") +
  scale_y_reverse(expand = c(0,0)) +
  theme_bw() +
  guides(fill=guide_legend(ncol=7)) +
  theme(strip.background = element_rect(fill="gray85"),
        panel.margin = unit(0.5, "lines"),
        legend.position="none",
        legend.title=element_blank(),
        axis.text.x=element_blank(),
        axis.ticks.x=element_blank(),
        axis.title.x=element_blank())
```

B1. Draw taxa summary, highlighting the ASV-level diversity within Corynebacterium

```{r}
#Output taxa summary ordered by phylogeny
#Subset to genera > 0.1% abundance
L7.otu <- subset(otu_table(qd.norm),rowSums(otu_table(qd.norm)) >
0.001)
#Merge OTU and TAX information
L7 <- merge(L7.otu, tax_table(qd), by = "row.names", all.x = TRUE)
#Set rownames to the Genus taxon assignments
rownames(L7) <- paste(L7$Row.names,"_L6_",L7$Genus,sep="") # "_L5_",
L7[,1] <- NULL
#Transpose data.frame
L7.t <- t(L7)
#Mapfile read in
mapfile <- read.table("map_AllElderly-metadata-edit.txt",
                         na.strings="#NULL!", header=TRUE,
fill=TRUE, sep="\t", row.names = 1)
#Ensure samples are ordered properly by mapfile
L7.map <- mapfile[rownames(L7.t),]
taxa <- data.frame(L7.map, L7.t)
L7.map <- subset(L7.map, rownames(L7.map) %in%
```

```
rownames(sample_data(qd)))
L7 <- subset(L7.t, rownames(L7.t) %in% rownames(L7.map))
#There are a few samples that aren't in the mapfile; pull them
L7.map <- subset(L7.map, rownames(L7.map) %in% rownames(L7))
taxa <- data.frame(L7.map, L7)
#Order rows in taxa by dendrogram
taxa <- taxa[match(hc.order, rownames(taxa)),]
#Reorder by most abundant taxa
x <- taxa[,114:length(taxa)]
#Make numeric
x[] <- lapply(x, as.character)
x[] <- lapply(x, as.numeric)
#Order by abundance
x <- x[,order(colSums(x),decreasing=TRUE)]
#Change any individual value < 1.0% to 0
new_x <- x
new_x[new_x<0.01] <- 0
#Remove any taxa that now sum to 0
new_x <- new_x[,colSums(new_x)>0]
#Add an Others column
new_x[,"Taxa < 1%"] <- 1-rowSums(new_x)
#Decide on colour vector
colours <- dada2_L7_Coryne(colnames(new_x))
#Plot
df<-NULL
for (i in 1:dim(new_x)[2]){
  tmp<-
data.frame(row.names=NULL,Sample=rownames(new_x),Taxa=rep(colnames(n
ew_x)[i],dim(new_x)[1]),Value=new_x[,i])
  if(i==1){df<-tmp} else {df<-rbind(df,tmp)}
}
df$Sample <- factor(df$Sample, levels=unique(df$Sample))
df$Taxa <- factor(df$Taxa, levels=unique(df$Taxa))
taxa.plot.Coryne <- ggplot(df,aes(Sample,Value,fill=Taxa))
+geom_bar(stat="identity") +
  scale_fill_manual(values=colours) +
  ylab("Proportions") +
  scale_y_reverse(expand = c(0,0)) +
  theme_bw() +
  theme(strip.background = element_rect(fill="gray85"),
        panel.margin = unit(0.5, "lines"),
        legend.position="none",
        axis.text.x=element_blank(),
        axis.ticks.x=element_blank(),
        axis.title.x=element_blank())
taxa.plot.Coryne
```

B1. Draw taxa summary, highlighting the ASV-level diversity within Moraxella

```{r}
#Output taxa summary ordered by phylogeny
#Subset to genera > 0.1% abundance
L7.otu <- subset(otu_table(qd.norm),rowSums(otu_table(qd.norm)) >
```

```
0.001)
#Merge OTU and TAX information
L7 <- merge(L7.otu, tax_table(qd), by = "row.names", all.x = TRUE)
#Set rownames to the Genus taxon assignments
rownames(L7) <- paste(L7$Row.names,"_L6_",L7$Genus,sep="") # "_L5_",
L7[,1] <- NULL
#Transpose data.frame
L7.t <- t(L7)
#Mapfile read in
mapfile <- read.table("map_AllElderly-metadata-edit.txt",
                        na.strings="#NULL!", header=TRUE,
fill=TRUE, sep="\t", row.names = 1)
#Ensure samples are ordered properly by mapfile
L7.map <- mapfile[rownames(L7.t),]
taxa <- data.frame(L7.map, L7.t)
L7.map <- subset(L7.map, rownames(L7.map) %in%
rownames(sample_data(qd)))
L7 <- subset(L7.t, rownames(L7.t) %in% rownames(L7.map))
#There are a few samples that aren't in the mapfile; pull them
L7.map <- subset(L7.map, rownames(L7.map) %in% rownames(L7))
taxa <- data.frame(L7.map, L7)
#Order rows in taxa by dendrogram
taxa <- taxa[match(hc.order, rownames(taxa)),]
#Reorder by most abundant taxa
x <- taxa[,114:length(taxa)]
#Make numeric
x[] <- lapply(x, as.character)
x[] <- lapply(x, as.numeric)
#Order by abundance
x <- x[,order(colSums(x),decreasing=TRUE)]
#Change any individual value < 1.0% to 0
new_x <- x
new_x[new_x<0.01] <- 0
#Remove any taxa that now sum to 0
new_x <- new_x[,colSums(new_x)>0]
#Add an Others column
new_x[,"Taxa < 1%"] <- 1-rowSums(new_x)
#Decide on colour vector
colours <- dada2_L7_Morax(colnames(new_x))
#Plot
df<-NULL
for (i in 1:dim(new_x)[2]){
  tmp<-
data.frame(row.names=NULL,Sample=rownames(new_x),Taxa=rep(colnames(n
ew_x)[i],dim(new_x)[1]),Value=new_x[,i])
  if(i==1){df<-tmp} else {df<-rbind(df,tmp)}
}
df$Sample <- factor(df$Sample, levels=unique(df$Sample))
df$Taxa <- factor(df$Taxa, levels=unique(df$Taxa))
taxa.plot.Morax <- ggplot(df,aes(Sample,Value,fill=Taxa))
+geom_bar(stat="identity") +
  scale_fill_manual(values=colours) +
  ylab("Proportions") +
  scale_y_reverse(expand = c(0,0)) +
```

````
  theme_bw() +
  theme(strip.background = element_rect(fill="gray85"),
        panel.margin = unit(0.5, "lines"),
        legend.position="none",
        axis.text.x=element_blank(),
        axis.ticks.x=element_blank(),
        axis.title.x=element_blank())
taxa.plot.Morax
```

C. Alpha diversity metric (& statistics)
```{r}
#Calculate Shannon diversity per sample
alpha = estimate_richness(qd, measures=c("Shannon"))
alpha$SampleID <- rownames(alpha)
#Add clustering data
alpha <- merge(alpha, clustdf, by.x="SampleID", by.y="SampleID")
#Order rows in taxa by dendrogram
alpha.or <- alpha[match(hc.order, alpha$SampleID),]
alpha.or$SampleID <- factor(alpha.or$SampleID, levels =
alpha.or$SampleID)
#Plot
plot.alpha <- ggplot(data=alpha.or, aes(x=SampleID, y=Shannon,
colour=cluster)) +
  geom_point() +
  theme_classic() +
  theme(axis.text.x = element_blank(),
        axis.ticks.x= element_blank(),
        axis.title.x= element_blank(),
        legend.position="none") +
  scale_colour_manual(values=cluster.colours.k10)
plot.alpha

#Significant?
leveneTest(Shannon ~ as.factor(cluster), data=alpha)

plot.alpha.box <- ggplot(data=alpha, aes(x=cluster, y=Shannon,
colour=cluster, group=cluster)) +
  geom_boxplot() + geom_point(position="jitter", alpha=0.2) +
  xlab("Cluster #") + ggtitle("p=0.04962*") +
  theme_bw() + theme(legend.position="none") +
  scale_colour_manual(values=cluster.colours.k10)
plot.alpha.box
```

D1. Stats as to the num of genera present, above different rel
abunds, per sample etc.
```{r}
qd.L6
otus <- as.data.frame(otu_table(qd.L6.norm))
colnames(otus)
otus$rowSum <- rowSums(otus)
head(otus$rowSum)
tail(otus$rowSum)
````

```
#Number of non-zero genera present
outs.sub <- subset(otus, otus$rowSum>0)
nrow(outs.sub)
#Number of genera present with a cumulative relative abundance of
>=0.01%
outs.sub <- subset(otus, otus$rowSum>=0.0001)
nrow(outs.sub)
#Number of genera which are >=1% rel abund in at least one sample
otus <- as.data.frame(otu_table(qd.L6.norm))
otus$maxAbund <- apply(otus, 1, max)
head(otus$maxAbund)
outs.sub <- subset(otus, otus$maxAbund>=0.01)
nrow(outs.sub)
#Median number of non-zero genera present per sample
otus <- as.data.frame(otu_table(qd.L6.norm))
otu.t <- as.data.frame(t(otus))
otu.t$countnozeroes <- apply(otu.t, 1, function(r)sum(r!=0))
median(otu.t$countnozeroes)
min(otu.t$countnozeroes)
max(otu.t$countnozeroes)
sd(otu.t$countnozeroes)
```

D. Mean relative abundance of top taxa per cluster

```{r}
#Abundance of top 10 taxa across 10 clusters
L6.otu <- otu_table(qd.L6.norm)
L6 <- merge(L6.otu, tax_table(qd.L6), by = "row.names", all.x =
TRUE)
rownames(L6) <- L6$Genus
L6[,1] <- NULL #remove ASV DNA string
L6 <- L6[1:(length(L6)-6)] #remove KPCOFGenus info
#identify most prevalant taxa
L6.sums <- rowSums(L6)
L6.sums <- sort(L6.sums, decreasing = TRUE)
head(L6.sums)
L6.t <- as.data.frame(t(L6))
L6.t$SampleID <- rownames(L6.t)
L6.top <- L6.t[,colnames(L6.t) %in% c("Corynebacterium_1",
"Moraxella", "Staphylococcus",  "Dolosigranulum")]
#Add cluster information to L6.top
L6.top$SampleID <- rownames(L6.top)
L6.top.clust <- merge(L6.top, clustdf, by.x="SampleID",
by.y="SampleID")
L6.top.clust$SampleID <- NULL
L6.top.long <- melt(L6.top.clust, id.vars = "cluster")
L6.top.long$value <- as.numeric(as.character(L6.top.long$value))
L6.top.long$variable <- factor(L6.top.long$variable,
levels=c("Corynebacterium_1", "Moraxella", "Staphylococcus",
"Dolosigranulum", "Streptococcus","Mycoplasma"))
p.top <- ggplot(L6.top.long, aes(x = cluster, y = value, colour =
variable, fill = variable)) +
  geom_boxplot(alpha=0.2) +
  scale_colour_manual(values=c("Streptococcus" = "#C7006F", #pink
```

```
                             "Staphylococcus" = "#875073", #purple
                             "Moraxella" = "#005B9E", #dark blue
                             "Mycoplasma" = "#7A7800", #puke
                             "Dolosigranulum" = "#7EB5FF", #baby
blue
                             "Corynebacterium_1" = "#d15919")) +
#orange
  scale_fill_manual(values=c("Streptococcus" = "#C7006F", #pink
                             "Staphylococcus" = "#875073", #purple
                             "Moraxella" = "#005B9E", #dark blue
                             "Mycoplasma" = "#7A7800", #puke
                             "Dolosigranulum" = "#7EB5FF", #baby
blue
                             "Corynebacterium_1" = "#d15919")) +
#orange
  theme_bw() +
  theme(legend.position = "none") +
        #legend.title = element_blank()) +
  xlab("Cluster #") + ylab("Relative abundance")
p.top
```

D1. Dominant taxa per cluster

```{r}
# >50% relative abundance in >90% of samples
L6.otu <- otu_table(qd.L6.norm)
L6 <- merge(L6.otu, tax_table(qd.L6), by = "row.names", all.x =
TRUE)
rownames(L6) <- L6$Genus
L6[,1] <- NULL #remove ASV DNA string
L6 <- L6[1:(length(L6)-6)] #remove KPCOFGenus info
#Add cluster information
L6 <- as.data.frame(t(L6))
L6$SampleID <- rownames(L6)
L6.clust <- merge(L6, clustdf, by.x="SampleID", by.y="SampleID")
L6.clust$SampleID <- NULL
#Subset out each
L6.clust1 <- subset(L6.clust, L6.clust$cluster=="1")
L6.clust2 <- subset(L6.clust, L6.clust$cluster=="2")
L6.clust3 <- subset(L6.clust, L6.clust$cluster=="3")
L6.clust4 <- subset(L6.clust, L6.clust$cluster=="4")
L6.clust5 <- subset(L6.clust, L6.clust$cluster=="5")
L6.clust6 <- subset(L6.clust, L6.clust$cluster=="6")
L6.clust7 <- subset(L6.clust, L6.clust$cluster=="7")
L6.clust8 <- subset(L6.clust, L6.clust$cluster=="8")
L6.clust9 <- subset(L6.clust, L6.clust$cluster=="9")
sum(nrow(L6.clust1),
    nrow(L6.clust2),
    nrow(L6.clust3),
    nrow(L6.clust4),
    nrow(L6.clust5),
    nrow(L6.clust6),
    nrow(L6.clust7),
    nrow(L6.clust8),
```

```
    nrow(L6.clust9)) #check
#cluster1
L6.clust1 <- as.data.frame(t(L6.clust1))
L6.clust1$over50 <- apply(L6.clust1, 1, function(r)
(sum(as.numeric(r)>0.35)))
head(L6.clust1)
which(L6.clust1$over50>0.7*(ncol(L6.clust1)-1))
L6.clust1[697,] #Corynebacterium
L6.clust1[991,]
#cluster2
L6.clust2 <- as.data.frame(t(L6.clust2))
L6.clust2$over50 <- apply(L6.clust2, 1, function(r)
(sum(as.numeric(r)>0.35)))
head(L6.clust2)
which(L6.clust2$over50>0.7*(ncol(L6.clust2)-1))
#none
#cluster3
L6.clust3 <- as.data.frame(t(L6.clust3))
L6.clust3$over50 <- apply(L6.clust3, 1, function(r)
(sum(as.numeric(r)>0.35)))
head(L6.clust3)
which(L6.clust3$over50>0.7*(ncol(L6.clust3)-1))
L6.clust1[697,] #Corynebacterium
#cluster4
L6.clust4 <- as.data.frame(t(L6.clust4))
L6.clust4$over50 <- apply(L6.clust4, 1, function(r)
(sum(as.numeric(r)>0.35)))
head(L6.clust4)
which(L6.clust4$over50>0.7*(ncol(L6.clust4)-1))
L6.clust4[772,] #Dolosig
#cluster5
L6.clust5 <- as.data.frame(t(L6.clust5))
L6.clust5$over50 <- apply(L6.clust5, 1, function(r)
(sum(as.numeric(r)>0.35)))
head(L6.clust5)
which(L6.clust5$over50>0.7*(ncol(L6.clust5)-1))
L6.clust5[18,] #Moraxella
#cluster6
L6.clust6 <- as.data.frame(t(L6.clust6))
L6.clust6$over50 <- apply(L6.clust6, 1, function(r)
(sum(as.numeric(r)>0.35)))
head(L6.clust6)
which(L6.clust6$over50>0.7*(ncol(L6.clust6)-1))
L6.clust6[871,] #Staphylococcus
#cluster7
L6.clust7 <- as.data.frame(t(L6.clust7))
L6.clust7$over50 <- apply(L6.clust7, 1, function(r)
(sum(as.numeric(r)>0.35)))
head(L6.clust7)
which(L6.clust7$over50>0.7*(ncol(L6.clust7)-1))
L6.clust7[18,] #Moraxella
#cluster8
L6.clust8 <- as.data.frame(t(L6.clust8))
L6.clust8$over50 <- apply(L6.clust8, 1, function(r)
```

```
(sum(as.numeric(r)>0.35)))
head(L6.clust8)
which(L6.clust8$over50>0.7*(ncol(L6.clust8)-1))
L6.clust8[18,] #Moraxella
#cluster9
L6.clust9 <- as.data.frame(t(L6.clust9))
L6.clust9$over50 <- apply(L6.clust9, 1, function(r)
(sum(as.numeric(r)>0.35)))
head(L6.clust9)
which(L6.clust9$over50>0.7*(ncol(L6.clust9)-1))
L6.clust9[697,] #Corynebacterium
```

D1. Prevalence vs. abundance of top taxa

```{r}
##Identify the most abundant taxa (i.e., taxa with the highest mean
abundances)
L6.abund <- rowMeans(as.matrix(L6))
names(L6.abund) <- rownames(L6)
L6.abund <- sort(L6.abund, decreasing = TRUE)
head(L6.abund)

L6.max <- rowMax(as.matrix(L6))
names(L6.max) <- rownames(L6)
L6.max["Dolosigranulum"]
L6.max["Moraxella"]
L6.max["Staphylococcus"]
min(L6["Dolosigranulum",][L6["Dolosigranulum",] > 0])*100
min(L6["Staphylococcus",][L6["Staphylococcus",] > 0])*100
min(L6["Moraxella",][L6["Moraxella",] > 0])*100
#How many samples contain Corynebacterium_1
apply(L6["Corynebacterium_1",], 1, function(r)sum(r!=0))
min(L6["Corynebacterium_1",][L6["Corynebacterium_1",] > 0])
max(L6["Corynebacterium_1",])
#5 taxa present with mean abund >1%
L6.abund.t5 <- subset(L6, rownames(L6) %in% names(L6.abund[1:4])) #5
head(L6.abund.t5)
L6.abund.t5$genus <- rownames(L6.abund.t5)
L6.abund.t5.long <- melt(L6.abund.t5, id.vars = "genus")
L6.abund.t5.long$genus <- factor(L6.abund.t5.long$genus,
levels=names(L6.abund[1:5]))
p.abund <- ggplot(L6.abund.t5.long, aes(x=genus, y=value,
fill=genus)) +
  geom_boxplot(alpha=0.8) +
  geom_point(alpha=0.05, position="jitter") +
  scale_fill_manual(values = c("Corynebacterium_1" = "#d15919",
                              "Staphylococcus" = "#875073",
                              "Moraxella" = "#005b9e",
                              "Dolosigranulum" = "#7eb5ff")) +
  xlab("Taxa with a mean relative abundance >1%") +
  ylab("Relative abundance") +
  theme(legend.position = "none")
p.abund
```

D2. Mean Bray Curtis distance across the dataset
```{r}
## Bray Curtis distance
mean(dist1)
median(dist1)
dist.df <- as.data.frame(as.character(dist1))
head(dist.df)
dist.df$all <- "all"
dist.df$values <- dist.df$`as.character(dist1)`
dist.df$values <- as.numeric(dist.df$values)
dist.df$`as.character(dist1)`<- NULL
plot.bray <- ggplot(data=dist.df, aes(x=all, y=values)) +
  geom_boxplot(position = position_nudge(x = -0.4, y = 0), width =
0.2, outlier.shape = NA, alpha = 0.4) +
  geom_violin(position = position_nudge(x = +0.2, y = 0), alpha =
0.8, fill="gray") +
  theme(axis.text.x = element_blank()) +
  xlab("All samples") + ylab("Bray Curtis dissimilarity metric")
plot.bray
```

F. Number of samples per cluster
```{r}
p.counts <- ggplot(clustdf, aes(x=cluster, fill=cluster)) +
  geom_bar() + #position="identity", binwidth=0.5) + #,
aes(fill=cluster)) +
  scale_fill_manual(values=cluster.colours.k10) +
  theme_bw() +
  theme(legend.position="none") +
  scale_y_continuous(expand=c(0,0)) +
  xlab("Cluster #") + ylab("# of Samples per cluster")
p.counts

table(clustdf$cluster)
median(table(clustdf$cluster))
min(table(clustdf$cluster))
max(table(clustdf$cluster))
```

G. Is there a difference in qPCR load per cluster?
```{r}
#Significant?
leveneTest(qPCR_Concentration ~ as.factor(cluster),
data=qd.clust.sam) #p=0.08108

#Alter the qPCR concentration (ng) to copies of 16S gene
head(qd.clust.sam)
qd.clust.sam$qPCR_adjust <- qd.clust.sam$qPCR_Concentration
qd.clust.sam$qPCR_adjust <- qd.clust.sam$qPCR_adjust/1000000000
qd.clust.sam$qPCR_adjust <- qd.clust.sam$qPCR_adjust/3063490320
qd.clust.sam$qPCR_adjust <- qd.clust.sam$qPCR_adjust*6.0223*10^23
qd.clust.sam$qPCR_adjust <- qd.clust.sam$qPCR_adjust*7
qPCR.Cluster <- ggplot(qd.clust.sam, aes(x=cluster, y=qPCR_adjust,
```

````
group=cluster, colour=cluster)) +
  geom_boxplot() +
  ggtitle("p=0.08108") +
  geom_jitter(alpha=0.2) +
  scale_y_log10() +
  xlab("Cluster #") +
  ylab("# of 16S rRNA gene copies per sample") +
  scale_colour_manual(values=cluster.colours.k10) +
  theme_bw() +
  theme(legend.position = "none")
qPCR.Cluster
```

Patchwork a Figure together
```{r}
### Put Figure together ###
layout = '
AAAAAAAA
BBBBBBBB
CCDDDEFF
GGGGGGGG
HHHHHHHH
IIIJJJKK
'

patchwork.Fig1 <-
  p.tree + #A
  taxa.plot + #B
  p.abund +
  p.top + #E
  plot.bray +
  clust.pcoa12 + #C
  taxa.plot.Coryne +
  taxa.plot.Morax +
  plot.alpha.box + #F
  qPCR.Cluster + #G
  p.counts + #D
  plot_layout(design = layout) +
  plot_annotation(tag_levels = 'A')
patchwork.Fig1
pdf("figures/Figure1.pdf", height=15, width=16)
print(patchwork.Fig1)
dev.off()

### Put Figure together ###
layout = '
AAAA
BBBB
CCCC
CCCC
CCCC
DEFG
HHHI
'
````

```
patchwork.Fig1 <- p.tree + #A
  taxa.plot + #B
  p.beta + #C
  clust.pcoa12 +
  clust.pcoa13 +
  clust.pcoa23 +
  clust.pcoa14 +
  p.wnbtw +
  plot_spacer() +
  plot_layout(design = layout) +
  plot_annotation(tag_levels = 'A')
patchwork.Fig1
pdf("figures/SupFigure2.pdf", height=20, width=16) #-Dolo
print(patchwork.Fig1)
dev.off()
```

Supplemental Table 1: Metadata vars correlating with mb (qd.baseline & qd.healthy)

1. Subset metadata vars which we will test for significance based on the following criteria:
(a) for any variable, there must be <=15% NAs (treat Unknowns as NAs)
(b) for binary variables, there must be >=10% variation
(c) for multi-value discrete variables, any variable accounting for <=3% variation is omitted
(d) include all continuous variables

```{r, include=FALSE}
qd.baseline.stats = c()
table(sample_data(qd.baseline)$SampleID) #unique ID variable; omit from stats
table(sample_data(qd.baseline)$SampleID.1) #unique ID variable; omit from stats
table(sample_data(qd.baseline)$Study.ID) #unique ID variable; omit from stats
table(sample_data(qd.baseline)$ID) #unique ID variable; omit from stats
table(sample_data(qd.baseline)$Initials) #unique ID variable; omit from stats
table(sample_data(qd.baseline)$DOB.Y.M.D.) #unique ID variable; omit from stats
table(sample_data(qd.baseline)$Age_at_enrollment) #continuous, include
qd.baseline.stats = c(qd.baseline.stats, "Age_at_enrollment")
table(sample_data(qd.baseline)$Sex) #Male
qd.baseline.stats = c(qd.baseline.stats, "Sex")
table(sample_data(qd.baseline)$Site)
qd.baseline.stats = c(qd.baseline.stats, "Site")
table(sample_data(qd.baseline)$Month) #omit Feb and Mar
qd.baseline.stats = c(qd.baseline.stats, "Month")
table(sample_data(qd.baseline)$Season) #omit Spring
```

```
qd.baseline.stats = c(qd.baseline.stats, "Season")
table(sample_data(qd.baseline)$Year) #continuous, include
qd.baseline.stats = c(qd.baseline.stats, "Year")
table(sample_data(qd.baseline)$Completed.Study) #FALSE, include
qd.baseline.stats = c(qd.baseline.stats, "Completed.Study")
table(sample_data(qd.baseline)$Allocation_Group_Probiotics)
#placebo, include
qd.baseline.stats = c(qd.baseline.stats,
"Allocation_Group_Probiotics")
table(sample_data(qd.baseline)$Type.B.E.F.) #unique ID variable;
omit from stats
table(sample_data(qd.baseline)$hadEvent) #T
qd.baseline.stats = c(qd.baseline.stats, "hadEvent")
table(sample_data(qd.baseline)$Smoker) #Yes is <3%, omit Yes
qd.baseline.stats = c(qd.baseline.stats, "Smoker")
table(sample_data(qd.baseline)$Num_Medications) #continuous
variable, include
qd.baseline.stats = c(qd.baseline.stats, "Num_Medications")
table(sample_data(qd.baseline)$Influenza_vacc_this_season) #No, omit
qd.baseline.stats = c(qd.baseline.stats,
"Influenza_vacc_this_season")
table(sample_data(qd.baseline)
$Influenza_seasonal_vaccine_last_season) #No, include
qd.baseline.stats = c(qd.baseline.stats,
"Influenza_seasonal_vaccine_last_season")
table(sample_data(qd.baseline)$Influenza_vaccine_ever) #No, omit
qd.baseline.stats = c(qd.baseline.stats, "Influenza_vaccine_ever")
table(sample_data(qd.baseline)
$Did_the_Pt_have_lab_confirmed_infu_last_season) #omit
table(sample_data(qd.baseline)$Has_pt_received_pneumonia_vaccine)
#No include
qd.baseline.stats = c(qd.baseline.stats,
"Has_pt_received_pneumonia_vaccine")
table(sample_data(qd.baseline)$If_pneumovax_within_5_yrs) #unknown,
omit
table(sample_data(qd.baseline)$Is_pt_in_shared_room) #Yes, include
qd.baseline.stats = c(qd.baseline.stats, "Is_pt_in_shared_room")
table(sample_data(qd.baseline)$If_shared_room_how_many) #unknown,
omit
table(sample_data(qd.baseline)$Barthel_Total) #continuous variable,
include
qd.baseline.stats = c(qd.baseline.stats, "Barthel_Total")
table(sample_data(qd.baseline)$COPD) #Yes, include
qd.baseline.stats = c(qd.baseline.stats, "COPD")
table(sample_data(qd.baseline)$CHF) #Yes, include
qd.baseline.stats = c(qd.baseline.stats, "CHF")
table(sample_data(qd.baseline)$CAD) #CAD, include
qd.baseline.stats = c(qd.baseline.stats, "CAD")
table(sample_data(qd.baseline)$Asthma) #Yes, omit
table(sample_data(qd.baseline)$Anemia) #Yes, include
qd.baseline.stats = c(qd.baseline.stats, "Anemia")
table(sample_data(qd.baseline)$Dementia) #No, include
qd.baseline.stats = c(qd.baseline.stats, "Dementia")
table(sample_data(qd.baseline)$CVA.Stroke) #Yes, include; omit Prior
```

```
qd.baseline.stats = c(qd.baseline.stats, "CVA.Stroke")
table(sample_data(qd.baseline)$DM) #Yes, include
qd.baseline.stats = c(qd.baseline.stats, "DM")
table(sample_data(qd.baseline)$Hypothyroid) #Yes, include
qd.baseline.stats = c(qd.baseline.stats, "Hypothyroid")
table(sample_data(qd.baseline)$Num_Comorbidities) #continuous
variable, include
qd.baseline.stats = c(qd.baseline.stats, "Num_Comorbidities")
table(sample_data(qd.baseline)$Seizures) #Yes, omit
qd.baseline.stats = c(qd.baseline.stats, "Seizures")
table(sample_data(qd.baseline)$SYes_S3_Arrhythmia) #Yes, omit
table(sample_data(qd.baseline)$Hyperthyroid) #Yes, omit
table(sample_data(qd.baseline)$CRF) #Yes, omit
table(sample_data(qd.baseline)$Liver_dz) #No, omit
table(sample_data(qd.baseline)$Cancer) #Yes, omit
qd.baseline.stats = c(qd.baseline.stats, "Cancer")
table(sample_data(qd.baseline)$IL1B)
qd.baseline.stats = c(qd.baseline.stats, "IL1B")
table(sample_data(qd.baseline)$IL6)
qd.baseline.stats = c(qd.baseline.stats, "IL6")
table(sample_data(qd.baseline)$TNFA)
qd.baseline.stats = c(qd.baseline.stats, "TNFA")
```

2. Identify correlations between metadata vars which pass the above criteria using the chi-squared test.
When p.value < 0.05, we reject the null hypothesis that the 2 variables tested are independent.
When p.value >= 0.05, we accept the null hypothesis that the 2 variables are independent.

```{r, include=FALSE}
#Start
baseline.alpha = estimate_richness(qd.baseline,
measures=c("Shannon"))
baseline.alpha$SampleID = rownames(baseline.alpha)
baseline.test = sample_data(qd.baseline)
baseline.test = cbind(baseline.alpha, baseline.test)

#Age_at_enrollment
summary(aov(Age_at_enrollment ~ as.factor(Sex), data=baseline.test))
###Significant, so:###
a1 <- aov(Age_at_enrollment ~ as.factor(Sex), data=baseline.test)
nd = data.frame(Sex = unique(baseline.test$Sex))
pred = predict(a1, newdata = nd, se.fit = TRUE)
pred
nd$Pred = pred$fit # adds the predicted group means to the data
frame
nd$Lo = nd$Pred - 1.96*pred$se.fit # lower bound of the confidence
interval by subtracting 1.96*se from the prediction
nd$Hi = nd$Pred + 1.96*pred$se.fit # upper bound ditto
plt = ggplot(nd, aes(x = Sex)) +
  geom_point(aes(y = Pred), size=4) +
  geom_linerange(aes(ymin = Lo, ymax = Hi), alpha=0.5) +
  theme(axis.text.x=element_text(angle=90,hjust=1,vjust=0.5))
```

```
plt
#Don't need to do a posthoc test; only 2 categories
###
summary(aov(Age_at_enrollment ~ as.factor(Site),
data=baseline.test))
summary(aov(Age_at_enrollment ~ as.factor(Month),
data=baseline.test))
summary(aov(Age_at_enrollment ~ as.factor(Season),
data=baseline.test))
summary(aov(Age_at_enrollment ~ as.factor(Year),
data=baseline.test))
summary(aov(Age_at_enrollment ~ as.factor(Completed.Study),
data=baseline.test))
summary(aov(Age_at_enrollment ~
as.factor(Allocation_Group_Probiotics), data=baseline.test))
summary(aov(Age_at_enrollment ~ as.factor(hadEvent),
data=baseline.test))
kruskal.test(baseline.test$Age_at_enrollment,
baseline.test$Num_Medications)
summary(aov(Age_at_enrollment ~
as.factor(Influenza_vacc_this_season), data=baseline.test))
summary(aov(Age_at_enrollment ~
as.factor(Influenza_seasonal_vaccine_last_season),
data=baseline.test))
summary(aov(Age_at_enrollment ~ as.factor(Influenza_vaccine_ever),
data=baseline.test))
summary(aov(Age_at_enrollment ~
as.factor(Has_pt_received_pneumonia_vaccine), data=baseline.test))
summary(aov(Age_at_enrollment ~ as.factor(Is_pt_in_shared_room),
data=baseline.test))
kruskal.test(baseline.test$Age_at_enrollment,
baseline.test$Barthel_Total)
summary(aov(Age_at_enrollment ~ as.factor(COPD),
data=baseline.test))
summary(aov(Age_at_enrollment ~ as.factor(CHF), data=baseline.test))
summary(aov(Age_at_enrollment ~ as.factor(CAD), data=baseline.test))
summary(aov(Age_at_enrollment ~ as.factor(Anemia),
data=baseline.test))
###Significant, so:###
a1 <- aov(Age_at_enrollment ~ as.factor(Anemia), data=baseline.test)
nd = data.frame(Anemia = unique(baseline.test$Anemia))
pred = predict(a1, newdata = nd, se.fit = TRUE)
pred
nd$Pred = pred$fit # adds the predicted group means to the data
frame
nd$Lo = nd$Pred - 1.96*pred$se.fit # lower bound of the confidence
interval by subtracting 1.96*se from the prediction
nd$Hi = nd$Pred + 1.96*pred$se.fit # upper bound ditto
plt = ggplot(nd, aes(x = Anemia)) +
  geom_point(aes(y = Pred), size=4) +
  geom_linerange(aes(ymin = Lo, ymax = Hi), alpha=0.5) +
  theme(axis.text.x=element_text(angle=90,hjust=1,vjust=0.5))
plt
#Don't need to do a posthoc test; only 2 categories
```

```
###
summary(aov(Age_at_enrollment ~ as.factor(Dementia),
data=baseline.test))
summary(aov(Age_at_enrollment ~ as.factor(CVA.Stroke),
data=baseline.test))
summary(aov(Age_at_enrollment ~ as.factor(DM), data=baseline.test))
summary(aov(Age_at_enrollment ~ as.factor(Hypothyroid),
data=baseline.test))
kruskal.test(baseline.test$Age_at_enrollment,
baseline.test$Num_Comorbidities)
summary(aov(Age_at_enrollment ~ as.factor(Seizures),
data=baseline.test))
###Significant, so:###
a1 <- aov(Age_at_enrollment ~ as.factor(Seizures),
data=baseline.test)
nd = data.frame(Seizures = unique(baseline.test$Seizures))
pred = predict(a1, newdata = nd, se.fit = TRUE)
pred
nd$Pred = pred$fit # adds the predicted group means to the data
frame
nd$Lo = nd$Pred - 1.96*pred$se.fit # lower bound of the confidence
interval by subtracting 1.96*se from the prediction
nd$Hi = nd$Pred + 1.96*pred$se.fit # upper bound ditto
plt = ggplot(nd, aes(x = Seizures)) +
  geom_point(aes(y = Pred), size=4) +
  geom_linerange(aes(ymin = Lo, ymax = Hi), alpha=0.5) +
  theme(axis.text.x=element_text(angle=90,hjust=1,vjust=0.5))
plt
#Don't need to do a posthoc test; only 2 categories
###
summary(aov(Age_at_enrollment ~ as.factor(Cancer),
data=baseline.test))
kruskal.test(baseline.test$Age_at_enrollment, baseline.test$IL1B)
kruskal.test(baseline.test$Age_at_enrollment, baseline.test$IL6)
kruskal.test(baseline.test$Age_at_enrollment, baseline.test$TNFA)
kruskal.test(baseline.test$Age_at_enrollment,
baseline.test$qPCR_Concentration)

#Sex
chisq.test(sample_data(qd.baseline)$Sex, sample_data(qd.baseline)
$Site)
chisq.test(sample_data(qd.baseline)$Sex, sample_data(qd.baseline)
$Month)
chisq.test(sample_data(qd.baseline)$Sex, sample_data(qd.baseline)
$Season)
chisq.test(sample_data(qd.baseline)$Sex, sample_data(qd.baseline)
$Year)
chisq.test(sample_data(qd.baseline)$Sex, sample_data(qd.baseline)
$Completed.Study)
chisq.test(sample_data(qd.baseline)$Sex, sample_data(qd.baseline)
$Allocation_Group_Probiotics)
chisq.test(sample_data(qd.baseline)$Sex, sample_data(qd.baseline)
$hadEvent)
summary(aov(Num_Medications ~ as.factor(Sex), data=baseline.test))
```

```
chisq.test(sample_data(qd.baseline)$Sex, sample_data(qd.baseline)
$Influenza_vacc_this_season)
chisq.test(sample_data(qd.baseline)$Sex, sample_data(qd.baseline)
$Influenza_seasonal_vaccine_last_season)
chisq.test(sample_data(qd.baseline)$Sex, sample_data(qd.baseline)
$Influenza_vaccine_ever)
chisq.test(sample_data(qd.baseline)$Sex, sample_data(qd.baseline)
$Has_pt_received_pneumonia_vaccine)
chisq.test(sample_data(qd.baseline)$Sex, sample_data(qd.baseline)
$Is_pt_in_shared_room)
summary(aov(Barthel_Total ~ as.factor(Sex), data=baseline.test))
chisq.test(sample_data(qd.baseline)$Sex, sample_data(qd.baseline)
$COPD)
chisq.test(sample_data(qd.baseline)$Sex, sample_data(qd.baseline)
$CHF)
chisq.test(sample_data(qd.baseline)$Sex, sample_data(qd.baseline)
$CAD)
chisq.test(sample_data(qd.baseline)$Sex, sample_data(qd.baseline)
$Anemia)
chisq.test(sample_data(qd.baseline)$Sex, sample_data(qd.baseline)
$Dementia)
chisq.test(sample_data(qd.baseline)$Sex, sample_data(qd.baseline)
$CVA.Stroke)
chisq.test(sample_data(qd.baseline)$Sex, sample_data(qd.baseline)
$DM)
chisq.test(sample_data(qd.baseline)$Sex, sample_data(qd.baseline)
$Hypothyroid)
summary(aov(Num_Comorbidities ~ as.factor(Sex), data=baseline.test))
chisq.test(sample_data(qd.baseline)$Sex, sample_data(qd.baseline)
$Seizures)
chisq.test(sample_data(qd.baseline)$Sex, sample_data(qd.baseline)
$Cancer)
summary(aov(as.numeric(IL1B) ~ as.factor(Sex), data=baseline.test))
summary(aov(as.numeric(IL6) ~ as.factor(Sex), data=baseline.test))
summary(aov(as.numeric(TNFA) ~ as.factor(Sex), data=baseline.test))
summary(aov(as.numeric(qPCR_Concentration) ~ as.factor(Sex),
data=baseline.test))

#Site; #omit
qd.baseline.Test = subset_samples(qd.baseline, (Site != "" | Site !=
"")) #names removed for GDPR reasons
baseline.test.sup = subset(baseline.test, (baseline.test$Site != ""
& baseline.test$Site != "")) #ditto

chisq <- chisq.test(sample_data(qd.baseline.Test)$Site,
sample_data(qd.baseline.Test)$Month)
chisq
##Significant
corrplot(chisq$residuals, is.cor = FALSE)
##
chisq <- chisq.test(sample_data(qd.baseline.Test)$Site,
sample_data(qd.baseline.Test)$Season)
chisq
##Significant
```

```
corrplot(chisq$residuals, is.cor = FALSE)
##
chisq <- chisq.test(sample_data(qd.baseline.Test)$Site,
sample_data(qd.baseline.Test)$Year)
chisq
##Significant
corrplot(chisq$residuals, is.cor = FALSE)
##
chisq.test(sample_data(qd.baseline.Test)$Site,
sample_data(qd.baseline.Test)$Completed.Study)
chisq.test(sample_data(qd.baseline.Test)$Site,
sample_data(qd.baseline.Test)$Allocation_Group_Probiotics)
chisq.test(sample_data(qd.baseline.Test)$Site,
sample_data(qd.baseline.Test)$hadEvent)
summary(aov(Num_Medications ~ as.factor(Site),
data=baseline.test.sup))
chisq.test(sample_data(qd.baseline.Test)$Site,
sample_data(qd.baseline.Test)$Influenza_vacc_this_season)
chisq <- chisq.test(sample_data(qd.baseline.Test)$Site,
sample_data(qd.baseline.Test)
$Influenza_seasonal_vaccine_last_season)
chisq
##Significant
corrplot(chisq$residuals, is.cor = FALSE)
##
chisq.test(sample_data(qd.baseline.Test)$Site,
sample_data(qd.baseline.Test)$Influenza_vaccine_ever)
qd.baseline.Test2 <- subset_samples(qd.baseline.Test,
Has_pt_received_pneumonia_vaccine != "Unknown")
chisq <- chisq.test(sample_data(qd.baseline.Test2)$Site,
sample_data(qd.baseline.Test2)$Has_pt_received_pneumonia_vaccine)
chisq
##Significant
corrplot(chisq$residuals, is.cor = FALSE)
##
qd.baseline.Test2 = subset_samples(qd.baseline.Test,
Is_pt_in_shared_room != ".")
chisq <- chisq.test(sample_data(qd.baseline.Test2)$Site,
sample_data(qd.baseline.Test2)$Is_pt_in_shared_room)
chisq
##Significant
corrplot(chisq$residuals, is.cor = FALSE)
##
summary(aov(Barthel_Total ~ as.factor(Site),
data=baseline.test.sup))
chisq.test(sample_data(qd.baseline.Test)$Site,
sample_data(qd.baseline.Test)$COPD)
chisq.test(sample_data(qd.baseline.Test)$Site,
sample_data(qd.baseline.Test)$CHF)
chisq.test(sample_data(qd.baseline.Test)$Site,
sample_data(qd.baseline.Test)$CAD)
chisq.test(sample_data(qd.baseline.Test)$Site,
sample_data(qd.baseline.Test)$Anemia)
chisq.test(sample_data(qd.baseline.Test)$Site,
```

```
sample_data(qd.baseline.Test)$Dementia)
chisq.test(sample_data(qd.baseline.Test)$Site,
sample_data(qd.baseline.Test)$CVA.Stroke)
chisq.test(sample_data(qd.baseline.Test)$Site,
sample_data(qd.baseline.Test)$DM)
chisq.test(sample_data(qd.baseline.Test)$Site,
sample_data(qd.baseline.Test)$Hypothyroid)
summary(aov(Num_Comorbidities ~ as.factor(Site),
data=baseline.test.sup))
chisq <- chisq.test(sample_data(qd.baseline.Test)$Site,
sample_data(qd.baseline.Test)$Seizures)
chisq
##Significant
corrplot(chisq$residuals, is.cor = FALSE)
##
chisq.test(sample_data(qd.baseline.Test)$Site,
sample_data(qd.baseline.Test)$Cancer)
summary(aov(as.numeric(IL1B) ~ as.factor(Site),
data=baseline.test.sup))
summary(aov(as.numeric(IL6) ~ as.factor(Site),
data=baseline.test.sup))
summary(aov(as.numeric(TNFA) ~ as.factor(Site),
data=baseline.test.sup))
summary(aov(as.numeric(qPCR_Concentration) ~ as.factor(Site),
data=baseline.test.sup))

##Month (omit Feb)
qd.baseline.Test = subset_samples(qd.baseline, Month != "Feb")
baseline.test.sup = subset(baseline.test, (baseline.test$Month !=
"Feb"))
chisq <- chisq.test(sample_data(qd.baseline.Test)$Month,
sample_data(qd.baseline.Test)$Season)
chisq
##Significant
corrplot(chisq$residuals, is.cor = FALSE)
##
chisq <- chisq.test(sample_data(qd.baseline.Test)$Month,
sample_data(qd.baseline.Test)$Year)
chisq
##Significant
corrplot(chisq$residuals, is.cor = FALSE)
##
chisq.test(sample_data(qd.baseline.Test)$Month,
sample_data(qd.baseline.Test)$Completed.Study)
chisq.test(sample_data(qd.baseline.Test)$Month,
sample_data(qd.baseline.Test)$Allocation_Group_Probiotics)
chisq.test(sample_data(qd.baseline.Test)$Month,
sample_data(qd.baseline.Test)$hadEvent)
summary(aov(as.numeric(Num_Medications) ~ as.factor(Month),
data=baseline.test.sup))
chisq.test(sample_data(qd.baseline.Test)$Month,
sample_data(qd.baseline.Test)$Influenza_vacc_this_season)
chisq.test(sample_data(qd.baseline.Test)$Month,
sample_data(qd.baseline.Test)
```

```
$Influenza_seasonal_vaccine_last_season)
chisq.test(sample_data(qd.baseline.Test)$Month,
sample_data(qd.baseline.Test)$Influenza_vaccine_ever)
qd.baseline.Test2 <- subset_samples(qd.baseline.Test,
Has_pt_received_pneumonia_vaccine != "Unknown")
chisq <- chisq.test(sample_data(qd.baseline.Test2)$Month,
sample_data(qd.baseline.Test2)$Has_pt_received_pneumonia_vaccine)
chisq
##Significant
corrplot(chisq$residuals, is.cor = FALSE)
##
chisq.test(sample_data(qd.baseline.Test)$Month,
sample_data(qd.baseline.Test)$Is_pt_in_shared_room)
summary(aov(as.numeric(Barthel_Total) ~ as.factor(Month),
data=baseline.test.sup))
chisq.test(sample_data(qd.baseline.Test)$Month,
sample_data(qd.baseline.Test)$COPD)
chisq.test(sample_data(qd.baseline.Test)$Month,
sample_data(qd.baseline.Test)$CHF)
chisq.test(sample_data(qd.baseline.Test)$Month,
sample_data(qd.baseline.Test)$CAD)
chisq.test(sample_data(qd.baseline.Test)$Month,
sample_data(qd.baseline.Test)$Anemia)
chisq.test(sample_data(qd.baseline.Test)$Month,
sample_data(qd.baseline.Test)$Dementia)
chisq.test(sample_data(qd.baseline.Test)$Month,
sample_data(qd.baseline.Test)$CVA.Stroke)
chisq.test(sample_data(qd.baseline.Test)$Month,
sample_data(qd.baseline.Test)$DM)
chisq.test(sample_data(qd.baseline.Test)$Month,
sample_data(qd.baseline.Test)$Hypothyroid)
summary(aov(as.numeric(Num_Comorbidities) ~ as.factor(Month),
data=baseline.test.sup))
chisq.test(sample_data(qd.baseline.Test)$Month,
sample_data(qd.baseline.Test)$Seizures)
chisq.test(sample_data(qd.baseline.Test)$Month,
sample_data(qd.baseline.Test)$Cancer)
summary(aov(as.numeric(IL1B) ~ as.factor(Month),
data=baseline.test.sup))
###Significant, so:###
a1 <- aov(IL1B ~ as.factor(Month), data=baseline.test.sup)
nd = data.frame(Month = unique(baseline.test.sup$Month))
pred = predict(a1, newdata = nd, se.fit = TRUE)
pred
nd$Pred = pred$fit # adds the predicted group means to the data
frame
nd$Lo = nd$Pred - 1.96*pred$se.fit # lower bound of the confidence
interval by subtracting 1.96*se from the prediction
nd$Hi = nd$Pred + 1.96*pred$se.fit # upper bound ditto
nd$Month <- factor(nd$Month, levels=c("Oct","Nov","Dec","Jan"))
plt = ggplot(nd, aes(x = Month)) +
  geom_point(aes(y = Pred), size=4) +
  geom_linerange(aes(ymin = Lo, ymax = Hi), alpha=0.5) +
  theme(axis.text.x=element_text(angle=90,hjust=1,vjust=0.5))
```

```
plt
###
summary(aov(as.numeric(IL6) ~ as.factor(Month),
data=baseline.test.sup))
summary(aov(as.numeric(TNFA) ~ as.factor(Month),
data=baseline.test.sup))
summary(aov(as.numeric(qPCR_Concentration) ~ as.factor(Month),
data=baseline.test.sup))

#Season
chisq <- chisq.test(sample_data(qd.baseline)$Season,
sample_data(qd.baseline)$Year)
chisq
##Significant
corrplot(chisq$residuals, is.cor = FALSE)
##
chisq.test(sample_data(qd.baseline)$Season, sample_data(qd.baseline)
$Completed.Study)
chisq.test(sample_data(qd.baseline)$Season, sample_data(qd.baseline)
$Allocation_Group_Probiotics)
chisq.test(sample_data(qd.baseline)$Season, sample_data(qd.baseline)
$hadEvent)
summary(aov(as.numeric(Num_Medications) ~ as.factor(Season),
data=baseline.test))
chisq.test(sample_data(qd.baseline)$Season, sample_data(qd.baseline)
$Influenza_vacc_this_season)
chisq.test(sample_data(qd.baseline)$Season, sample_data(qd.baseline)
$Influenza_seasonal_vaccine_last_season)
chisq.test(sample_data(qd.baseline)$Season, sample_data(qd.baseline)
$Influenza_vaccine_ever)
qd.baseline.Test <- subset_samples(qd.baseline,
Has_pt_received_pneumonia_vaccine != "Unknown")
chisq <- chisq.test(sample_data(qd.baseline.Test)$Season,
sample_data(qd.baseline.Test)$Has_pt_received_pneumonia_vaccine)
##Significant
corrplot(chisq$residuals, is.cor = FALSE)
##
chisq.test(sample_data(qd.baseline)$Season, sample_data(qd.baseline)
$Is_pt_in_shared_room)
summary(aov(as.numeric(Barthel_Total) ~ as.factor(Season),
data=baseline.test))
chisq.test(sample_data(qd.baseline)$Season, sample_data(qd.baseline)
$COPD)
chisq.test(sample_data(qd.baseline)$Season, sample_data(qd.baseline)
$CHF)
chisq.test(sample_data(qd.baseline)$Season, sample_data(qd.baseline)
$CAD)
chisq.test(sample_data(qd.baseline)$Season, sample_data(qd.baseline)
$Anemia)
chisq <- chisq.test(sample_data(qd.baseline)$Season,
sample_data(qd.baseline)$Dementia)
chisq
##Significant
corrplot(chisq$residuals, is.cor = FALSE)
```

```
##
chisq.test(sample_data(qd.baseline)$Season, sample_data(qd.baseline)
$CVA.Stroke)
chisq.test(sample_data(qd.baseline)$Season, sample_data(qd.baseline)
$DM)
chisq.test(sample_data(qd.baseline)$Season, sample_data(qd.baseline)
$Hypothyroid)
summary(aov(as.numeric(Num_Comorbidities) ~ as.factor(Season),
data=baseline.test))
chisq.test(sample_data(qd.baseline)$Season, sample_data(qd.baseline)
$Seizures)
chisq.test(sample_data(qd.baseline)$Season, sample_data(qd.baseline)
$Cancer)
summary(aov(as.numeric(IL1B) ~ as.factor(Season),
data=baseline.test))
###Significant, so:###
a1 <- aov(IL1B ~ as.factor(Season), data=baseline.test)
nd = data.frame(Season = unique(baseline.test$Season))
pred = predict(a1, newdata = nd, se.fit = TRUE)
pred
nd$Pred = pred$fit # adds the predicted group means to the data
frame
nd$Lo = nd$Pred - 1.96*pred$se.fit # lower bound of the confidence
interval by subtracting 1.96*se from the prediction
nd$Hi = nd$Pred + 1.96*pred$se.fit # upper bound ditto
plt = ggplot(nd, aes(x = Season)) +
  geom_point(aes(y = Pred), size=4) +
  geom_linerange(aes(ymin = Lo, ymax = Hi), alpha=0.5) +
  theme(axis.text.x=element_text(angle=90,hjust=1,vjust=0.5))
plt
###
summary(aov(as.numeric(IL6) ~ as.factor(Season),
data=baseline.test))
summary(aov(as.numeric(TNFA) ~ as.factor(Season),
data=baseline.test))
summary(aov(as.numeric(qPCR_Concentration) ~ as.factor(Season),
data=baseline.test))

#Year
chisq.test(sample_data(qd.baseline)$Year, sample_data(qd.baseline)
$Completed.Study)
chisq.test(sample_data(qd.baseline)$Year, sample_data(qd.baseline)
$Allocation_Group_Probiotics)
chisq.test(sample_data(qd.baseline)$Year, sample_data(qd.baseline)
$hadEvent)
summary(aov(as.numeric(Num_Medications) ~ as.factor(Year),
data=baseline.test))
chisq.test(sample_data(qd.baseline)$Year, sample_data(qd.baseline)
$Influenza_vacc_this_season)
qd.baseline.test <- subset_samples(qd.baseline,
Influenza_seasonal_vaccine_last_season != "Unknown")
chisq.test(sample_data(qd.baseline.test)$Year,
sample_data(qd.baseline.test)
$Influenza_seasonal_vaccine_last_season)
```

```
chisq.test(sample_data(qd.baseline)$Year, sample_data(qd.baseline)
$Influenza_vaccine_ever)
chisq.test(sample_data(qd.baseline)$Year, sample_data(qd.baseline)
$Has_pt_received_pneumonia_vaccine)
chisq.test(sample_data(qd.baseline)$Year, sample_data(qd.baseline)
$Is_pt_in_shared_room)
summary(aov(as.numeric(Barthel_Total) ~ as.factor(Year),
data=baseline.test))
chisq.test(sample_data(qd.baseline)$Year, sample_data(qd.baseline)
$COPD)
chisq.test(sample_data(qd.baseline)$Year, sample_data(qd.baseline)
$CHF)
chisq.test(sample_data(qd.baseline)$Year, sample_data(qd.baseline)
$CAD)
chisq.test(sample_data(qd.baseline)$Year, sample_data(qd.baseline)
$Anemia)
chisq.test(sample_data(qd.baseline)$Year, sample_data(qd.baseline)
$Dementia)
chisq.test(sample_data(qd.baseline)$Year, sample_data(qd.baseline)
$CVA.Stroke)
chisq.test(sample_data(qd.baseline)$Year, sample_data(qd.baseline)
$DM)
chisq.test(sample_data(qd.baseline)$Year, sample_data(qd.baseline)
$Hypothyroid)
summary(aov(as.numeric(Num_Comorbidities) ~ as.factor(Year),
data=baseline.test))
chisq.test(sample_data(qd.baseline)$Year, sample_data(qd.baseline)
$Seizures)
chisq.test(sample_data(qd.baseline)$Year, sample_data(qd.baseline)
$Cancer)
summary(aov(as.numeric(IL1B) ~ as.factor(Year), data=baseline.test))
summary(aov(as.numeric(IL6) ~ as.factor(Year), data=baseline.test))
summary(aov(as.numeric(TNFA) ~ as.factor(Year), data=baseline.test))
summary(aov(as.numeric(qPCR_Concentration) ~ as.factor(Year),
data=baseline.test))

#Completed_Study
chisq.test(sample_data(qd.baseline)$Completed.Study,
sample_data(qd.baseline)$Allocation_Group_Probiotics)
chisq.test(sample_data(qd.baseline)$Completed.Study,
sample_data(qd.baseline)$hadEvent)
summary(aov(as.numeric(Num_Medications) ~
as.factor(Completed.Study), data=baseline.test))
chisq.test(sample_data(qd.baseline)$Completed.Study,
sample_data(qd.baseline)$Influenza_vacc_this_season)
chisq.test(sample_data(qd.baseline)$Completed.Study,
sample_data(qd.baseline)$Influenza_seasonal_vaccine_last_season)
chisq.test(sample_data(qd.baseline)$Completed.Study,
sample_data(qd.baseline)$Influenza_vaccine_ever)
chisq.test(sample_data(qd.baseline)$Completed.Study,
sample_data(qd.baseline)$Has_pt_received_pneumonia_vaccine)
chisq.test(sample_data(qd.baseline)$Completed.Study,
sample_data(qd.baseline)$Is_pt_in_shared_room)
summary(aov(as.numeric(Barthel_Total) ~ as.factor(Completed.Study),
```

```
data=baseline.test))
###Significant, so:###
a1 <- aov(Barthel_Total ~ as.factor(Completed.Study),
data=baseline.test)
nd = data.frame(Completed.Study =
unique(baseline.test$Completed.Study))
pred = predict(a1, newdata = nd, se.fit = TRUE)
pred
nd$Pred = pred$fit # adds the predicted group means to the data
frame
nd$Lo = nd$Pred - 1.96*pred$se.fit # lower bound of the confidence
interval by subtracting 1.96*se from the prediction
nd$Hi = nd$Pred + 1.96*pred$se.fit # upper bound ditto
plt = ggplot(nd, aes(x = Completed.Study)) +
  geom_point(aes(y = Pred), size=4) +
  geom_linerange(aes(ymin = Lo, ymax = Hi), alpha=0.5) +
  theme(axis.text.x=element_text(angle=90,hjust=1,vjust=0.5))
plt
###
chisq <- chisq.test(sample_data(qd.baseline)$Completed.Study,
sample_data(qd.baseline)$COPD)
chisq
##Significant
corrplot(chisq$residuals, is.cor = FALSE)
##
chisq.test(sample_data(qd.baseline)$Completed.Study,
sample_data(qd.baseline)$CHF)
chisq.test(sample_data(qd.baseline)$Completed.Study,
sample_data(qd.baseline)$CAD)
chisq.test(sample_data(qd.baseline)$Completed.Study,
sample_data(qd.baseline)$Anemia)
chisq.test(sample_data(qd.baseline)$Completed.Study,
sample_data(qd.baseline)$Dementia)
chisq.test(sample_data(qd.baseline)$Completed.Study,
sample_data(qd.baseline)$CVA.Stroke)
chisq.test(sample_data(qd.baseline)$Completed.Study,
sample_data(qd.baseline)$DM)
chisq.test(sample_data(qd.baseline)$Completed.Study,
sample_data(qd.baseline)$Hypothyroid)
summary(aov(as.numeric(Num_Comorbidities) ~
as.factor(Completed.Study), data=baseline.test))
chisq.test(sample_data(qd.baseline)$Completed.Study,
sample_data(qd.baseline)$Seizures)
chisq.test(sample_data(qd.baseline)$Completed.Study,
sample_data(qd.baseline)$Cancer)
summary(aov(as.numeric(IL1B) ~ as.factor(Completed.Study),
data=baseline.test))
summary(aov(as.numeric(IL6) ~ as.factor(Completed.Study),
data=baseline.test))
summary(aov(as.numeric(TNFA) ~ as.factor(Completed.Study),
data=baseline.test))
summary(aov(as.numeric(qPCR_Concentration) ~
as.factor(Completed.Study), data=baseline.test))
```

```
#Allocation_Group_Probiotics
chisq.test(sample_data(qd.baseline)$Allocation_Group_Probiotics,
sample_data(qd.baseline)$hadEvent)
summary(aov(as.numeric(Num_Medications) ~
as.factor(Allocation_Group_Probiotics), data=baseline.test))
chisq.test(sample_data(qd.baseline)$Allocation_Group_Probiotics,
sample_data(qd.baseline)$Influenza_vacc_this_season)
chisq.test(sample_data(qd.baseline)$Allocation_Group_Probiotics,
sample_data(qd.baseline)$Influenza_seasonal_vaccine_last_season)
chisq.test(sample_data(qd.baseline)$Allocation_Group_Probiotics,
sample_data(qd.baseline)$Influenza_vaccine_ever)
chisq.test(sample_data(qd.baseline)$Allocation_Group_Probiotics,
sample_data(qd.baseline)$Has_pt_received_pneumonia_vaccine)
chisq.test(sample_data(qd.baseline)$Allocation_Group_Probiotics,
sample_data(qd.baseline)$Is_pt_in_shared_room)
summary(aov(as.numeric(Barthel_Total) ~
as.factor(Allocation_Group_Probiotics), data=baseline.test))
chisq.test(sample_data(qd.baseline)$Allocation_Group_Probiotics,
sample_data(qd.baseline)$COPD)
chisq.test(sample_data(qd.baseline)$Allocation_Group_Probiotics,
sample_data(qd.baseline)$CHF)
chisq.test(sample_data(qd.baseline)$Allocation_Group_Probiotics,
sample_data(qd.baseline)$CAD)
chisq.test(sample_data(qd.baseline)$Allocation_Group_Probiotics,
sample_data(qd.baseline)$Anemia)
chisq.test(sample_data(qd.baseline)$Allocation_Group_Probiotics,
sample_data(qd.baseline)$Dementia)
chisq.test(sample_data(qd.baseline)$Allocation_Group_Probiotics,
sample_data(qd.baseline)$CVA.Stroke)
chisq.test(sample_data(qd.baseline)$Allocation_Group_Probiotics,
sample_data(qd.baseline)$DM)
chisq.test(sample_data(qd.baseline)$Allocation_Group_Probiotics,
sample_data(qd.baseline)$Hypothyroid)
summary(aov(as.numeric(Num_Comorbidities) ~
as.factor(Allocation_Group_Probiotics), data=baseline.test))
chisq.test(sample_data(qd.baseline)$Allocation_Group_Probiotics,
sample_data(qd.baseline)$Seizures)
chisq.test(sample_data(qd.baseline)$Allocation_Group_Probiotics,
sample_data(qd.baseline)$Cancer)
summary(aov(as.numeric(IL1B) ~
as.factor(Allocation_Group_Probiotics), data=baseline.test))
summary(aov(as.numeric(IL6) ~
as.factor(Allocation_Group_Probiotics), data=baseline.test))
summary(aov(as.numeric(TNFA) ~
as.factor(Allocation_Group_Probiotics), data=baseline.test))
summary(aov(as.numeric(qPCR_Concentration) ~
as.factor(Allocation_Group_Probiotics), data=baseline.test))

#hadEvent
summary(aov(as.numeric(Num_Medications) ~ as.factor(hadEvent),
data=baseline.test))
chisq.test(sample_data(qd.baseline)$hadEvent,
sample_data(qd.baseline)$Influenza_vacc_this_season)
chisq.test(sample_data(qd.baseline)$hadEvent,
```

```
sample_data(qd.baseline)$Influenza_seasonal_vaccine_last_season)
chisq.test(sample_data(qd.baseline)$hadEvent,
sample_data(qd.baseline)$Influenza_vaccine_ever)
chisq.test(sample_data(qd.baseline)$hadEvent,
sample_data(qd.baseline)$Has_pt_received_pneumonia_vaccine)
chisq.test(sample_data(qd.baseline)$hadEvent,
sample_data(qd.baseline)$Is_pt_in_shared_room)
summary(aov(as.numeric(Barthel_Total) ~ as.factor(hadEvent),
data=baseline.test))
chisq.test(sample_data(qd.baseline)$hadEvent,
sample_data(qd.baseline)$COPD)
chisq.test(sample_data(qd.baseline)$hadEvent,
sample_data(qd.baseline)$CHF)
chisq.test(sample_data(qd.baseline)$hadEvent,
sample_data(qd.baseline)$CAD)
chisq.test(sample_data(qd.baseline)$hadEvent,
sample_data(qd.baseline)$Anemia)
chisq.test(sample_data(qd.baseline)$hadEvent,
sample_data(qd.baseline)$Dementia)
chisq.test(sample_data(qd.baseline)$hadEvent,
sample_data(qd.baseline)$CVA.Stroke)
chisq.test(sample_data(qd.baseline)$hadEvent,
sample_data(qd.baseline)$DM)
chisq.test(sample_data(qd.baseline)$hadEvent,
sample_data(qd.baseline)$Hypothyroid)
summary(aov(as.numeric(Num_Comorbidities) ~ as.factor(hadEvent),
data=baseline.test))
chisq.test(sample_data(qd.baseline)$hadEvent,
sample_data(qd.baseline)$Seizures)
chisq.test(sample_data(qd.baseline)$hadEvent,
sample_data(qd.baseline)$Cancer)
summary(aov(as.numeric(IL1B) ~ as.factor(hadEvent),
data=baseline.test))

summary(aov(as.numeric(IL6) ~ as.factor(hadEvent),
data=baseline.test))
summary(aov(as.numeric(TNFA) ~ as.factor(hadEvent),
data=baseline.test))
summary(aov(as.numeric(qPCR_Concentration) ~ as.factor(hadEvent),
data=baseline.test))

#Smoker
qd.baseline.Test <- subset_samples(qd.baseline, Smoker != "Unknown")
baseline.test.sup = subset(baseline.test, (baseline.test$Smoker !=
"Unknown"))
summary(aov(as.numeric(Age_at_enrollment) ~ as.factor(Smoker),
data=baseline.test.sup))
###Significant, so:###
a1 <- aov(Age_at_enrollment ~ as.factor(Smoker),
data=baseline.test.sup)
nd = data.frame(Smoker = unique(baseline.test.sup$Smoker))
pred = predict(a1, newdata = nd, se.fit = TRUE)
pred
nd$Pred = pred$fit # adds the predicted group means to the data
```

```
frame
nd$Lo = nd$Pred - 1.96*pred$se.fit # lower bound of the confidence
interval by subtracting 1.96*se from the prediction
nd$Hi = nd$Pred + 1.96*pred$se.fit # upper bound ditto
plt = ggplot(nd, aes(x = Smoker)) +
  geom_point(aes(y = Pred), size=4) +
  geom_linerange(aes(ymin = Lo, ymax = Hi), alpha=0.5) +
  theme(axis.text.x=element_text(angle=90,hjust=1,vjust=0.5))
plt
###
chisq.test(sample_data(qd.baseline.Test)$Smoker,
sample_data(qd.baseline.Test)$Site)
chisq <- chisq.test(sample_data(qd.baseline.Test)$Smoker,
sample_data(qd.baseline.Test)$Month)
chisq
##Significant
corrplot(chisq$residuals, is.cor = FALSE)
##
chisq.test(sample_data(qd.baseline.Test)$Smoker,
sample_data(qd.baseline.Test)$Season)
chisq.test(sample_data(qd.baseline.Test)$Smoker,
sample_data(qd.baseline.Test)$Year)
chisq.test(sample_data(qd.baseline.Test)$Smoker,
sample_data(qd.baseline.Test)$Completed.Study)
chisq.test(sample_data(qd.baseline.Test)$Smoker,
sample_data(qd.baseline.Test)$Allocation_Group_Probiotics)
chisq.test(sample_data(qd.baseline.Test)$Smoker,
sample_data(qd.baseline.Test)$hadEvent)
summary(aov(Num_Medications ~ as.factor(Smoker),
data=baseline.test.sup))
chisq.test(sample_data(qd.baseline.Test)$Smoker,
sample_data(qd.baseline.Test)$Influenza_vacc_this_season)
chisq.test(sample_data(qd.baseline.Test)$Smoker,
sample_data(qd.baseline.Test)
$Influenza_seasonal_vaccine_last_season)
chisq.test(sample_data(qd.baseline.Test)$Smoker,
sample_data(qd.baseline.Test)$Influenza_vaccine_ever)
chisq.test(sample_data(qd.baseline.Test)$Smoker,
sample_data(qd.baseline.Test)$Has_pt_received_pneumonia_vaccine)
chisq.test(sample_data(qd.baseline.Test)$Smoker,
sample_data(qd.baseline.Test)$Is_pt_in_shared_room)
summary(aov(Barthel_Total ~ as.factor(Smoker),
data=baseline.test.sup))
chisq <- chisq.test(sample_data(qd.baseline.Test)$Smoker,
sample_data(qd.baseline.Test)$COPD)
chisq
##Significant
corrplot(chisq$residuals, is.cor = FALSE)
chisq.test(sample_data(qd.baseline.Test)$Smoker,
sample_data(qd.baseline.Test)$CHF)
chisq.test(sample_data(qd.baseline.Test)$Smoker,
sample_data(qd.baseline.Test)$CAD)
chisq.test(sample_data(qd.baseline.Test)$Smoker,
sample_data(qd.baseline.Test)$Anemia)
```

```
chisq.test(sample_data(qd.baseline.Test)$Smoker,
sample_data(qd.baseline.Test)$Dementia)
chisq.test(sample_data(qd.baseline.Test)$Smoker,
sample_data(qd.baseline.Test)$CVA.Stroke)
chisq.test(sample_data(qd.baseline.Test)$Smoker,
sample_data(qd.baseline.Test)$DM)
chisq.test(sample_data(qd.baseline.Test)$Smoker,
sample_data(qd.baseline.Test)$Hypothyroid)
summary(aov(Num_Comorbidities ~ as.factor(Smoker),
data=baseline.test.sup))
chisq.test(sample_data(qd.baseline.Test)$Smoker,
sample_data(qd.baseline.Test)$Seizures)
chisq.test(sample_data(qd.baseline.Test)$Smoker,
sample_data(qd.baseline.Test)$Cancer)
summary(aov(as.numeric(IL1B) ~ as.factor(Smoker),
data=baseline.test.sup))
summary(aov(as.numeric(IL6) ~ as.factor(Smoker),
data=baseline.test.sup))
summary(aov(as.numeric(TNFA) ~ as.factor(Smoker),
data=baseline.test.sup))
summary(aov(as.numeric(qPCR_Concentration) ~ as.factor(Smoker),
data=baseline.test.sup))

#Num_Medications
summary(aov(as.numeric(Num_Medications) ~
as.factor(Influenza_vacc_this_season), data=baseline.test))
summary(aov(as.numeric(Num_Medications) ~
as.factor(Influenza_seasonal_vaccine_last_season),
data=baseline.test))
summary(aov(as.numeric(Num_Medications) ~
as.factor(Influenza_vaccine_ever), data=baseline.test))
summary(aov(as.numeric(Num_Medications) ~
as.factor(Has_pt_received_pneumonia_vaccine), data=baseline.test))
summary(aov(as.numeric(Num_Medications) ~
as.factor(Is_pt_in_shared_room), data=baseline.test))
summary(aov(as.numeric(Num_Medications) ~ as.factor(Barthel_Total),
data=baseline.test))
summary(aov(as.numeric(Num_Medications) ~ as.factor(COPD),
data=baseline.test))
###Significant, so:###
a1 <- aov(Num_Medications ~ as.factor(COPD), data=baseline.test)
nd = data.frame(COPD = unique(baseline.test$COPD))
pred = predict(a1, newdata = nd, se.fit = TRUE)
pred
nd$Pred = pred$fit # adds the predicted group means to the data
frame
nd$Lo = nd$Pred - 1.96*pred$se.fit # lower bound of the confidence
interval by subtracting 1.96*se from the prediction
nd$Hi = nd$Pred + 1.96*pred$se.fit # upper bound ditto
plt = ggplot(nd, aes(x = COPD)) +
  geom_point(aes(y = Pred), size=4) +
  geom_linerange(aes(ymin = Lo, ymax = Hi), alpha=0.5) +
  theme(axis.text.x=element_text(angle=90,hjust=1,vjust=0.5))
plt
```

```
###
summary(aov(as.numeric(Num_Medications) ~ as.factor(CHF),
data=baseline.test))
###Significant, so:###
a1 <- aov(Num_Medications ~ as.factor(CHF), data=baseline.test)
nd = data.frame(CHF = unique(baseline.test$CHF))
pred = predict(a1, newdata = nd, se.fit = TRUE)
pred
nd$Pred = pred$fit # adds the predicted group means to the data
frame
nd$Lo = nd$Pred - 1.96*pred$se.fit # lower bound of the confidence
interval by subtracting 1.96*se from the prediction
nd$Hi = nd$Pred + 1.96*pred$se.fit # upper bound ditto
plt = ggplot(nd, aes(x = CHF)) +
  geom_point(aes(y = Pred), size=4) +
  geom_linerange(aes(ymin = Lo, ymax = Hi), alpha=0.5) +
  theme(axis.text.x=element_text(angle=90,hjust=1,vjust=0.5))
plt
###
summary(aov(as.numeric(Num_Medications) ~ as.factor(CAD),
data=baseline.test))
summary(aov(as.numeric(Num_Medications) ~ as.factor(Anemia),
data=baseline.test))
summary(aov(as.numeric(Num_Medications) ~ as.factor(Dementia),
data=baseline.test))
###Significant, so:###
a1 <- aov(Num_Medications ~ as.factor(Dementia), data=baseline.test)
nd = data.frame(Dementia = unique(baseline.test$Dementia))
pred = predict(a1, newdata = nd, se.fit = TRUE)
pred
nd$Pred = pred$fit # adds the predicted group means to the data
frame
nd$Lo = nd$Pred - 1.96*pred$se.fit # lower bound of the confidence
interval by subtracting 1.96*se from the prediction
nd$Hi = nd$Pred + 1.96*pred$se.fit # upper bound ditto
plt = ggplot(nd, aes(x = Dementia)) +
  geom_point(aes(y = Pred), size=4) +
  geom_linerange(aes(ymin = Lo, ymax = Hi), alpha=0.5) +
  theme(axis.text.x=element_text(angle=90,hjust=1,vjust=0.5))
plt
###
summary(aov(as.numeric(Num_Medications) ~ as.factor(CVA.Stroke),
data=baseline.test))
summary(aov(as.numeric(Num_Medications) ~ as.factor(DM),
data=baseline.test))
###Significant, so:###
a1 <- aov(Num_Medications ~ as.factor(DM), data=baseline.test)
nd = data.frame(DM = unique(baseline.test$DM))
pred = predict(a1, newdata = nd, se.fit = TRUE)
pred
nd$Pred = pred$fit # adds the predicted group means to the data
frame
nd$Lo = nd$Pred - 1.96*pred$se.fit # lower bound of the confidence
interval by subtracting 1.96*se from the prediction
```

```
nd$Hi = nd$Pred + 1.96*pred$se.fit # upper bound ditto
plt = ggplot(nd, aes(x = DM)) +
  geom_point(aes(y = Pred), size=4) +
  geom_linerange(aes(ymin = Lo, ymax = Hi), alpha=0.5) +
  theme(axis.text.x=element_text(angle=90,hjust=1,vjust=0.5))
plt
###
summary(aov(as.numeric(Num_Medications) ~ as.factor(Hypothyroid),
data=baseline.test))
summary(aov(as.numeric(Num_Medications) ~
as.numeric(Num_Comorbidities), data=baseline.test))
###Significant, so:###
a1 <- aov(Num_Medications ~ as.factor(Num_Comorbidities),
data=baseline.test)
nd = data.frame(Num_Comorbidities =
unique(baseline.test$Num_Comorbidities))
pred = predict(a1, newdata = nd, se.fit = TRUE)
pred
nd$Pred = pred$fit # adds the predicted group means to the data
frame
nd$Lo = nd$Pred - 1.96*pred$se.fit # lower bound of the confidence
interval by subtracting 1.96*se from the prediction
nd$Hi = nd$Pred + 1.96*pred$se.fit # upper bound ditto
plt = ggplot(nd, aes(x = Num_Comorbidities)) +
  geom_point(aes(y = Pred), size=4) +
  geom_linerange(aes(ymin = Lo, ymax = Hi), alpha=0.5) +
  theme(axis.text.x=element_text(angle=90,hjust=1,vjust=0.5))
plt
###
summary(aov(as.numeric(Num_Medications) ~ as.factor(Seizures),
data=baseline.test))
summary(aov(as.numeric(Num_Medications) ~ as.factor(Cancer),
data=baseline.test))
summary(aov(as.numeric(Num_Medications) ~ as.factor(IL1B),
data=baseline.test))
summary(aov(as.numeric(Num_Medications) ~ as.factor(IL6),
data=baseline.test))
summary(aov(as.numeric(Num_Medications) ~ as.factor(TNFA),
data=baseline.test))
summary(aov(as.numeric(Num_Medications) ~
as.factor(qPCR_Concentration), data=baseline.test))

#Influenza_vacc_this_season
qd.baseline.Test <- subset_samples(qd.baseline,
Influenza_vacc_this_season != "Unknown")
baseline.test.sup = subset(baseline.test,
(baseline.test$Influenza_vacc_this_season != "Unknown"))
qd.baseline.Test2 <- subset_samples(qd.baseline.Test,
Influenza_seasonal_vaccine_last_season != "Unknown")
chisq <-
chisq.test(sample_data(qd.baseline.Test2)$Influenza_vacc_this_season
,
sample_data(qd.baseline.Test2)$Influenza_seasonal_vaccine_last_seaso
n)
```

```
chisq
##Significant
corrplot(chisq$residuals, is.cor = FALSE)
##
qd.baseline.Test2 <- subset_samples(qd.baseline.Test,
Influenza_vaccine_ever != "Unknown")
chisq <-
chisq.test(sample_data(qd.baseline.Test2)$Influenza_vacc_this_season
, sample_data(qd.baseline.Test2)$Influenza_vaccine_ever)
chisq
##Significant
corrplot(chisq$residuals, is.cor = FALSE)
##
chisq <-
chisq.test(sample_data(qd.baseline.Test2)$Influenza_vacc_this_season
, sample_data(qd.baseline.Test2)$Has_pt_received_pneumonia_vaccine)
chisq
##Significant
corrplot(chisq$residuals, is.cor = FALSE)
##
chisq.test(sample_data(qd.baseline.Test)$Influenza_vacc_this_season,
sample_data(qd.baseline.Test)$Is_pt_in_shared_room)
summary(aov(as.numeric(Barthel_Total) ~
as.factor(Influenza_vacc_this_season), data=baseline.test.sup))
chisq.test(sample_data(qd.baseline.Test)$Influenza_vacc_this_season,
sample_data(qd.baseline.Test)$COPD)
chisq.test(sample_data(qd.baseline.Test)$Influenza_vacc_this_season,
sample_data(qd.baseline.Test)$CHF)
chisq.test(sample_data(qd.baseline.Test)$Influenza_vacc_this_season,
sample_data(qd.baseline.Test)$CAD)
chisq.test(sample_data(qd.baseline.Test)$Influenza_vacc_this_season,
sample_data(qd.baseline.Test)$Anemia)
chisq.test(sample_data(qd.baseline.Test)$Influenza_vacc_this_season,
sample_data(qd.baseline.Test)$Dementia)
chisq.test(sample_data(qd.baseline.Test)$Influenza_vacc_this_season,
sample_data(qd.baseline.Test)$CVA.Stroke)
chisq.test(sample_data(qd.baseline.Test)$Influenza_vacc_this_season,
sample_data(qd.baseline.Test)$DM)
chisq.test(sample_data(qd.baseline.Test)$Influenza_vacc_this_season,
sample_data(qd.baseline.Test)$Hypothyroid)
summary(aov(as.numeric(Num_Comorbidities) ~
as.factor(Influenza_vacc_this_season), data=baseline.test.sup))
chisq.test(sample_data(qd.baseline.Test)$Influenza_vacc_this_season,
sample_data(qd.baseline.Test)$Seizures)
chisq.test(sample_data(qd.baseline.Test)$Influenza_vacc_this_season,
sample_data(qd.baseline.Test)$Cancer)
summary(aov(as.numeric(IL1B) ~
as.factor(Influenza_vacc_this_season), data=baseline.test.sup))
###Significant, so:###
a1 <- aov(IL1B ~ as.factor(Influenza_vacc_this_season),
data=baseline.test.sup)
nd = data.frame(Influenza_vacc_this_season =
unique(baseline.test.sup$Influenza_vacc_this_season))
pred = predict(a1, newdata = nd, se.fit = TRUE)
```

```
pred
nd$Pred = pred$fit # adds the predicted group means to the data
frame
nd$Lo = nd$Pred - 1.96*pred$se.fit # lower bound of the confidence
interval by subtracting 1.96*se from the prediction
nd$Hi = nd$Pred + 1.96*pred$se.fit # upper bound ditto
plt = ggplot(nd, aes(x = Influenza_vacc_this_season)) +
  geom_point(aes(y = Pred), size=4) +
  geom_linerange(aes(ymin = Lo, ymax = Hi), alpha=0.5) +
  theme(axis.text.x=element_text(angle=90,hjust=1,vjust=0.5))
plt
p <- ggplot(baseline.test.sup, aes(x=Influenza_vacc_this_season,
y=IL1B)) +
  geom_point() +
  geom_boxplot() +
  scale_y_log10()
p
###
summary(aov(as.numeric(IL6) ~ as.factor(Influenza_vacc_this_season),
data=baseline.test.sup))
summary(aov(as.numeric(TNFA) ~
as.factor(Influenza_vacc_this_season), data=baseline.test.sup))
summary(aov(as.numeric(qPCR_Concentration) ~
as.factor(Influenza_vacc_this_season), data=baseline.test.sup))
###Significant, so:###
a1 <- aov(qPCR_Concentration ~
as.factor(Influenza_vacc_this_season), data=baseline.test.sup)
nd = data.frame(Influenza_vacc_this_season =
unique(baseline.test.sup$Influenza_vacc_this_season))
pred = predict(a1, newdata = nd, se.fit = TRUE)
pred
nd$Pred = pred$fit # adds the predicted group means to the data
frame
nd$Lo = nd$Pred - 1.96*pred$se.fit # lower bound of the confidence
interval by subtracting 1.96*se from the prediction
nd$Hi = nd$Pred + 1.96*pred$se.fit # upper bound ditto
plt = ggplot(nd, aes(x = Influenza_vacc_this_season)) +
  geom_point(aes(y = Pred), size=4) +
  geom_linerange(aes(ymin = Lo, ymax = Hi), alpha=0.5) +
  theme(axis.text.x=element_text(angle=90,hjust=1,vjust=0.5))
plt
p <- ggplot(baseline.test.sup, aes(x=Influenza_vacc_this_season,
y=qPCR_Concentration)) +
  geom_point() +
  geom_boxplot() +
  scale_y_log10()
p
###

#Influenza_seasonal_vaccine_last_season
qd.baseline.Test <- subset_samples(qd.baseline,
Influenza_seasonal_vaccine_last_season != "Unknown")
baseline.test.sup = subset(baseline.test,
baseline.test$Influenza_seasonal_vaccine_last_season != "Unknown")
```

```
qd.baseline.Test2 <- subset_samples(qd.baseline.Test,
Influenza_vaccine_ever != "Unknown")
chisq <-
chisq.test(sample_data(qd.baseline.Test2)$Influenza_seasonal_vaccine
_last_season, sample_data(qd.baseline.Test2)$Influenza_vaccine_ever)
chisq
##Significant
corrplot(chisq$residuals, is.cor = FALSE)
##
qd.baseline.Test2 <- subset_samples(qd.baseline.Test,
Has_pt_received_pneumonia_vaccine != "Unknown")
chisq <-
chisq.test(sample_data(qd.baseline.Test2)$Influenza_seasonal_vaccine
_last_season,
sample_data(qd.baseline.Test2)$Has_pt_received_pneumonia_vaccine)
chisq
##Significant
corrplot(chisq$residuals, is.cor = FALSE)
##
chisq.test(sample_data(qd.baseline.Test)
$Influenza_seasonal_vaccine_last_season,
sample_data(qd.baseline.Test)$Is_pt_in_shared_room)
summary(aov(Barthel_Total ~
as.factor(Influenza_seasonal_vaccine_last_season),
data=baseline.test.sup))
chisq.test(sample_data(qd.baseline.Test)
$Influenza_seasonal_vaccine_last_season,
sample_data(qd.baseline.Test)$COPD)
chisq.test(sample_data(qd.baseline.Test)
$Influenza_seasonal_vaccine_last_season,
sample_data(qd.baseline.Test)$CHF)
chisq.test(sample_data(qd.baseline.Test)
$Influenza_seasonal_vaccine_last_season,
sample_data(qd.baseline.Test)$CAD)
chisq.test(sample_data(qd.baseline.Test)
$Influenza_seasonal_vaccine_last_season,
sample_data(qd.baseline.Test)$Anemia)
chisq.test(sample_data(qd.baseline.Test)
$Influenza_seasonal_vaccine_last_season,
sample_data(qd.baseline.Test)$Dementia)
chisq.test(sample_data(qd.baseline.Test)
$Influenza_seasonal_vaccine_last_season,
sample_data(qd.baseline.Test)$CVA.Stroke)
chisq.test(sample_data(qd.baseline.Test)
$Influenza_seasonal_vaccine_last_season,
sample_data(qd.baseline.Test)$DM)
chisq.test(sample_data(qd.baseline.Test)
$Influenza_seasonal_vaccine_last_season,
sample_data(qd.baseline.Test)$Hypothyroid)
summary(aov(as.numeric(Num_Comorbidities) ~
as.factor(Influenza_seasonal_vaccine_last_season),
data=baseline.test.sup))
###Significant, so:###
a1 <- aov(Num_Comorbidities ~
```

```
as.factor(Influenza_seasonal_vaccine_last_season),
data=baseline.test.sup)
nd = data.frame(Influenza_seasonal_vaccine_last_season =
unique(baseline.test.sup$Influenza_seasonal_vaccine_last_season))
pred = predict(a1, newdata = nd, se.fit = TRUE)
pred
nd$Pred = pred$fit # adds the predicted group means to the data
frame
nd$Lo = nd$Pred - 1.96*pred$se.fit # lower bound of the confidence
interval by subtracting 1.96*se from the prediction
nd$Hi = nd$Pred + 1.96*pred$se.fit # upper bound ditto
plt = ggplot(nd, aes(x = Influenza_seasonal_vaccine_last_season)) +
  geom_point(aes(y = Pred), size=4) +
  geom_linerange(aes(ymin = Lo, ymax = Hi), alpha=0.5) +
  theme(axis.text.x=element_text(angle=90,hjust=1,vjust=0.5))
plt
p <- ggplot(baseline.test.sup,
aes(x=Influenza_seasonal_vaccine_last_season, y=Num_Comorbidities))
+
  geom_point() +
  geom_boxplot() +
  scale_y_log10()
p
###
chisq.test(sample_data(qd.baseline.Test)
$Influenza_seasonal_vaccine_last_season,
sample_data(qd.baseline.Test)$Seizures)
chisq.test(sample_data(qd.baseline.Test)
$Influenza_seasonal_vaccine_last_season,
sample_data(qd.baseline.Test)$Cancer)
summary(aov(as.numeric(IL1B) ~
as.factor(Influenza_seasonal_vaccine_last_season),
data=baseline.test.sup))
summary(aov(as.numeric(IL6) ~
as.factor(Influenza_seasonal_vaccine_last_season),
data=baseline.test.sup))
summary(aov(as.numeric(TNFA) ~
as.factor(Influenza_seasonal_vaccine_last_season),
data=baseline.test.sup))
summary(aov(as.numeric(qPCR_Concentration) ~
as.factor(Influenza_seasonal_vaccine_last_season),
data=baseline.test.sup))

#Influenza_vaccine_ever
qd.baseline.Test <- subset_samples(qd.baseline,
Influenza_vaccine_ever != "Unknown")
baseline.test.sup = subset(baseline.test,
baseline.test$Influenza_vaccine_ever != "Unknown")
qd.baseline.Test2 <- subset_samples(qd.baseline.Test,
Has_pt_received_pneumonia_vaccine != "Unknown")
chisq <-
chisq.test(sample_data(qd.baseline.Test2)$Influenza_vaccine_ever,
sample_data(qd.baseline.Test2)$Has_pt_received_pneumonia_vaccine)
chisq
```

```
##Significant
corrplot(chisq$residuals, is.cor = FALSE)
##
chisq.test(sample_data(qd.baseline.Test)$Influenza_vaccine_ever,
sample_data(qd.baseline.Test)$Is_pt_in_shared_room)
summary(aov(as.numeric(Barthel_Total) ~
as.factor(Influenza_vaccine_ever), data=baseline.test.sup))
###Significant, so:###
a1 <- aov(Barthel_Total ~ as.factor(Influenza_vaccine_ever),
data=baseline.test.sup)
nd = data.frame(Influenza_vaccine_ever =
unique(baseline.test.sup$Influenza_vaccine_ever))
pred = predict(a1, newdata = nd, se.fit = TRUE)
pred
nd$Pred = pred$fit # adds the predicted group means to the data
frame
nd$Lo = nd$Pred - 1.96*pred$se.fit # lower bound of the confidence
interval by subtracting 1.96*se from the prediction
nd$Hi = nd$Pred + 1.96*pred$se.fit # upper bound ditto
plt = ggplot(nd, aes(x = Influenza_vaccine_ever)) +
  geom_point(aes(y = Pred), size=4) +
  geom_linerange(aes(ymin = Lo, ymax = Hi), alpha=0.5) +
  theme(axis.text.x=element_text(angle=90,hjust=1,vjust=0.5))
plt
p <- ggplot(baseline.test.sup, aes(x=Influenza_vaccine_ever,
y=Barthel_Total)) +
  geom_point() +
  geom_boxplot()
p
###
chisq.test(sample_data(qd.baseline.Test)$Influenza_vaccine_ever,
sample_data(qd.baseline.Test)$COPD)
chisq.test(sample_data(qd.baseline.Test)$Influenza_vaccine_ever,
sample_data(qd.baseline.Test)$CHF)
chisq.test(sample_data(qd.baseline.Test)$Influenza_vaccine_ever,
sample_data(qd.baseline.Test)$CAD)
chisq.test(sample_data(qd.baseline.Test)$Influenza_vaccine_ever,
sample_data(qd.baseline.Test)$Anemia)
chisq.test(sample_data(qd.baseline.Test)$Influenza_vaccine_ever,
sample_data(qd.baseline.Test)$Dementia)
chisq.test(sample_data(qd.baseline.Test)$Influenza_vaccine_ever,
sample_data(qd.baseline.Test)$CVA.Stroke)
chisq.test(sample_data(qd.baseline.Test)$Influenza_vaccine_ever,
sample_data(qd.baseline.Test)$DM)
chisq.test(sample_data(qd.baseline.Test)$Influenza_vaccine_ever,
sample_data(qd.baseline.Test)$Hypothyroid)
summary(aov(as.numeric(Num_Comorbidities) ~
as.factor(Influenza_vaccine_ever), data=baseline.test.sup))
###Significant, so:###
a1 <- aov(Num_Comorbidities ~ as.factor(Influenza_vaccine_ever),
data=baseline.test.sup)
nd = data.frame(Influenza_vaccine_ever =
unique(baseline.test.sup$Influenza_vaccine_ever))
pred = predict(a1, newdata = nd, se.fit = TRUE)
```

```
pred
nd$Pred = pred$fit # adds the predicted group means to the data
frame
nd$Lo = nd$Pred - 1.96*pred$se.fit # lower bound of the confidence
interval by subtracting 1.96*se from the prediction
nd$Hi = nd$Pred + 1.96*pred$se.fit # upper bound ditto
plt = ggplot(nd, aes(x = Influenza_vaccine_ever)) +
  geom_point(aes(y = Pred), size=4) +
  geom_linerange(aes(ymin = Lo, ymax = Hi), alpha=0.5) +
  theme(axis.text.x=element_text(angle=90,hjust=1,vjust=0.5))
plt
p <- ggplot(baseline.test.sup, aes(x=Influenza_vaccine_ever,
y=Num_Comorbidities)) +
  geom_point() +
  geom_boxplot() +
  scale_y_log10()
p
###
chisq.test(sample_data(qd.baseline.Test)$Influenza_vaccine_ever,
sample_data(qd.baseline.Test)$Seizures)
chisq.test(sample_data(qd.baseline.Test)$Influenza_vaccine_ever,
sample_data(qd.baseline.Test)$Cancer)
summary(aov(as.numeric(IL1B) ~ as.factor(Influenza_vaccine_ever),
data=baseline.test.sup))
###Significant, so:###
a1 <- aov(IL1B ~ as.factor(Influenza_vaccine_ever),
data=baseline.test.sup)
nd = data.frame(Influenza_vaccine_ever =
unique(baseline.test.sup$Influenza_vaccine_ever))
pred = predict(a1, newdata = nd, se.fit = TRUE)
pred
nd$Pred = pred$fit # adds the predicted group means to the data
frame
nd$Lo = nd$Pred - 1.96*pred$se.fit # lower bound of the confidence
interval by subtracting 1.96*se from the prediction
nd$Hi = nd$Pred + 1.96*pred$se.fit # upper bound ditto
plt = ggplot(nd, aes(x = Influenza_vaccine_ever)) +
  geom_point(aes(y = Pred), size=4) +
  geom_linerange(aes(ymin = Lo, ymax = Hi), alpha=0.5) +
  theme(axis.text.x=element_text(angle=90,hjust=1,vjust=0.5))
plt
p <- ggplot(baseline.test.sup, aes(x=Influenza_vaccine_ever,
y=IL1B)) +
  geom_point() +
  geom_boxplot() +
  scale_y_log10()
p
###
summary(aov(as.numeric(IL6) ~ as.factor(Influenza_vaccine_ever),
data=baseline.test.sup))
summary(aov(as.numeric(TNFA) ~ as.factor(Influenza_vaccine_ever),
data=baseline.test.sup))
summary(aov(as.numeric(qPCR_Concentration) ~
as.factor(Influenza_vaccine_ever), data=baseline.test.sup))
```

```
###Significant, so:###
a1 <- aov(qPCR_Concentration ~ as.factor(Influenza_vaccine_ever),
data=baseline.test.sup)
nd = data.frame(Influenza_vaccine_ever =
unique(baseline.test.sup$Influenza_vaccine_ever))
pred = predict(a1, newdata = nd, se.fit = TRUE)
pred
nd$Pred = pred$fit # adds the predicted group means to the data
frame
nd$Lo = nd$Pred - 1.96*pred$se.fit # lower bound of the confidence
interval by subtracting 1.96*se from the prediction
nd$Hi = nd$Pred + 1.96*pred$se.fit # upper bound ditto
plt = ggplot(nd, aes(x = Influenza_vaccine_ever)) +
  geom_point(aes(y = Pred), size=4) +
  geom_linerange(aes(ymin = Lo, ymax = Hi), alpha=0.5) +
  theme(axis.text.x=element_text(angle=90,hjust=1,vjust=0.5))
plt
p <- ggplot(baseline.test.sup, aes(x=Influenza_vaccine_ever,
y=qPCR_Concentration)) +
  geom_point() +
  geom_boxplot() +
  scale_y_log10()
p
###

#Has_pt_received_pneumonia_vaccine
#####
qd.baseline.Test2 <- subset_samples(qd.baseline.Test,
Has_pt_received_pneumonia_vaccine != "Unknown")
baseline.test.sup = subset(baseline.test,
baseline.test$Has_pt_received_pneumonia_vaccine != "Unknown")
chisq.test(sample_data(qd.baseline.Test2)$Has_pt_received_pneumonia_
vaccine, sample_data(qd.baseline.Test2)$Is_pt_in_shared_room)
summary(aov(as.numeric(Barthel_Total) ~
as.factor(Has_pt_received_pneumonia_vaccine),
data=baseline.test.sup))
chisq.test(sample_data(qd.baseline.Test2)$Has_pt_received_pneumonia_
vaccine, sample_data(qd.baseline.Test2)$COPD)
chisq.test(sample_data(qd.baseline.Test2)$Has_pt_received_pneumonia_
vaccine, sample_data(qd.baseline.Test2)$CHF)
chisq.test(sample_data(qd.baseline.Test2)$Has_pt_received_pneumonia_
vaccine, sample_data(qd.baseline.Test2)$CAD)
chisq.test(sample_data(qd.baseline.Test2)$Has_pt_received_pneumonia_
vaccine, sample_data(qd.baseline.Test2)$Anemia)
chisq.test(sample_data(qd.baseline.Test2)$Has_pt_received_pneumonia_
vaccine, sample_data(qd.baseline.Test2)$Dementia)
chisq.test(sample_data(qd.baseline.Test2)$Has_pt_received_pneumonia_
vaccine, sample_data(qd.baseline.Test2)$CVA.Stroke)
chisq.test(sample_data(qd.baseline.Test2)$Has_pt_received_pneumonia_
vaccine, sample_data(qd.baseline.Test2)$DM)
chisq.test(sample_data(qd.baseline.Test2)$Has_pt_received_pneumonia_
vaccine, sample_data(qd.baseline.Test2)$Hypothyroid)
summary(aov(as.numeric(Num_Comorbidities) ~
as.factor(Has_pt_received_pneumonia_vaccine),
```

```
data=baseline.test.sup))
chisq.test(sample_data(qd.baseline.Test2)$Has_pt_received_pneumonia_
vaccine, sample_data(qd.baseline.Test2)$Seizures)
qd.baseline.Test3 <- subset_samples(qd.baseline.Test2, Cancer !=
"Prior")
chisq <-
chisq.test(sample_data(qd.baseline.Test3)$Has_pt_received_pneumonia_
vaccine, sample_data(qd.baseline.Test3)$Cancer)
chisq
##Significant
corrplot(chisq$residuals, is.cor = FALSE)
##
summary(aov(as.numeric(IL1B) ~
as.factor(Has_pt_received_pneumonia_vaccine),
data=baseline.test.sup))
summary(aov(as.numeric(IL6) ~
as.factor(Has_pt_received_pneumonia_vaccine),
data=baseline.test.sup))
summary(aov(as.numeric(TNFA) ~
as.factor(Has_pt_received_pneumonia_vaccine),
data=baseline.test.sup))
summary(aov(as.numeric(qPCR_Concentration) ~
as.factor(Has_pt_received_pneumonia_vaccine),
data=baseline.test.sup))

#Is_pt_in_shared_room
qd.baseline.Test <- subset_samples(qd.baseline,
Is_pt_in_shared_room != ".")
baseline.test.sup = subset(baseline.test,
baseline.test$Is_pt_in_shared_room != ".")
summary(aov(as.numeric(Barthel_Total) ~
as.factor(Is_pt_in_shared_room), data=baseline.test.sup))
chisq.test(sample_data(qd.baseline.Test)$Is_pt_in_shared_room,
sample_data(qd.baseline.Test)$COPD)
chisq.test(sample_data(qd.baseline.Test)$Is_pt_in_shared_room,
sample_data(qd.baseline.Test)$CHF)
chisq.test(sample_data(qd.baseline.Test)$Is_pt_in_shared_room,
sample_data(qd.baseline.Test)$CAD)
chisq.test(sample_data(qd.baseline.Test)$Is_pt_in_shared_room,
sample_data(qd.baseline.Test)$Anemia)
chisq.test(sample_data(qd.baseline.Test)$Is_pt_in_shared_room,
sample_data(qd.baseline.Test)$Dementia)
chisq.test(sample_data(qd.baseline.Test)$Is_pt_in_shared_room,
sample_data(qd.baseline.Test)$CVA.Stroke)
chisq.test(sample_data(qd.baseline.Test)$Is_pt_in_shared_room,
sample_data(qd.baseline.Test)$DM)
chisq.test(sample_data(qd.baseline.Test)$Is_pt_in_shared_room,
sample_data(qd.baseline.Test)$Hypothyroid)
summary(aov(as.numeric(Num_Comorbidities) ~
as.factor(Is_pt_in_shared_room), data=baseline.test.sup))
chisq.test(sample_data(qd.baseline.Test)$Is_pt_in_shared_room,
sample_data(qd.baseline.Test)$Seizures)
chisq.test(sample_data(qd.baseline.Test)$Is_pt_in_shared_room,
sample_data(qd.baseline.Test)$Cancer)
```

```
summary(aov(as.numeric(IL1B) ~ as.factor(Is_pt_in_shared_room),
data=baseline.test.sup))
summary(aov(as.numeric(IL6) ~ as.factor(Is_pt_in_shared_room),
data=baseline.test.sup))
summary(aov(as.numeric(TNFA) ~ as.factor(Is_pt_in_shared_room),
data=baseline.test.sup))
summary(aov(as.numeric(qPCR_Concentration) ~
as.factor(Is_pt_in_shared_room), data=baseline.test.sup))
###Significant, so:###
a1 <- aov(qPCR_Concentration ~ as.factor(Is_pt_in_shared_room),
data=baseline.test.sup)
nd = data.frame(Is_pt_in_shared_room =
unique(baseline.test.sup$Is_pt_in_shared_room))
pred = predict(a1, newdata = nd, se.fit = TRUE)
pred
nd$Pred = pred$fit # adds the predicted group means to the data
frame
nd$Lo = nd$Pred - 1.96*pred$se.fit # lower bound of the confidence
interval by subtracting 1.96*se from the prediction
nd$Hi = nd$Pred + 1.96*pred$se.fit # upper bound ditto
plt = ggplot(nd, aes(x = Is_pt_in_shared_room)) +
  geom_point(aes(y = Pred), size=4) +
  geom_linerange(aes(ymin = Lo, ymax = Hi), alpha=0.5) +
  theme(axis.text.x=element_text(angle=90,hjust=1,vjust=0.5))
plt
p <- ggplot(baseline.test.sup, aes(x=Is_pt_in_shared_room,
y=qPCR_Concentration)) +
  geom_point() +
  geom_boxplot() +
  scale_y_log10()
p
###

#Barthel_Total
summary(aov(as.numeric(Barthel_Total) ~ as.factor(COPD),
data=baseline.test))
summary(aov(as.numeric(Barthel_Total) ~ as.factor(CHF),
data=baseline.test))
summary(aov(as.numeric(Barthel_Total) ~ as.factor(CAD),
data=baseline.test))
summary(aov(as.numeric(Barthel_Total) ~ as.factor(Anemia),
data=baseline.test))
summary(aov(as.numeric(Barthel_Total) ~ as.factor(Dementia),
data=baseline.test))
summary(aov(as.numeric(Barthel_Total) ~ as.factor(CVA.Stroke),
data=baseline.test))
summary(aov(as.numeric(Barthel_Total) ~ as.factor(DM),
data=baseline.test))
summary(aov(as.numeric(Barthel_Total) ~ as.factor(Hypothyroid),
data=baseline.test))
summary(aov(as.numeric(Barthel_Total) ~
as.numeric(Num_Comorbidities), data=baseline.test))
summary(aov(as.numeric(Barthel_Total) ~ as.factor(Seizures),
data=baseline.test))
```

```
summary(aov(as.numeric(Barthel_Total) ~ as.factor(Cancer),
data=baseline.test))
summary(aov(as.numeric(Barthel_Total) ~ as.numeric(IL1B),
data=baseline.test))
summary(aov(as.numeric(Barthel_Total) ~ as.numeric(IL6),
data=baseline.test))
summary(aov(as.numeric(Barthel_Total) ~ as.numeric(TNFA),
data=baseline.test))
summary(aov(as.numeric(Barthel_Total) ~
as.numeric(qPCR_Concentration), data=baseline.test))
###Significant, so:###
a1 <- aov(qPCR_Concentration ~ as.factor(Barthel_Total),
data=baseline.test)
nd = data.frame(Barthel_Total = unique(baseline.test$Barthel_Total))
pred = predict(a1, newdata = nd, se.fit = TRUE)
pred
nd$Pred = pred$fit # adds the predicted group means to the data
frame
nd$Lo = nd$Pred - 1.96*pred$se.fit # lower bound of the confidence
interval by subtracting 1.96*se from the prediction
nd$Hi = nd$Pred + 1.96*pred$se.fit # upper bound ditto
plt = ggplot(nd, aes(x = Barthel_Total)) +
  geom_point(aes(y = Pred), size=4) +
  geom_linerange(aes(ymin = Lo, ymax = Hi), alpha=0.5) +
  theme(axis.text.x=element_text(angle=90,hjust=1,vjust=0.5))
plt
p <- ggplot(baseline.test.sup, aes(x=Barthel_Total,
y=qPCR_Concentration)) +
  geom_point() +
  scale_y_log10()
p

#COPD
chisq.test(sample_data(qd.baseline)$COPD, sample_data(qd.baseline)
$CHF)
chisq <- chisq.test(sample_data(qd.baseline)$COPD,
sample_data(qd.baseline)$CAD)
chisq
##Significant
corrplot(chisq$residuals, is.cor = FALSE)
##
chisq.test(sample_data(qd.baseline)$COPD, sample_data(qd.baseline)
$Anemia)
chisq.test(sample_data(qd.baseline)$COPD, sample_data(qd.baseline)
$Dementia)
chisq.test(sample_data(qd.baseline)$COPD, sample_data(qd.baseline)
$CVA.Stroke)
chisq.test(sample_data(qd.baseline)$COPD, sample_data(qd.baseline)
$DM)
chisq.test(sample_data(qd.baseline)$COPD, sample_data(qd.baseline)
$Hypothyroid)
summary(aov(as.numeric(Num_Comorbidities) ~ as.factor(COPD),
data=baseline.test))
chisq.test(sample_data(qd.baseline)$COPD, sample_data(qd.baseline)
```

```
$Seizures)
chisq.test(sample_data(qd.baseline)$COPD, sample_data(qd.baseline)
$Cancer)
summary(aov(as.numeric(IL1B) ~ as.factor(COPD), data=baseline.test))
summary(aov(as.numeric(IL6) ~ as.factor(COPD), data=baseline.test))
summary(aov(as.numeric(TNFA) ~ as.factor(COPD), data=baseline.test))
summary(aov(as.numeric(qPCR_Concentration) ~ as.factor(COPD),
data=baseline.test))

#CHF
chisq.test(sample_data(qd.baseline)$CHF, sample_data(qd.baseline)
$CAD)
chisq.test(sample_data(qd.baseline)$CHF, sample_data(qd.baseline)
$Anemia)
chisq.test(sample_data(qd.baseline)$CHF, sample_data(qd.baseline)
$Dementia)
chisq.test(sample_data(qd.baseline)$CHF, sample_data(qd.baseline)
$CVA.Stroke)
chisq.test(sample_data(qd.baseline)$CHF, sample_data(qd.baseline)
$DM)
chisq.test(sample_data(qd.baseline)$CHF, sample_data(qd.baseline)
$Hypothyroid)
summary(aov(as.numeric(Num_Comorbidities) ~ as.factor(CHF),
data=baseline.test))
###Significant, so:###
a1 <- aov(Num_Comorbidities ~ as.factor(CHF),
data=baseline.test.sup)
nd = data.frame(CHF = unique(baseline.test.sup$CHF))
pred = predict(a1, newdata = nd, se.fit = TRUE)
pred
nd$Pred = pred$fit # adds the predicted group means to the data
frame
nd$Lo = nd$Pred - 1.96*pred$se.fit # lower bound of the confidence
interval by subtracting 1.96*se from the prediction
nd$Hi = nd$Pred + 1.96*pred$se.fit # upper bound ditto
plt = ggplot(nd, aes(x = CHF)) +
  geom_point(aes(y = Pred), size=4) +
  geom_linerange(aes(ymin = Lo, ymax = Hi), alpha=0.5) +
  theme(axis.text.x=element_text(angle=90,hjust=1,vjust=0.5))
plt
p <- ggplot(baseline.test.sup, aes(x=CHF, y=Num_Comorbidities)) +
  geom_point() +
  geom_boxplot() +
  scale_y_log10()
p
###
chisq.test(sample_data(qd.baseline)$CHF, sample_data(qd.baseline)
$Seizures)
chisq.test(sample_data(qd.baseline)$CHF, sample_data(qd.baseline)
$Cancer)
summary(aov(as.numeric(IL1B) ~ as.factor(CHF), data=baseline.test))
summary(aov(as.numeric(IL6) ~ as.factor(CHF), data=baseline.test))
summary(aov(as.numeric(TNFA) ~ as.factor(CHF), data=baseline.test))
summary(aov(as.numeric(qPCR_Concentration) ~ as.factor(CHF),
```

```
data=baseline.test))

#CAD/CVD
chisq.test(sample_data(qd.baseline)$CAD, sample_data(qd.baseline)
$Anemia)
chisq.test(sample_data(qd.baseline)$CAD, sample_data(qd.baseline)
$Dementia)
chisq.test(sample_data(qd.baseline)$CAD, sample_data(qd.baseline)
$CVA.Stroke)
chisq.test(sample_data(qd.baseline)$CAD, sample_data(qd.baseline)
$DM)
chisq.test(sample_data(qd.baseline)$CAD, sample_data(qd.baseline)
$Hypothyroid)
summary(aov(as.numeric(Num_Comorbidities) ~ as.factor(CAD),
data=baseline.test))
###Significant, so:###
a1 <- aov(Num_Comorbidities ~ as.factor(CAD), data=baseline.test)
nd = data.frame(CAD = unique(baseline.test.sup$CAD))
pred = predict(a1, newdata = nd, se.fit = TRUE)
pred
nd$Pred = pred$fit # adds the predicted group means to the data
frame
nd$Lo = nd$Pred - 1.96*pred$se.fit # lower bound of the confidence
interval by subtracting 1.96*se from the prediction
nd$Hi = nd$Pred + 1.96*pred$se.fit # upper bound ditto
plt = ggplot(nd, aes(x = CAD)) +
  geom_point(aes(y = Pred), size=4) +
  geom_linerange(aes(ymin = Lo, ymax = Hi), alpha=0.5) +
  theme(axis.text.x=element_text(angle=90,hjust=1,vjust=0.5))
plt
p <- ggplot(baseline.test.sup, aes(x=CAD, y=Num_Comorbidities)) +
  geom_point() +
  geom_boxplot() +
  scale_y_log10()
p
###
chisq.test(sample_data(qd.baseline)$CAD, sample_data(qd.baseline)
$Seizures)
chisq.test(sample_data(qd.baseline)$CAD, sample_data(qd.baseline)
$Cancer)
summary(aov(as.numeric(IL1B) ~ as.factor(CAD), data=baseline.test))
summary(aov(as.numeric(IL6) ~ as.factor(CAD), data=baseline.test))
summary(aov(as.numeric(TNFA) ~ as.factor(CAD), data=baseline.test))
summary(aov(as.numeric(qPCR_Concentration) ~ as.factor(CAD),
data=baseline.test))

#Anemia
chisq.test(sample_data(qd.baseline)$Anemia, sample_data(qd.baseline)
$Dementia)
chisq.test(sample_data(qd.baseline)$Anemia, sample_data(qd.baseline)
$CVA.Stroke)
chisq.test(sample_data(qd.baseline)$Anemia, sample_data(qd.baseline)
$DM)
chisq.test(sample_data(qd.baseline)$Anemia, sample_data(qd.baseline)
```

```
$Hypothyroid)
summary(aov(as.numeric(Num_Comorbidities) ~ as.factor(Anemia),
data=baseline.test))
###Significant, so:###
a1 <- aov(Num_Comorbidities ~ as.factor(Anemia), data=baseline.test)
nd = data.frame(Anemia = unique(baseline.test.sup$Anemia))
pred = predict(a1, newdata = nd, se.fit = TRUE)
pred
nd$Pred = pred$fit # adds the predicted group means to the data
frame
nd$Lo = nd$Pred - 1.96*pred$se.fit # lower bound of the confidence
interval by subtracting 1.96*se from the prediction
nd$Hi = nd$Pred + 1.96*pred$se.fit # upper bound ditto
plt = ggplot(nd, aes(x = Anemia)) +
  geom_point(aes(y = Pred), size=4) +
  geom_linerange(aes(ymin = Lo, ymax = Hi), alpha=0.5) +
  theme(axis.text.x=element_text(angle=90,hjust=1,vjust=0.5))
plt
p <- ggplot(baseline.test.sup, aes(x=Anemia, y=Num_Comorbidities)) +
  geom_point() +
  geom_boxplot() +
  scale_y_log10()
p
###
chisq.test(sample_data(qd.baseline)$Anemia, sample_data(qd.baseline)
$Seizures)
qd.baseline.Test <- subset_samples(qd.baseline, Cancer != "Prior")
chisq.test(sample_data(qd.baseline.Test)$Anemia,
sample_data(qd.baseline.Test)$Cancer)
summary(aov(as.numeric(IL1B) ~ as.factor(Anemia),
data=baseline.test))
summary(aov(as.numeric(IL6) ~ as.factor(Anemia),
data=baseline.test))
summary(aov(as.numeric(TNFA) ~ as.factor(Anemia),
data=baseline.test))
summary(aov(as.numeric(qPCR_Concentration) ~ as.factor(Anemia),
data=baseline.test))

#Dementia
chisq.test(sample_data(qd.baseline)$Dementia,
sample_data(qd.baseline)$CVA.Stroke)
chisq.test(sample_data(qd.baseline)$Dementia,
sample_data(qd.baseline)$DM)
chisq.test(sample_data(qd.baseline)$Dementia,
sample_data(qd.baseline)$Hypothyroid)
summary(aov(as.numeric(Num_Comorbidities) ~ as.factor(Dementia),
data=baseline.test))
chisq.test(sample_data(qd.baseline)$Dementia,
sample_data(qd.baseline)$Seizures)
chisq.test(sample_data(qd.baseline)$Dementia,
sample_data(qd.baseline)$Cancer)
summary(aov(as.numeric(IL1B) ~ as.factor(Dementia),
data=baseline.test))
summary(aov(as.numeric(IL6) ~ as.factor(Dementia),
```

```
data=baseline.test))
###Significant, so:###
a1 <- aov(IL6 ~ as.factor(Dementia), data=baseline.test)
nd = data.frame(Dementia = unique(baseline.test.sup$Dementia))
pred = predict(a1, newdata = nd, se.fit = TRUE)
pred
nd$Pred = pred$fit # adds the predicted group means to the data
frame
nd$Lo = nd$Pred - 1.96*pred$se.fit # lower bound of the confidence
interval by subtracting 1.96*se from the prediction
nd$Hi = nd$Pred + 1.96*pred$se.fit # upper bound ditto
plt = ggplot(nd, aes(x = Dementia)) +
  geom_point(aes(y = Pred), size=4) +
  geom_linerange(aes(ymin = Lo, ymax = Hi), alpha=0.5) +
  theme(axis.text.x=element_text(angle=90,hjust=1,vjust=0.5))
plt
p <- ggplot(baseline.test.sup, aes(x=Dementia, y=IL6)) +
  geom_point() +
  geom_boxplot()
p
###
summary(aov(as.numeric(TNFA) ~ as.factor(Dementia),
data=baseline.test))
summary(aov(as.numeric(qPCR_Concentration) ~ as.factor(Dementia),
data=baseline.test))

#CVA.Stroke
qd.baseline.Test <- subset_samples(qd.baseline, CVA.Stroke !=
"Prior")
baseline.test.sup <- subset(baseline.test,
baseline.test$CVA.Stroke != "Prior")
chisq.test(sample_data(qd.baseline.Test)$CVA.Stroke,
sample_data(qd.baseline.Test)$DM)
chisq.test(sample_data(qd.baseline.Test)$CVA.Stroke,
sample_data(qd.baseline.Test)$Hypothyroid)
summary(aov(as.numeric(Num_Comorbidities) ~ as.factor(CVA.Stroke),
data=baseline.test.sup))
###Significant, so:###
a1 <- aov(Num_Comorbidities ~ as.factor(CVA.Stroke),
data=baseline.test)
nd = data.frame(CVA.Stroke = unique(baseline.test.sup$CVA.Stroke))
pred = predict(a1, newdata = nd, se.fit = TRUE)
pred
nd$Pred = pred$fit # adds the predicted group means to the data
frame
nd$Lo = nd$Pred - 1.96*pred$se.fit # lower bound of the confidence
interval by subtracting 1.96*se from the prediction
nd$Hi = nd$Pred + 1.96*pred$se.fit # upper bound ditto
plt = ggplot(nd, aes(x = CVA.Stroke)) +
  geom_point(aes(y = Pred), size=4) +
  geom_linerange(aes(ymin = Lo, ymax = Hi), alpha=0.5) +
  theme(axis.text.x=element_text(angle=90,hjust=1,vjust=0.5))
plt
p <- ggplot(baseline.test.sup, aes(x=CVA.Stroke,
```

```
y=Num_Comorbidities)) +
  geom_point() +
  geom_boxplot() +
  scale_y_log10()
p
###
chisq.test(sample_data(qd.baseline.Test)$CVA.Stroke,
sample_data(qd.baseline.Test)$Seizures)
chisq.test(sample_data(qd.baseline.Test)$CVA.Stroke,
sample_data(qd.baseline.Test)$Cancer)
summary(aov(as.numeric(IL1B) ~ as.factor(CVA.Stroke),
data=baseline.test.sup))
summary(aov(as.numeric(IL6) ~ as.factor(CVA.Stroke),
data=baseline.test.sup))
summary(aov(as.numeric(TNFA) ~ as.factor(CVA.Stroke),
data=baseline.test.sup))
summary(aov(as.numeric(qPCR_Concentration) ~ as.factor(CVA.Stroke),
data=baseline.test.sup))

#DM
chisq.test(sample_data(qd.baseline)$DM, sample_data(qd.baseline)
$Hypothyroid)
summary(aov(as.numeric(Num_Comorbidities) ~ as.factor(DM),
data=baseline.test))
###Significant, so:###
a1 <- aov(Num_Comorbidities ~ as.factor(DM), data=baseline.test)
nd = data.frame(DM = unique(baseline.test.sup$DM))
pred = predict(a1, newdata = nd, se.fit = TRUE)
pred
nd$Pred = pred$fit # adds the predicted group means to the data
frame
nd$Lo = nd$Pred - 1.96*pred$se.fit # lower bound of the confidence
interval by subtracting 1.96*se from the prediction
nd$Hi = nd$Pred + 1.96*pred$se.fit # upper bound ditto
plt = ggplot(nd, aes(x = DM)) +
  geom_point(aes(y = Pred), size=4) +
  geom_linerange(aes(ymin = Lo, ymax = Hi), alpha=0.5) +
  theme(axis.text.x=element_text(angle=90,hjust=1,vjust=0.5))
plt
p <- ggplot(baseline.test.sup, aes(x=DM, y=Num_Comorbidities)) +
  geom_point() +
  geom_boxplot() +
  scale_y_log10()
p
###
chisq.test(sample_data(qd.baseline)$DM, sample_data(qd.baseline)
$Seizures)
chisq.test(sample_data(qd.baseline)$DM, sample_data(qd.baseline)
$Cancer)
summary(aov(as.numeric(IL1B) ~ as.factor(DM), data=baseline.test))
summary(aov(as.numeric(IL6) ~ as.factor(DM), data=baseline.test))
summary(aov(as.numeric(TNFA) ~ as.factor(DM), data=baseline.test))
summary(aov(as.numeric(qPCR_Concentration) ~ as.factor(DM),
data=baseline.test))
```

```
#Hypothyroid
summary(aov(as.numeric(Num_Comorbidities) ~ as.factor(Hypothyroid),
data=baseline.test))
chisq.test(sample_data(qd.baseline)$Hypothyroid,
sample_data(qd.baseline)$Seizures)
chisq.test(sample_data(qd.baseline)$Hypothyroid,
sample_data(qd.baseline)$Cancer)
summary(aov(as.numeric(IL1B) ~ as.factor(Hypothyroid),
data=baseline.test))
summary(aov(as.numeric(IL6) ~ as.factor(Hypothyroid),
data=baseline.test))
summary(aov(as.numeric(TNFA) ~ as.factor(Hypothyroid),
data=baseline.test))
summary(aov(as.numeric(qPCR_Concentration) ~ as.factor(Hypothyroid),
data=baseline.test))

#Num_Comorbidities
summary(aov(as.numeric(Num_Comorbidities) ~ as.factor(Seizures),
data=baseline.test))
summary(aov(as.numeric(Num_Comorbidities) ~ as.factor(Cancer),
data=baseline.test))
summary(aov(as.numeric(Num_Comorbidities) ~ as.factor(IL1B),
data=baseline.test))
summary(aov(as.numeric(IL1B) ~ as.factor(Num_Comorbidities),
data=baseline.test))
summary(aov(as.numeric(IL6) ~ as.factor(Num_Comorbidities),
data=baseline.test))
summary(aov(as.numeric(TNFA) ~ as.factor(Num_Comorbidities),
data=baseline.test))
summary(aov(as.numeric(qPCR_Concentration) ~
as.factor(Num_Comorbidities), data=baseline.test))

#Seizures
qd.baseline.Test <- subset_samples(qd.baseline, Cancer != "Prior")
chisq.test(sample_data(qd.baseline.Test)$Seizures,
sample_data(qd.baseline.Test)$Cancer)
summary(aov(as.numeric(IL1B) ~ as.factor(Seizures),
data=baseline.test))
summary(aov(as.numeric(IL6) ~ as.factor(Seizures),
data=baseline.test))
summary(aov(as.numeric(TNFA) ~ as.factor(Seizures),
data=baseline.test))
summary(aov(as.numeric(qPCR_Concentration) ~ as.factor(Seizures),
data=baseline.test))

#Cancer
summary(aov(as.numeric(IL1B) ~ as.factor(Cancer),
data=baseline.test))
summary(aov(as.numeric(IL6) ~ as.factor(Cancer),
data=baseline.test))
summary(aov(as.numeric(TNFA) ~ as.factor(Cancer),
data=baseline.test))
summary(aov(as.numeric(qPCR_Concentration) ~ as.factor(Cancer),
```

```
data=baseline.test))

#IL1B
summary(aov(as.numeric(IL6) ~ as.factor(IL1B), data=baseline.test))
summary(aov(as.numeric(TNFA) ~ as.factor(IL1B), data=baseline.test))
summary(aov(as.numeric(qPCR_Concentration) ~ as.numeric(IL1B),
data=baseline.test))
###Significant, so:###
a1 <- aov(qPCR_Concentration ~ as.numeric(IL1B), data=baseline.test)
nd = data.frame(IL1B = unique(baseline.test.sup$IL1B))
pred = predict(a1, newdata = nd, se.fit = TRUE)
pred
nd$Pred = pred$fit # adds the predicted group means to the data
frame
nd$Lo = nd$Pred - 1.96*pred$se.fit # lower bound of the confidence
interval by subtracting 1.96*se from the prediction
nd$Hi = nd$Pred + 1.96*pred$se.fit # upper bound ditto
plt = ggplot(nd, aes(x = IL1B)) +
  geom_point(aes(y = Pred), size=4) +
  geom_linerange(aes(ymin = Lo, ymax = Hi), alpha=0.5) +
  theme(axis.text.x=element_text(angle=90,hjust=1,vjust=0.5))
plt
p <- ggplot(baseline.test.sup, aes(x=IL1B, y=qPCR_Concentration)) +
  geom_point() +
  scale_y_log10()
p
###
```

Statistics on metadata vars which pass the above criteria; plot results.

3. Setup universal vars qd.baseline.rare, df.baseline, d.baseline, dist.baseline, iMDS.baseline,
                     qd.healthy.rare, df.healthy, d.healthy, dist.healthy, iMDS.healthy

```{r}
#Baseline vars
qd.baseline.rare = rarefy_even_depth(qd.baseline, sample.size =
min(sample_sums(qd.baseline)), rngseed=1414)
df.baseline = as(sample_data(qd.baseline.rare), "data.frame")
dist.baseline <- phyloseq::distance(qd.baseline.rare, method =
"bray") #calc dist metrix
iMDS.baseline <- ordinate(qd.baseline.rare, "PCoA", distance =
dist.baseline)

qd.baseline.clr <- microbiome::transform(qd.baseline, "clr")
df.ait.baseline = as(sample_data(qd.baseline.clr), "data.frame")
dist.ait.baseline <- phyloseq::distance(qd.baseline.clr, method =
"euclidean")
iMDS.ait.baseline <- ordinate(qd.baseline.clr, method = "RDA",
distance = dist.ait.baseline)

myPalette <- colorRampPalette(rev(brewer.pal(11, "Spectral")))
```

```

4. Calculate anova & pcoas for baseline dataset and by cluster
```{r}
##Age_at_enrollment
a.Age = adonis(dist.baseline ~ Age_at_enrollment, df.baseline)
a.Age
ait.Age = adonis(dist.ait.baseline ~ Age_at_enrollment,
df.ait.baseline)
ait.Age

aov.Age <- aov(Age_at_enrollment ~ cluster, data=qd.baseline.clust)
summary(aov.Age)

##Sex
a.Sex = adonis(dist.baseline ~ Sex, df.baseline)
a.Sex
ait.Sex = adonis(dist.ait.baseline ~ Sex, df.ait.baseline)
ait.Sex

qd.baseline.clust$Sex[qd.baseline.clust$Sex=="Female"] <- 1
qd.baseline.clust$Sex[qd.baseline.clust$Sex=="Male"] <- 0
aov.Sex <- aov(Sex ~ cluster, data=qd.baseline.clust)
summary(aov.Sex)

##Site
#omit
qd.baseline.Site = subset_samples(qd.baseline, (Site != "" & Site !=
"")) #site names removed
qd.baseline.Site.rare = rarefy_even_depth(qd.baseline.Site,
sample.size = min(sample_sums(qd.baseline.Site)), rngseed=1414)
df.baseline.Site = as(sample_data(qd.baseline.Site.rare),
"data.frame")
dist.baseline.Site = phyloseq::distance(qd.baseline.Site.rare,
"bray")

qd.baseline.Site.clr <- microbiome::transform(qd.baseline.Site,
"clr")
df.ait.baseline.Site = as(sample_data(qd.baseline.Site.clr),
"data.frame")
dist.ait.baseline.Site <- phyloseq::distance(qd.baseline.Site.clr,
method = "euclidean")
iMDS.ait.baseline.Site <- ordinate(qd.baseline.Site.clr, method =
"RDA", distance = dist.ait.baseline.Site)

a.Site = adonis(dist.baseline.Site ~ Site, df.baseline.Site)
a.Site
ait.Site = adonis(dist.ait.baseline.Site ~ Site,
df.ait.baseline.Site)
ait.Site

#chi-squared
qd.baseline.clust1 <- qd.baseline.clust
qd.baseline.clust1 <- qd.baseline.clust1[qd.baseline.clust1$Site !=

```
"",]
qd.baseline.clust1 <- qd.baseline.clust1[qd.baseline.clust1$Site !=
"",]
tbl.Site <- table(qd.baseline.clust1$Site,
qd.baseline.clust1$cluster)
tbl.Site
chi.Site <- chisq.test(tbl.Site)
chi.Site

##Month
table(sample_data(qd.baseline)$Month)
qd.baseline.Month = subset_samples(qd.baseline, Month != "Feb")
qd.baseline.Month.rare = rarefy_even_depth(qd.baseline.Month,
sample.size = min(sample_sums(qd.baseline.Month)), rngseed=1414)
df.baseline.Month = as(sample_data(qd.baseline.Month.rare),
"data.frame")
d.baseline.Month = phyloseq::distance(qd.baseline.Month.rare,
"bray")

qd.baseline.Month.clr <- microbiome::transform(qd.baseline.Month,
"clr")
df.ait.baseline.Month = as(sample_data(qd.baseline.Month.clr),
"data.frame")
dist.ait.baseline.Month <- phyloseq::distance(qd.baseline.Month.clr,
method = "euclidean")
iMDS.ait.baseline.Month <- ordinate(qd.baseline.Month.clr, method =
"RDA", distance = dist.ait.baseline.Month)

a.Month = adonis(d.baseline.Month ~ Month, df.baseline.Month)
a.Month
ait.Month = adonis(dist.ait.baseline.Month ~ Month,
df.ait.baseline.Month)
ait.Month

qd.baseline.clust1 <- qd.baseline.clust
qd.baseline.clust1 <- qd.baseline.clust1[qd.baseline.clust1$Month !=
"Feb",]
tbl.Site <- table(qd.baseline.clust1$Month,
qd.baseline.clust1$cluster)
tbl.Site
chi.Site <- chisq.test(tbl.Site)
chi.Site

##Season
table(sample_data(qd.baseline)$Season)

a.Season = adonis(dist.baseline ~ Season, df.baseline)
a.Season
ait.Season = adonis(dist.ait.baseline ~ Season, df.ait.baseline)
ait.Season

qd.baseline.clust1 <- qd.baseline.clust
qd.baseline.clust1$Season[qd.baseline.clust1$Season=="Autumn"] <- 1
```

```
qd.baseline.clust1$Season[qd.baseline.clust1$Season=="Winter"] <- 2
aov.Season <- aov(Season ~ cluster, data=qd.baseline.clust1)
summary(aov.Season)

##Year
#omit 2017
table(sample_data(qd.baseline)$Year)
qd.baseline.Year = subset_samples(qd.baseline, Year !="2017")
qd.baseline.Year.rare = rarefy_even_depth(qd.baseline.Year,
sample.size = min(sample_sums(qd.baseline.Year)), rngseed=1414)
df.baseline.Year = as(sample_data(qd.baseline.Year.rare),
"data.frame")
dist.baseline.Year = phyloseq::distance(qd.baseline.Year.rare,
"bray")

qd.baseline.Year.clr <- microbiome::transform(qd.baseline.Year,
"clr")
df.ait.baseline.Year = as(sample_data(qd.baseline.Year.clr),
"data.frame")
dist.ait.baseline.Year <- phyloseq::distance(qd.baseline.Year.clr,
method = "euclidean")
iMDS.ait.baseline.Year <- ordinate(qd.baseline.Year.clr, method =
"RDA", distance = dist.ait.baseline.Year)

a.Year = adonis(dist.baseline.Year ~ Year, df.baseline.Year)
a.Year
ait.Year = adonis(dist.ait.baseline.Year ~ Year,
df.ait.baseline.Year)
ait.Year

#chi-squared
aov.Year <- aov(Year ~ cluster, data=qd.baseline.clust)
summary(aov.Year)

##Completed Study
a.CS = adonis(dist.baseline ~ Completed.Study, df.baseline)
a.CS
ait.CS = adonis(dist.ait.baseline ~ Completed.Study,
df.ait.baseline)
ait.CS
aov.CS <- aov(Completed.Study ~ cluster, data=qd.baseline.clust)
summary(aov.CS)

##Allocation_Group_Probiotics
a.AGP = adonis(dist.baseline ~ Allocation_Group_Probiotics,
df.baseline)
a.AGP
ait.AGP = adonis(dist.ait.baseline ~ Allocation_Group_Probiotics,
df.ait.baseline)
ait.AGP
qd.baseline.clust1 <- qd.baseline.clust
qd.baseline.clust1$Allocation_Group_Probiotics[qd.baseline.clust1$Al
location_Group_Probiotics=="Active"] <- 1
qd.baseline.clust1$Allocation_Group_Probiotics[qd.baseline.clust1$Al
```

```
location_Group_Probiotics=="Placebo"] <- 2
aov.AGP <- aov(Allocation_Group_Probiotics ~ cluster,
data=qd.baseline.clust1)
summary(aov.AGP)

##hadEvent
a.Event = adonis(dist.baseline ~ hadEvent, df.baseline)
a.Event
ait.Event = adonis(dist.ait.baseline ~ hadEvent, df.ait.baseline)
ait.Event
table(qd.baseline.clust$hadEvent)
qd.baseline.clust1 <- qd.baseline.clust
qd.baseline.clust1$hadEvent[qd.baseline.clust1$hadEvent=="T"] <- 1
qd.baseline.clust1$hadEvent[qd.baseline.clust1$hadEvent=="F"] <- 2
aov.Event <- aov(hadEvent ~ cluster, data=qd.baseline.clust1)
summary(aov.Event)

##Smoker
a.Smoker = adonis(dist.baseline ~ Smoker, df.baseline)
a.Smoker
ait.Smoker = adonis(dist.ait.baseline ~ Smoker, df.ait.baseline)
ait.Smoker

qd.baseline.clust1 <- qd.baseline.clust
qd.baseline.clust1 <- qd.baseline.clust1[qd.baseline.clust1$Smoker !
= "Unknown",]
unique(qd.baseline.clust1$Smoker)
qd.baseline.clust1$Smoker[qd.baseline.clust1$Smoker=="No"] <- 1
qd.baseline.clust1$Smoker[qd.baseline.clust1$Smoker=="Prior"] <- 2
qd.baseline.clust1$Smoker[qd.baseline.clust1$Smoker=="Yes"] <- 3
aov.Smoker <- aov(Smoker ~ cluster, data=qd.baseline.clust1)
summary(aov.Smoker)

##Num_Medications
table(sample_data(qd.baseline)$Num_Medications)

a.NumMeds = adonis(dist.baseline ~ Num_Medications, df.baseline)
a.NumMeds
ait.NumMeds = adonis(dist.ait.baseline ~ Num_Medications,
df.ait.baseline)
ait.NumMeds

qd.baseline.clust1 <- qd.baseline.clust
unique(qd.baseline.clust1$Num_Medications)
aov.NumMed <- aov(Num_Medications ~ cluster,
data=qd.baseline.clust1)
summary(aov.NumMed)

##Influenza_vacc_this_season
a.Flu = adonis(dist.baseline ~ Influenza_vacc_this_season,
df.baseline)
a.Flu
ait.Flu = adonis(dist.ait.baseline ~ Influenza_vacc_this_season,
df.ait.baseline)
```

```
ait.Flu
table(qd.baseline.clust$Influenza_vacc_this_season)
qd.baseline.clust1 <- qd.baseline.clust
qd.baseline.clust1$Influenza_vacc_this_season[qd.baseline.clust1$Inf
luenza_vacc_this_season=="No"] <- 1
qd.baseline.clust1$Influenza_vacc_this_season[qd.baseline.clust1$Inf
luenza_vacc_this_season=="Yes"] <- 2
aov.Flu <- aov(Influenza_vacc_this_season ~ cluster,
data=qd.baseline.clust1)
summary(aov.Flu)

##Influenza_seasonal_vaccine_last_season
table(sample_data(qd.baseline)
$Influenza_seasonal_vaccine_last_season)

a.Flulast = adonis(dist.baseline ~
Influenza_seasonal_vaccine_last_season, df.baseline)
a.Flulast
ait.Flulast = adonis(dist.ait.baseline ~
Influenza_seasonal_vaccine_last_season, df.ait.baseline)
ait.Flulast

qd.baseline.clust1 <- qd.baseline.clust
qd.baseline.clust1$Influenza_seasonal_vaccine_last_season[qd.baselin
e.clust1$Influenza_seasonal_vaccine_last_season=="Yes"] <- 1
qd.baseline.clust1$Influenza_seasonal_vaccine_last_season[qd.baselin
e.clust1$Influenza_seasonal_vaccine_last_season=="No"] <- 0
qd.baseline.clust1$Influenza_seasonal_vaccine_last_season[qd.baselin
e.clust1$Influenza_seasonal_vaccine_last_season=="Unknown"] <- 2
aov.Flulast <- aov(Influenza_seasonal_vaccine_last_season ~ cluster,
data=qd.baseline.clust1)
summary(aov.Flulast)

##Influenza_vaccine_ever
table(sample_data(qd.baseline)$Influenza_vaccine_ever)
a.Flulast = adonis(dist.baseline ~ Influenza_vaccine_ever,
df.baseline)
a.Flulast
ait.Flulast = adonis(dist.ait.baseline ~ Influenza_vaccine_ever,
df.ait.baseline)
ait.Flulast
qd.baseline.clust1 <- qd.baseline.clust
qd.baseline.clust1$Influenza_vaccine_ever[qd.baseline.clust1$Influen
za_vaccine_ever=="Yes"] <- 1
qd.baseline.clust1$Influenza_vaccine_ever[qd.baseline.clust1$Influen
za_vaccine_ever=="No"] <- 0
qd.baseline.clust1$Influenza_vaccine_ever[qd.baseline.clust1$Influen
za_vaccine_ever=="Unknown"] <- 2
aov.Flulast <- aov(Influenza_vaccine_ever ~ cluster,
data=qd.baseline.clust1)
summary(aov.Flulast)

##Has_pt_received_pneumonia_vaccine
table(sample_data(qd.baseline)$Has_pt_received_pneumonia_vaccine)
```

```
qd.baseline.Pneum = subset_samples(qd.baseline,
Has_pt_received_pneumonia_vaccine != "Unknown")
qd.baseline.Pneum.rare = rarefy_even_depth(qd.baseline.Pneum,
sample.size = min(sample_sums(qd.baseline.Pneum)), rngseed=1414)
df.baseline.Pneum = as(sample_data(qd.baseline.Pneum.rare),
"data.frame")
dist.baseline.Pneum = phyloseq::distance(qd.baseline.Pneum.rare,
"bray")

qd.baseline.Pneum.clr <- microbiome::transform(qd.baseline.Pneum,
"clr")
df.ait.baseline.Pneum = as(sample_data(qd.baseline.Pneum.clr),
"data.frame")
dist.ait.baseline.Pneum <- phyloseq::distance(qd.baseline.Pneum.clr,
method = "euclidean")
iMDS.ait.baseline.Pneum <- ordinate(qd.baseline.Pneum.clr, method =
"RDA", distance = dist.ait.baseline.Pneum)

a.Pneum = adonis(dist.baseline.Pneum ~
Has_pt_received_pneumonia_vaccine, df.baseline.Pneum)
a.Pneum
ait.Pneum = adonis(dist.ait.baseline.Pneum ~
Has_pt_received_pneumonia_vaccine, df.ait.baseline.Pneum)
ait.Pneum

qd.baseline.clust1 <- qd.baseline.clust
qd.baseline.clust1 <-
qd.baseline.clust1[qd.baseline.clust1$Has_pt_received_pneumonia_vacc
ine!="Unknown",]
unique(qd.baseline.clust1$Has_pt_received_pneumonia_vaccine)
qd.baseline.clust1$Has_pt_received_pneumonia_vaccine[qd.baseline.clu
st1$Has_pt_received_pneumonia_vaccine=="Yes"] <- 1
qd.baseline.clust1$Has_pt_received_pneumonia_vaccine[qd.baseline.clu
st1$Has_pt_received_pneumonia_vaccine=="No"] <- 0
aov.Pneum <- aov(Has_pt_received_pneumonia_vaccine ~ cluster,
data=qd.baseline.clust1)
summary(aov.Pneum)

##Is_pt_in_shared_room
table(sample_data(qd.baseline)$Is_pt_in_shared_room)
qd.baseline.Room = subset_samples(qd.baseline,
Is_pt_in_shared_room != ".")
qd.baseline.Room.rare = rarefy_even_depth(qd.baseline.Room,
sample.size = min(sample_sums(qd.baseline.Room)), rngseed=1414)
df.baseline.Room = as(sample_data(qd.baseline.Room.rare),
"data.frame")
dist.baseline.Room = phyloseq::distance(qd.baseline.Room.rare,
"bray")

qd.baseline.Room.clr <- microbiome::transform(qd.baseline.Room,
"clr")
df.ait.baseline.Room = as(sample_data(qd.baseline.Room.clr),
"data.frame")
dist.ait.baseline.Room <- phyloseq::distance(qd.baseline.Room.clr,
```

```
method = "euclidean")
iMDS.ait.baseline.Room <- ordinate(qd.baseline.Room.clr, method =
"RDA", distance = dist.ait.baseline.Room)

a.Room = adonis(dist.baseline.Room ~ Is_pt_in_shared_room,
df.baseline.Room)
a.Room
ait.Room = adonis(dist.ait.baseline.Room ~ Is_pt_in_shared_room,
df.ait.baseline.Room)
ait.Room

qd.baseline.clust1 <- qd.baseline.clust
qd.baseline.clust1 <-
qd.baseline.clust1[qd.baseline.clust1$Is_pt_in_shared_room!=".",]
unique(qd.baseline.clust1$Is_pt_in_shared_room)
qd.baseline.clust1$Is_pt_in_shared_room[qd.baseline.clust1$Is_pt_in_
shared_room=="Yes"] <- 1
qd.baseline.clust1$Is_pt_in_shared_room[qd.baseline.clust1$Is_pt_in_
shared_room=="No"] <- 0
aov.Room <- aov(Is_pt_in_shared_room ~ cluster,
data=qd.baseline.clust1)
summary(aov.Room)

##Barthel_Total
table(sample_data(qd.baseline)$Barthel_Total)

a.Barthel = adonis(dist.baseline ~ Barthel_Total, df.baseline)
a.Barthel
ait.Barthel = adonis(dist.ait.baseline ~ Barthel_Total,
df.ait.baseline)
ait.Barthel

qd.baseline.clust1 <- qd.baseline.clust
qd.baseline.clust1 <- qd.baseline.clust1[!
is.na(qd.baseline.clust1$Barthel_Total),]
unique(qd.baseline.clust1$Barthel_Total)
unique(qd.baseline.clust1$Swab)
aov.Barthel <- aov(Barthel_Total ~ cluster, data=qd.baseline.clust1)
summary(aov.Barthel)

##COPD
table(sample_data(qd.baseline)$COPD)

a.COPD = adonis(dist.baseline ~ COPD, df.baseline)
a.COPD
ait.COPD = adonis(dist.ait.baseline ~ COPD, df.ait.baseline)
ait.COPD

qd.baseline.clust1 <- qd.baseline.clust
unique(qd.baseline.clust1$COPD)
qd.baseline.clust1$COPD[qd.baseline.clust1$COPD=="Yes"] <- 1
qd.baseline.clust1$COPD[qd.baseline.clust1$COPD=="No"] <- 0
aov.COPD <- aov(COPD ~ cluster, data=qd.baseline.clust1)
summary(aov.COPD)
```

```
##CHF
table(sample_data(qd.baseline)$CHF)
a.CHF = adonis(dist.baseline ~ CHF, df.baseline)
a.CHF
ait.CHF = adonis(dist.ait.baseline ~ CHF, df.ait.baseline)
ait.CHF
qd.baseline.clust1 <- qd.baseline.clust
qd.baseline.clust1$CHF[qd.baseline.clust1$CHF=="Yes"] <- 1
qd.baseline.clust1$CHF[qd.baseline.clust1$CHF=="No"] <- 0
aov.CHF <- aov(CHF ~ cluster, data=qd.baseline.clust1)
summary(aov.CHF)

##CAD
table(sample_data(qd.baseline)$CAD)

a.CAD = adonis(dist.baseline ~ CAD, df.baseline)
a.CAD
ait.CAD = adonis(dist.ait.baseline ~ CAD, df.ait.baseline)
ait.CAD

qd.baseline.clust1 <- qd.baseline.clust
qd.baseline.clust1$CAD[qd.baseline.clust1$CAD=="Yes"] <- 1
qd.baseline.clust1$CAD[qd.baseline.clust1$CAD=="No"] <- 0
aov.CAD <- aov(CAD ~ cluster, data=qd.baseline.clust1)
summary(aov.CAD)

##Anemia
table(sample_data(qd.baseline)$Anemia)

a.Anemia = adonis(dist.baseline ~ Anemia, df.baseline)
a.Anemia
ait.Anemia = adonis(dist.ait.baseline ~ Anemia, df.ait.baseline)
ait.Anemia

qd.baseline.clust1 <- qd.baseline.clust
qd.baseline.clust1$Anemia[qd.baseline.clust1$Anemia=="Yes"] <- 1
qd.baseline.clust1$Anemia[qd.baseline.clust1$Anemia=="No"] <- 0
aov.Anemia <- aov(Anemia ~ cluster, data=qd.baseline.clust1)
summary(aov.Anemia)

##Dementia
table(sample_data(qd.baseline)$Dementia)

a.Dementia = adonis(dist.baseline ~ Dementia, df.baseline)
a.Dementia
ait.Dementia = adonis(dist.ait.baseline ~ Dementia, df.ait.baseline)
ait.Dementia

qd.baseline.clust1 <- qd.baseline.clust
qd.baseline.clust1$Dementia[qd.baseline.clust1$Dementia=="Yes"] <- 1
qd.baseline.clust1$Dementia[qd.baseline.clust1$Dementia=="No"] <- 0
aov.Dementia <- aov(Dementia ~ cluster, data=qd.baseline.clust1)
summary(aov.Dementia)
```

```
##CVA.Stroke
table(sample_data(qd.baseline)$CVA.Stroke)
qd.baseline.CVA.Stroke = subset_samples(qd.baseline, CVA.Stroke !=
"Prior")
qd.baseline.CVA.Stroke.rare =
rarefy_even_depth(qd.baseline.CVA.Stroke, sample.size =
min(sample_sums(qd.baseline.CVA.Stroke)), rngseed=1414)
df.baseline.CVA.Stroke =
as(sample_data(qd.baseline.CVA.Stroke.rare), "data.frame")
dist.baseline.CVA.Stroke =
phyloseq::distance(qd.baseline.CVA.Stroke.rare, "bray")

qd.baseline.CVA.Stroke.clr <-
microbiome::transform(qd.baseline.CVA.Stroke, "clr")
df.ait.baseline.CVA.Stroke =
as(sample_data(qd.baseline.CVA.Stroke.clr), "data.frame")
dist.ait.baseline.CVA.Stroke <-
phyloseq::distance(qd.baseline.CVA.Stroke.clr, method = "euclidean")
iMDS.ait.baseline.CVA.Stroke <- ordinate(qd.baseline.CVA.Stroke.clr,
method = "RDA", distance = dist.ait.baseline.CVA.Stroke)

a.CVA.Stroke = adonis(dist.baseline.CVA.Stroke ~ CVA.Stroke,
df.baseline.CVA.Stroke)
a.CVA.Stroke
ait.CVA.Stroke = adonis(dist.ait.baseline.CVA.Stroke ~ CVA.Stroke,
df.ait.baseline.CVA.Stroke)
ait.CVA.Stroke

qd.baseline.clust1 <- qd.baseline.clust
qd.baseline.clust1 <- subset(qd.baseline.clust1,
qd.baseline.clust1$CVA.Stroke != "Prior")
qd.baseline.clust1$CVA.Stroke[qd.baseline.clust1$CVA.Stroke=="Yes"]
<- 1
qd.baseline.clust1$CVA.Stroke[qd.baseline.clust1$CVA.Stroke=="No"]
<- 0
aov.CVA.Stroke <- aov(CVA.Stroke ~ cluster, data=qd.baseline.clust1)
summary(aov.CVA.Stroke)

##DM
table(sample_data(qd.baseline)$DM)

a.DM = adonis(dist.baseline ~ DM, df.baseline)
a.DM
ait.DM = adonis(dist.ait.baseline ~ DM, df.ait.baseline)
ait.DM

qd.baseline.clust1 <- qd.baseline.clust
qd.baseline.clust1$DM[qd.baseline.clust1$DM=="Yes"] <- 1
qd.baseline.clust1$DM[qd.baseline.clust1$DM=="No"] <- 0
aov.DM <- aov(DM ~ cluster, data=qd.baseline.clust1)
summary(aov.DM)

##Hypothyroid
```

```
table(sample_data(qd.baseline)$Hypothyroid)

a.Hypothyroid = adonis(dist.baseline ~ Hypothyroid, df.baseline)
a.Hypothyroid
ait.Hypothyroid = adonis(dist.ait.baseline ~ Hypothyroid,
df.ait.baseline)
ait.Hypothyroid

qd.baseline.clust1 <- qd.baseline.clust
qd.baseline.clust1$Hypothyroid[qd.baseline.clust1$Hypothyroid=="Yes"
] <- 1
qd.baseline.clust1$Hypothyroid[qd.baseline.clust1$Hypothyroid=="No"]
<- 0
aov.Hypothyroid <- aov(Hypothyroid ~ cluster,
data=qd.baseline.clust1)
summary(aov.Hypothyroid)

##Num_Comorbidities
table(sample_data(qd.baseline)$Num_Comorbidities)

a.Num_Comorbidities = adonis(dist.baseline ~ Num_Comorbidities,
df.baseline)
a.Num_Comorbidities
ait.Num_Comorbidities = adonis(dist.ait.baseline ~
Num_Comorbidities, df.ait.baseline)
ait.Num_Comorbidities

qd.baseline.clust1 <- qd.baseline.clust
qd.baseline.clust1 <- qd.baseline.clust1[!
is.na(qd.baseline.clust1$Num_Comorbidities),]
unique(qd.baseline.clust1$Num_Comorbidities)
aov.Num_Comorbidities <- aov(Num_Comorbidities ~ cluster,
data=qd.baseline.clust1)
summary(aov.Num_Comorbidities)

##Seizures
table(sample_data(qd.baseline)$Seizures)
a.Seizures = adonis(dist.baseline ~ Seizures, df.baseline)
a.Seizures
ait.Seizures = adonis(dist.ait.baseline ~ Seizures, df.ait.baseline)
ait.Seizures
qd.baseline.clust1 <- qd.baseline.clust
qd.baseline.clust1$Seizures[qd.baseline.clust1$Seizures=="Yes"] <- 1
qd.baseline.clust1$Seizures[qd.baseline.clust1$Seizures=="No"] <- 0
aov.Seizures <- aov(Seizures ~ cluster, data=qd.baseline.clust1)
summary(aov.Seizures)

##Cancer
table(sample_data(qd.baseline)$Cancer)
qd.baseline.Cancer = subset_samples(qd.baseline, Cancer != "Prior")
qd.baseline.Cancer.rare = rarefy_even_depth(qd.baseline.Cancer,
sample.size = min(sample_sums(qd.baseline.Cancer)), rngseed=1414)
df.baseline.Cancer = as(sample_data(qd.baseline.Cancer.rare),
"data.frame")
```

```
dist.baseline.Cancer = phyloseq::distance(qd.baseline.Cancer.rare,
"bray")

qd.baseline.Cancer.clr <- microbiome::transform(qd.baseline.Cancer,
"clr")
df.ait.baseline.Cancer = as(sample_data(qd.baseline.Cancer.clr),
"data.frame")
dist.ait.baseline.Cancer <-
phyloseq::distance(qd.baseline.Cancer.clr, method = "euclidean")
iMDS.ait.baseline.Cancer <- ordinate(qd.baseline.Cancer.clr, method
= "RDA", distance = dist.ait.baseline.Cancer)

a.Cancer = adonis(dist.baseline.Cancer ~ Cancer, df.baseline.Cancer)
a.Cancer
ait.Cancer = adonis(dist.ait.baseline.Cancer ~ Cancer,
df.ait.baseline.Cancer)
ait.Cancer

qd.baseline.clust1 <- qd.baseline.clust
qd.baseline.clust1 <- subset(qd.baseline.clust1,
qd.baseline.clust1$Cancer != "Prior")
qd.baseline.clust1$Cancer[qd.baseline.clust1$Cancer=="Yes"] <- 1
qd.baseline.clust1$Cancer[qd.baseline.clust1$Cancer=="No"] <- 0
aov.Cancer <- aov(Cancer ~ cluster, data=qd.baseline.clust1)
summary(aov.Cancer)

##IL1B
table(sample_data(qd.baseline)$IL1B)
qd.baseline.IL1B = subset_samples(qd.baseline, !is.na(IL1B))
qd.baseline.IL1B.rare = rarefy_even_depth(qd.baseline.IL1B,
sample.size = min(sample_sums(qd.baseline.IL1B)), rngseed=1414)
df.baseline.IL1B = as(sample_data(qd.baseline.IL1B.rare),
"data.frame")
dist.baseline.IL1B = phyloseq::distance(qd.baseline.IL1B.rare,
"bray")

qd.baseline.IL1B.clr <- microbiome::transform(qd.baseline.IL1B,
"clr")
df.ait.baseline.IL1B = as(sample_data(qd.baseline.IL1B.clr),
"data.frame")
dist.ait.baseline.IL1B <- phyloseq::distance(qd.baseline.IL1B.clr,
method = "euclidean")
iMDS.ait.baseline.IL1B <- ordinate(qd.baseline.IL1B.clr, method =
"RDA", distance = dist.ait.baseline.IL1B)

a.IL1B = adonis(dist.baseline.IL1B ~ IL1B, df.baseline.IL1B)
a.IL1B
ait.IL1B = adonis(dist.ait.baseline.IL1B ~ IL1B,
df.ait.baseline.IL1B)
ait.IL1B

qd.baseline.clust1 <- qd.baseline.clust
qd.baseline.clust1 <- qd.baseline.clust1[!
is.na(qd.baseline.clust1$IL1B),]
```

```
unique(qd.baseline.clust1$IL1B)
aov.IL1B <- aov(IL1B ~ cluster, data=qd.baseline.clust1)
summary(aov.IL1B)

##IL6
sample_data(qd.baseline)$IL6
qd.baseline.IL6 = subset_samples(qd.baseline, !is.na(IL6))
qd.baseline.IL6.rare = rarefy_even_depth(qd.baseline.IL6,
sample.size = min(sample_sums(qd.baseline.IL6)), rngseed=1414)
df.baseline.IL6 = as(sample_data(qd.baseline.IL6.rare),
"data.frame")
dist.baseline.IL6 = phyloseq::distance(qd.baseline.IL6.rare, "bray")

qd.baseline.IL6.clr <- microbiome::transform(qd.baseline.IL6, "clr")
df.ait.baseline.IL6 = as(sample_data(qd.baseline.IL6.clr),
"data.frame")
dist.ait.baseline.IL6 <- phyloseq::distance(qd.baseline.IL6.clr,
method = "euclidean")
iMDS.ait.baseline.IL6 <- ordinate(qd.baseline.IL6.clr, method =
"RDA", distance = dist.ait.baseline.IL6)

a.IL6 = adonis(dist.baseline.IL6 ~ IL6, df.baseline.IL6)
a.IL6
ait.IL6 = adonis(dist.ait.baseline.IL6 ~ IL6, df.ait.baseline.IL6)
ait.IL6

qd.baseline.clust1 <- qd.baseline.clust
qd.baseline.clust1 <- qd.baseline.clust1[!
is.na(qd.baseline.clust1$IL6),]
unique(qd.baseline.clust1$IL6)
aov.IL6 <- aov(IL6 ~ cluster, data=qd.baseline.clust1)
summary(aov.IL6)

##TNFA
sample_data(qd.baseline)$TNFA
qd.baseline.TNFA = subset_samples(qd.baseline, !is.na(TNFA))
qd.baseline.TNFA.rare = rarefy_even_depth(qd.baseline.TNFA,
sample.size = min(sample_sums(qd.baseline.TNFA)), rngseed=1414)
df.baseline.TNFA = as(sample_data(qd.baseline.TNFA.rare),
"data.frame")
dist.baseline.TNFA = phyloseq::distance(qd.baseline.TNFA.rare,
"bray")

qd.baseline.TNFA.clr <- microbiome::transform(qd.baseline.TNFA,
"clr")
df.ait.baseline.TNFA = as(sample_data(qd.baseline.TNFA.clr),
"data.frame")
dist.ait.baseline.TNFA <- phyloseq::distance(qd.baseline.TNFA.clr,
method = "euclidean")
iMDS.ait.baseline.TNFA <- ordinate(qd.baseline.TNFA.clr, method =
"RDA", distance = dist.ait.baseline.TNFA)

a.TNFA = adonis(dist.baseline.TNFA ~ TNFA, df.baseline.TNFA)
a.TNFA
```

```
ait.TNFA = adonis(dist.ait.baseline.TNFA ~ TNFA,
df.ait.baseline.TNFA)
ait.TNFA

qd.baseline.clust1 <- qd.baseline.clust
qd.baseline.clust1 <- qd.baseline.clust1[!
is.na(qd.baseline.clust1$TNFA),]
unique(qd.baseline.clust1$TNFA)
aov.TNFA <- aov(TNFA ~ cluster, data=qd.baseline.clust1)
summary(aov.TNFA)

##qPCR_Concentration
sample_data(qd.baseline)$qPCR_Concentration

a.qPCR_Concentration = adonis(dist.baseline ~ qPCR_Concentration,
df.baseline)
a.qPCR_Concentration
ait.qPCR_Concentration = adonis(dist.ait.baseline ~
qPCR_Concentration, df.ait.baseline)
ait.qPCR_Concentration

qd.baseline.clust1 <- qd.baseline.clust
qd.baseline.clust1 <- qd.baseline.clust1[!
is.na(qd.baseline.clust1$qPCR_Concentration),]
unique(qd.baseline.clust1$qPCR_Concentration)
aov.qPCR_Concentration <- aov(qPCR_Concentration ~ cluster,
data=qd.baseline.clust1)
summary(aov.qPCR_Concentration)
```

Figure 2: Metadata vars correlating with mb (qd.baseline & qd.healthy)

A. Calculate pcoa plots for significant vars; see calcs for Supp Table 1

```{r}
##Sex
pb.Sex <- plot_ordination(qd.baseline.rare, axes=c(1,2), iMDS.all) +
  ggtitle("Sex: p=0.035*") +
  geom_point(size = 2, colour="gray") +
  geom_point(size = 3, aes(colour=Sex)) +
  theme_bw() +
  theme(legend.title=element_blank(),
        legend.position = c(0.9,0.1)) +
  scale_colour_manual(na.translate = F,
                      values=c("#ca0020","#0571b0"), labels=c(
    paste("Female (", length(which(sample_data(qd.baseline)$Sex ==
"Female")), ")", sep=""),
    paste("Male (", length(which(sample_data(qd.baseline)$Sex ==
"Male")), ")", sep="")))
pb.Sex

pb.ait.Sex <- plot_ordination(qd.baseline.clr, axes=c(1,2),
iMDS.ait.all) +
```

```
  ggtitle("Sex: p=0.149") +
  geom_point(size = 2, colour="gray") +
  geom_point(size = 3, aes(colour=Sex)) +
  theme_bw() +
  theme(legend.title=element_blank(),
        legend.position = c(0.9,0.1)) +
  scale_colour_manual(values=c("#ca0020","#0571b0"), labels=c(
    paste("Female (", length(which(sample_data(qd.baseline)$Sex ==
"Female")), ")", sep=""),
    paste("Male (", length(which(sample_data(qd.baseline)$Sex ==
"Male")), ")", sep="")))
pb.ait.Sex

##Site
#omit
qd.baseline.Site = subset_samples(qd.baseline, (Site != "" & Site !=
"")) #site names removed
qd.baseline.Site.rare = rarefy_even_depth(qd.baseline.Site,
sample.size = min(sample_sums(qd.baseline.Site)), rngseed=1414)
qd.baseline.Site.clr <- microbiome::transform(qd.baseline.Site,
"clr")

pb.Site <- plot_ordination(qd.baseline.Site.rare, axes=c(1,2),
iMDS.all) +
  ggtitle(paste("Site: p=0.033*")) +
  geom_point(size = 2, colour="gray") +
  geom_point(size = 3, aes(colour=Site)) +
  theme_bw() +
  theme(legend.title=element_blank()) +
        #text = element_text(size=20)) +
  scale_colour_manual(na.translate = F,
                      values=c("" = "#e6194b",
                               "" = "#ffe119",
                               "" = "#46f0f0",
                               "" = "#f032e6",
                               "" = "#d2f53c",
                               "" = "#fabebe",
                               "" = "#008080",
                               "" = "#e6beff",
                               "" = "#aa6e28",
                               "" = "#fffac8",
                               "" = "#800000"), labels=c(
    paste("A (", length(which(sample_data(qd.baseline)$Site == "")),
")", sep=""),
    paste("B (", length(which(sample_data(qd.baseline)$Site == "")),
")", sep=""),
    paste("C (", length(which(sample_data(qd.baseline)$Site == "")),
")", sep=""),
    paste("D (", length(which(sample_data(qd.baseline)$Site == "")),
")", sep=""),
    paste("E (", length(which(sample_data(qd.baseline)$Site == "")),
")", sep=""),
    paste("F (", length(which(sample_data(qd.baseline)$Site == "")),
")", sep=""),
```

```
    paste("G (", length(which(sample_data(qd.baseline)$Site == "")),
")", sep=""),
    paste("H (", length(which(sample_data(qd.baseline)$Site == "")),
")", sep=""),
    paste("I (", length(which(sample_data(qd.baseline)$Site == "")),
")", sep=""),
    paste("J (", length(which(sample_data(qd.baseline)$Site == "")),
")", sep="")))
pb.Site

pb.ait.Site <- plot_ordination(qd.baseline.Site.clr, axes=c(1,2),
iMDS.ait.all) +
  ggtitle(paste("Site: p=0.084")) +
  geom_point(size = 2, colour="gray") +
  geom_point(size = 3, aes(colour=Site)) +
  theme_bw() +
  theme(legend.title=element_blank()) +
  scale_colour_manual(values=c("" = "#e6194b",
                               "" = "#ffe119",
                               "" = "#46f0f0",
                               "" = "#f032e6",
                               "" = "#d2f53c",
                               "" = "#fabebe",
                               "" = "#008080",
                               "" = "#e6beff",
                               "" = "#aa6e28",
                               "" = "#fffac8",
                               "" = "#800000"), labels=c(
    paste("A (", length(which(sample_data(qd.baseline)$Site == "")),
")", sep=""),
    paste("B (", length(which(sample_data(qd.baseline)$Site == "")),
")", sep=""),
    paste("C (", length(which(sample_data(qd.baseline)$Site == "")),
")", sep=""),
    paste("D (", length(which(sample_data(qd.baseline)$Site == "")),
")", sep=""),
    paste("E (", length(which(sample_data(qd.baseline)$Site == "")),
")", sep=""),
    paste("F (", length(which(sample_data(qd.baseline)$Site == "")),
")", sep=""),
    paste("G (", length(which(sample_data(qd.baseline)$Site == "")),
")", sep=""),
    paste("H (", length(which(sample_data(qd.baseline)$Site == "")),
")", sep=""),
    paste("I (", length(which(sample_data(qd.baseline)$Site == "")),
")", sep=""),
    paste("J (", length(which(sample_data(qd.baseline)$Site == "")),
")", sep="")))
pb.ait.Site

##Month
#omit Feb
qd.baseline.Month = subset_samples(qd.baseline, (Month != "Feb"))
qd.baseline.Month.rare = rarefy_even_depth(qd.baseline.Month,
```

```
sample.size = min(sample_sums(qd.baseline.Month)), rngseed=1414)
qd.baseline.Month.clr <- microbiome::transform(qd.baseline.Month,
"clr")
pb.Month <- plot_ordination(qd.baseline.Month.rare, axes=c(1,2),
iMDS.all) +
  ggtitle("Month: p=0.699") +
  theme_bw() +
  theme(legend.title=element_blank(),
        legend.position = c(0.9,0.1)) +
  geom_point(size = 2, colour = "gray") +
  geom_point(size = 3, aes(colour=Month)) +
  scale_colour_manual(values=c("#fdae61","#1a9641", "red", "blue"),
labels=c(
    paste("Oct (", length(which(sample_data(qd.baseline)$Month ==
"Oct")), ")", sep=""),
    paste("Nov (", length(which(sample_data(qd.baseline)$Month ==
"Nov")), ")", sep=""),
    paste("Dec (", length(which(sample_data(qd.baseline)$Month ==
"Dec")), ")", sep=""),
    paste("Jan (", length(which(sample_data(qd.baseline)$Month ==
"Jan")), ")", sep="")))
pb.Month

pb.ait.Month <- plot_ordination(qd.baseline.Month.rare, axes=c(1,2),
iMDS.ait.all) +
  ggtitle("Month: p=0.001***") +
  theme_bw() +
  theme(legend.title=element_blank(),
        legend.position = c(0.9,0.1)) +
  geom_point(size = 2, colour = "gray") +
  geom_point(size = 3, aes(colour=Month)) +
  scale_colour_manual(values=c("#fdae61","#1a9641", "red", "blue"),
labels=c(
    paste("Oct (", length(which(sample_data(qd.baseline)$Month ==
"Oct")), ")", sep=""),
    paste("Nov (", length(which(sample_data(qd.baseline)$Month ==
"Nov")), ")", sep=""),
    paste("Dec (", length(which(sample_data(qd.baseline)$Month ==
"Dec")), ")", sep=""),
    paste("Jan (", length(which(sample_data(qd.baseline)$Month ==
"Jan")), ")", sep="")))
pb.ait.Month

##Season
pb.Season <- plot_ordination(qd.baseline.rare, axes=c(1,2),
iMDS.all) +
  ggtitle("Season: p=0.966") +
  theme_bw() +
  theme(legend.title=element_blank(),
        legend.position = c(0.9,0.1)) +
  geom_point(size = 2, colour = "gray") +
  geom_point(size = 3, aes(colour=Season)) +
  scale_colour_manual(values=c("#fdae61","#1a9641"), labels=c(
    paste("Autumn (", length(which(sample_data(qd.baseline)$Season
```

```
== "Autumn")), ")", sep=""),
    paste("Winter (", length(which(sample_data(qd.baseline)$Season
== "Winter")), ")", sep="")))
pb.Season

pb.ait.Season <- plot_ordination(qd.baseline.rare, axes=c(1,2),
iMDS.ait.all) +
  ggtitle("Season: p=0.004**") +
  theme_bw() +
  theme(legend.title=element_blank(),
        legend.position = c(0.9,0.1)) +
  geom_point(size = 2, colour = "gray") +
  geom_point(size = 3, aes(colour=Season)) +
  scale_colour_manual(values=c("#fdae61","#1a9641"), labels=c(
    paste("Autumn (", length(which(sample_data(qd.baseline)$Season
== "Autumn")), ")", sep=""),
    paste("Winter (", length(which(sample_data(qd.baseline)$Season
== "Winter")), ")", sep="")))
pb.ait.Season

##Year
#omit 2017
qd.baseline.Year = subset_samples(qd.baseline, (Year != "2017"))
qd.baseline.Year.rare = rarefy_even_depth(qd.baseline.Year,
sample.size = min(sample_sums(qd.baseline.Year)), rngseed=1414)
qd.baseline.Year.clr <- microbiome::transform(qd.baseline.Year,
"clr")
pb.Year <- plot_ordination(qd.baseline.Year.rare, axes=c(1,2),
iMDS.all) +
  ggtitle(paste("Year: p=0.204")) +
  theme_bw() +
  theme(legend.title=element_blank(),
        legend.position = c(0.9,0.85)) +
        #text = element_text(size=20)) +
  geom_point(size = 2, colour = "gray") +
  geom_point(size = 3, aes(colour=Year)) +
  scale_colour_manual(na.translate = F,
                      values=c("2014" = "#4daf4a",
                               "2015" = "#984ea3",
                               "2016" = "#ff7f00"), labels=c(
    paste("2014 (", length(which(sample_data(qd.baseline)$Year ==
"2014")), ")", sep=""),
    paste("2015 (", length(which(sample_data(qd.baseline)$Year ==
"2015")), ")", sep=""),
    paste("2016 (", length(which(sample_data(qd.baseline)$Year ==
"2016")), ")", sep="")))
pb.Year

pb.ait.Year <- plot_ordination(qd.baseline.Year.clr, axes=c(1,2),
iMDS.ait.all) +
  ggtitle(paste("Year: p=0.001***")) +
  theme_bw() +
  theme(legend.title=element_blank(),
        legend.position = c(0.95,0.2)) +
```

```
  geom_point(size = 2, colour = "gray") +
  geom_point(size = 3, aes(colour=Year)) +
  scale_colour_manual(values=c("2014" = "#4daf4a",
                               "2015" = "#984ea3",
                               "2016" = "#ff7f00"), labels=c(
    paste("2014 (", length(which(sample_data(qd.baseline.clr)$Year
== "2014")), ")", sep=""),
    paste("2015 (", length(which(sample_data(qd.baseline.clr)$Year
== "2015")), ")", sep=""),
    paste("2016 (", length(which(sample_data(qd.baseline.clr)$Year
== "2016")), ")", sep="")))
pb.ait.Year

##Barthel_Total
size.data <- plot_ordination(qd.baseline.rare, axes=c(1,2),
iMDS.all, justDF = TRUE)
size.data$size1 <- ifelse(size.data$Type.B.E.F.== "B" |
size.data$Swab == "1", 3,2)
size.data$Barthel_Total <- ifelse(size.data$size1 == 3,
size.data$Barthel_Total,NA)

pb.Barthel <- plot_ordination(qd.baseline.rare, axes=c(1,2),
iMDS.all) +
  ggtitle(paste("Barthel (Total): p=0.121")) +
  theme_bw() +
  theme(legend.title=element_blank(),
        legend.position=c(0.95,0.2)) +
  geom_point(size = 2, colour = "gray") +
  geom_point(data = size.data, aes(size = size1,
colour=Barthel_Total), show.legend = F) +
  scale_size_continuous(breaks = 2:3, range = c(2,3)) +
  scale_colour_gradientn(colours = myPalette(100), limits=c(0,22),
na.value = "gray")
pb.Barthel

size.data <- plot_ordination(qd.baseline.rare, axes=c(1,2),
iMDS.ait.all, justDF = TRUE)
size.data$size1 <- ifelse(size.data$Type.B.E.F.== "B" |
size.data$Swab == "1", 3,2)
size.data$Barthel_Total <- ifelse(size.data$size1 == 3,
size.data$Barthel_Total,NA)

pb.ait.Barthel <- plot_ordination(qd.baseline.clr, axes=c(1,2),
iMDS.ait.all) +
  ggtitle(paste("Barthel (Total): p=0.003**")) +
  theme_bw() +
  theme(legend.title=element_blank(),
        legend.position=c(0.95,0.2)) +
  geom_point(size = 2, colour = "gray") +
  geom_point(data = size.data, aes(size = size1,
colour=Barthel_Total), show.legend = F) +
  scale_size_continuous(breaks = 2:3, range = c(2,3)) +
  scale_colour_gradientn(colours = myPalette(100), limits=c(0,22),
na.value = "gray")
```

pb.ait.Barthel

##CAD
pb.CAD <- plot_ordination(qd.baseline.rare, axes=c(1,2), iMDS.all) +
  ggtitle(paste("CVD: p=0.017*")) +
  theme_bw() +
  theme(legend.title=element_blank(),
        legend.position=c(0.9,0.1)) +
  geom_point(size = 2, colour = "gray") +
  geom_point(size = 3, aes(colour=CAD)) +
  scale_colour_manual(na.translate = F,
                      values=c("No"   = "#9ebcda",
                             "Yes" = "#8856a7"), labels=c(
    paste("No (", length(which(sample_data(qd.baseline)$CAD ==
"No")), ")", sep=""),
    paste("Yes (", length(which(sample_data(qd.baseline)$CAD ==
"Yes")), ")", sep="")))
pb.CAD

pb.ait.CAD <- plot_ordination(qd.baseline.clr, axes=c(1,2),
iMDS.ait.all) +
  ggtitle(paste("CVD: p=0.083")) +
  theme_bw() +
  theme(legend.title=element_blank(),
        legend.position=c(0.9,0.1)) +
  geom_point(size = 2, colour = "gray") +
  geom_point(size = 3, aes(colour=CAD)) +
  scale_colour_manual(values=c("No"   = "#9ebcda",
                             "Yes" = "#8856a7"), labels=c(
    paste("No (", length(which(sample_data(qd.baseline)$CAD ==
"No")), ")", sep=""),
    paste("Yes (", length(which(sample_data(qd.baseline)$CAD ==
"Yes")), ")", sep="")))
pb.ait.CAD
```

B. Investigations: Sex

B0. Colour defintion
```{r}
sex.colours <- c("Female" = "#ca0020",
                 "Male"   = "#0571b0")
```
B1. Do any ASVs correlate with Sex?
```{r}
#ANCOMBC
ancom_da = ancombc(phyloseq = qd.baseline, formula = "Sex",
              p_adj_method = "holm", zero_cut = 0.90, lib_cut =
1000,
              group = "Sex", struc_zero = TRUE, neg_lb = FALSE, tol
= 1e-5,
              max_iter = 100, conserve = TRUE, alpha = 0.05, global
= TRUE)
res = ancom_da$res

```
reg = ancom_da$res_global

#Visualize log fold change
samp_frac = ancom_da$samp_frac
# Replace NA with 0
samp_frac[is.na(samp_frac)] = 0
# Add pesudo-count (1) to avoid taking the log of 0
log_obs_abn = log(abundances(qd.baseline) + 1)
# Adjust the log observed abundances
log_obs_abn_adj = t(t(log_obs_abn) - samp_frac)
head(log_obs_abn_adj)

df_fig1 = data.frame(res$beta * res$diff_abn, check.names = FALSE)
%>%
  rownames_to_column("taxon_id")
df_fig2 = data.frame(res$se * res$diff_abn, check.names = FALSE) %>%
  rownames_to_column("taxon_id")
colnames(df_fig2)[-1] = paste0(colnames(df_fig2)[-1], "SD")
df_fig = df_fig1 %>% left_join(df_fig2, by = "taxon_id") %>%
  transmute(taxon_id, SexMale, SexMaleSD) %>%
  filter(SexMale != 0) %>% arrange(desc(SexMale)) %>%
  mutate(group = ifelse(SexMale > 0, "g1", "g2"))
head(df_fig)
#0 diff_abn taxa
```
B2. Is there a correlation between Sex and cluster membership?
```{r}
nrow(qd.baseline.clust.sam)
#Count frequency of each cluster by Site
#chi-squared
tbl.ev <- table(qd.baseline.clust.sam$Sex,
qd.baseline.clust.sam$cluster)
tbl.ev
chi <- chisq.test(tbl.ev) #p=0.248732
chi
contrib <- 100*chi$residuals^2/chi$statistic
corrplot(contrib, is.cor=FALSE)
corrplot(chi$residuals, is.cor=FALSE)

#Plot
counts.ev <- qd.baseline.clust.sam %>% dplyr::select(Sex, cluster)
freq.ev <- counts.ev %>%
  group_by(Sex, cluster) %>%
  summarize(n = n()) %>%
  mutate(freq = n / sum(n) * 100)

sex.clust <- ggplot(freq.ev, aes(x=Sex, y=freq, fill=cluster)) +
  geom_bar(position="stack", stat="identity") +
  scale_fill_manual(values=cluster.colours.k10) +
  scale_y_continuous(expand=c(0,0)) +
  theme_bw() +
  xlab("Sex") + ylab("Proportion of clusters per sample type") +
  ggtitle("p=0.248732")
sex.clust
```

````
```
B3. Is there a correlation between Sex and alpha-diversity?
```{r}
#Calculate Shannon diversity per sample
alpha = estimate_richness(qd.baseline, measures=c("Shannon"))
#Add clustering data
alpha <- cbind(alpha, as.data.frame(sample_data(qd.baseline)))

#Significant?
leveneTest(Shannon ~ as.factor(Sex), data=alpha) #p=0.771

sex.alpha.box <- ggplot(data=alpha, aes(x=Sex, y=Shannon,
colour=Sex, group=Sex)) +
  geom_boxplot() + geom_point(position="jitter", alpha=0.2) +
  xlab("Sex") + ggtitle("p=0.771") +
  theme_bw() + theme(legend.position="none") +
  scale_colour_manual(values=sex.colours)
sex.alpha.box
```

C. Investigations: Site

C0. Colour definition
```{r}
site.colours <- c("" = "#e6194b", #site names removed
                             "" = "#ffe119",
                             "" = "#46f0f0",
                             "" = "#f032e6",
                             "" = "#d2f53c",
                             "" = "#fabebe",
                             "" = "#008080",
                             "" = "#e6beff",
                             "" = "#aa6e28",
                             "" = "#fffac8",
                             "" = "#800000")
```
C1. [ancombc] Do any ASVs correlate with LTC Site?
```{r}
#omit
qd.baseline.Site = subset_samples(qd.baseline, (Site != "" & Site !=
""))

#ANCOMBC
ancom_da = ancombc(phyloseq = qd.baseline.Site, formula = "Site",
              p_adj_method = "holm", zero_cut = 0.90, lib_cut =
1000,
              group = "Site", struc_zero = TRUE, neg_lb = FALSE, tol
= 1e-5,
              max_iter = 100, conserve = TRUE, alpha = 0.05, global
= TRUE)
res = ancom_da$res
reg = ancom_da$res_global

#Visualize log fold change
````

````
samp_frac = ancom_da$samp_frac
# Replace NA with 0
samp_frac[is.na(samp_frac)] = 0
# Add pesudo-count (1) to avoid taking the log of 0
log_obs_abn = log(abundances(qd.baseline) + 1)
# Adjust the log observed abundances
log_obs_abn_adj = t(t(log_obs_abn) - samp_frac)
head(log_obs_abn_adj)
reg.dif <- subset(reg, reg$diff_abn == "TRUE")

#Replace ASV strings with genus names
df_figX <- merge(reg.dif, tax_table(qd), by = "row.names", all.x =
TRUE)
df_figX$taxon_id <- df_figX$Genus
df_figX$taxon_id #fix any that are missing the genus name
df_figX$taxon_id[1] <- "Chloroplast"
df_figX$taxon_id[23] <- "Muribaculaceae"
df_figX$taxon_id[36] <- "Enterobacteriaceae"
df_figX$taxon_id[48] <- "Ruminococcaceae"
df_figX$taxon_id[60] <- "Alicyclobacillaceae"
df_figX$taxon_id[63] <- "Lachnospiraceae"
df_figX$taxon_id[65] <- "Lachnospiraceae"
df_figX$taxon_id[67] <- "Lachnospiraceae"
df_figX$taxon_id[68] <- "Lachnospiraceae"
df_figX$taxon_id[73] <- "Bacillaceae"
df_figX$taxon_id[81] <- "Burkholderiaceae"
df_figX$taxon_id[88] <- "Neisseriaceae"
df_figX$taxon_id[90] <- "Corynebacteriaceae"
df_figX$taxon_id[93] <- "Corynebacteriaceae"
df_figX$taxon_id[94] <- "Corynebacteriaceae"
df_figX$taxon_id[110] <- "Ruminococcaceae"
df_figX$taxon_id[135] <- "Lachnospiraceae"
df_figX$taxon_id[139] <- "Mitochondria"
df_figX$taxon_id
head(df_figX)
df_figX[,c(6:11)] <- NULL
df_figX$q_val <- format(df_figX$q_val, digits=3)
waterfall.site = ggplot(data = df_figX,
           aes(x = taxon_id, y = W)) + #, fill = group, color =
group
  geom_bar(stat = "identity", width = 0.7,
           position = position_dodge(width = 0.4)) +
  geom_text(aes(label=q_val), color="black", vjust=-0.2) +
  labs(x = NULL, y = "Test statistic (W)",
       title = "Waterfall Plot for the Sample Type Effect") +
  theme_bw() +
  theme(legend.position = "none",
        plot.title = element_text(hjust = 0.5),
        panel.grid.minor.y = element_blank(),
        axis.text.x = element_text(angle = 90, hjust = 1))
waterfall.site
```
C1. Box plots per ASV
```{r}
````

```
#Set up dataframe
test = cbind(as(reg.dif, "data.frame"),
             as(otu_table(qd.baseline.norm)[rownames(reg.dif), ],
"matrix"))
test = test[,14:ncol(test)]
test = rbind(as(test, "data.frame"), t(as(sample_data(qd.baseline)
[colnames(test), ], "matrix")))
test = t(test)
test = as.data.frame(test)
options(digits=9)
nrow(test)
test <- subset(test, !(test$Site == "" | test$Site == ""))
nrow(test)

#Let's plot the mean relative abundance of them?
mean.relabund <- rowMeans(otu_table(qd.baseline.norm))
mean.relabund <- as.data.frame(mean.relabund)
mean.relabund$ASV <- rownames(mean.relabund)
reg.dif$ASV <- rownames(reg.dif)
sigtab.relabund <- merge(reg.dif, mean.relabund, by.x="ASV",
by.y="ASV")
p.relabund <- ggplot(data=sigtab.relabund, aes(x=ASV,
y=mean.relabund)) +
  geom_point() +
  geom_hline(yintercept=0.001, color="gray", linetype="dashed") +
  scale_y_log10()
p.relabund
length(which(sigtab.relabund$mean.relabund>0.001)) #9 ASVs > 0.001
mean rel abund
sigtab.high <- subset(sigtab.relabund,
sigtab.relabund$mean.relabund>0.001)
rownames(sigtab.high) <- sigtab.high$ASV
tmp <- merge(sigtab.high, tax_table(qd), by = "row.names", all.x =
TRUE)
tmp$Genus

head(sigtab.high)
head(test)

rownames(df_figX) <- df_figX$Row.names

i<-1
rownames(sigtab.high)[i]
df_figX[rownames(sigtab.high)[i],]$taxon_id
test[[rownames(sigtab.high)[i]]] <-
as.numeric(as.character(as.factor(test[[rownames(sigtab.high)
[i]]])))
site.p1 <- ggplot(data=test, aes(x=Site,
y=.data[[rownames(sigtab.high)[i]]])) +
  geom_point(size=2, aes(colour=Site)) +
  geom_boxplot(aes(colour=Site), alpha=0.1) +
  xlab("Sample type") + ylab("log(Relative abundance)") +
  scale_y_log10() +
  scale_x_discrete(labels=c("A", "B", "C", "D", "E", "F", "G", "H",
```

```
"I", "J")) +
  theme_bw() +
  ggtitle(paste(df_figX[rownames(sigtab.high)[i],]$taxon_id)) +
  theme(legend.position = "none") +
  scale_colour_manual(values=site.colours)

i<-2
rownames(sigtab.high)[i]
df_figX[rownames(sigtab.high)[i],]$taxon_id
test[[rownames(sigtab.high)[i]]] <-
as.numeric(as.character(as.factor(test[[rownames(sigtab.high)
[i]]])))
site.p2 <- ggplot(data=test, aes(x=Site,
y=.data[[rownames(sigtab.high)[i]]])) +
  geom_point(size=2, aes(colour=Site)) +
  geom_boxplot(aes(colour=Site), alpha=0.1) +
  xlab("Sample type") + ylab("log(Relative abundance)") +
  scale_y_log10() +
  scale_x_discrete(labels=c("A", "B", "C", "D", "E", "F", "G", "H",
"I", "J")) +
  theme_bw() +
  ggtitle(paste(df_figX[rownames(sigtab.high)[i],]$taxon_id)) +
  theme(legend.position = "none") +
  scale_colour_manual(values=site.colours) #+

i<-3
rownames(sigtab.high)[i]
df_figX[rownames(sigtab.high)[i],]$taxon_id
test[[rownames(sigtab.high)[i]]] <-
as.numeric(as.character(as.factor(test[[rownames(sigtab.high)
[i]]])))
site.p3 <- ggplot(data=test, aes(x=Site,
y=.data[[rownames(sigtab.high)[i]]])) +
  geom_point(size=2, aes(colour=Site)) +
  geom_boxplot(aes(colour=Site), alpha=0.1) +
  xlab("Sample type") + ylab("log(Relative abundance)") +
  scale_y_log10() +
  scale_x_discrete(labels=c("A", "B", "C", "D", "E", "F", "G", "H",
"I", "J")) +
  theme_bw() +
  ggtitle(paste(df_figX[rownames(sigtab.high)[i],]$taxon_id)) +
  theme(legend.position = "none") +
  scale_colour_manual(values=site.colours)

i<-4
rownames(sigtab.high)[i]
df_figX[rownames(sigtab.high)[i],]$taxon_id
test[[rownames(sigtab.high)[i]]] <-
as.numeric(as.character(as.factor(test[[rownames(sigtab.high)
[i]]])))
site.p4 <- ggplot(data=test, aes(x=Site,
y=.data[[rownames(sigtab.high)[i]]])) +
  geom_point(size=2, aes(colour=Site)) +
  geom_boxplot(aes(colour=Site), alpha=0.1) +
```

```
  xlab("Sample type") + ylab("log(Relative abundance)") +
  scale_y_log10() +
  scale_x_discrete(labels=c("A", "B", "C", "D", "E", "F", "G", "H",
"I", "J")) +
  theme_bw() +
  ggtitle(paste(df_figX[rownames(sigtab.high)[i],]$taxon_id)) +
  theme(legend.position = "none") +
  scale_colour_manual(values=site.colours)

i<-5
rownames(sigtab.high)[i]
df_figX[rownames(sigtab.high)[i],]$taxon_id
test[[rownames(sigtab.high)[i]]] <-
as.numeric(as.character(as.factor(test[[rownames(sigtab.high)
[i]]])))
site.p5 <- ggplot(data=test, aes(x=Site,
y=.data[[rownames(sigtab.high)[i]]])) +
  geom_point(size=2, aes(colour=Site)) +
  geom_boxplot(aes(colour=Site), alpha=0.1) +
  xlab("Sample type") + ylab("log(Relative abundance)") +
  scale_y_log10() +
  scale_x_discrete(labels=c("A", "B", "C", "D", "E", "F", "G", "H",
"I", "J")) +
  theme_bw() +
  ggtitle(paste(df_figX[rownames(sigtab.high)[i],]$taxon_id)) +
  theme(legend.position = "none") +
  scale_colour_manual(values=site.colours)

i<-6
rownames(sigtab.high)[i]
df_figX[rownames(sigtab.high)[i],]$taxon_id
test[[rownames(sigtab.high)[i]]] <-
as.numeric(as.character(as.factor(test[[rownames(sigtab.high)
[i]]])))
site.p6 <- ggplot(data=test, aes(x=Site,
y=.data[[rownames(sigtab.high)[i]]])) +
  geom_point(size=2, aes(colour=Site)) +
  geom_boxplot(aes(colour=Site), alpha=0.1) +
  xlab("Sample type") + ylab("log(Relative abundance)") +
  scale_y_log10() +
  scale_x_discrete(labels=c("A", "B", "C", "D", "E", "F", "G", "H",
"I", "J")) +
  theme_bw() +
  ggtitle(paste(df_figX[rownames(sigtab.high)[i],]$taxon_id)) +
  theme(legend.position = "none") +
  scale_colour_manual(values=site.colours)

i<-7
rownames(sigtab.high)[i]
df_figX[rownames(sigtab.high)[i],]$taxon_id
test[[rownames(sigtab.high)[i]]] <-
as.numeric(as.character(as.factor(test[[rownames(sigtab.high)
[i]]])))
site.p7 <- ggplot(data=test, aes(x=Site,
```

~~~~
y=.data[[rownames(sigtab.high)[i]]])) +
  geom_point(size=2, aes(colour=Site)) +
  geom_boxplot(aes(colour=Site), alpha=0.1) +
  xlab("Sample type") + ylab("log(Relative abundance)") +
  scale_y_log10() +
  scale_x_discrete(labels=c("A", "B", "C", "D", "E", "F", "G", "H",
"I", "J")) +
  theme_bw() +
  ggtitle(paste(df_figX[rownames(sigtab.high)[i],]$taxon_id)) +
  theme(legend.position = "none") +
  scale_colour_manual(values=site.colours)

i<-8
rownames(sigtab.high)[i]
df_figX[rownames(sigtab.high)[i],]$taxon_id
test[[rownames(sigtab.high)[i]]] <-
as.numeric(as.character(as.factor(test[[rownames(sigtab.high)
[i]]])))
site.p8 <- ggplot(data=test, aes(x=Site,
y=.data[[rownames(sigtab.high)[i]]])) +
  geom_point(size=2, aes(colour=Site)) +
  geom_boxplot(aes(colour=Site), alpha=0.1) +
  xlab("Sample type") + ylab("log(Relative abundance)") +
  scale_y_log10() +
  scale_x_discrete(labels=c("A", "B", "C", "D", "E", "F", "G", "H",
"I", "J")) +
  theme_bw() +
  ggtitle(paste(df_figX[rownames(sigtab.high)[i],]$taxon_id)) +
  theme(legend.position = "none") +
  scale_colour_manual(values=site.colours)

i<-9
rownames(sigtab.high)[i]
df_figX[rownames(sigtab.high)[i],]$taxon_id
test[[rownames(sigtab.high)[i]]] <-
as.numeric(as.character(as.factor(test[[rownames(sigtab.high)
[i]]])))
site.p9 <- ggplot(data=test, aes(x=Site,
y=.data[[rownames(sigtab.high)[i]]])) +
  geom_point(size=2, aes(colour=Site)) +
  geom_boxplot(aes(colour=Site), alpha=0.1) +
  xlab("Sample type") + ylab("log(Relative abundance)") +
  scale_y_log10() +
  scale_x_discrete(labels=c("A", "B", "C", "D", "E", "F", "G", "H",
"I", "J")) +
  theme_bw() +
  ggtitle(paste(df_figX[rownames(sigtab.high)[i],]$taxon_id)) +
  theme(legend.position = "none") +
  scale_colour_manual(values=site.colours) #+
```
C2. Is there a correlation between LTC Site and cluster membership?
```{r}
nrow(qd.baseline.clust.sam)
qd.baseline.clust.sam.Site <- subset(qd.baseline.clust.sam, !
~~~~

```
(qd.baseline.clust.sam$Site == "" | qd.baseline.clust.sam$Site ==
""))
nrow(qd.baseline.clust.sam.Site)
#Count frequency of each cluster by Site
#chi-squared
tbl.ev <- table(qd.baseline.clust.sam.Site$Site,
qd.baseline.clust.sam.Site$cluster)
tbl.ev
chi <- chisq.test(tbl.ev) #p=
chi
contrib <- 100*chi$residuals^2/chi$statistic
corrplot(contrib, is.cor=FALSE)
corrplot(chi$residuals, is.cor=FALSE)

#Plot
counts.ev <- qd.baseline.clust.sam.Site %>% dplyr::select(Site,
cluster)
freq.ev <- counts.ev %>%
  group_by(Site, cluster) %>%
  summarize(n = n()) %>%
  mutate(freq = n / sum(n) * 100)

site.clust <- ggplot(freq.ev, aes(x=Site, y=freq, fill=cluster)) +
  geom_bar(position="stack", stat="identity") +
  #geom_text(aes(label=freq)) +
  scale_fill_manual(values=cluster.colours.k10) +
  scale_x_discrete(labels =
c("A","B","C","D","E","F","G","H","I","J")) +
  scale_y_continuous(expand=c(0,0)) +
  theme_bw() +
  xlab("LTC Site") + ylab("Proportion of clusters per sample type")
+
  ggtitle("p=0.616413")
site.clust
```
C3. Is there a correlation between LTC Site and alpha-diversity?
```{r}
#Calculate Shannon diversity per sample
alpha = estimate_richness(qd.baseline.Site, measures=c("Shannon"))
alpha$SampleID <- rownames(alpha)
#Add clustering data
alpha <- cbind(alpha, as.data.frame(sample_data(qd.baseline.Site)))
#Remove 2 LTC Sites
alpha <- subset(alpha, (alpha$SampleID != "ML130" & alpha$SampleID!
="ML131" & alpha$SampleID!= "ML173" & alpha$SampleID!="ML174" &
alpha$SampleID!="ML175" & alpha$SampleID!="ML176" & alpha$SampleID!
="ML455" & alpha$SampleID!="ML457"))
nrow(alpha)

#Significant?
leveneTest(Shannon ~ as.factor(Site), data=alpha) #p=

plot.alpha.box <- ggplot(data=alpha, aes(x=Site, y=Shannon,
colour=Site, group=Site)) +
```

```
  geom_boxplot() + geom_point(position="jitter", alpha=0.2) +
  xlab("LTC Site") + ggtitle("p=0.80649") +
  theme_bw() + theme(legend.position="none") +
  scale_colour_manual(values=site.colours) +

scale_x_discrete(labels=c("A","B","C","D","E","F","G","H","I","J"))
plot.alpha.box
```

D. Investigations: Month, Season, Year

D0. Colour definition

```{r}
month.colours <- c("Oct" = "#fdae61",
                   "Nov" = "#1a9641",
                   "Dec" = "red",
                   "Jan" = "blue")
season.colours <- c("Autumn" = "#fdae61",
                    "Winter" = "#1a9641")
year.colours <- c("2014" = "#4daf4a",
                  "2015" = "#984ea3",
                  "2016" = "#ff7f00")
```

D1. Do any ASVs correlate with time?
Month

```{r}
qd.baseline.Month <- subset_samples(qd.baseline, Month != "Feb")
#ANCOMBC
ancom_da = ancombc(phyloseq = qd.baseline.Month, formula = "Month",
              p_adj_method = "holm", zero_cut = 0.90, lib_cut =
1000,
              group = "Month", struc_zero = TRUE, neg_lb = FALSE,
tol = 1e-5,
              max_iter = 100, conserve = TRUE, alpha = 0.05, global
= TRUE)
res = ancom_da$res
reg = ancom_da$res_global

#Visualize log fold change
samp_frac = ancom_da$samp_frac
# Replace NA with 0
samp_frac[is.na(samp_frac)] = 0
# Add pesudo-count (1) to avoid taking the log of 0
log_obs_abn = log(abundances(qd.baseline) + 1)
# Adjust the log observed abundances
log_obs_abn_adj = t(t(log_obs_abn) - samp_frac)
head(log_obs_abn_adj)
reg.dif <- subset(reg, reg$diff_abn == "TRUE")
nrow(reg.dif)
#28 DA ASVs

#Replace ASV strings with genus names
df_figX <- merge(reg.dif, tax_table(qd), by = "row.names", all.x =
TRUE)
```

````
df_figX$taxon_id <- df_figX$Genus
df_figX$taxon_id #fix any that are missing the genus name
df_figX$taxon_id[6] <- "Muribaculaceae"
df_figX$taxon_id[19] <- "Corynebacteriaceae"
df_figX$taxon_id[20] <- "Corynebacteriaceae"
df_figX$taxon_id
head(df_figX)
df_figX[,c(5:10)] <- NULL
df_figX$q_val <- format(df_figX$q_val, digits=3)
waterfall.month = ggplot(data = df_figX,
           aes(x = taxon_id, y = W)) + #, fill = group, color =
group
  geom_bar(stat = "identity", width = 0.7,
           position = position_dodge(width = 0.4)) +
  geom_text(aes(label=q_val), color="black", vjust=-0.2) +
  labs(x = NULL, y = "Test statistic (W)",
       title = "Waterfall Plot for the Sample Type Effect") +
  theme_bw() +
  theme(legend.position = "none",
        plot.title = element_text(hjust = 0.5),
        panel.grid.minor.y = element_blank(),
        axis.text.x = element_text(angle = 90, hjust = 1))
waterfall.month
rownames(df_figX) <- df_figX$Row.names
```
Season
```{r}
#ANCOMBC
ancom_da = ancombc(phyloseq = qd.baseline, formula = "Season",
              p_adj_method = "holm", zero_cut = 0.90, lib_cut =
1000,
              group = "Season", struc_zero = TRUE, neg_lb = FALSE,
tol = 1e-5,
              max_iter = 100, conserve = TRUE, alpha = 0.05, global
= TRUE)
res = ancom_da$res

#Visualize log fold change
samp_frac = ancom_da$samp_frac
# Replace NA with 0
samp_frac[is.na(samp_frac)] = 0
# Add pesudo-count (1) to avoid taking the log of 0
log_obs_abn = log(abundances(qd.baseline) + 1)
# Adjust the log observed abundances
log_obs_abn_adj = t(t(log_obs_abn) - samp_frac)
head(log_obs_abn_adj)

df_fig1 = data.frame(res$beta * res$diff_abn, check.names = FALSE)
%>%
  rownames_to_column("taxon_id")
df_fig2 = data.frame(res$se * res$diff_abn, check.names = FALSE) %>%
  rownames_to_column("taxon_id")
colnames(df_fig2)[-1] = paste0(colnames(df_fig2)[-1], "SD")
df_fig = df_fig1 %>% left_join(df_fig2, by = "taxon_id") %>%
````

```
  transmute(taxon_id, SeasonWinter, SeasonWinterSD) %>%
  filter(SeasonWinter != 0) %>% arrange(desc(SeasonWinter)) %>%
  mutate(group = ifelse(SeasonWinter > 0, "g1", "g2"))
head(df_fig)
nrow(df_fig)
#4 DA ASVs
df_fig$taxon_id = factor(df_fig$taxon_id, levels = df_fig$taxon_id)
#Grab adjusted p-values to display
rownames(df_fig) <- as.character(df_fig$taxon_id)
df_qvals <- as.data.frame(res$q_val)
colnames(df_qvals)[1] = paste0(colnames(df_qvals)[1],"ADJPVAL")
df_figY <- merge(df_fig, df_qvals, by = "row.names", all.x = TRUE)
rownames(df_figY) <- df_figY$Row.names
df_figY$Row.names <- NULL
#Replace ASV strings with genus names
df_figX <- merge(df_figY, tax_table(qd), by = "row.names", all.x =
TRUE)
df_figX$taxon_id <- df_figX$Genus
df_figX$taxon_id #fix any that are missing the genus name
colnames(df_figX)
df_figX[,c(7:12)] <- NULL
df_figX$q_val <- format(df_figX$q_val, digits=3)
waterfall.season = ggplot(data = df_figX,
           aes(x = taxon_id, y = SeasonWinter, fill = group, color =
group)) +
  geom_bar(stat = "identity", width = 0.7,
           position = position_dodge(width = 0.4)) +
  geom_errorbar(aes(ymin = SeasonWinter - SeasonWinterSD, ymax =
SeasonWinter + SeasonWinterSD), width = 0.2,
                position = position_dodge(0.05), color = "black") +
  geom_text(aes(label=SeasonWinterADJPVAL), color="black",
vjust=-0.2) +
  labs(x = NULL, y = "Test statistic (W)",
       title = "Waterfall Plot for the Sample Type Effect") +
  theme_bw() +
  theme(legend.position = "none",
        plot.title = element_text(hjust = 0.5),
        panel.grid.minor.y = element_blank(),
        axis.text.x = element_text(angle = 90, hjust = 1))
waterfall.season
rownames(df_figX) <- df_figX$Row.names
```
Year
```{r}
qd.baseline.Year <- subset_samples(qd.baseline, Year != "2017")
#ANCOMBC
ancom_da = ancombc(phyloseq = qd.baseline.Year, formula = "Year",
              p_adj_method = "holm", zero_cut = 0.90, lib_cut =
1000,
              group = "Year", struc_zero = TRUE, neg_lb = FALSE, tol
= 1e-5,
              max_iter = 100, conserve = TRUE, alpha = 0.05, global
= TRUE)
res = ancom_da$res
```

```
reg = ancom_da$res_global

#Visualize log fold change
samp_frac = ancom_da$samp_frac
# Replace NA with 0
samp_frac[is.na(samp_frac)] = 0
# Add pesudo-count (1) to avoid taking the log of 0
log_obs_abn = log(abundances(qd.baseline) + 1)
# Adjust the log observed abundances
log_obs_abn_adj = t(t(log_obs_abn) - samp_frac)
head(log_obs_abn_adj)
reg.dif <- subset(reg, reg$diff_abn == "TRUE")
nrow(reg.dif)
#25 DA ASVs

#Replace ASV strings with genus names
df_figX <- merge(reg.dif, tax_table(qd), by = "row.names", all.x =
TRUE)
df_figX$taxon_id <- df_figX$Genus
df_figX$taxon_id #fix any that are missing the genus name
df_figX$taxon_id[6] <- "Xanthobacteraceae"
df_figX$taxon_id[12] <- "Alicyclobacillaceae"
df_figX$taxon_id[15] <- "Burkholderiaceae"
df_figX$taxon_id[22] <- "Ruminococcaceae"
df_figX$taxon_id[25] <- "Bacteria"
df_figX$taxon_id
head(df_figX)
df_figX$q_val <- format(df_figX$q_val, digits=3)
waterfall.year = ggplot(data = df_figX,
           aes(x = taxon_id, y = W)) + #, fill = group, color =
group
  geom_bar(stat = "identity", width = 0.7,
           position = position_dodge(width = 0.4)) +
  geom_text(aes(label=q_val), color="black", vjust=-0.2) +
  labs(x = NULL, y = "Test statistic (W)",
       title = "Waterfall Plot for the Sample Type Effect") +
  theme_bw() +
  theme(legend.position = "none",
        plot.title = element_text(hjust = 0.5),
        panel.grid.minor.y = element_blank(),
        axis.text.x = element_text(angle = 90, hjust = 1))
waterfall.year
rownames(df_figX) <- df_figX$Row.names
```

D1. Box plots per ASV

Month

```{r}
qd.baseline.Month.norm <- transform_sample_counts(qd.baseline.Month,
normf)
#Set up dataframe
test = cbind(as(reg.dif, "data.frame"),
             as(otu_table(qd.baseline.Month.norm)
[rownames(reg.dif), ], "matrix"))
colnames(test)
```

```
test = test[,6:ncol(test)]
test = rbind(as(test, "data.frame"),
t(as(sample_data(qd.baseline.Month)[colnames(test), ], "matrix")))
test = t(test)
test = as.data.frame(test)
options(digits=9)
nrow(test)

#Let's plot the mean relative abundance of them?
mean.relabund <- rowMeans(otu_table(qd.baseline.Month.norm))
mean.relabund <- as.data.frame(mean.relabund)
mean.relabund$ASV <- rownames(mean.relabund)
reg.dif$ASV <- rownames(reg.dif)
sigtab.relabund <- merge(reg.dif, mean.relabund, by.x="ASV",
by.y="ASV")
p.relabund <- ggplot(data=sigtab.relabund, aes(x=ASV,
y=mean.relabund)) +
  geom_point() +
  geom_hline(yintercept=0.001, color="gray", linetype="dashed") +
  scale_y_log10()
p.relabund
length(which(sigtab.relabund$mean.relabund>0.001)) #5 ASVs > 0.001
mean rel abund
sigtab.high <- subset(sigtab.relabund,
sigtab.relabund$mean.relabund>0.001)
rownames(sigtab.high) <- sigtab.high$ASV
tmp <- merge(sigtab.high, tax_table(qd), by = "row.names", all.x =
TRUE)
tmp$Genus

test$Month <- factor(test$Month, levels=c("Oct","Nov","Dec","Jan"))
i<-1
rownames(sigtab.high)[i]
df_figX[rownames(sigtab.high)[i],]$taxon_id
test[[rownames(sigtab.high)[i]]] <-
as.numeric(as.character(as.factor(test[[rownames(sigtab.high)
[i]]])))
month.p1 <- ggplot(data=test, aes(x=Month,
y=.data[[rownames(sigtab.high)[i]]])) +
  geom_point(size=2, aes(colour=Month)) +
  geom_boxplot(aes(colour=Month), alpha=0.1) +
  xlab("Month") + ylab("log(Relative abundance)") +
  scale_y_log10() +
  theme_bw() +
  ggtitle(paste(df_figX[rownames(sigtab.high)[i],]$taxon_id)) +
  theme(legend.position = "none") +
  scale_colour_manual(values=month.colours) #+

i<-2
rownames(sigtab.high)[i]
df_figX[rownames(sigtab.high)[i],]$taxon_id
test[[rownames(sigtab.high)[i]]] <-
as.numeric(as.character(as.factor(test[[rownames(sigtab.high)
[i]]])))
```

```
month.p2 <- ggplot(data=test, aes(x=Month,
y=.data[[rownames(sigtab.high)[i]]])) +
  geom_point(size=2, aes(colour=Month)) +
  geom_boxplot(aes(colour=Month), alpha=0.1) +
  xlab("Month") + ylab("log(Relative abundance)") +
  scale_y_log10() +
  theme_bw() +
  ggtitle(paste(df_figX[rownames(sigtab.high)[i],]$taxon_id)) +
  theme(legend.position = "none") +
  scale_colour_manual(values=month.colours)

i<-3
rownames(sigtab.high)[i]
df_figX[rownames(sigtab.high)[i],]$taxon_id
test[[rownames(sigtab.high)[i]]] <-
as.numeric(as.character(as.factor(test[[rownames(sigtab.high)
[i]]])))
month.p3 <- ggplot(data=test, aes(x=Month,
y=.data[[rownames(sigtab.high)[i]]])) +
  geom_point(size=2, aes(colour=Month)) +
  geom_boxplot(aes(colour=Month), alpha=0.1) +
  xlab("Month") + ylab("log(Relative abundance)") +
  scale_y_log10() +
  theme_bw() +
  ggtitle(paste(df_figX[rownames(sigtab.high)[i],]$taxon_id)) +
  theme(legend.position = "none") +
  scale_colour_manual(values=month.colours) #+

i<-4
rownames(sigtab.high)[i]
df_figX[rownames(sigtab.high)[i],]$taxon_id
test[[rownames(sigtab.high)[i]]] <-
as.numeric(as.character(as.factor(test[[rownames(sigtab.high)
[i]]])))
month.p4 <- ggplot(data=test, aes(x=Month,
y=.data[[rownames(sigtab.high)[i]]])) +
  geom_point(size=2, aes(colour=Month)) +
  geom_boxplot(aes(colour=Month), alpha=0.1) +
  xlab("Month") + ylab("log(Relative abundance)") +
  scale_y_log10() +
  theme_bw() +
  ggtitle(paste(df_figX[rownames(sigtab.high)[i],]$taxon_id)) +
  theme(legend.position = "none") +
  scale_colour_manual(values=month.colours)

i<-5
rownames(sigtab.high)[i]
df_figX[rownames(sigtab.high)[i],]$taxon_id
test[[rownames(sigtab.high)[i]]] <-
as.numeric(as.character(as.factor(test[[rownames(sigtab.high)
[i]]])))
month.p5 <- ggplot(data=test, aes(x=Month,
y=.data[[rownames(sigtab.high)[i]]])) +
  geom_point(size=2, aes(colour=Month)) +
```

````
  geom_boxplot(aes(colour=Month), alpha=0.1) +
  xlab("Month") + ylab("log(Relative abundance)") +
  scale_y_log10() +
  theme_bw() +
  ggtitle(paste(df_figX[rownames(sigtab.high)[i],]$taxon_id)) +
  theme(legend.position = "none") +
  scale_colour_manual(values=month.colours)
```
Season
```{r}
#Set up dataframe
test = cbind(as(df_figX, "data.frame"),
             as(otu_table(qd.baseline.norm)[rownames(df_figX), ],
"matrix"))
colnames(test)
test = test[,8:ncol(test)]
test = rbind(as(test, "data.frame"), t(as(sample_data(qd.baseline)
[colnames(test), ], "matrix")))
test = t(test)
test = as.data.frame(test)
options(digits=9)
nrow(test)

#Let's plot the mean relative abundance of them?
mean.relabund <- rowMeans(otu_table(qd.baseline.norm))
mean.relabund <- as.data.frame(mean.relabund)
mean.relabund$ASV <- rownames(mean.relabund)
df_figX$ASV <- rownames(df_figX)
sigtab.relabund <- merge(df_figX, mean.relabund, by.x="ASV",
by.y="ASV")
p.relabund <- ggplot(data=sigtab.relabund, aes(x=ASV,
y=mean.relabund)) +
  geom_point() +
  geom_hline(yintercept=0.001, color="gray", linetype="dashed") +
  scale_y_log10()
p.relabund
length(which(sigtab.relabund$mean.relabund>0.001)) #1 ASVs > 0.001
mean rel abund
sigtab.high <- subset(sigtab.relabund,
sigtab.relabund$mean.relabund>0.001)
rownames(sigtab.high) <- sigtab.high$ASV
tmp <- merge(sigtab.high, tax_table(qd), by = "row.names", all.x =
TRUE)
tmp$Genus

i<-1
rownames(sigtab.high)[i]
df_figX[rownames(sigtab.high)[i],]$taxon_id
test[[rownames(sigtab.high)[i]]] <-
as.numeric(as.character(as.factor(test[[rownames(sigtab.high)
[i]]])))
season.p1 <- ggplot(data=test, aes(x=Season,
y=.data[[rownames(sigtab.high)[i]]])) +
  geom_point(size=2, aes(colour=Season)) +
````

```
  geom_boxplot(aes(colour=Season), alpha=0.1) +
  xlab("Season") + ylab("log(Relative abundance)") +
  scale_y_log10() +
  theme_bw() +
  ggtitle(paste(df_figX[rownames(sigtab.high)[i],]$taxon_id)) +
  theme(legend.position = "none") +
  scale_colour_manual(values=season.colours)
```

Year

```{r}
qd.baseline.Year.norm <- transform_sample_counts(qd.baseline.Year,
normf)
#Set up dataframe
test = cbind(as(reg.dif, "data.frame"),
             as(otu_table(qd.baseline.Year.norm)
[rownames(reg.dif), ], "matrix"))
colnames(test)
test = test[,6:ncol(test)]
test = rbind(as(test, "data.frame"),
t(as(sample_data(qd.baseline.Year)[colnames(test), ], "matrix")))
test = t(test)
test = as.data.frame(test)
options(digits=9)
nrow(test)

#Let's plot the mean relative abundance of them?
mean.relabund <- rowMeans(otu_table(qd.baseline.Year.norm))
mean.relabund <- as.data.frame(mean.relabund)
mean.relabund$ASV <- rownames(mean.relabund)
reg.dif$ASV <- rownames(reg.dif)
sigtab.relabund <- merge(reg.dif, mean.relabund, by.x="ASV",
by.y="ASV")
p.relabund <- ggplot(data=sigtab.relabund, aes(x=ASV,
y=mean.relabund)) +
  geom_point() +
  geom_hline(yintercept=0.001, color="gray", linetype="dashed") +
  scale_y_log10()
p.relabund
length(which(sigtab.relabund$mean.relabund>0.001)) #9 ASVs > 0.001
mean rel abund
sigtab.high <- subset(sigtab.relabund,
sigtab.relabund$mean.relabund>0.001)
rownames(sigtab.high) <- sigtab.high$ASV
tmp <- merge(sigtab.high, tax_table(qd), by = "row.names", all.x =
TRUE)
tmp$Genus

i<-1
rownames(sigtab.high)[i]
df_figX[rownames(sigtab.high)[i],]$taxon_id
test[[rownames(sigtab.high)[i]]] <-
as.numeric(as.character(as.factor(test[[rownames(sigtab.high)
[i]]])))
year.p1 <- ggplot(data=test, aes(x=Year,
```

```
y=.data[[rownames(sigtab.high)[i]]])) +
  geom_point(size=2, aes(colour=Year)) +
  geom_boxplot(aes(colour=Year), alpha=0.1) +
  xlab("Year") + ylab("log(Relative abundance)") +
  scale_y_log10() +
  theme_bw() +
  ggtitle(paste(df_figX[rownames(sigtab.high)[i],]$taxon_id)) +
  theme(legend.position = "none") +
  scale_colour_manual(values=year.colours)

i<-2
rownames(sigtab.high)[i]
df_figX[rownames(sigtab.high)[i],]$taxon_id
test[[rownames(sigtab.high)[i]]] <-
as.numeric(as.character(as.factor(test[[rownames(sigtab.high)
[i]]])))
year.p2 <- ggplot(data=test, aes(x=Year,
y=.data[[rownames(sigtab.high)[i]]])) +
  geom_point(size=2, aes(colour=Year)) +
  geom_boxplot(aes(colour=Year), alpha=0.1) +
  xlab("Year") + ylab("log(Relative abundance)") +
  scale_y_log10() +
  theme_bw() +
  ggtitle(paste(df_figX[rownames(sigtab.high)[i],]$taxon_id)) +
  theme(legend.position = "none") +
  scale_colour_manual(values=year.colours)

i<-3
rownames(sigtab.high)[i]
df_figX[rownames(sigtab.high)[i],]$taxon_id
test[[rownames(sigtab.high)[i]]] <-
as.numeric(as.character(as.factor(test[[rownames(sigtab.high)
[i]]])))
year.p3 <- ggplot(data=test, aes(x=Year,
y=.data[[rownames(sigtab.high)[i]]])) +
  geom_point(size=2, aes(colour=Year)) +
  geom_boxplot(aes(colour=Year), alpha=0.1) +
  xlab("Year") + ylab("log(Relative abundance)") +
  scale_y_log10() +
  theme_bw() +
  ggtitle(paste(df_figX[rownames(sigtab.high)[i],]$taxon_id)) +
  theme(legend.position = "none") +
  scale_colour_manual(values=year.colours)

i<-4
rownames(sigtab.high)[i]
df_figX[rownames(sigtab.high)[i],]$taxon_id
test[[rownames(sigtab.high)[i]]] <-
as.numeric(as.character(as.factor(test[[rownames(sigtab.high)
[i]]])))
year.p4 <- ggplot(data=test, aes(x=Year,
y=.data[[rownames(sigtab.high)[i]]])) +
  geom_point(size=2, aes(colour=Year)) +
  geom_boxplot(aes(colour=Year), alpha=0.1) +
```

```
  xlab("Year") + ylab("log(Relative abundance)") +
  scale_y_log10() +
  theme_bw() +
  ggtitle(paste(df_figX[rownames(sigtab.high)[i],]$taxon_id)) +
  theme(legend.position = "none") +
  scale_colour_manual(values=year.colours)

i<-5
rownames(sigtab.high)[i]
df_figX[rownames(sigtab.high)[i],]$taxon_id
test[[rownames(sigtab.high)[i]]] <-
as.numeric(as.character(as.factor(test[[rownames(sigtab.high)
[i]]])))
year.p5 <- ggplot(data=test, aes(x=Year,
y=.data[[rownames(sigtab.high)[i]]])) +
  geom_point(size=2, aes(colour=Year)) +
  geom_boxplot(aes(colour=Year), alpha=0.1) +
  xlab("Year") + ylab("log(Relative abundance)") +
  scale_y_log10() +
  theme_bw() +
  ggtitle(paste(df_figX[rownames(sigtab.high)[i],]$taxon_id)) +
  theme(legend.position = "none") +
  scale_colour_manual(values=year.colours)

i<-6
rownames(sigtab.high)[i]
df_figX[rownames(sigtab.high)[i],]$taxon_id
test[[rownames(sigtab.high)[i]]] <-
as.numeric(as.character(as.factor(test[[rownames(sigtab.high)
[i]]])))
year.p6 <- ggplot(data=test, aes(x=Year,
y=.data[[rownames(sigtab.high)[i]]])) +
  geom_point(size=2, aes(colour=Year)) +
  geom_boxplot(aes(colour=Year), alpha=0.1) +
  xlab("Year") + ylab("log(Relative abundance)") +
  scale_y_log10() +
  theme_bw() +
  ggtitle(paste(df_figX[rownames(sigtab.high)[i],]$taxon_id)) +
  theme(legend.position = "none") +
  scale_colour_manual(values=year.colours)

i<-7
rownames(sigtab.high)[i]
df_figX[rownames(sigtab.high)[i],]$taxon_id
test[[rownames(sigtab.high)[i]]] <-
as.numeric(as.character(as.factor(test[[rownames(sigtab.high)
[i]]])))
year.p7 <- ggplot(data=test, aes(x=Year,
y=.data[[rownames(sigtab.high)[i]]])) +
  geom_point(size=2, aes(colour=Year)) +
  geom_boxplot(aes(colour=Year), alpha=0.1) +
  xlab("Year") + ylab("log(Relative abundance)") +
  scale_y_log10() +
  theme_bw() +
```

```
  ggtitle(paste(df_figX[rownames(sigtab.high)[i],]$taxon_id)) +
  theme(legend.position = "none") +
  scale_colour_manual(values=year.colours)

i<-8
rownames(sigtab.high)[i]
df_figX[rownames(sigtab.high)[i],]$taxon_id
test[[rownames(sigtab.high)[i]]] <-
as.numeric(as.character(as.factor(test[[rownames(sigtab.high)
[i]]])))
year.p8 <- ggplot(data=test, aes(x=Year,
y=.data[[rownames(sigtab.high)[i]]])) +
  geom_point(size=2, aes(colour=Year)) +
  geom_boxplot(aes(colour=Year), alpha=0.1) +
  xlab("Year") + ylab("log(Relative abundance)") +
  scale_y_log10() +
  theme_bw() +
  ggtitle(paste(df_figX[rownames(sigtab.high)[i],]$taxon_id)) +
  theme(legend.position = "none") +
  scale_colour_manual(values=year.colours)

i<-9
rownames(sigtab.high)[i]
df_figX[rownames(sigtab.high)[i],]$taxon_id
test[[rownames(sigtab.high)[i]]] <-
as.numeric(as.character(as.factor(test[[rownames(sigtab.high)
[i]]])))
year.p9 <- ggplot(data=test, aes(x=Year,
y=.data[[rownames(sigtab.high)[i]]])) +
  geom_point(size=2, aes(colour=Year)) +
  geom_boxplot(aes(colour=Year), alpha=0.1) +
  xlab("Year") + ylab("log(Relative abundance)") +
  scale_y_log10() +
  theme_bw() +
  ggtitle(paste(df_figX[rownames(sigtab.high)[i],]$taxon_id)) +
  theme(legend.position = "none") +
  scale_colour_manual(values=year.colours)
```
D2. Is there a correlation between time and cluster membership?
```{r}
nrow(qd.baseline.clust.sam)
qd.baseline.clust.sam.Month <- subset(qd.baseline.clust.sam, Month !
= "Feb")
nrow(qd.baseline.clust.sam.Month)
#Count frequency of each cluster by Site
#chi-squared
tbl.ev <- table(qd.baseline.clust.sam.Month$Month,
qd.baseline.clust.sam.Month$cluster)
tbl.ev
chi <- chisq.test(tbl.ev) #p=
chi
contrib <- 100*chi$residuals^2/chi$statistic
corrplot(contrib, is.cor=FALSE)
corrplot(chi$residuals, is.cor=FALSE)
```

```
#Plot
counts.ev <- qd.baseline.clust.sam.Month %>% dplyr::select(Month,
cluster)
freq.ev <- counts.ev %>%
  group_by(Month, cluster) %>%
  summarize(n = n()) %>%
  mutate(freq = n / sum(n) * 100)

month.clust <- ggplot(freq.ev, aes(x=Month, y=freq, fill=cluster)) +
  geom_bar(position="stack", stat="identity") +
  scale_fill_manual(values=cluster.colours.k10) +
  scale_y_continuous(expand=c(0,0)) +
  theme_bw() +
  xlab("Month") + ylab("Proportion of clusters per Month") +
  ggtitle("p=0.745318")
month.clust

tbl.ev <- table(qd.baseline.clust.sam$Season,
qd.baseline.clust.sam$cluster)
tbl.ev
chi <- chisq.test(tbl.ev) #p=0.
chi
contrib <- 100*chi$residuals^2/chi$statistic
corrplot(contrib, is.cor=FALSE)
corrplot(chi$residuals, is.cor=FALSE)

#Plot
counts.ev <- qd.baseline.clust.sam %>% dplyr::select(Season,
cluster)
freq.ev <- counts.ev %>%
  group_by(Season, cluster) %>%
  summarize(n = n()) %>%
  mutate(freq = n / sum(n) * 100)

season.clust <- ggplot(freq.ev, aes(x=Season, y=freq, fill=cluster))
+
  geom_bar(position="stack", stat="identity") +
  scale_fill_manual(values=cluster.colours.k10) +
  scale_y_continuous(expand=c(0,0)) +
  theme_bw() +
  xlab("Season") + ylab("Proportion of clusters per Season") +
  ggtitle("p=0.734067")
season.clust

qd.baseline.clust.sam.Year <- subset(qd.baseline.clust.sam,
qd.baseline.clust.sam$Year != "2017")
tbl.ev <- table(qd.baseline.clust.sam.Year$Year,
qd.baseline.clust.sam.Year$cluster)
tbl.ev
chi <- chisq.test(tbl.ev) #p=0.146409
chi
contrib <- 100*chi$residuals^2/chi$statistic
corrplot(contrib, is.cor=FALSE)
```

```
corrplot(chi$residuals, is.cor=FALSE)

#Plot
counts.ev <- qd.baseline.clust.sam.Year %>% dplyr::select(Year,
cluster)
freq.ev <- counts.ev %>%
  group_by(Year, cluster) %>%
  summarize(n = n()) %>%
  mutate(freq = n / sum(n) * 100)

year.clust <- ggplot(freq.ev, aes(x=Year, y=freq, fill=cluster)) +
  geom_bar(position="stack", stat="identity") +
  scale_fill_manual(values=cluster.colours.k10) +
  scale_y_continuous(expand=c(0,0)) +
  theme_bw() +
  xlab("Year") + ylab("Proportion of clusters per Year") +
  ggtitle("p=0.146409")
year.clust
```
D3. Is there a correlation between time and alpha-diversity?
```{r}
#Calculate Shannon diversity per sample
alpha = estimate_richness(qd.baseline.Month, measures=c("Shannon"))
#Add clustering data
alpha <- cbind(alpha, as.data.frame(sample_data(qd.baseline.Month)))

#Significant?
leveneTest(Shannon ~ as.factor(Month), data=alpha) #p=0.85244

month.alpha.box <- ggplot(data=alpha, aes(x=Month, y=Shannon,
colour=Month, group=Month)) +
  geom_boxplot() + geom_point(position="jitter", alpha=0.2) +
  xlab("Month") + ggtitle("p=0.85244") +
  theme_bw() + theme(legend.position="none") +
  scale_colour_manual(values=month.colours)
month.alpha.box

#Calculate Shannon diversity per sample
alpha = estimate_richness(qd.baseline, measures=c("Shannon"))
#Add clustering data
alpha <- cbind(alpha, as.data.frame(sample_data(qd.baseline)))

#Significant?
leveneTest(Shannon ~ as.factor(Season), data=alpha) #p=0.60661

season.alpha.box <- ggplot(data=alpha, aes(x=Season, y=Shannon,
colour=Season, group=Season)) +
  geom_boxplot() + geom_point(position="jitter", alpha=0.2) +
  xlab("Season") + ggtitle("p=0.60661") +
  theme_bw() + theme(legend.position="none") +
  scale_colour_manual(values=season.colours)
season.alpha.box

#Calculate Shannon diversity per sample
```

````
alpha = estimate_richness(qd.baseline.Year, measures=c("Shannon"))
#Add clustering data
alpha <- cbind(alpha, as.data.frame(sample_data(qd.baseline.Year)))

#Significant?
leveneTest(Shannon ~ Year, data=alpha) #p=0.049917

year.alpha.box <- ggplot(data=alpha, aes(x=Year, y=Shannon,
colour=Year, group=Year)) +
  geom_boxplot() + geom_point(position="jitter", alpha=0.2) +
  xlab("Year") + ggtitle("p=0.049917*") +
  theme_bw() + theme(legend.position="none") +
  scale_colour_manual(values=year.colours)
year.alpha.box
```

E. Investigations: Barthel total

E0. Colour definition
```{r}
#scale_colour_gradientn(colours = myPalette(100), limits=c(0,22),
na.value = "gray")
```
E1. [ancombc] Do any ASVs correlate with Barthel?
```{r}
#ANCOMBC
#Continuous variable, therefore
#https://github.com/FrederickHuangLin/ANCOMBC/issues/6
ancom_da = ancombc(phyloseq = qd.baseline, formula =
"Barthel_Total",
              p_adj_method = "holm", zero_cut = 0.90, lib_cut =
1000,
              group = NULL, struc_zero = FALSE, neg_lb = FALSE, tol
= 1e-5,
              max_iter = 100, conserve = TRUE, alpha = 0.05, global
= FALSE)
res = ancom_da$res

#Visualize log fold change
samp_frac = ancom_da$samp_frac
# Replace NA with 0
samp_frac[is.na(samp_frac)] = 0
# Add pesudo-count (1) to avoid taking the log of 0
log_obs_abn = log(abundances(qd.baseline) + 1)
# Adjust the log observed abundances
log_obs_abn_adj = t(t(log_obs_abn) - samp_frac)
head(log_obs_abn_adj)
df_fig1 = data.frame(res$beta * res$diff_abn, check.names = FALSE)
%>%
  rownames_to_column("taxon_id")
df_fig2 = data.frame(res$se * res$diff_abn, check.names = FALSE) %>%
  rownames_to_column("taxon_id")
colnames(df_fig2)[-1] = paste0(colnames(df_fig2)[-1], "SD")
df_fig = df_fig1 %>% left_join(df_fig2, by = "taxon_id") %>%
````

```
  transmute(taxon_id, Barthel_Total, Barthel_TotalSD) %>%
  filter(Barthel_Total != 0) %>% arrange(desc(Barthel_Total)) %>%
  mutate(group = ifelse(Barthel_Total > 0, "g1", "g2"))
df_fig$taxon_id = factor(df_fig$taxon_id, levels = df_fig$taxon_id)
#Grab adjusted p-values to display
rownames(df_fig) <- as.character(df_fig$taxon_id)
df_qvals <- as.data.frame(res$q_val)
colnames(df_qvals)[1] = paste0(colnames(df_qvals)[1],"ADJPVAL")
df_figY <- merge(df_fig, df_qvals, by = "row.names", all.x = TRUE)
rownames(df_figY) <- df_figY$Row.names
df_figY$Row.names <- NULL
#Replace ASV strings with genus names
df_figX <- merge(df_figY, tax_table(qd), by = "row.names", all.x =
TRUE)
df_figX$taxon_id <- df_figX$Genus
df_figX$taxon_id #fix any that are missing the genus name
#0 DA ASVs
```
E2. Is there a correlation between Barthel and cluster membership?
```{r}
nrow(qd.baseline.clust.sam)
#Count frequency of each cluster by Barthel_Total
leveneTest(Barthel_Total ~ as.factor(cluster),
data=qd.baseline.clust.sam) #p=0.82001

#Plot
barthel.clust <- ggplot(data=qd.baseline.clust.sam, aes(x=cluster,
y=Barthel_Total, colour=cluster, group=cluster)) +
  geom_boxplot() + geom_point(position="jitter", alpha=0.2) +
  xlab("Cluster membership") + ggtitle("p=0.82001") +
  theme_bw() + theme(legend.position="none") +
  scale_colour_manual(values=cluster.colours.k10)
barthel.clust
```
E3. Is there a correlation between Barthel and alpha-diversity?
```{r}
#Calculate Shannon diversity per sample
alpha = estimate_richness(qd.baseline, measures=c("Shannon"))
#Add clustering data
alpha <- cbind(alpha, as.data.frame(sample_data(qd.baseline)))

#Significant?
summary(aov(Shannon~ Barthel_Total, data=alpha)) #p=0.032605*
a1 <- aov(Shannon ~ Barthel_Total, data=alpha)
nd = data.frame(Barthel_Total = unique(alpha$Barthel_Total))
pred = predict(a1, newdata = nd, se.fit = TRUE)
pred
nd$Pred = pred$fit # adds the predicted group means to the data
frame
nd$Lo = nd$Pred - 1.96*pred$se.fit # lower bound of the confidence
interval by subtracting 1.96*se from the prediction
nd$Hi = nd$Pred + 1.96*pred$se.fit # upper bound ditto
plt = ggplot(nd, aes(x = Barthel_Total)) +
  geom_point(aes(y = Pred), size=4) +
```

````
  geom_linerange(aes(ymin = Lo, ymax = Hi), alpha=0.5) +
  theme(axis.text.x=element_text(angle=90,hjust=1,vjust=0.5))
plt

barthel.alpha.box <- ggplot(data=alpha, aes(x=Barthel_Total,
y=Shannon, colour=Barthel_Total, group=Barthel_Total)) +
  geom_boxplot() + geom_point(position="identity") +
  xlab("Barthel Total") + ggtitle("p=0.032605") +
  theme_bw() + theme(legend.position="none")
barthel.alpha.box
```

F. Investigations: CAD/CVD

F0. Colour definition
```{r}
cad.colours <- c("No"   = "#9ebcda",
                 "Yes"  = "#8856a7")
```
F1. Do any ASVs correlate with CAD?
```{r}
#ANCOMBC
ancom_da = ancombc(phyloseq = qd.baseline, formula = "CAD",
              p_adj_method = "holm", zero_cut = 0.90, lib_cut =
1000,
              group = "CAD", struc_zero = TRUE, neg_lb = FALSE, tol
= 1e-5,
              max_iter = 100, conserve = TRUE, alpha = 0.05, global
= TRUE)
res = ancom_da$res

#Visualize log fold change
samp_frac = ancom_da$samp_frac
# Replace NA with 0
samp_frac[is.na(samp_frac)] = 0
# Add pesudo-count (1) to avoid taking the log of 0
log_obs_abn = log(abundances(qd.baseline) + 1)
# Adjust the log observed abundances
log_obs_abn_adj = t(t(log_obs_abn) - samp_frac)
head(log_obs_abn_adj)

df_fig1 = data.frame(res$beta * res$diff_abn, check.names = FALSE)
%>%
  rownames_to_column("taxon_id")
df_fig2 = data.frame(res$se * res$diff_abn, check.names = FALSE) %>%
  rownames_to_column("taxon_id")
colnames(df_fig2)[-1] = paste0(colnames(df_fig2)[-1], "SD")
df_fig = df_fig1 %>% left_join(df_fig2, by = "taxon_id") %>%
  transmute(taxon_id, CADYes, CADYesSD) %>%
  filter(CADYes != 0) %>% arrange(desc(CADYes)) %>%
  mutate(group = ifelse(CADYes > 0, "g1", "g2"))
head(df_fig)
nrow(df_fig)
#17 diff_abn taxa
````

````
df_fig$taxon_id = factor(df_fig$taxon_id, levels = df_fig$taxon_id)
#Grab adjusted p-values to display
rownames(df_fig) <- as.character(df_fig$taxon_id)
df_qvals <- as.data.frame(res$q_val)
colnames(df_qvals)[1] = paste0(colnames(df_qvals)[1],"ADJPVAL")
df_figY <- merge(df_fig, df_qvals, by = "row.names", all.x = TRUE)
rownames(df_figY) <- df_figY$Row.names
df_figY$Row.names <- NULL
#Replace ASV strings with genus names
df_figX <- merge(df_figY, tax_table(qd), by = "row.names", all.x =
TRUE)
df_figX$taxon_id <- df_figX$Genus
df_figX$taxon_id #fix any that are missing the genus name
df_figX$taxon_id[9] <- "Burkholderiaceae"
df_figX$taxon_id[13] <- "Ruminococcaceae"
colnames(df_figX)
df_figX[,c(7:12)] <- NULL
df_figX$q_val <- format(df_figX$q_val, digits=3)
#df_figX$taxon_id <- factor(df_figX$taxon_id, levels =
df_figX$taxon_id)
waterfall.cad = ggplot(data = df_figX,
           aes(x = taxon_id, y = CADYes, fill = group, color =
group)) +
  geom_bar(stat = "identity", width = 0.7,
           position = position_dodge(width = 0.4)) +
  geom_errorbar(aes(ymin = CADYes - CADYesSD, ymax = CADYes +
CADYesSD), width = 0.2,
                position = position_dodge(0.05), color = "black") +
  geom_text(aes(label=CADYesADJPVAL), color="black", vjust=-0.2) +
  labs(x = NULL, y = "Test statistic (W)",
       title = "Waterfall Plot for the CAD Effect") +
  theme_bw() +
  theme(legend.position = "none",
        plot.title = element_text(hjust = 0.5),
        panel.grid.minor.y = element_blank(),
        axis.text.x = element_text(angle = 90, hjust = 1))
waterfall.cad
rownames(df_figX) <- df_figX$Row.names
```
````

F1. Correlation plots per ASV

````
```{r}
#Set up dataframe
test = cbind(as(df_figX, "data.frame"),
             as(otu_table(qd.baseline.norm)[rownames(df_figX), ],
"matrix"))
colnames(test)
test = test[,8:ncol(test)]
test = rbind(as(test, "data.frame"), t(as(sample_data(qd.baseline)
[colnames(test), ], "matrix")))
test = t(test)
test = as.data.frame(test)
options(digits=9)
nrow(test)
````

```
#Let's plot the mean relative abundance of them?
mean.relabund <- rowMeans(otu_table(qd.baseline.norm))
mean.relabund <- as.data.frame(mean.relabund)
mean.relabund$ASV <- rownames(mean.relabund)
df_figX$ASV <- rownames(df_figX)
sigtab.relabund <- merge(df_figX, mean.relabund, by.x="ASV",
by.y="ASV")
p.relabund <- ggplot(data=sigtab.relabund, aes(x=ASV,
y=mean.relabund)) +
  geom_point() +
  geom_hline(yintercept=0.001, color="gray", linetype="dashed") +
  scale_y_log10()
p.relabund
length(which(sigtab.relabund$mean.relabund>0.001)) #1 ASVs > 0.001
mean rel abund
sigtab.high <- subset(sigtab.relabund,
sigtab.relabund$mean.relabund>0.001)
rownames(sigtab.high) <- sigtab.high$ASV
tmp <- merge(sigtab.high, tax_table(qd), by = "row.names", all.x =
TRUE)
tmp$Genus

i<-1
rownames(sigtab.high)[i]
df_figX[rownames(sigtab.high)[i],]$taxon_id
test[[rownames(sigtab.high)[i]]] <-
as.numeric(as.character(as.factor(test[[rownames(sigtab.high)
[i]]])))
cad.p1 <- ggplot(data=test, aes(x=CAD,
y=.data[[rownames(sigtab.high)[i]]])) +
  geom_point(size=2, aes(colour=CAD)) +
  geom_boxplot(aes(colour=CAD), alpha=0.1) +
  xlab("CAD") + ylab("log(Relative abundance)") +
  scale_y_log10() +
  theme_bw() +
  ggtitle(paste(df_figX[rownames(sigtab.high)[i],]$taxon_id)) +
  theme(legend.position = "none") +
  scale_colour_manual(values=cad.colours)
```
F2. Is there a correlation between CAD and cluster membership?
```{r}
nrow(qd.baseline.clust.sam)
#Count frequency of each cluster by Site
#chi-squared
tbl.ev <- table(qd.baseline.clust.sam$CAD,
qd.baseline.clust.sam$cluster)
tbl.ev
chi <- chisq.test(tbl.ev) #p=0.465202
chi
contrib <- 100*chi$residuals^2/chi$statistic
corrplot(contrib, is.cor=FALSE)
corrplot(chi$residuals, is.cor=FALSE)
```

````
#Plot
counts.ev <- qd.baseline.clust.sam %>% dplyr::select(CAD, cluster)
freq.ev <- counts.ev %>%
  group_by(CAD, cluster) %>%
  summarize(n = n()) %>%
  mutate(freq = n / sum(n) * 100)

cad.clust <- ggplot(freq.ev, aes(x=CAD, y=freq, fill=cluster)) +
  geom_bar(position="stack", stat="identity") +
  scale_fill_manual(values=cluster.colours.k10) +
  scale_y_continuous(expand=c(0,0)) +
  theme_bw() +
  xlab("CAD") + ylab("Proportion of clusters per sample type") +
  ggtitle("p=0.465202")
cad.clust
```
F3. Is there a correlation between CAD and alpha-diversity?
```{r}
#Calculate Shannon diversity per sample
alpha = estimate_richness(qd.baseline, measures=c("Shannon"))
#Add clustering data
alpha <- cbind(alpha, as.data.frame(sample_data(qd.baseline)))

#Significant?
leveneTest(Shannon ~ as.factor(CAD), data=alpha) #p=0.38373

cad.alpha.box <- ggplot(data=alpha, aes(x=CAD, y=Shannon,
colour=CAD, group=CAD)) +
  geom_boxplot() + geom_point(position="jitter", alpha=0.2) +
  xlab("Sex") + ggtitle("p=0.38373") +
  theme_bw() + theme(legend.position="none") +
  scale_colour_manual(values=cad.colours)
cad.alpha.box
```

Patchwork, Figure 2
```{r}
patchwork.Fig2.b <-
  ( pb.Sex + pb.ait.Barthel + pb.CAD ) /
  ( cad.p1 + pb.Site ) +
  ( site.p1 + site.p6 + site.p9 ) +
  ( pb.ait.Month + month.p2 + pb.qPCR )
patchwork.Fig2.b
pdf("figures/Figure2.pdf", height=18, width=14)
print(patchwork.Fig2.b)
dev.off()
```
Patchwork, Sup Figure 5
```{r}
patchwork.Fig2.b.sup <-
  (site.p2 + site.p3 + site.p4) /
  (site.p5 + site.p7 + site.p8) /
  (pb.ait.Season + pb.ait.Year ) +
  plot_annotation(tag_levels = 'A')
````

```
patchwork.Fig2.b.sup
pdf("figures/SupFigure5.pdf", height=14, width=14)
print(patchwork.Fig2.b.sup)
dev.off()
```

Do any ASVs correlate with qPCR concentration?
```{r}
#ANCOMBC
ancom_da = ancombc(phyloseq = qd.baseline, formula =
"qPCR_Concentration",
              p_adj_method = "holm", zero_cut = 0.90, lib_cut =
1000,
              group = NULL, struc_zero = FALSE, neg_lb = FALSE, tol
= 1e-5,
              max_iter = 100, conserve = TRUE, alpha = 0.05, global
= FALSE)
res = ancom_da$res

#Visualize log fold change
samp_frac = ancom_da$samp_frac
# Replace NA with 0
samp_frac[is.na(samp_frac)] = 0
# Add pesudo-count (1) to avoid taking the log of 0
log_obs_abn = log(abundances(qd.baseline) + 1)
# Adjust the log observed abundances
log_obs_abn_adj = t(t(log_obs_abn) - samp_frac)
head(log_obs_abn_adj)
df_fig1 = data.frame(res$beta * res$diff_abn, check.names = FALSE)
%>%
  rownames_to_column("taxon_id")
df_fig2 = data.frame(res$se * res$diff_abn, check.names = FALSE) %>%
  rownames_to_column("taxon_id")
colnames(df_fig2)[-1] = paste0(colnames(df_fig2)[-1], "SD")
df_fig = df_fig1 %>% left_join(df_fig2, by = "taxon_id") %>%
  transmute(taxon_id, qPCR_Concentration, qPCR_ConcentrationSD) %>%
  filter(qPCR_Concentration != 0) %>%
arrange(desc(qPCR_Concentration)) %>%
  mutate(group = ifelse(qPCR_Concentration > 0, "g1", "g2"))
df_fig$taxon_id = factor(df_fig$taxon_id, levels = df_fig$taxon_id)
#Grab adjusted p-values to display
rownames(df_fig) <- as.character(df_fig$taxon_id)
df_qvals <- as.data.frame(res$q_val)
colnames(df_qvals)[1] = paste0(colnames(df_qvals)[1],"ADJPVAL")
df_figY <- merge(df_fig, df_qvals, by = "row.names", all.x = TRUE)
rownames(df_figY) <- df_figY$Row.names
df_figY$Row.names <- NULL
#Replace ASV strings with genus names
df_figX <- merge(df_figY, tax_table(qd), by = "row.names", all.x =
TRUE)
df_figX$taxon_id <- df_figX$Genus
df_figX$taxon_id #fix any that are missing the genus name
df_figX$taxon_id[4] <- "Burkholderiaceae"
df_figX[,c(7:12)] <- NULL
```

```
df_figX$qPCR_ConcentrationADJPVAL <-
format(df_figX$qPCR_ConcentrationADJPVAL, digits=3)
df_figX$taxon_id <- factor(df_figX$taxon_id, levels =
df_figX$taxon_id)
p = ggplot(data = df_figX,
           aes(x = taxon_id, y = qPCR_Concentration, fill = group,
color = group)) +
  geom_bar(stat = "identity", width = 0.7,
           position = position_dodge(width = 0.4)) +
  geom_errorbar(aes(ymin = qPCR_Concentration -
qPCR_ConcentrationSD, ymax = qPCR_Concentration +
qPCR_ConcentrationSD), width = 0.2,
                position = position_dodge(0.05), color = "black") +
  geom_text(aes(label=qPCR_ConcentrationADJPVAL), color="black",
vjust=1.1) +
  labs(x = NULL, y = "Log fold change",
       title = "Waterfall Plot for the qPCR_Concentration Effect") +
  theme_bw() +
  theme(legend.position = "none",
        plot.title = element_text(hjust = 0.5),
        panel.grid.minor.y = element_blank(),
        axis.text.x = element_text(angle = 0, hjust = 0.5))
p
rownames(df_figX) <- df_figX$Row.names
```
Correlation plots per ASV
```{r}
#Set up dataframe
test = cbind(as(df_fig, "data.frame"),
             as(otu_table(qd.norm)[rownames(df_fig), ], "matrix"))
test = test[,14:ncol(test)]
test = rbind(as(test, "data.frame"), t(as(sample_data(qd)
[colnames(test), ], "matrix")))
test = t(test)
test = as.data.frame(test)
options(digits=9)
test$qPCR_Concentration <-
as.numeric(as.character(as.factor(test$qPCR_Concentration)))

#Let's plot the mean relative abundance of them?
mean.relabund <- rowMeans(otu_table(qd.baseline.Month.norm))
mean.relabund <- as.data.frame(mean.relabund)
mean.relabund$ASV <- rownames(mean.relabund)
df_figX$ASV <- rownames(df_figX)
sigtab.relabund <- merge(df_figX, mean.relabund, by.x="ASV",
by.y="ASV")
p.relabund <- ggplot(data=sigtab.relabund, aes(x=ASV,
y=mean.relabund)) +
  geom_point() +
  geom_hline(yintercept=0.001, color="gray", linetype="dashed") +
  scale_y_log10()
p.relabund
length(which(sigtab.relabund$mean.relabund>0.001)) #6 ASVs > 0.001
mean rel abund
```

```
sigtab.high <- subset(sigtab.relabund,
sigtab.relabund$mean.relabund>0.001)
rownames(sigtab.high) <- sigtab.high$ASV
tmp <- merge(sigtab.high, tax_table(qd), by = "row.names", all.x =
TRUE)
tmp$Genus

i<-1
rownames(df_fig)[i]
df_figX$taxon_id[i]
test[[rownames(df_fig)[i]]] <-
as.numeric(as.character(as.factor(test[[rownames(df_fig)[i]]])))
qPCR.p1 <- ggplot(data=test, aes(x=qPCR_Concentration,
y=.data[[rownames(df_fig)[i]]])) +
  geom_point(size=2, aes(colour=qPCR_Concentration)) +
  geom_smooth(method=lm) +
  xlab("log(qPCR Concentration)") + ylab("log(Relative abundance)")
+
  scale_x_log10() + scale_y_log10(limits=c(0.00001,1.0)) +
  ggtitle(paste(df_figX$taxon_id[i])) +
  theme(legend.position = "none")
qPCR.p1

i<-2
rownames(df_fig)[i]
df_figX$taxon_id[i]
test[[rownames(df_fig)[i]]] <-
as.numeric(as.character(as.factor(test[[rownames(df_fig)[i]]])))
qPCR.p2 <- ggplot(data=test, aes(x=qPCR_Concentration,
y=.data[[rownames(df_fig)[i]]])) +
  geom_point(size=2, aes(colour=qPCR_Concentration)) +
  geom_smooth(method=lm) +
  xlab("log(qPCR Concentration)") + ylab("log(Relative abundance)")
+
  scale_x_log10() + scale_y_log10(limits=c(0.00001,1.0)) +
  ggtitle(paste(df_figX$taxon_id[i])) +
  theme(legend.position = "none")
qPCR.p2

i<-3
rownames(df_fig)[i]
df_figX$taxon_id[i]
test[[rownames(df_fig)[i]]] <-
as.numeric(as.character(as.factor(test[[rownames(df_fig)[i]]])))
qPCR.p3 <- ggplot(data=test, aes(x=qPCR_Concentration,
y=.data[[rownames(df_fig)[i]]])) +
  geom_point(size=2, aes(colour=qPCR_Concentration)) +
  geom_smooth(method=lm) +
  xlab("log(qPCR Concentration)") + ylab("log(Relative abundance)")
+
  scale_x_log10() + scale_y_log10(limits=c(0.00001,1.0)) +
  ggtitle(paste(df_figX$taxon_id[i])) +
  theme(legend.position = "none")
qPCR.p3
```

```
i<-4
rownames(df_fig)[i]
df_figX$taxon_id[i]
test[[rownames(df_fig)[i]]] <-
as.numeric(as.character(as.factor(test[[rownames(df_fig)[i]]])))
qPCR.p4 <- ggplot(data=test, aes(x=qPCR_Concentration,
y=.data[[rownames(df_fig)[i]]])) +
  geom_point(size=2, aes(colour=qPCR_Concentration)) +
  geom_smooth(method=lm) +
  xlab("log(qPCR Concentration)") + ylab("log(Relative abundance)")
+
  scale_x_log10() + scale_y_log10(limits=c(0.00001,1.0)) +
  ggtitle(paste(df_figX$taxon_id[i])) +
  theme(legend.position = "none")
qPCR.p4

i<-5
rownames(df_fig)[i]
df_figX$taxon_id[i]
test[[rownames(df_fig)[i]]] <-
as.numeric(as.character(as.factor(test[[rownames(df_fig)[i]]])))
qPCR.p5 <- ggplot(data=test, aes(x=qPCR_Concentration,
y=.data[[rownames(df_fig)[i]]])) +
  geom_point(size=2, aes(colour=qPCR_Concentration)) +
  geom_smooth(method=lm) +
  xlab("log(qPCR Concentration)") + ylab("log(Relative abundance)")
+
  scale_x_log10() + scale_y_log10(limits=c(0.00001,1.0)) +
  ggtitle(paste(df_figX$taxon_id[i])) +
  theme(legend.position = "none")
qPCR.p5

i<-6
rownames(df_fig)[i]
df_figX$taxon_id[i]
test[[rownames(df_fig)[i]]] <-
as.numeric(as.character(as.factor(test[[rownames(df_fig)[i]]])))
qPCR.p6 <- ggplot(data=test, aes(x=qPCR_Concentration,
y=.data[[rownames(df_fig)[i]]])) +
  geom_point(size=2, aes(colour=qPCR_Concentration)) +
  geom_smooth(method=lm) +
  xlab("log(qPCR Concentration)") + ylab("log(Relative abundance)")
+
  scale_x_log10() + scale_y_log10(limits=c(0.00001,1.0)) +
  ggtitle(paste(df_figX$taxon_id[i])) +
  theme(legend.position = "none")
qPCR.p6
```

Patchwork, SupFigure4

```{r}
layout='
AAAB
```

````
AAAC
DEFG
'
### Put Figure together ###
patchwork.ANCOMBC.qPCR <- p +
  qPCR.p1 +
  qPCR.p2 +
  qPCR.p3 +
  qPCR.p4 +
  qPCR.p5 +
  qPCR.p6 +
  plot_layout(design = layout) +
  plot_annotation(tag_levels = 'A')
patchwork.ANCOMBC.qPCR
pdf("figures/SupFigure4.pdf", height=10, width=18)
print(patchwork.ANCOMBC.qPCR)
dev.off()
```

# Figure 3:

Define colours for B (pre-C&F), E (ILI), F (post-C&F)
```{r}
type.colours <- c("B" = "#8BC34A",
                  "E" = "#FF5722",
                  "F" = "#00796B")

ILIorNot.colours <- c("B" = "#2ca25f",
                  "E" = "#FF5722",
                  "F" = "#2ca25f")
```

A. Does the overall community differ between B/F and E samples?
```{r}
#Setup data
qd.event.rare = rarefy_even_depth(qd.event, sample.size =
min(sample_sums(qd.event)), rngseed=1414)
df.event = as(sample_data(qd.event.rare), "data.frame")
#Add column that summarizes B and F samples into one variable, and E
into another
df.event$ILIorNot <- ifelse(df.event$Type.B.E.F. != "E", "Not",
"ILI")
d.event = phyloseq::distance(qd.event.rare, "bray")

qd.event.clr <- microbiome::transform(qd.event, "clr")
df.ait.event = as(sample_data(qd.event.clr), "data.frame")
#Add column that summarizes B and F samples into one variable, and E
into another
df.ait.event$ILIorNot <- ifelse(df.ait.event$Type.B.E.F. != "E",
"Not", "ILI")
dist.ait.event <- phyloseq::distance(qd.event.clr, method =
"euclidean")
iMDS.ait.event <- ordinate(qd.event.clr, method = "RDA", distance =
dist.ait.event)
````

```
#anova for Type
a.Type = adonis(d.event ~ ILIorNot + ID, df.event)
a.Type
ait.Type = adonis(dist.ait.event ~ ILIorNot + ID, df.ait.event)
ait.Type

#Plot results
pe.TypeILI <- plot_ordination(qd.event.rare, axes=c(1,2), iMDS.all)
+
  ggtitle(paste("p=",a.Type[[1]][["Pr(>F)"]][1],"/",ait.Type[[1]]
[["Pr(>F)"]][1],"***")) +
  geom_point(size = 2, colour="gray") +
  geom_point(size = 3, aes(colour=Type.B.E.F.)) +
  theme_bw() +
  theme(legend.title=element_blank(),
        legend.position=c(0.9,0.15),
        text = element_text(size=20)) +

  scale_colour_manual(na.translate = F,
                      values=ILIorNot.colours,
                      labels=c(
    paste("pre-C&F (", length(which(sample_data(qd.event)
$Type.B.E.F. == "B")), ")", sep=""),
    paste("ILI (", length(which(sample_data(qd.event)$Type.B.E.F. ==
"E")), ")", sep=""),
    paste("post-C&F (", length(which(sample_data(qd.event)
$Type.B.E.F. == "F")), ")", sep="")))
pe.TypeILI
```

B. Does alpha diversity differ between B, E, F samples?

```{r}
#Get alpha diversity values
event.alpha = estimate_richness(qd.event, measures=c("Shannon"))
event.alpha$SampleID = rownames(event.alpha)
event.test = sample_data(qd.event)
event.test = cbind(event.alpha, event.test)
event.test$SampleID <- NULL
#Significant?
leveneTest(Shannon ~ as.factor(Type.B.E.F.), data=event.test) #No

#Boxplot comparing the B-E-F alpha diversity
alpha.Type <- ggplot(event.test, aes(x=Type.B.E.F., y=Shannon,
group=Type.B.E.F., colour=Type.B.E.F.)) +
  geom_boxplot() +
  ggtitle("p=0.29654") +
  geom_jitter(alpha=0.2) +
  xlab("Sample type") +
  ylab("Shannon diversity") +
  scale_colour_manual(values=type.colours) +
  scale_x_discrete(labels=c(
    "B" = "pre-C&F",
    "E" = "ILI",
```

````
    "F" = "post-C&F")) +
  theme_bw() +
  theme(legend.position = "none")
alpha.Type
```

C. Does qPCR [] differ between B, E, F samples?
```{r}
event.test = sample_data(qd.event)
event.test <- data.frame(event.test)
#Significant?
leveneTest(qPCR_Concentration ~ as.factor(Type.B.E.F.),
data=event.test) #No

#Boxplot comparing the B-E-F alpha diversity
qPCR.Type <- ggplot(event.test, aes(x=Type.B.E.F.,
y=qPCR_Concentration, group=Type.B.E.F., colour=Type.B.E.F.)) +
  geom_boxplot() +
  ggtitle("p=0.12023") +
  geom_jitter(alpha=0.2) +
  scale_y_log10() +
  xlab("Sample type") +
  ylab("qPCR Concentration)") +
  scale_colour_manual(values=type.colours) +
  scale_x_discrete(labels=c(
    "B" = "pre-C&F",
    "E" = "ILI",
    "F" = "post-C&F")) +
  theme_bw() +
  theme(legend.position = "none")
qPCR.Type
```

Are any ASVs DA with Type.B.E.F.?
```{r}
#ANCOMBC
ancom_da = ancombc(phyloseq = qd.event, formula = "Type.B.E.F.",
              p_adj_method = "holm", zero_cut = 0.90, lib_cut =
1000,
              group = "Type.B.E.F.", struc_zero = FALSE, neg_lb =
FALSE, tol = 1e-5,
              max_iter = 100, conserve = TRUE, alpha = 0.05, global
= TRUE)
res = ancom_da$res
reg = ancom_da$res_global

#Visualize log fold change
samp_frac = ancom_da$samp_frac
# Replace NA with 0
samp_frac[is.na(samp_frac)] = 0
# Add pesudo-count (1) to avoid taking the log of 0
log_obs_abn = log(abundances(qd.baseline) + 1)
# Adjust the log observed abundances
log_obs_abn_adj = t(t(log_obs_abn) - samp_frac)
````

```
head(log_obs_abn_adj)
reg.dif <- subset(reg, reg$diff_abn == "TRUE")

#Replace ASV strings with genus names
df_figX <- merge(reg.dif, tax_table(qd), by = "row.names", all.x =
TRUE)
df_figX$taxon_id <- df_figX$Genus
df_figX$taxon_id #fix any that are missing the genus name
df_figX$taxon_id[4] <- "Alicyclobacillaceae"
df_figX$taxon_id[7] <- "Erysipelotrichaceae"
df_figX[,c(1,6:11)] <- NULL
df_figX$q_val <- format(df_figX$q_val, digits=3)
df_figX$taxon_id <- factor(df_figX$taxon_id, levels =
df_figX$taxon_id)
p = ggplot(data = df_figX,
           aes(x = taxon_id, y = W)) +
  geom_bar(stat = "identity", width = 0.7,
           position = position_dodge(width = 0.4)) +
  geom_text(aes(label=q_val), color="black", vjust=-0.2) +
  labs(x = NULL, y = "Test statistic (W)",
       title = "Waterfall Plot for the Sample Type Effect") +
  theme_bw() +
  theme(legend.position = "none",
        plot.title = element_text(hjust = 0.5),
        panel.grid.minor.y = element_blank(),
        axis.text.x = element_text(angle = 0, hjust = 0.5))
p
```
Box plots per ASV
```{r}
#Set up dataframe
test = cbind(as(reg.dif, "data.frame"),
             as(otu_table(qd.norm)[rownames(reg.dif), ], "matrix"))
test = test[,14:ncol(test)]
test = rbind(as(test, "data.frame"), t(as(sample_data(qd)
[colnames(test), ], "matrix")))
test = t(test)
test = as.data.frame(test)
options(digits=9)

i<-1
rownames(reg.dif)[i]
df_figX$taxon_id[i]
test[[rownames(reg.dif)[i]]] <-
as.numeric(as.character(as.factor(test[[rownames(reg.dif)[i]]])))
BEF.p1 <- ggplot(data=test, aes(x=Type.B.E.F.,
y=.data[[rownames(reg.dif)[i]]])) +
  geom_point(size=2, aes(colour=Type.B.E.F.)) +
  geom_boxplot(aes(colour=Type.B.E.F.), alpha=0.1) +
  xlab("Sample type") + ylab("log(Relative abundance)") +
  scale_y_log10() +
  scale_x_discrete(labels=c("B" = paste("pre-C&F (n=",
length(which(sample_data(qd)$Type.B.E.F. == "B")), ")", sep=""),
                            "E" = paste("ILI (n=",
```

```
length(which(sample_data(qd)$Type.B.E.F. == "E")), ")", sep=""),
                              "F" = paste("post-C&F (n=",
length(which(sample_data(qd)$Type.B.E.F. == "F")), ")", sep=""))) +
  theme_bw() +
  ggtitle(paste(df_figX$taxon_id[i])) +
  theme(legend.position = "none") +
  scale_colour_manual(values=type.colours)

i<-2
rownames(reg.dif)[i]
df_figX$taxon_id[i]
test[[rownames(reg.dif)[i]]] <-
as.numeric(as.character(as.factor(test[[rownames(reg.dif)[i]]])))
BEF.p2 <- ggplot(data=test, aes(x=Type.B.E.F.,
y=.data[[rownames(reg.dif)[i]]])) +
  geom_point(size=2, aes(colour=Type.B.E.F.)) +
  geom_boxplot(aes(colour=Type.B.E.F.), alpha=0.1) +
  xlab("Sample type") + ylab("log(Relative abundance)") +
  scale_y_log10() +
  scale_x_discrete(labels=c("B" = paste("pre-C&F (n=",
length(which(sample_data(qd)$Type.B.E.F. == "B")), ")", sep=""),
                              "E" = paste("ILI (n=",
length(which(sample_data(qd)$Type.B.E.F. == "E")), ")", sep=""),
                              "F" = paste("post-C&F (n=",
length(which(sample_data(qd)$Type.B.E.F. == "F")), ")", sep=""))) +
  theme_bw() +
  ggtitle(paste(df_figX$taxon_id[i])) +
  theme(legend.position = "none") +
  scale_colour_manual(values=type.colours)

i<-3
rownames(reg.dif)[i]
df_figX$taxon_id[i]
test[[rownames(reg.dif)[i]]] <-
as.numeric(as.character(as.factor(test[[rownames(reg.dif)[i]]])))
BEF.p3 <- ggplot(data=test, aes(x=Type.B.E.F.,
y=.data[[rownames(reg.dif)[i]]])) +
  geom_point(size=2, aes(colour=Type.B.E.F.)) +
  geom_boxplot(aes(colour=Type.B.E.F.), alpha=0.1) +
  xlab("Sample type") + ylab("log(Relative abundance)") +
  scale_y_log10() +
  scale_x_discrete(labels=c("B" = paste("pre-C&F (n=",
length(which(sample_data(qd)$Type.B.E.F. == "B")), ")", sep=""),
                              "E" = paste("ILI (n=",
length(which(sample_data(qd)$Type.B.E.F. == "E")), ")", sep=""),
                              "F" = paste("post-C&F (n=",
length(which(sample_data(qd)$Type.B.E.F. == "F")), ")", sep=""))) +
  theme_bw() +
  ggtitle(paste(df_figX$taxon_id[i])) +
  theme(legend.position = "none") +
  scale_colour_manual(values=type.colours)

i<-4
rownames(reg.dif)[i]
```

```
df_figX$taxon_id[i]
test[[rownames(reg.dif)[i]]] <-
as.numeric(as.character(as.factor(test[[rownames(reg.dif)[i]]])))
BEF.p4 <- ggplot(data=test, aes(x=Type.B.E.F.,
y=.data[[rownames(reg.dif)[i]]])) +
  geom_point(size=2, aes(colour=Type.B.E.F.)) +
  geom_boxplot(aes(colour=Type.B.E.F.), alpha=0.1) +
  xlab("Sample type") + ylab("log(Relative abundance)") +
  scale_y_log10() +
  scale_x_discrete(labels=c("B" = paste("pre-C&F (n=",
length(which(sample_data(qd)$Type.B.E.F. == "B")), ")", sep=""),
                            "E" = paste("ILI (n=",
length(which(sample_data(qd)$Type.B.E.F. == "E")), ")", sep=""),
                            "F" = paste("post-C&F (n=",
length(which(sample_data(qd)$Type.B.E.F. == "F")), ")", sep=""))) +
  theme_bw() +
  ggtitle(paste(df_figX$taxon_id[i])) +
  theme(legend.position = "none") +
  scale_colour_manual(values=type.colours)

i<-5
rownames(reg.dif)[i]
df_figX$taxon_id[i]
test[[rownames(reg.dif)[i]]] <-
as.numeric(as.character(as.factor(test[[rownames(reg.dif)[i]]])))
BEF.p5 <- ggplot(data=test, aes(x=Type.B.E.F.,
y=.data[[rownames(reg.dif)[i]]])) +
  geom_point(size=2, aes(colour=Type.B.E.F.)) +
  geom_boxplot(aes(colour=Type.B.E.F.), alpha=0.1) +
  xlab("Sample type") + ylab("log(Relative abundance)") +
  scale_y_log10() +
  scale_x_discrete(labels=c("B" = paste("pre-C&F (n=",
length(which(sample_data(qd)$Type.B.E.F. == "B")), ")", sep=""),
                            "E" = paste("ILI (n=",
length(which(sample_data(qd)$Type.B.E.F. == "E")), ")", sep=""),
                            "F" = paste("post-C&F (n=",
length(which(sample_data(qd)$Type.B.E.F. == "F")), ")", sep=""))) +
  theme_bw() +
  ggtitle(paste(df_figX$taxon_id[i])) +
  theme(legend.position = "none") +
  scale_colour_manual(values=type.colours)

i<-6
rownames(reg.dif)[i]
df_figX$taxon_id[i]
test[[rownames(reg.dif)[i]]] <-
as.numeric(as.character(as.factor(test[[rownames(reg.dif)[i]]])))
BEF.p6 <- ggplot(data=test, aes(x=Type.B.E.F.,
y=.data[[rownames(reg.dif)[i]]])) +
  geom_point(size=2, aes(colour=Type.B.E.F.)) +
  geom_boxplot(aes(colour=Type.B.E.F.), alpha=0.1) +
  xlab("Sample type") + ylab("log(Relative abundance)") +
  scale_y_log10() +
  scale_x_discrete(labels=c("B" = paste("pre-C&F (n=",
```

```
length(which(sample_data(qd)$Type.B.E.F. == "B")), ")", sep=""),
                              "E" = paste("ILI (n=",
length(which(sample_data(qd)$Type.B.E.F. == "E")), ")", sep=""),
                              "F" = paste("post-C&F (n=",
length(which(sample_data(qd)$Type.B.E.F. == "F")), ")", sep=""))) +
  theme_bw() +
  ggtitle(paste(df_figX$taxon_id[i])) +
  theme(legend.position = "none") +
  scale_colour_manual(values=type.colours)

i<-7
rownames(reg.dif)[i]
df_figX$taxon_id[i]
test[[rownames(reg.dif)[i]]] <-
as.numeric(as.character(as.factor(test[[rownames(reg.dif)[i]]])))
BEF.p7 <- ggplot(data=test, aes(x=Type.B.E.F.,
y=.data[[rownames(reg.dif)[i]]])) +
  geom_point(size=2, aes(colour=Type.B.E.F.)) +
  geom_boxplot(aes(colour=Type.B.E.F.), alpha=0.1) +
  xlab("Sample type") + ylab("log(Relative abundance)") +
  scale_y_log10() +
  scale_x_discrete(labels=c("B" = paste("pre-C&F (n=",
length(which(sample_data(qd)$Type.B.E.F. == "B")), ")", sep=""),
                              "E" = paste("ILI (n=",
length(which(sample_data(qd)$Type.B.E.F. == "E")), ")", sep=""),
                              "F" = paste("post-C&F (n=",
length(which(sample_data(qd)$Type.B.E.F. == "F")), ")", sep=""))) +
  theme_bw() +
  ggtitle(paste(df_figX$taxon_id[i])) +
  theme(legend.position = "none") +
  scale_colour_manual(values=type.colours)

i<-8
rownames(reg.dif)[i]
df_figX$taxon_id[i]
test[[rownames(reg.dif)[i]]] <-
as.numeric(as.character(as.factor(test[[rownames(reg.dif)[i]]])))
BEF.p8 <- ggplot(data=test, aes(x=Type.B.E.F.,
y=.data[[rownames(reg.dif)[i]]])) +
  geom_point(size=2, aes(colour=Type.B.E.F.)) +
  geom_boxplot(aes(colour=Type.B.E.F.), alpha=0.1) +
  xlab("Sample type") + ylab("log(Relative abundance)") +
  scale_y_log10() +
  scale_x_discrete(labels=c("B" = paste("pre-C&F (n=",
length(which(sample_data(qd)$Type.B.E.F. == "B")), ")", sep=""),
                              "E" = paste("ILI (n=",
length(which(sample_data(qd)$Type.B.E.F. == "E")), ")", sep=""),
                              "F" = paste("post-C&F (n=",
length(which(sample_data(qd)$Type.B.E.F. == "F")), ")", sep=""))) +
  theme_bw() +
  ggtitle(paste(df_figX$taxon_id[i])) +
  theme(legend.position = "none") +
  scale_colour_manual(values=type.colours)
```

Patchwork, SupFigure8
```{r}
layout='
AABD
AACE
FGHI
'
### Put Figure together ###
patchwork.ANCOMBC.BEF <- p +
  BEF.p1 +
  BEF.p2 +
  BEF.p3 +
  BEF.p4 +
  BEF.p5 +
  BEF.p6 +
  BEF.p7 +
  BEF.p8 +
  plot_layout(design = layout) +
  plot_annotation(tag_levels = 'A')
patchwork.ANCOMBC.BEF
pdf("figures/SupFigure8.pdf", height=10, width=24)
print(patchwork.ANCOMBC.BEF)
dev.off()
```

J. Differences between B, E, F between clusters
```{r}
#Prepare data
qd.event.clust <- qd.clust
qd.event.clustBE <- qd.event.clust[qd.event.clust$Type.B.E.F. !=
"F",]
qd.event.clustEF <- qd.event.clust[qd.event.clust$Type.B.E.F. !=
"B",]
qd.event.clustBF <- qd.event.clust[qd.event.clust$Type.B.E.F. !=
"E",]

#Count frequency of each cluster by events
#chi-squared
qd.event.clust.sam <- qd.clust.sam
tbl.ev <- table(qd.event.clust.sam$Type.B.E.F.,
qd.event.clust.sam$cluster)
tbl.ev
chi <- chisq.test(tbl.ev) #p=0.
chi
contrib <- 100*chi$residuals^2/chi$statistic
corrplot(contrib, is.cor=FALSE)
corrplot(chi$residuals, is.cor=FALSE)

#Plot
counts.ev <- qd.event.clust.sam %>% dplyr::select(Type.B.E.F.,
cluster)
freq.ev <- counts.ev %>%
  group_by(Type.B.E.F., cluster) %>%
```

```r
  summarize(n = n()) %>%
  mutate(freq = n / sum(n) * 100)

pc.Type <- ggplot(freq.ev, aes(x=Type.B.E.F., y=freq, fill=cluster))
+
  geom_bar(position="dodge", stat="identity") +
  scale_fill_manual(values=cluster.colours.k10) +
  scale_x_discrete(expand=c(0,0), labels = c(
    "B" = "pre-C&F",
    "E" = "ILI",
    "F" = "post-C&F")) +
  theme_bw() +
  xlab("Sample type") + ylab("Proportion of clusters per sample
type") +
  ggtitle("p=0.148252")
pc.Type
pdf("figures/SupFigure6.pdf", height=4, width=9)
print(pc.Type)
dev.off()
```

Patchwork, Figure 3

```{r}
### Put Figure together ###
layout = '
AA
BC
'
patchwork.Fig3a <- pe.TypeILI + #A
  alpha.Type + #B
  qPCR.Type + #C
  plot_layout(design = layout) +
  plot_annotation(tag_levels = 'A')
patchwork.Fig3a
pdf("figures/Figure3.pdf", height=16, width=10)
print(patchwork.Fig3a)
dev.off()
```

Figure 4: The effect of respiratory events on the microbiota (qd.event)

Setup universal vars qd.rare, dist, iMDS

```{r}
qd.rare = rarefy_even_depth(qd, sample.size = min(sample_sums(qd)),
rngseed=1414)
dist <- phyloseq::distance(qd.rare, method = "bray") #calc dist
matrix
iMDS <- ordinate(qd.rare, "PCoA", distance = dist)

qd.clr <- microbiome::transform(qd, "clr")
df.ait = as(sample_data(qd.clr), "data.frame")
dist.ait <- phyloseq::distance(qd.clr, method = "euclidean")
iMDS.ait <- ordinate(qd.clr, method = "RDA", distance = dist.ait)
```

```

A. When individuals had E's, do the B's differ significantly from F's?
```{r}
qd.event.noE <- subset_samples(qd.event, Type.B.E.F. != "E")
qd.event.noE <- subset_samples(qd.event.noE, hadEvent == "T")
qd.event.noE.rare = rarefy_even_depth(qd.event.noE, sample.size =
min(sample_sums(qd.event.noE)), rngseed=1414)
#Setup distance matrix
df.event.noE = as(sample_data(qd.event.noE.rare), "data.frame")
d.event.noE = phyloseq::distance(qd.event.noE.rare, "bray")

qd.event.noE.clr <- microbiome::transform(qd.event.noE, "clr")
df.ait.event.noE = as(sample_data(qd.event.noE.clr), "data.frame")
dist.ait.event.noE <- phyloseq::distance(qd.event.noE.clr, method =
"euclidean")

a.BF = adonis(d.event.noE ~ Type.B.E.F. + ID, df.event.noE)
a.BF
ait.BF = adonis(dist.ait.event.noE ~ Type.B.E.F. + ID,
df.ait.event.noE)
ait.BF

pe.BF <- plot_ordination(qd.event.noE.rare, axes=c(1,2), iMDS) +
  ggtitle(paste("p=",a.BF[[1]][["Pr(>F)"]][1])) +
  geom_point(size = 2, colour = "gray") +
  geom_point(size = 3, aes(colour=Type.B.E.F.)) +
  theme_bw() +
  theme(legend.title=element_blank(),
        legend.position = c(0.9,0.1)) +
  scale_colour_manual(na.translate = F,
                    values=c(type.colours),
                    labels = c("B" = paste("pre-C&F (",
length(which(df.event.noE$Type.B.E.F. == "B")), ")", sep=""),
                               "E" = paste("ILI (",
length(which(df.event.noE$Type.B.E.F. == "E")), ")", sep=""),
                               "F" = paste("post-C&F (",
length(which(df.event.noE$Type.B.E.F. == "F")), ")", sep="")))


pe.ait.BF <- plot_ordination(qd.event.noE.clr, axes=c(1,2),
iMDS.ait) +
  ggtitle(paste("p=","0.005","***")) +
  geom_point(size = 2, colour = "gray") +
  geom_point(size = 3, aes(colour=Type.B.E.F.)) +
  theme_bw() +
  theme(legend.title=element_blank(),
        legend.position = c(0.9,0.1)) +
  scale_colour_manual(na.translate = F,
                      values=c(type.colours),
                    labels = c("B" = paste("pre-C&F (",
length(which(df.event.noE$Type.B.E.F. == "B")), ")", sep=""),
                               "E" = paste("ILI (",

````
length(which(df.event.noE$Type.B.E.F. == "E")), ")", sep=""),
                              "F" = paste("post-C&F (",
length(which(df.event.noE$Type.B.E.F. == "F")), ")", sep="")))
```

B. Do E's affect the microbiota in F's?
```{r}
qd.event.F <- subset_samples(qd.event, Type.B.E.F. == "F")
qd.event.F.rare = rarefy_even_depth(qd.event.F, sample.size =
min(sample_sums(qd.event.F)), rngseed=1414)
#Setup distance matrix
df.event.F = as(sample_data(qd.event.F.rare), "data.frame")
d.event.F = phyloseq::distance(qd.event.F.rare, "bray")

qd.event.F.clr <- microbiome::transform(qd.event.F, "clr")
df.ait.event.F = as(sample_data(qd.event.F.clr), "data.frame")
dist.ait.event.F <- phyloseq::distance(qd.event.F.clr, method =
"euclidean")

a.FwwoE = adonis(d.event.F ~ hadEvent, df.event.F)
a.FwwoE
ait.FwwoE = adonis(dist.ait.event.F ~ hadEvent, df.ait.event.F)
ait.FwwoE

p.FwwoE <- plot_ordination(qd.event.F.rare, axes=c(1,2), iMDS) +
  ggtitle(paste("p=",a.FwwoE[[1]][["Pr(>F)"]][1])) +
  geom_point(size = 2, colour = "gray") +
  geom_point(size = 3, aes(colour=hadEvent)) +
  theme_bw() +
  theme(legend.title=element_blank(),
        legend.position = c(0.87,0.1)) +
  scale_colour_manual(na.translate = F,
                    values=c("T" = "#B2DFDB",
                             "F" = "#00796B"),
                    labels = c("T" = paste("had ILI (",
length(which(df.event.F$hadEvent == "T")), ")", sep=""),
                              "F" = paste("did not have ILI (",
length(which(df.event.F$hadEvent == "F")), ")", sep="")))

p.ait.FwwoE <- plot_ordination(qd.event.F.clr, axes=c(1,2),
iMDS.ait) +
  ggtitle(paste("p=","0.186")) +
  theme_bw() +
  geom_point(size = 2, colour = "gray") +
  geom_point(size = 3, aes(colour=hadEvent)) +
  theme(legend.title=element_blank(),
        legend.position = c(0.9,0.1)) +
  scale_colour_manual(na.translate = F,
                      values=c("T" = "#B2DFDB",
                             "F" = "#00796B"),
                    labels = c("T" = paste("had ILI (",
length(which(df.event.F$hadEvent == "T")), ")", sep=""),
                              "F" = paste("did not have ILI (",
length(which(df.event.F$hadEvent == "F")), ")", sep="")))
````

````
```
````

C. Differences between B & F in those with Es by cluster

````
```{r}
#Subset qd.event to only those individuals who had an Event
qd.event.clust.sam.e <-
qd.event.clust.sam[qd.event.clust.sam$hadEvent == "T",]
qd.event.clust.sam.e.bf <-
qd.event.clust.sam.e[qd.event.clust.sam.e$Type.B.E.F. != "E",]
#Count frequency of each cluster by timepoint
#chi-squared
tbl.ev <- table(qd.event.clust.sam.e.bf$Type.B.E.F.,
qd.event.clust.sam.e.bf$cluster)
tbl.ev
chi <- chisq.test(tbl.ev) #p=0.635
chi

counts.hadEvent <- qd.event.clust.sam %>% dplyr::select(hadEvent,
Type.B.E.F., cluster)
counts.hadEvent = counts.hadEvent %>%
  group_by(hadEvent, Type.B.E.F., cluster,
           .drop=FALSE) %>%
    tally()
counts.hadEvent
counts.hadEvent <- counts.hadEvent %>% filter(hadEvent == "T")
counts.hadEvent <- counts.hadEvent %>% filter(Type.B.E.F. != "E")
counts.hadEvent
p.clustBF <- ggplot(counts.hadEvent, aes(x=Type.B.E.F., y=n,
fill=cluster)) +
  geom_bar(position="dodge", stat="identity") +
  scale_fill_manual(values=c(cluster.colours.k10)) +
  theme_bw() +
  theme(legend.position="none") +
  xlab("Sample type") + ylab("# of Samples per cluster") +
  ggtitle("p=0.528194") +
  scale_x_discrete(labels=c(
    "B" = "pre-C&F",
    "F" = "post-C&F"
  ))
```
````

D. Differences in Fs in those with and without Es by cluster

````
```{r}
#subset qd.event to only include Followup samples
qd.event.clust.sam.f <-
qd.event.clust.sam[qd.event.clust.sam$Type.B.E.F. == "F",]
#Count frequency of each cluster by whether individual had Event or
not
#chi-squared
tbl.ev <- table(qd.event.clust.sam.f$hadEvent,
qd.event.clust.sam.f$cluster)
tbl.ev
chi <- chisq.test(tbl.ev) #p=
chi
````

````
counts.hadEvent <- qd.event.clust.sam %>% dplyr::select(hadEvent,
Type.B.E.F., cluster)
counts.hadEvent = counts.hadEvent %>%
  group_by(hadEvent, Type.B.E.F., cluster,
           .drop = FALSE) %>%
    tally()

counts.hadEvent <- counts.hadEvent %>% filter(Type.B.E.F. == "F")
p.clustFwwoE <- ggplot(counts.hadEvent, aes(x=hadEvent, y=n,
fill=cluster)) +
  geom_bar(position="dodge", stat="identity") +
  scale_fill_manual(values=c(cluster.colours.k10)) +
  theme_bw() +
  theme(legend.position="none") +
  xlab("Boolean: had Event") + ylab("# of Samples per cluster") +
  ggtitle("p=0.1333477") +
  scale_x_discrete(labels=c(
    "F" = "did not have ILI",
    "T" = "had ILI"
  ))
```

E. pcoa of all samples by cluster
```{r}
#Plot
clust.pcoa <- plot_ordination(qd.rare, axes=c(1,2), iMDS) +
  theme_bw() +
  theme(legend.title=element_blank(),
        legend.position="none") +
  geom_point(size = 3, shape=21, colour="black", aes(fill=cluster))
+
  scale_fill_manual(values=c(cluster.colours.k10))
clust.pcoa
```

F. pcoa of all samples by cluster, with lines
```{r}
#Plot with lines
indiv.pcoa.data <- plot_ordination(qd.rare, axes=c(1,2), iMDS,
justDF = TRUE)

lines.pcoa <- plot_ordination(qd.rare, axes=c(1,2), iMDS) +
  theme_bw() +
  theme(legend.title=element_blank(),
        legend.position="none") +
  geom_path(data = indiv.pcoa.data[order(indiv.pcoa.data$ID,
indiv.pcoa.data$Swab, indiv.pcoa.data$Type.B.E.F.),],
            colour = "gray", alpha=0.8,
            #aes(group=ID, colour=ID),
            arrow = arrow(length = unit(0.1, "inches"), ends="last",
type="closed")) +
  geom_point(size = 3, colour = "gray") +
  geom_point(size = 3, alpha=0.5, aes(colour=cluster)) +
````

````
  scale_colour_manual(values=c(cluster.colours.k10))
lines.pcoa
```

G-J. Highlight certain individuals in the plot from E
```{r}
indiv.pcoa.data <- plot_ordination(qd.rare, axes=c(1,2), iMDS,
justDF = TRUE)

p.all3same <- indiv.pcoa.data[indiv.pcoa.data$ID =="",] #IDs removed
indiv.same <- plot_ordination(qd.rare, axes=c(1,2), iMDS) +
  theme_bw() +
  theme(legend.title=element_blank(),
        legend.position="none") +
  geom_path(data = indiv.pcoa.data[order(indiv.pcoa.data$ID,
indiv.pcoa.data$Swab, indiv.pcoa.data$Type.B.E.F.),],
            aes(group=ID, colour=ID), alpha=0.8,
            arrow = arrow(length = unit(0.1, "inches"), ends="last",
type="closed")) +
  geom_point(size = 2, shape=21, colour="black", fill="gray") +
  geom_rect(mapping=aes(xmin=-0.49, xmax=0.435, ymin=-0.38,
ymax=0.59), fill="white", color="white", alpha=0.01) +
  geom_line(data=p.all3same, colour="black", alpha=1) +
  geom_segment(data=p.all3same, aes(xend=lead(Axis.1),
yend=lead(Axis.2)),
               arrow = arrow(length = unit(0.1, "inches"),
ends="last", type="closed")) +
  geom_point(data=p.all3same, size=3, shape=21, colour="black",
aes(fill=cluster)) +
  scale_colour_manual(values=c(rep("gray",234))) +
  scale_fill_manual(values=c(cluster.colours.k10))

p.eventback <- indiv.pcoa.data[indiv.pcoa.data$ID =="",]
indiv.eventback <- plot_ordination(qd.rare, axes=c(1,2), iMDS) +
  theme_bw() +
  theme(legend.title=element_blank(),
        legend.position="none") +
  geom_path(data = indiv.pcoa.data[order(indiv.pcoa.data$ID,
indiv.pcoa.data$Swab, indiv.pcoa.data$Type.B.E.F.),],
            aes(group=ID, colour=ID), alpha=0.8,
            arrow = arrow(length = unit(0.1, "inches"), ends="last",
type="closed")) +
  geom_point(size = 2, shape=21, colour="black", fill="gray") +
  geom_rect(mapping=aes(xmin=-0.49, xmax=0.435, ymin=-0.38,
ymax=0.59), fill="white", color="white", alpha=0.01) +
  geom_line(data=p.eventback[1:2,], colour="black", alpha=1, arrow =
arrow(length = unit(0.1, "inches"), ends="last", type="closed")) +
  geom_line(data=p.eventback[c(2,3),], colour="black", alpha=1,
arrow = arrow(length = unit(0.1, "inches"), ends="first",
type="closed")) +
  geom_line(data=p.eventback[3:4,], colour="black", alpha=1, arrow =
arrow(length = unit(0.1, "inches"), ends="first", type="closed")) +
  geom_line(data=p.eventback[4:5,], colour="black", alpha=1, arrow =
arrow(length = unit(0.1, "inches"), ends="last", type="closed")) +
````

```
  geom_line(data=p.eventback[5:6,], colour="black", alpha=1, arrow =
arrow(length = unit(0.1, "inches"), ends="last", type="closed")) +
  geom_point(data=p.eventback, size=3, shape=21, colour="black",
aes(fill=cluster)) +
  scale_colour_manual(values=c(rep("gray",234))) +
  scale_fill_manual(values=c(cluster.colours.k10))
indiv.eventback

p.all3diff <- indiv.pcoa.data[indiv.pcoa.data$ID =="",]
indiv.diff <- plot_ordination(qd.rare, axes=c(1,2), iMDS) +
  theme_bw() +
  theme(legend.title=element_blank(),
        legend.position="none") +
  geom_path(data = indiv.pcoa.data[order(indiv.pcoa.data$ID,
indiv.pcoa.data$Swab, indiv.pcoa.data$Type.B.E.F.),],
            aes(group=ID, colour=ID), alpha=0.8,
            arrow = arrow(length = unit(0.1, "inches"), ends="last",
type="closed")) +
  geom_point(size = 2, shape=21, colour="black", fill="gray") +
  geom_rect(mapping=aes(xmin=-0.49, xmax=0.435, ymin=-0.38,
ymax=0.59), fill="white", color="white", alpha=0.01) +
  geom_line(data=p.all3diff[1:2,], colour="black", alpha=1, arrow =
arrow(length = unit(0.1, "inches"), ends="last", type="closed")) +
  geom_line(data=p.all3diff[c(2,3),], colour="black", alpha=1, arrow
= arrow(length = unit(0.1, "inches"), ends="last", type="closed")) +
  geom_point(data=p.all3diff, size=3, shape=21, colour="black",
aes(fill=cluster)) +
  scale_colour_manual(values=c(rep("gray",234))) +
  scale_fill_manual(values=c(cluster.colours.k10))

p.followchange <- indiv.pcoa.data[indiv.pcoa.data$ID=="",]
indiv.followchange <- plot_ordination(qd.rare, axes=c(1,2), iMDS) +
  theme_bw() +
  theme(legend.title=element_blank(),
        legend.position="none") +
  geom_path(data = indiv.pcoa.data[order(indiv.pcoa.data$ID,
indiv.pcoa.data$Swab, indiv.pcoa.data$Type.B.E.F.),],
            aes(group=ID, colour=ID), alpha=0.8,
            arrow = arrow(length = unit(0.1, "inches"), ends="last",
type="closed")) +
  geom_point(size = 2, shape=21, colour="black", fill="gray") +
  geom_rect(mapping=aes(xmin=-0.49, xmax=0.435, ymin=-0.38,
ymax=0.59), fill="white", color="white", alpha=0.01) +
  geom_line(data=p.followchange[1:2,], colour="black", alpha=1,
arrow = arrow(length = unit(0.1, "inches"), ends="first",
type="closed")) +
  geom_line(data=p.followchange[2:3,], colour="black", alpha=1,
arrow = arrow(length = unit(0.1, "inches"), ends="first",
type="closed")) +
  geom_line(data=p.followchange[3:4,], colour="black", alpha=1,
arrow = arrow(length = unit(0.1, "inches"), ends="first",
type="closed")) +
  geom_point(data=p.followchange, size=3, shape=21, colour="black",
aes(fill=cluster)) +
```

```
  scale_colour_manual(values=c(rep("gray",234))) +
  scale_fill_manual(values=c(cluster.colours.k10))
```

J&K. Use per-individual manual definitions of the below added to the mapping file
  same: no event, all samples per inidividual same cluster
  change: no event, not all samples per individual in the same cluster
  eventsame: event, all samples per individual same cluster
  eventreturn: event, baseline and followup same cluster but event different
  followupchange: event, baseline and event same cluster but followup different
  eventchange: event, not the above and >1 cluster per individual

```{r}
indiv.pcoa.data <- plot_ordination(qd.rare, axes=c(1,2), iMDS,
justDF = TRUE)
colnames(indiv.pcoa.data)
perid <- indiv.pcoa.data[c(6,7,116, 115)] #ID, JJ_ML, Swab,
Type.B.E.F., hadEvent, cluster, cluster_movment
head(perid)
perid.uniq <- perid[!duplicated(perid$ID),]
perid.uniq <- subset(perid.uniq, perid.uniq$cluster_movement !=
"na")
perid.uniq <- subset(perid.uniq, perid.uniq$JJ_ML != "JJ")
perid.uniq$cluster_movement <- factor(perid.uniq$cluster_movement,
                                      levels=c("same","change",

"eventsame","eventchange","eventreturn","followchange"))

counts.id = perid.uniq %>% dplyr::select(cluster_movement)
counts.id = counts.id %>%
  group_by(cluster_movement) %>%
  tally()
counts.id

#detailed results
plot.movement <- ggplot(counts.id, aes(x=cluster_movement, y=n)) +
  geom_bar(stat="identity", position="dodge") +
  scale_y_continuous(expand=c(0,0)) +
  ylab("# of individuals") +
  theme_bw() +
  theme(axis.title.x=element_blank(),
        axis.text.x = element_text(angle = 45, hjust = 1))
plot.movement

#detailed results, split into 2 plots
counts.id.event <- subset(counts.id, counts.id$cluster_movement !=
c("same", "change"))
counts.id.event$cluster_movement <-
factor(counts.id.event$cluster_movement,
                                           levels = c("eventsame",
"eventreturn",
```

```
                                                     "eventchange",
"followchange"))
plot.event.movement <- ggplot(counts.id.event,
aes(x=cluster_movement, y=n)) +
  geom_bar(stat="identity", position="dodge") +
  ylab("# of individuals with ILI") +
  scale_x_discrete(labels=c("eventsame" = "No cluster movement (1)",
                            "eventchange" = "Change with ILI,\n no
return to baseline (3)",
                            "eventreturn" = "Change with ILI,\n
return to baseline (2)",
                            "followchange" = "Change following ILI
(4)")) +
  scale_y_continuous(limits=c(0,40)) +
  theme_bw() +
  theme(axis.title.x=element_blank(),
        axis.text.x = element_text(angle = 0))
plot.event.movement

counts.id.health <- subset(counts.id, counts.id$cluster_movement ==
c("same", "change"))
plot.health.movement <- ggplot(counts.id.health,
aes(x=cluster_movement, y=n)) +
  geom_bar(stat="identity", position="dodge") +
  ylab("# of individuals") +
  scale_x_discrete(labels=c("same" = "No cluster\n movement",
                            "change" = "Cluster\n movement")) +
  scale_y_continuous(limits=c(0,40)) +
  theme_bw() +
  theme(axis.title.x=element_blank(),
        axis.text.x = element_text(angle = 0))
plot.health.movement

#Statistics on cluster movement in events vs. healthys
perid.uniq$healthtype <- ifelse((perid.uniq$cluster_movement ==
"same" | perid.uniq$cluster_movement == "change"), "healthy",
"event")
perid.uniq$cluster_movement_simple <- ifelse(grepl('same',
perid.uniq$cluster_movement), "same", "change")
perid.uniq
table(perid.uniq$cluster_movement_simple, perid.uniq$healthtype)
chisq <- chisq.test(x=perid.uniq$cluster_movement_simple,
y=perid.uniq$healthtype)
chisq
corrplot(chisq$residuals, is.cor = FALSE)

#Proportion of individuals in each group
perid.uniq.long <- melt(perid.uniq)
head(perid.uniq.long)
temp <- data.frame(change= c(48.7,76.2), healthtype = c("did not
have ILI", "had ILI"))
temp
plot.movement.proportions <- ggplot(temp, aes(x=healthtype,
y=change)) +
```

```
  geom_bar(stat="identity") +
  ylab("% of individuals with cluster movement") + xlab("") +
  scale_y_continuous(limit=c(0,100)) +
  geom_signif(y_position=c(84), xmin=c(1), xmax=c(2),
annotation=c("p=0.006**"))
plot.movement.proportions
```

Is there a difference in metadata variables between healthy sames and changers?

```{r}
colnames(indiv.pcoa.data)
perid <- indiv.pcoa.data
colnames(perid)
perid.uniq <- perid[!duplicated(perid$ID),]
table(perid.uniq$cluster_movement)
perid.uniq <- subset(perid.uniq, cluster_movement != "na")
perid.uniq <- subset(perid.uniq, cluster_movement != "same")
perid.uniq <- subset(perid.uniq, cluster_movement != "change")
table(perid.uniq$cluster_movement)
perid.uniq$cluster_movement <- factor(perid.uniq$cluster_movement,
                                      levels=c(#"same","change",

"eventsame","eventreturn","eventchange","followchange"))
table(perid.uniq$cluster_movement)
perid.uniq$cluster_movement_simple <- ifelse(grepl('same',
perid.uniq$cluster_movement), "same", "change")
table(perid.uniq$cluster_movement_simple)

#Tests for correlation of metadata with cluster_movement per
individual
#Age
summary(aov(Age_at_enrollment ~ as.factor(cluster_movement),
data=perid.uniq))
summary(aov(Age_at_enrollment ~ as.factor(cluster_movement_simple),
data=perid.uniq))
#Sex
chi <- chisq.test(perid.uniq$Sex, perid.uniq$cluster_movement)
chi <- chisq.test(perid.uniq$Sex,
perid.uniq$cluster_movement_simple)
chi
#Site
chi <- chisq.test(perid.uniq$Site, perid.uniq$cluster_movement)
chi <- chisq.test(perid.uniq$Site,
perid.uniq$cluster_movement_simple)
chi
#Probiotics
chi <- chisq.test(perid.uniq$Allocation_Group_Probiotics,
perid.uniq$cluster_movement)
chi <- chisq.test(perid.uniq$Allocation_Group_Probiotics,
perid.uniq$cluster_movement_simple)
chi
#Smoker
chi <- chisq.test(perid.uniq$Smoker, perid.uniq$cluster_movement)
```

```
chi <- chisq.test(perid.uniq$Smoker,
perid.uniq$cluster_movement_simple)
chi
#Num_Medications
summary(aov(Num_Medications ~ as.factor(cluster_movement),
data=perid.uniq))
summary(aov(Num_Medications ~ as.factor(cluster_movement_simple),
data=perid.uniq))
#Influenza_vacc_this_season
chi <- chisq.test(perid.uniq$Influenza_vacc_this_season,
perid.uniq$cluster_movement)
chi
#Influenza_seasonal_vaccine_last_season
chi <- chisq.test(perid.uniq$Influenza_seasonal_vaccine_last_season,
perid.uniq$cluster_movement)
chi <- chisq.test(perid.uniq$Influenza_seasonal_vaccine_last_season,
perid.uniq$cluster_movement_simple)
chi
#Influenza_vaccine_ever
chi <- chisq.test(perid.uniq$Influenza_vaccine_ever,
perid.uniq$cluster_movement)
chi <- chisq.test(perid.uniq$Influenza_vaccine_ever,
perid.uniq$cluster_movement_simple)
chi
#Has_pt_received_pneumonia_vaccine
chi <- chisq.test(perid.uniq$Has_pt_received_pneumonia_vaccine,
perid.uniq$cluster_movement)
chi <- chisq.test(perid.uniq$Has_pt_received_pneumonia_vaccine,
perid.uniq$cluster_movement_simple)
chi
#Is_pt_in_shared_room
chi <- chisq.test(perid.uniq$Is_pt_in_shared_room,
perid.uniq$cluster_movement)
chi <- chisq.test(perid.uniq$Is_pt_in_shared_room,
perid.uniq$cluster_movement_simple)
chi
#Barthel_Total
summary(aov(Barthel_Total ~ as.factor(cluster_movement),
data=perid.uniq))
summary(aov(Barthel_Total ~ as.factor(cluster_movement_simple),
data=perid.uniq))
#COPD
chi <- chisq.test(perid.uniq$COPD, perid.uniq$cluster_movement)
chi <- chisq.test(perid.uniq$COPD,
perid.uniq$cluster_movement_simple)
chi
#CHF
chi <- chisq.test(perid.uniq$CHF, perid.uniq$cluster_movement)
chi <- chisq.test(perid.uniq$CHF,
perid.uniq$cluster_movement_simple)
chi
#CAD
chi <- chisq.test(perid.uniq$CAD, perid.uniq$cluster_movement)
chi <- chisq.test(perid.uniq$CAD,
```

```
perid.uniq$cluster_movement_simple)
chi
#Anemia
chi <- chisq.test(perid.uniq$Anemia, perid.uniq$cluster_movement)
chi <- chisq.test(perid.uniq$Anemia,
perid.uniq$cluster_movement_simple)
chi
#Dementia
chi <- chisq.test(perid.uniq$Dementia, perid.uniq$cluster_movement)
chi <- chisq.test(perid.uniq$Dementia,
perid.uniq$cluster_movement_simple)
chi
#CVA.Stroke
chi <- chisq.test(perid.uniq$CVA.Stroke,
perid.uniq$cluster_movement)
chi <- chisq.test(perid.uniq$CVA.Stroke,
perid.uniq$cluster_movement_simple)
chi
#DM
chi <- chisq.test(perid.uniq$DM, perid.uniq$cluster_movement)
chi <- chisq.test(perid.uniq$DM, perid.uniq$cluster_movement_simple)
chi
#Hypothyroid
chi <- chisq.test(perid.uniq$Hypothyroid,
perid.uniq$cluster_movement)
chi <- chisq.test(perid.uniq$Hypothyroid,
perid.uniq$cluster_movement_simple)
chi
#Num_Comorbidities
summary(aov(Num_Comorbidities ~ as.factor(cluster_movement),
data=perid.uniq))
summary(aov(Num_Comorbidities ~ as.factor(cluster_movement_simple),
data=perid.uniq))
#Seizures
chi <- chisq.test(perid.uniq$Seizures, perid.uniq$cluster_movement)
chi <- chisq.test(perid.uniq$Seizures,
perid.uniq$cluster_movement_simple)
chi
#Cancer
chi <- chisq.test(perid.uniq$Cancer, perid.uniq$cluster_movement)
chi <- chisq.test(perid.uniq$Cancer,
perid.uniq$cluster_movement_simple)
chi
#IL1B
summary(aov(IL1B ~ as.factor(cluster_movement), data=perid.uniq))
summary(aov(IL1B ~ as.factor(cluster_movement_simple),
data=perid.uniq))
#IL6
summary(aov(IL6 ~ as.factor(cluster_movement), data=perid.uniq))
summary(aov(IL6 ~ as.factor(cluster_movement_simple),
data=perid.uniq))
#TNFA
summary(aov(TNFA ~ as.factor(cluster_movement), data=perid.uniq))
summary(aov(TNFA ~ as.factor(cluster_movement_simple),
```

````
data=perid.uniq))
```

# Sup Figure 11: Movement between clusters

Export to gephi
```{r}
#tutorial https://igraph.org/r/doc/graph_from_adjacency_matrix.html
#Define colour dataframe
cluster.colours.k10.df <- as.data.frame(cluster.colours.k10)

#Helper function, via: https://stackoverflow.com/questions/16875547/
#using-igraph-how-to-force-curvature-when-arrows-point-in-opposite-
directions
autocurve.edges2 <-function (graph, start = 0.5)
{
    cm <- count.multiple(graph)
    mut <-is.mutual(graph)  #are connections mutual?
    el <- apply(get.edgelist(graph, names = FALSE), 1, paste,
        collapse = ":")
    ord <- order(el)
    res <- numeric(length(ord))
    p <- 1
    while (p <= length(res)) {
        m <- cm[ord[p]]
        mut.obs <-mut[ord[p]] #are the connections mutual for this
point?
        idx <- p:(p + m - 1)
        if (m == 1 & mut.obs==FALSE) { #no mutual conn = no curve
            r <- 0
        }
        else {
            r <- seq(-start, start, length = m)
        }
        res[ord[idx]] <- r
        p <- p + m
    }
    res
}

head(indiv.pcoa.data)
indiv.pcoa.data$CollectionDate.Y.M.D. <-
as.Date(indiv.pcoa.data$CollectionDate.Y.M.D., format="%d/%m/%Y")
indiv.pcoa.data1 <- indiv.pcoa.data[order(indiv.pcoa.data$ID,
indiv.pcoa.data$CollectionDate.Y.M.D., indiv.pcoa.data$Swab,
indiv.pcoa.data$Type.B.E.F.),]
head(indiv.pcoa.data1)
adjmatrix <- indiv.pcoa.data1[,c('ID', 'CollectionDate.Y.M.D.',
'Swab', 'Type.B.E.F.', 'cluster')]
head(adjmatrix)
#add column of event_cluster information
adjmatrix$EveClust <- paste(adjmatrix$Type.B.E.F.,
adjmatrix$cluster, sep="_")
#make an edgelist from the ordered cluster information
````

```
adjlist <- data.frame(from = adjmatrix$EveClust[-
length(adjmatrix$EveClust)],
                      to = adjmatrix$EveClust[-1],
                      idsame = ifelse(adjmatrix$ID[-
length(adjmatrix$ID)] == adjmatrix$ID[-1], TRUE, FALSE))
#unlink end of ID1 and beginning of ID2
adjlist <- subset(adjlist, adjlist$idsame == TRUE)
adjlist$idsame <- NULL
head(adjlist)
library("igraph")
gel <- graph.data.frame(adjlist)
gad <- get.adjacency(gel, sparse=FALSE)
g <- graph_from_adjacency_matrix(gad, mode = "directed", weighted =
TRUE)
table(adjmatrix$EveClust)
V(g) #3  5  8  6  10 2  7  1  9  4
#V(g)$size <- c(61, 163, 129, 65, 42, 62, 16, 12, 5, 5)
#              B_9 B_4 B_2 B_8 E_8 B_6 E_9 B_1 E_1 B_5 E_5 E_2 B_3
B_7 E_3 E_7 E_4 F_4 F_8 F_6 F_1 F_2 F_9 F_3 F_7 E_6 F_5
V(g)$size <- c(33, 21, 31, 16, 6, 16,  21, 19, 10,  5,  1, 15,  7,
2,  1,  1,  1, 13, 12, 14, 21, 22, 32,  7,  5,  1,  1)
#V(g)$size <-
c(18,59,18,5,13,8,2,47,23,51,26,13,22,13,5,21,4,3,19,15,3,3,1,6,
#               2,1,2,31,18,17,41,25,11,7,1,5,1)
#V(g)$size <- V(g)$size/10+1
#E(g)$weight <- E(g)$weight/10+1
V(g)["B_5"]$color <- cluster.colours.k10.df["5",]
V(g)["B_2"]$color <- cluster.colours.k10.df["2",]
V(g)["B_8"]$color <- cluster.colours.k10.df["8",]
V(g)["B_6"]$color <- cluster.colours.k10.df["6",]
V(g)["B_3"]$color <- cluster.colours.k10.df["3",]
V(g)["B_1"]$color <- cluster.colours.k10.df["1",]
V(g)["B_9"]$color <- cluster.colours.k10.df["9",]
V(g)["B_7"]$color <- cluster.colours.k10.df["7",]
V(g)["B_4"]$color <- cluster.colours.k10.df["4",]
V(g)["E_5"]$color <- cluster.colours.k10.df["5",]
V(g)["E_2"]$color <- cluster.colours.k10.df["2",]
V(g)["E_6"]$color <- cluster.colours.k10.df["6",]
V(g)["E_3"]$color <- cluster.colours.k10.df["3",]
V(g)["E_8"]$color <- cluster.colours.k10.df["8",]
V(g)["E_1"]$color <- cluster.colours.k10.df["1",]
V(g)["E_9"]$color <- cluster.colours.k10.df["9",]
V(g)["E_7"]$color <- cluster.colours.k10.df["7",]
V(g)["E_4"]$color <- cluster.colours.k10.df["4",]
V(g)["F_5"]$color <- cluster.colours.k10.df["5",]
V(g)["F_2"]$color <- cluster.colours.k10.df["2",]
V(g)["F_3"]$color <- cluster.colours.k10.df["3",]
V(g)["F_8"]$color <- cluster.colours.k10.df["8",]
V(g)["F_6"]$color <- cluster.colours.k10.df["6",]
V(g)["F_7"]$color <- cluster.colours.k10.df["7",]
V(g)["F_4"]$color <- cluster.colours.k10.df["4",]
V(g)["F_1"]$color <- cluster.colours.k10.df["1",]
V(g)["F_9"]$color <- cluster.colours.k10.df["9",]
```

````
#Export to Gephi
library("gephi")
gephi_write_edges(g, "gephi_edges-alldata.csv")
```

Figure 5 (part of): Movement between states by cluster- B-E-F active
vs. placebo
```{r}
head(indiv.pcoa.data)
indiv.pcoa.data$CollectionDate.Y.M.D. <-
as.Date(indiv.pcoa.data$CollectionDate.Y.M.D., format="%d/%m/%Y")
indiv.pcoa.data1 <- indiv.pcoa.data[order(indiv.pcoa.data$ID,
indiv.pcoa.data$CollectionDate.Y.M.D., indiv.pcoa.data$Swab,
indiv.pcoa.data$Type.B.E.F.),]
head(indiv.pcoa.data1)
adjmatrix <- indiv.pcoa.data1[,c('ID', 'CollectionDate.Y.M.D.',
'Swab', 'Type.B.E.F.', 'cluster', 'JJ_ML', 'hadEvent',
'Allocation_Group_Probiotics')]
head(adjmatrix)
adjmatrix.ML <- subset(adjmatrix, adjmatrix$JJ_ML == "ML")
head(adjmatrix.ML)
adjmatrix.ML.eT <- subset(adjmatrix.ML, adjmatrix.ML$hadEvent ==
"T")
#add column of event_cluster information
adjmatrix.ML.eT$EveClust <- paste(adjmatrix.ML.eT$Type.B.E.F.,
adjmatrix.ML.eT$cluster, sep="_")

adjmatrix.ML.eT.A <- subset(adjmatrix.ML.eT,
adjmatrix.ML.eT$Allocation_Group_Probiotics == "Active")
adjmatrix.ML.eT.P <- subset(adjmatrix.ML.eT,
adjmatrix.ML.eT$Allocation_Group_Probiotics == "Placebo")


adjlist <- data.frame(from = adjmatrix.ML.eT.A$EveClust[-
length(adjmatrix.ML.eT.A$EveClust)],
                      to = adjmatrix.ML.eT.A$EveClust[-1],
                      idsame = ifelse(adjmatrix.ML.eT.A$ID[-
length(adjmatrix.ML.eT.A$ID)] == adjmatrix.ML.eT.A$ID[-1], TRUE,
FALSE))
#unlink end of ID1 and beginning of ID2
adjlist <- subset(adjlist, adjlist$idsame == TRUE)
adjlist$idsame <- NULL
head(adjlist)
library("igraph")
gel.Active <- graph.data.frame(adjlist)
gad.Active <- get.adjacency(gel.Active, sparse=FALSE)
g.Active <- graph_from_adjacency_matrix(gad.Active, mode =
"directed", weighted = TRUE)
table(adjmatrix.ML.eT.A$EveClust)
V(g.Active)
V(g.Active)$size <- c(3, 9, 7, 2, 1, 6, 5, 2, 1, 2, 1, 1, 3, 5, 1,
3, 1, 1, 1)
V(g.Active)["B_2"]$color <- cluster.colours.k10.df["2",]
V(g.Active)["B_8"]$color <- cluster.colours.k10.df["8",]
````

```
V(g.Active)["B_6"]$color <- cluster.colours.k10.df["6",]
V(g.Active)["B_3"]$color <- cluster.colours.k10.df["3",]
V(g.Active)["B_1"]$color <- cluster.colours.k10.df["1",]
V(g.Active)["B_9"]$color <- cluster.colours.k10.df["9",]
V(g.Active)["B_4"]$color <- cluster.colours.k10.df["4",]
V(g.Active)["E_2"]$color <- cluster.colours.k10.df["2",]
V(g.Active)["E_6"]$color <- cluster.colours.k10.df["6",]
V(g.Active)["E_3"]$color <- cluster.colours.k10.df["3",]
V(g.Active)["E_8"]$color <- cluster.colours.k10.df["8",]
V(g.Active)["E_1"]$color <- cluster.colours.k10.df["1",]
V(g.Active)["E_9"]$color <- cluster.colours.k10.df["9",]
V(g.Active)["E_4"]$color <- cluster.colours.k10.df["4",]
V(g.Active)["F_2"]$color <- cluster.colours.k10.df["2",]
V(g.Active)["F_8"]$color <- cluster.colours.k10.df["8",]
V(g.Active)["F_6"]$color <- cluster.colours.k10.df["6",]
V(g.Active)["F_4"]$color <- cluster.colours.k10.df["4",]
V(g.Active)["F_1"]$color <- cluster.colours.k10.df["1",]
V(g.Active)["F_9"]$color <- cluster.colours.k10.df["9",]

#Export to Gephi
library("gephi")
gephi_write_edges(g.Active, "gephi_edges-active.csv")

adjlist <- data.frame(from = adjmatrix.ML.eT.P$EveClust[-
length(adjmatrix.ML.eT.P$EveClust)],
                      to = adjmatrix.ML.eT.P$EveClust[-1],
                      idsame = ifelse(adjmatrix.ML.eT.P$ID[-
length(adjmatrix.ML.eT.P$ID)] == adjmatrix.ML.eT.P$ID[-1], TRUE,
FALSE))
#unlink end of ID1 and beginning of ID2
adjlist <- subset(adjlist, adjlist$idsame == TRUE)
adjlist$idsame <- NULL
head(adjlist)
library("igraph")
gel.Placebo <- graph.data.frame(adjlist)
gad.Placebo <- get.adjacency(gel.Placebo, sparse=FALSE)
g.Placebo <- graph_from_adjacency_matrix(gad.Placebo, mode =
"directed", weighted = TRUE)
table(adjmatrix.ML.eT.P$EveClust)
V(g.Placebo)
V(g.Placebo)$size <- c(3, 5, 6, 16, 6, 4, 1, 1, 2, 8, 5, 1, 5, 1, 1,
1, 9, 8, 3, 1, 1, 3, 3, 1)
V(g.Placebo)["B_5"]$color <- cluster.colours.k10.df["5",]
V(g.Placebo)["B_2"]$color <- cluster.colours.k10.df["2",]
V(g.Placebo)["B_8"]$color <- cluster.colours.k10.df["8",]
V(g.Placebo)["B_6"]$color <- cluster.colours.k10.df["6",]
V(g.Placebo)["B_3"]$color <- cluster.colours.k10.df["3",]
V(g.Placebo)["B_1"]$color <- cluster.colours.k10.df["1",]
V(g.Placebo)["B_9"]$color <- cluster.colours.k10.df["9",]
V(g.Placebo)["B_7"]$color <- cluster.colours.k10.df["7",]
V(g.Placebo)["B_4"]$color <- cluster.colours.k10.df["4",]
V(g.Placebo)["E_5"]$color <- cluster.colours.k10.df["5",]
V(g.Placebo)["E_2"]$color <- cluster.colours.k10.df["2",]
V(g.Placebo)["E_3"]$color <- cluster.colours.k10.df["3",]
```

````
V(g.Placebo)["E_8"]$color <- cluster.colours.k10.df["8",]
V(g.Placebo)["E_1"]$color <- cluster.colours.k10.df["1",]
V(g.Placebo)["E_9"]$color <- cluster.colours.k10.df["9",]
V(g.Placebo)["E_7"]$color <- cluster.colours.k10.df["7",]
V(g.Placebo)["F_2"]$color <- cluster.colours.k10.df["2",]
V(g.Placebo)["F_3"]$color <- cluster.colours.k10.df["3",]
V(g.Placebo)["F_8"]$color <- cluster.colours.k10.df["8",]
V(g.Placebo)["F_6"]$color <- cluster.colours.k10.df["6",]
V(g.Placebo)["F_7"]$color <- cluster.colours.k10.df["7",]
V(g.Placebo)["F_4"]$color <- cluster.colours.k10.df["4",]
V(g.Placebo)["F_1"]$color <- cluster.colours.k10.df["1",]
V(g.Placebo)["F_9"]$color <- cluster.colours.k10.df["9",]

#Export to Gephi
library("gephi")
gephi_write_edges(g.Placebo, "gephi_edges-placebo.csv")
```

Patchwork, Sup Figure 9
```{r}
layout = 'AB'
patch3 <-
  pe.BF +
  p.FwwoE +
  plot_layout(design = layout) +
  plot_annotation(tag_levels = 'A')
patch3

pdf("figures/SupFigure9.pdf", height=7, width=16)
print(patch3)
dev.off()
```

# Sup Figure 7: Respiratory events are difficult to predict
(qd.event B samples)

A. Is there a difference in B's from individuals who did or did not
have E's?
```{r}
qd.event.B <- subset_samples(qd.event, Type.B.E.F. == "B")
qd.event.B.rare = rarefy_even_depth(qd.event.B, sample.size =
min(sample_sums(qd.event.B)), rngseed=1414)
#Setup distance matrix
df.event.B = as(sample_data(qd.event.B.rare), "data.frame")
d.event.B = phyloseq::distance(qd.event.B.rare, "bray")
#Setup plotting backend
dist.event.B <- phyloseq::distance(qd.event.B.rare, method = "bray")
#calc dist matrix

qd.event.B.clr <- microbiome::transform(qd.event.B, "clr")
df.ait.event.B = as(sample_data(qd.event.B.clr), "data.frame")
dist.ait.event.B <- phyloseq::distance(qd.event.B.clr, method =
"euclidean")
````

```
a.BwwoE = adonis(d.event.B ~ hadEvent, df.event.B)
a.BwwoE
ait.BwwoE = adonis(dist.ait.event.B ~ hadEvent, df.ait.event.B)
ait.BwwoE

p.BwwoE <- plot_ordination(qd.event.B.rare, axes=c(1,2), iMDS) +
  ggtitle(paste("p=",a.BwwoE[[1]][["Pr(>F)"]][1],"/",ait.BwwoE[[1]]
[["Pr(>F)"]][1])) +
  theme_bw() +
  theme(legend.title=element_blank(),
        legend.position = c(0.87,0.1)) +
  geom_point(size = 2, colour="gray") +
  geom_point(size = 3, aes(colour=hadEvent)) +
  scale_colour_manual(na.translate = F,
                        values=c("T" = "#689F38",
                                 "F" = "#B0C15A"),
                      labels = c("T" = "had ILI",
                                   "F" = "did not have ILI"))
p.BwwoE
```

B. Is there a difference in B's from individuals who did or did not have E's at the cluster level?

```{r}
#Subset qd.event to only those individuals who had an Event
qd.event.clust.sam.b <-
qd.event.clust.sam[qd.event.clust.sam$Type.B.E.F. == "B",]
#Count frequency of each cluster by timepoint
#chi-squared
tbl.ev <- table(qd.event.clust.sam.b$hadEvent,
qd.event.clust.sam.b$cluster)
tbl.ev
chi <- chisq.test(tbl.ev) #p=0.36228
chi
contrib <- 100*chi$residuals^2/chi$statistic
corrplot(contrib, is.cor=FALSE)
corrplot(chi$residuals, is.cor=FALSE)

counts.hadEvent <- qd.event.clust.sam %>% dplyr::select(hadEvent,
Type.B.E.F., cluster)
counts.hadEvent = counts.hadEvent %>%
  group_by(hadEvent, Type.B.E.F., cluster,
           .drop=FALSE) %>%
    tally()
counts.hadEvent
counts.hadEvent <- counts.hadEvent %>% filter(Type.B.E.F. == "B")
p.clustBwwoE <- ggplot(counts.hadEvent, aes(x=hadEvent, y=n,
fill=cluster)) +
  geom_bar(position="dodge", stat="identity") +
  scale_fill_manual(values=c(cluster.colours.k10)) +
  theme_bw() +
  theme(legend.position="none") +
  xlab("Boolean: had ILI") + ylab("# of Samples per cluster") +
  ggtitle("pre-C&F samples (p=0.121349)")
```

```
p.clustBwwoE
```

Patchwork, Sup Figure 7
```{r}
### Put Figure together ###
layout = '
AB
'
patchwork.Fig5 <- p.BwwoE + p.clustBwwoE +
  plot_layout(design = layout) +
  plot_annotation(tag_levels = 'A')
patchwork.Fig5
pdf("figures/SupFigure7.pdf", height=6, width=14)
print(patchwork.Fig5)
dev.off()
```

Figure 5 (rest of): Do probiotics have an effect on respiratory events? (qd.event)

Define colours for probiotic groups
```{r}
probiotic.colours <- c("Active" = "#536DFE",
                      "Placebo" = "#607D8B")
```

A. How many participants had Events?
```{r}
df.event = sample_data(qd.event)
tmp = df.event[,c(4,17,20)]
head(tmp)
tmp <- tmp[tmp$hadEvent=="T",]
head(tmp)
tmp <- tmp[!duplicated(tmp[,c(1)]),]
head(tmp)
counts.w = tmp %>%
  group_by(Allocation_Group_Probiotics) %>%
  tally()
counts.w
nrow(counts.w)
#without
df.event = sample_data(qd.event)
tmp = df.event[,c(4,17,20)]
head(tmp)
tmp <- tmp[tmp$hadEvent=="F",]
head(tmp)
tmp <- tmp[!duplicated(tmp[,c(1)]),]
head(tmp)
counts = tmp %>%
  group_by(Allocation_Group_Probiotics) %>%
  tally()
counts
#chi.sq test: is there a correlation between who has RE and who is
```

~~~
on probiotic?
tbl <- data.frame(c(31,74), c(41,57))
rownames(tbl) <- c("withRE", "woRE")
colnames(tbl) <- c("Active", "Placebo")
tbl
chisq.test(tbl)

p.PAcounts <- ggplot(data=counts.w,
aes(x=Allocation_Group_Probiotics, y=n,
fill=Allocation_Group_Probiotics)) +
  geom_bar(stat="identity") +
  xlab("Probiotic grouping") + ylab("# of individuals with ILI
events") +
  scale_fill_manual(values=c(probiotic.colours)) +
  theme_bw() +
  theme(legend.position = "none") +
  ggtitle("p=0.0918757")
```

B. Number of events per individual
```{r}
tmp = df.event[,c(4,17,19,20)]
head(tmp)
tmp <- tmp[tmp$hadEvent=="T",]
tmp <- tmp[tmp$Type.B.E.F.=="E",]
head(tmp)
counts = tmp %>%
  group_by(ID, Allocation_Group_Probiotics) %>%
  tally()
counts

#t-test
res.ftest <- var.test(n ~ Allocation_Group_Probiotics, data =
counts)
res.ftest #NS between variances
actives <- subset(counts, Allocation_Group_Probiotics == "Active")
placebos <- subset(counts, Allocation_Group_Probiotics == "Placebo")
res <- t.test(actives$n, placebos$n, var.equal = TRUE)
res

p.PAperind <- ggplot(data=counts, aes(x=Allocation_Group_Probiotics,
y=n, colour=Allocation_Group_Probiotics)) +
  geom_jitter(alpha=0.4, width=0.3, height=0) +
  xlab("Probiotic grouping") + ylab("# of ILI per individual") +
  scale_colour_manual(values=c(probiotic.colours)) +
  scale_fill_manual(values=c(probiotic.colours)) +
  theme_bw() +
  theme(legend.position = "none") +
  ggtitle("p=0.588504")
p.PAperind
```

C. Is the Event microbiota statistically different between
participants on and off the probiotic?
~~~

```{r}
qd.events.Events <- subset_samples(qd.event, Type.B.E.F. == "E")
qd.events.Events.rare = rarefy_even_depth(qd.events.Events,
sample.size = min(sample_sums(qd.events.Events)), rngseed=1414)
#Setup distance matrix
df.events.Events = as(sample_data(qd.events.Events.rare),
"data.frame")
d.events.Events = phyloseq::distance(qd.events.Events.rare, "bray")
#Setup plotting backend
dist.events.Events <- phyloseq::distance(qd.events.Events.rare,
method = "bray") #calc dist metrix

qd.events.Events.clr <- microbiome::transform(qd.events.Events,
"clr")
df.ait.events.Events = as(sample_data(qd.events.Events.clr),
"data.frame")
dist.ait.events.Events <- phyloseq::distance(qd.events.Events.clr,
method = "euclidean")

##Adonis
a.Event = adonis(d.events.Events ~ Allocation_Group_Probiotics - ID,
df.events.Events)
a.Event
ait.Event = adonis(dist.ait.events.Events ~
Allocation_Group_Probiotics - ID, df.ait.events.Events)
ait.Event

p.PAE <- plot_ordination(qd.events.Events.rare, axes=c(1,2), iMDS) +
  ggtitle(paste("p=",a.Event[[1]][["Pr(>F)"]][1],"/",ait.Event[[1]]
[["Pr(>F)"]][1])) +
  theme_bw() +
  theme(legend.title=element_blank(),
        legend.position = c(0.9,0.15)) +
  geom_point(size = 2, colour = "gray") +
  geom_point(size = 3, aes(colour=Allocation_Group_Probiotics)) +
  scale_colour_manual(na.translate = F,
                      values=c(probiotic.colours))
```

D. Is the Followup microbiota statistically different between participants on and off the probiotic?

```{r}
qd.events.F <- subset_samples(qd.event, Type.B.E.F. == "F")
qd.events.F.rare = rarefy_even_depth(qd.events.F, sample.size =
min(sample_sums(qd.events.F)), rngseed=1414)
#Setup distance matrix
df.events.F = as(sample_data(qd.events.F.rare), "data.frame")
d.events.F = phyloseq::distance(qd.events.F.rare, "bray")
#Setup plotting backend
dist.events.F <- phyloseq::distance(qd.events.F.rare, method =
"bray") #calc dist matrix

qd.events.F.clr <- microbiome::transform(qd.events.F, "clr")
df.ait.events.F = as(sample_data(qd.events.F.clr), "data.frame")
```

~~~
dist.ait.events.F <- phyloseq::distance(qd.events.F.clr, method =
"euclidean")

##Adonis
a.PAF = adonis(d.events.F ~ Allocation_Group_Probiotics,
df.events.F)
a.PAF
ait.PAF = adonis(dist.ait.events.F ~ Allocation_Group_Probiotics,
df.ait.events.F)
ait.PAF

p.PAF <- plot_ordination(qd.events.F.rare, axes=c(1,2), iMDS) +
  ggtitle(paste("p=",a.PAF[[1]][["Pr(>F)"]][1],"/",ait.PAF[[1]]
[["Pr(>F)"]][1])) +
  theme_bw() +
  theme(legend.title=element_blank(),
        legend.position = c(0.9,0.15)) +
  geom_point(size = 2, colour = "gray") +
  geom_point(size = 3, aes(colour=Allocation_Group_Probiotics)) +
  scale_colour_manual(na.translate = F,
                      values=c(probiotic.colours))
```
~~~

E. Are individuals on probiotics more likely to return to their previous cluster?

~~~
```{r}
colnames(indiv.pcoa.data)
perid <- indiv.pcoa.data[c(6,7,10, 11, 12, 15, 16, 19, 44, 48, 55,
116, 115)] #ID, JJ_ML,Barthel_Total, cluster_movement
colnames(perid)
perid.uniq <- perid[!duplicated(perid$ID),]
perid.uniq <- subset(perid.uniq, !is.na(perid.uniq$Barthel_Total))
perid.uniq <- subset(perid.uniq, cluster_movement != "na")
perid.uniq <- subset(perid.uniq, perid.uniq$JJ_ML != "JJ")
perid.uniq$cluster_movement <- factor(perid.uniq$cluster_movement,
                                      levels=c("same","change",

"eventsame","eventreturn","eventchange","followchange"))
perid.uniq <- subset(perid.uniq, cluster_movement != "same")
perid.uniq <- subset(perid.uniq, cluster_movement != "change")

probmove.ev <- perid.uniq %>%
dplyr::select(Allocation_Group_Probiotics, cluster_movement)
probmove.ev <- probmove.ev %>%
  group_by(Allocation_Group_Probiotics, cluster_movement) %>%
  summarize(n = n())
p.PAclust <- ggplot(probmove.ev, aes(x=cluster_movement, y=n,
fill=Allocation_Group_Probiotics)) +
  geom_bar(position="dodge", stat="identity") +
    theme(axis.title.x=element_blank(),
        axis.text.x = element_text(angle = 0),
        legend.position = c(0.905, 0.9)) +
  scale_fill_manual(values=c(probiotic.colours),
                    name = "") +
~~~

````
  scale_x_discrete(labels=c("eventsame" = "No cluster movement (1)",
                            "eventreturn" = "Change with ILI,
\nreturn to baseline (2)",
                            "eventchange" = "Change with ILI,\nno
return to baseline (3)",
                            "followchange" = "Change following ILI
(4)")) +
  ylab("# of individuals with ILI")
p.PAclust

#Statistics between same vs. change
perid.uniq$cluster_movement_simple <- ifelse(grepl('same',
perid.uniq$cluster_movement), "same", "change")
perid.uniq

table(perid.uniq$cluster_movement_simple,
perid.uniq$Allocation_Group_Probiotics)
table(perid.uniq$cluster_movement,
perid.uniq$Allocation_Group_Probiotics)
chisq <- chisq.test(x=perid.uniq$cluster_movement_simple,
y=perid.uniq$Allocation_Group_Probiotics)
chisq
corrplot(chisq$residuals, is.cor = FALSE)

p <- ggplot(data = perid.uniq, aes(x=cluster_movement_simple,
fill=Allocation_Group_Probiotics)) +
  geom_bar(stat="count")

#Proportion of individuals in each group
temp <- data.frame(change= c(57.1,85.7), Allocation_Group_Probiotics
= c("Active", "Placebo"))
temp
plot.movement.proportions <- ggplot(temp,
aes(x=Allocation_Group_Probiotics, y=change,
fill=Allocation_Group_Probiotics)) +
  geom_bar(stat="identity") +
  ylab("% of individuals with cluster movement") + xlab("") +
  scale_y_continuous(limit=c(0,100)) +
  ggtitle("p=0.096") +
  scale_fill_manual(values=c(probiotic.colours)) +
  theme(legend.position = "none")
plot.movement.proportions
```

F. Is there a difference in E's by probiotic grouping by cluster?
```{r}
#Subset qd.event to only those individuals who had an Event
qd.event.clust.sam.e <-
qd.event.clust.sam[qd.event.clust.sam$Type.B.E.F. == "E",]
#Count frequency of each cluster by timepoint
#chi-squared
tbl.ev <- table(qd.event.clust.sam.e$Allocation_Group_Probiotics,
qd.event.clust.sam.e$cluster)
tbl.ev
````

```
chi <- chisq.test(tbl.ev) #p=0.9492
chi

#Plot
counts.placebo <- qd.event.clust.sam %>%
dplyr::select(Allocation_Group_Probiotics, Type.B.E.F., cluster)
counts.placebo <- counts.placebo %>%
filter(Allocation_Group_Probiotics != ".") #remove JJs
counts.placebo <- counts.placebo %>% filter(Type.B.E.F. == "E")
#only look at Events, prior to probiotic exposure
counts.placebo = counts.placebo %>%
  group_by(Allocation_Group_Probiotics, cluster,
           .drop = FALSE) %>%
    tally()
counts.placebo
p.clustPAE <- ggplot(counts.placebo,
aes(x=Allocation_Group_Probiotics, y=n, fill=cluster)) +
  geom_bar(position="dodge", stat="identity") +
  scale_fill_manual(values=c(cluster.colours.k10)) +
  theme_bw() +
  theme(legend.position="none") +
  xlab("Probiotic grouping") + ylab("# samples per cluster") +
  ggtitle("ILI samples (p=0.220009)")
p.clustPAE
```

G. Is there a difference in F's by probiotic grouping by cluster?

```{r}
#Subset qd.event to only those individuals who had an Event
qd.event.clust.sam.f <-
qd.event.clust.sam[qd.event.clust.sam$Type.B.E.F. == "F",]
#Count frequency of each cluster by timepoint
#chi-squared
tbl.ev <- table(qd.event.clust.sam.f$Allocation_Group_Probiotics,
qd.event.clust.sam.f$cluster)
tbl.ev
chi <- chisq.test(tbl.ev) #p=0.2374
chi

#Plot
counts.placebo <- qd.event.clust.sam %>%
dplyr::select(Allocation_Group_Probiotics, Type.B.E.F., cluster)
counts.placebo <- counts.placebo %>%
filter(Allocation_Group_Probiotics != ".")
counts.placebo <- counts.placebo %>% filter(Type.B.E.F. == "F")
#only look at Followups, prior to probiotic exposure
counts.placebo = counts.placebo %>%
  group_by(Allocation_Group_Probiotics, cluster,
           .drop = FALSE) %>%
    tally()
counts.placebo
p.clustPAF <- ggplot(counts.placebo,
aes(x=Allocation_Group_Probiotics, y=n, fill=cluster)) +
  geom_bar(position="dodge", stat="identity") +
```

````
  scale_fill_manual(values=c(cluster.colours.k10)) +
  theme_bw() +
  theme(legend.position="none") +
  xlab("Probiotic grouping") + ylab("# samples per cluster") +
  ggtitle("post-C&F samples (p=0.178156)")
p.clustPAF
```

Patchwork, Figure 5:
```{r}
### Put Figure together ###
layout = '
ABCD
EFFG
'
patchwork.Fig6 <- p.PAcounts +
  #p.PAperind +
  p.PAE + #p.PAE +
  p.clustPAE +
  p.PAF + #p.PAF +
  p.clustPAF +
  p.PAclust +
  plot.movement.proportions +

  plot_layout(design = layout) +
  plot_annotation(tag_levels = 'A')
patchwork.Fig6
pdf("figures/Figure5.pdf", height=9, width=17)
print(patchwork.Fig6)
dev.off()
```

# Sup Figure 12: Relative abundance of Lactobacillus genera in
Active/Placebo Followup samples

```{r}
psmelt.event.L6 = psmelt(qd.event.L6.norm)
df.sub = subset(psmelt.event.L6, Genus == "Lactobacillus")
p.Lactobacillus1 = ggplot(data=df.sub,
aes(x=Allocation_Group_Probiotics, y=Abundance, color=Type.B.E.F.))
+

geom_point(position=position_dodge(width=0.75),aes(group=Type.B.E.F.
)) +
  geom_boxplot(aes(x = Allocation_Group_Probiotics, color =
Type.B.E.F.), alpha=0.1) +
  ggtitle("Abundance of genus Lactobacillus") + #title
  scale_y_log10() +
  xlab("Sample type") + ylab("log(Abundance)") +
  scale_x_discrete(labels=c("B" = paste("B (n=",
length(which(df.sub$Type.B.E.F. == "B")), ")", sep=""),
                            "E" = paste("E (n=",
length(which(df.sub$Type.B.E.F. == "E")), ")", sep=""),
                            "F" = paste("F (n=",
````

length(which(df.sub$Type.B.E.F. == "F")), ")", sep=""))) +
  theme_bw() +
  theme(legend.position="none") +
  scale_colour_manual(values=type.colours)

pdf("figures/SupFigure12.pdf", height=8, width=10)
print(p.Lactobacillus1)
dev.off()
```
Do any Lactobacillus ASVs DA between Active and Placebo individuals?
```{r}
#ANCOMBC
ancom_da = ancombc(phyloseq = qd, formula =
"Allocation_Group_Probiotics",
              p_adj_method = "holm", zero_cut = 0.90, lib_cut =
1000,
              group = "Allocation_Group_Probiotics", struc_zero =
TRUE, neg_lb = FALSE, tol = 1e-5,
              max_iter = 100, conserve = TRUE, alpha = 0.05, global
= TRUE)
res = ancom_da$res

#Visualize log fold change
samp_frac = ancom_da$samp_frac
# Replace NA with 0
samp_frac[is.na(samp_frac)] = 0
# Add pesudo-count (1) to avoid taking the log of 0
log_obs_abn = log(abundances(qd) + 1)
# Adjust the log observed abundances
log_obs_abn_adj = t(t(log_obs_abn) - samp_frac)
head(log_obs_abn_adj)

df_fig1 = data.frame(res$beta * res$diff_abn, check.names = FALSE)
%>%
  rownames_to_column("taxon_id")
df_fig2 = data.frame(res$se * res$diff_abn, check.names = FALSE) %>%
  rownames_to_column("taxon_id")
colnames(df_fig2)[-1] = paste0(colnames(df_fig2)[-1], "SD")
df_fig = df_fig1 %>% left_join(df_fig2, by = "taxon_id") %>%
  transmute(taxon_id, Allocation_Group_ProbioticsPlacebo,
Allocation_Group_ProbioticsPlaceboSD) %>%
  filter(Allocation_Group_ProbioticsPlacebo != 0) %>%
arrange(desc(Allocation_Group_ProbioticsPlacebo)) %>%
  mutate(group = ifelse(Allocation_Group_ProbioticsPlacebo > 0,
"g1", "g2"))
head(df_fig)
nrow(df_fig)
#0 diff_abn taxa
```

Sup Figure 10: Number of overlapping ASVs between B/F pairs in same and different clusters after an event

i.e. mean beta-diversity distance between individuals
```

```{r}
#Calculate beta-diversity by individual
qd.bray.dist = phyloseq::distance(qd.rare, method="bray")
qd.bray.dist.df = melt(as.matrix(qd.bray.dist))
df <- as.data.frame(as.matrix(sample_data(qd)[,c(1,4)])) #SampleId =
1; ID=4
head(df)
qd.bray.dist.df <- merge(qd.bray.dist.df, df, by.x="Var1",
by.y="SampleID")
head(qd.bray.dist.df)
qd.bray.dist.df$ID_Var1 <- qd.bray.dist.df$ID
qd.bray.dist.df <- merge(qd.bray.dist.df, df, by.x="Var2",
by.y="SampleID")
head(qd.bray.dist.df)
qd.bray.dist.df$ID_Var2 <- qd.bray.dist.df$ID.y
qd.bray.dist.df$ID.x <- NULL
qd.bray.dist.df$ID.y <- NULL
qd.bray.dist.df.IDs <- subset(qd.bray.dist.df, as.character(Var1) <
as.character(Var2)) #only want sample1 to sample2 not sample2 to
sample1 comparisons
qd.bray.dist.df.IDs <- subset(qd.bray.dist.df.IDs, ID_Var1 ==
ID_Var2) #only want within individual comparisons
qd.bray.dist.df.IDs <- subset(qd.bray.dist.df.IDs, Var1 != Var2)
#don't want self to self comparisons
length(unique(qd.bray.dist.df.IDs$ID_Var1))
nrow(qd.bray.dist.df.IDs)
head(qd.bray.dist.df.IDs)
#calculate the mean for each individual
per.indiv.means <- aggregate( value ~ ID_Var1, qd.bray.dist.df.IDs,
mean )
df <- as.data.frame(as.matrix(sample_data(qd.baseline)[,c(4,113)]))
#ID =4; cluster_movement=111
qd.bray.dist.df.IDs <- merge(per.indiv.means, df, by.x="ID_Var1",
by.y="ID", all.x=TRUE)
nrow(qd.bray.dist.df.IDs)
head(qd.bray.dist.df.IDs)
qd.bray.dist.df.IDs <- subset(qd.bray.dist.df.IDs,
cluster_movement != "na")
nrow(qd.bray.dist.df.IDs)
qd.bray.dist.df.IDs$cluster_movement <-
factor(qd.bray.dist.df.IDs$cluster_movement,
                                               levels = c("same",
"change", "eventsame", "eventreturn", "eventchange",
"followchange"))

p.dist.move <- ggplot(data=qd.bray.dist.df.IDs,
aes(x=cluster_movement, y=value)) +
  geom_boxplot() +
  geom_point() + #position="jitter", alpha=0.2) +
  ggtitle("Bray curtis distance within an individual per cluster
movement group") +
      theme(axis.title.x=element_blank(),
        axis.text.x = element_text(angle = 0, hjust = 0.5)) +
  scale_x_discrete(labels=c("same" = "Same",
```

```
                              "change" = "Change",
                              "eventsame" = "No cluster movement (1)",
                              "eventreturn" = "Change with ILI,
\nreturn to baseline (2)",
                              "eventchange" = "Change with ILI,\nno
return to baseline (3)",
                              "followchange" = "Change following ILI
(4)")) +
  ylab("Bray curtis distance") +
  geom_signif(
              xmin=c(1.0, 4.0, 1.0, 1.0),
                                xmax=c(2.0, 5.0, 5.0, 6.0),
                                y_position=c(1.05, 1.05, 1.1, 1.15),
                                annotation=c("***","*","***","**")) +
  scale_y_continuous(limits=c(0,1.5))
p.dist.move
pdf("figures/SupFigure10.pdf", height=8, width=10)
print(p.dist.move)
dev.off()

#Statistical difference between category 1 vs. 2, 1 vs. 3, 1 vs.4?
#t-test
head(qd.bray.dist.df.IDs)
same <- subset(qd.bray.dist.df.IDs, cluster_movement == "same")
chan <- subset(qd.bray.dist.df.IDs, cluster_movement == "change")
cat1 <- subset(qd.bray.dist.df.IDs, cluster_movement == "eventsame")
cat2 <- subset(qd.bray.dist.df.IDs, cluster_movement ==
"eventreturn")
cat3 <- subset(qd.bray.dist.df.IDs, cluster_movement ==
"eventchange")
cat4 <- subset(qd.bray.dist.df.IDs, cluster_movement ==
"followchange")
res <- t.test(cat1$value, cat2$value, var.equal = TRUE)
res
res <- t.test(cat1$value, cat3$value, var.equal = TRUE)
res
res <- t.test(cat1$value, cat4$value, var.equal = TRUE)
res
res <- t.test(cat2$value, cat3$value, var.equal = TRUE)
res
res <- t.test(cat2$value, cat4$value, var.equal = TRUE)
res
res <- t.test(cat3$value, cat4$value, var.equal = TRUE)
res
res <- t.test(same$value, chan$value, var.equal = TRUE)
res
res <- t.test(same$value, cat1$value, var.equal = TRUE)
res
res <- t.test(same$value, cat2$value, var.equal = TRUE)
res
res <- t.test(same$value, cat3$value, var.equal = TRUE)
res
res <- t.test(same$value, cat4$value, var.equal = TRUE)
res
```

```
res <- t.test(chan$value, cat1$value, var.equal = TRUE)
res
res <- t.test(chan$value, cat2$value, var.equal = TRUE)
res
res <- t.test(chan$value, cat3$value, var.equal = TRUE)
res
res <- t.test(chan$value, cat4$value, var.equal = TRUE)
res
```
